# Cereblon-recruiting proteolysis targeting chimeras (PROTACs) can determine the selective degradation of HDAC1 over HDAC3†

**DOI:** 10.1039/d4cc05138f

**Published:** 2024-11-05

**Authors:** Aline R. Pavan, Joshua P. Smalley, Urvashi Patel, Wiktoria A. Pytel, Jean Leandro dos Santos, Shaun M. Cowley, John W. R. Schwabe, James T. Hodgkinson

**Affiliations:** a Leicester Institute of Structural and Chemical Biology and School of Chemistry, University of Leicester University Road Leicester LE1 7RH UK JTHodgkinson@le.ac.uk; b São Paulo State University (UNESP), School of Pharmaceutical Sciences Araraquara Brazil jean.santos@unesp.br; c A Department of Molecular and Cell Biology, University of Leicester Leicester LE1 9HN UK smc57@leicester.ac.uk; d Leicester Institute of Structural and Chemical Biology and Department of Molecular and Cell Biology, University of Leicester Leicester LE1 9HN UK john.schwabe@le.ac.uk

## Abstract

Histone deacetylase (HDAC) enzymes 1–3 exist in several corepressor complexes and are viable drug targets. To date, proteolysis targeting chimeras (PROTACs) designed to target HDAC1–3 typically exhibit the selective degradation of HDAC3. Herein, we report cereblon-recruiting PROTACs that degrade HDAC1 with selectivity over HDAC3.

## Abstract 2

Cereblon-recruiting PROTACs can degrade HDAC1 with selectivity over HDAC3.

## Graphical abstract

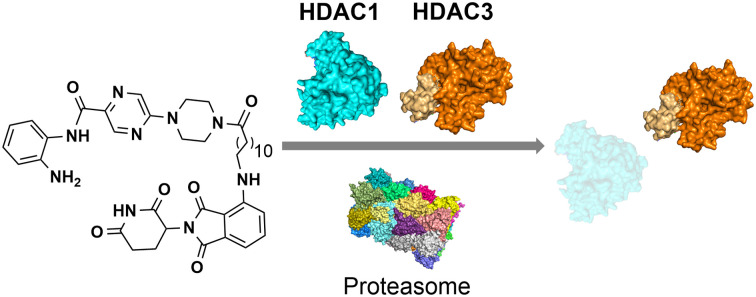

Proteolysis targeting chimeras (PROTACs) are heterobifunctional molecules that consist of a ligand for the protein of interest (POI), an E3-ligase ligand and a linker covalently bonding these two ligands together (Fig. 1).^1^ In the cell, PROTACs have the potential to recruit the POI and E3-ligase into an artificially induced protein–protein interaction and ternary complex.^2^ PROTACs that consist of the optimal components, in terms of linker length, linker composition, choice of POI ligand and choice of E3-ligand result in transfer of ubiquitin from the E2 ubiquitin-conjugating enzyme recruited by the E3-ligase to the POI. Subsequent poly-ubiquitination of the POI leads to its ‘tagging’ for degradation by the proteasome. Due to the potential advantages that can be achieved by PROTAC-mediated degradation, sometimes referred to as ‘event driven pharmacology’,^3^ PROTACs have received copious attention in drug discovery, with over 20 PROTACs currently in clinical trials.^4^ Additionally, in the field of chemical biology PROTACs offer an alternative strategy *via* their degradation mode of action to study proteins that have previously been more challenging to profile with small molecule inhibitors.^5^ One such family of proteins, directly relevant to this, are Histone deacetylase (HDAC) enzymes. In total, 18 different HDAC isoforms exist in humans.

**Fig. 1:**
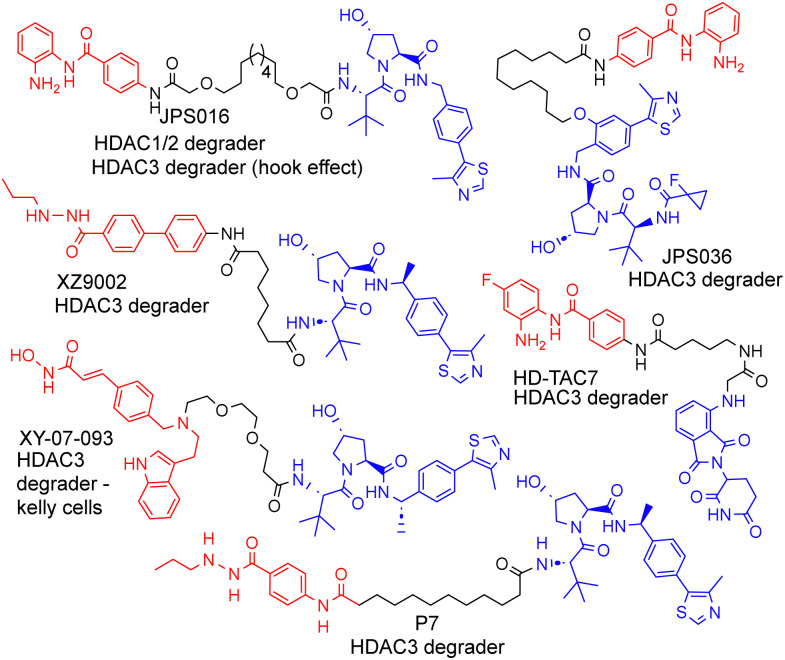
Selected examples of PROTACs designed to target HDAC1–3, most of which exhibit the selective degradation of HDAC3.^6–9^ HDAC ligand highlighted in red, linker in black and E3-ligand in blue for each PROTAC.

We and others have been investigating the development of PROTACs that target HDAC1, HDAC2 and HDAC3 for degradation.^6–13^ HDAC1–3 exist *in vivo* in seven multi-protein corepressor complexes and play an important role in influencing chromatin structure and gene transcription.^14^ HDAC1 and HDAC2, with approximately 86% amino acid sequence homology, can exist interchangeably in the corepressor complexes MiDAC, NuRD, CoREST, SIN3, RERE and MIER, while HDAC3 exists in the SMRT/NCoR complex.^14^ These HDACs and their corepressor complexes are also important drug targets for a number of diseases.^15^

As far as we are aware, there have been no PROTACs reported to date that exhibit the selective degradation of HDAC1/2. Aside from HD-TAC7, PROTACs reported in the literature designed to target HDAC1–HDAC3 incorporate the VHL E3-ligand (Fig. 1).^6–13^ Importantly, nearly all these PROTACs are more effective and selective degraders of HDAC3 over HDAC1/2.

These results also correlate with our previous findings, while PROTACs such as JPS016 degrade HDAC1/2 and HDAC3 (hook effect for HDAC3) and PROTAC JPS036 can enhance HDAC3 degradation selectivity over HDAC1/2,^11^ we have not, as of yet, been able to identify PROTACs that exhibit the selective degradation of HDAC1/2 over HDAC3 utilising the VHL ligand. In a proteomics study utilising 48 HDAC targeting PROTACs reported by Xiong *et al.*,^8^ HDAC3 was found to be more prone to proteasome-mediated degradation than most other HDAC isoforms (HDAC6 and HDAC8 the only exceptions), with HDAC1 and HDAC2 some of the least prone HDACs to PROTAC-mediated degradation, highlighting the challenge in targeting HDAC1/2 for degradation over other HDAC isoforms such as HDAC3. Based on our previous findings and those reported in the literature, we hypothesised that utilising the cereblon E3-ligand may be an approach to facilitate HDAC1/2 degradation over HDAC3. With the exception of HD-TAC7 all the HDAC3 selective PROTACs reported to date utilise the VHL E3-liagnd.

In our previous studies we observed that the cereblon recruiting PROTACs, utilising thalidomide analogues in combination with CI-994 as the HDAC1–3 ligand (Fig. 2A) typically exhibited much poorer water solubility compared to VHL based PROTACs.^10^ We hypothesised that if we could improve the physiochemical properties of these cereblon recruiting PROTACs this may also improve their in-cell degradation.

**Fig. 2:**
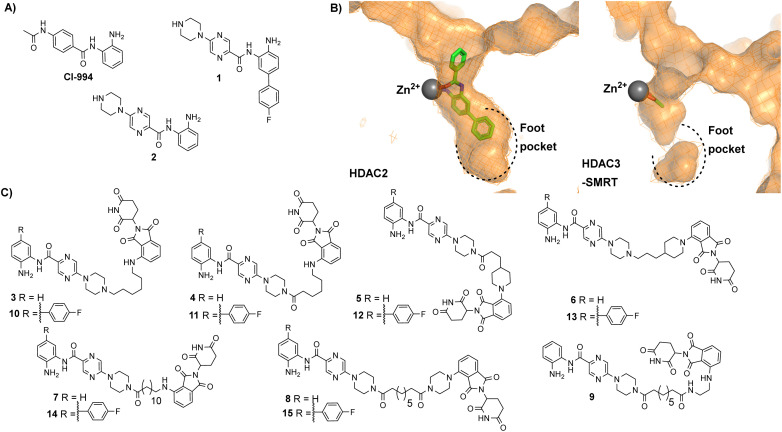
(A) CI-994 was incorporated as the HDAC ligand in our previously reported PROTACs, 1 is a HDAC1 and HDAC2 selective inhibitor reported by Ibrahim *et al.*^16^ and 2 is a direct analogue of 1 without the *para*-fluorine phenyl ring (B) Comparison of HDAC2 active site with the zinc bound to a benzamide inhibitor on the left (PDB: 3MAX) and the HDAC3 active site with acetate bound to the zinc on the right (PDB: 4A69), the *para*-fluorine phenyl ring in 1 has been proposed to occupy the larger foot pocket present in HDAC1/2 (C) A library of potential cereblon-recruiting PROTACs utilising 1 and 2 as the HDAC ligand in attempt to improve the physiochemical properties compared to previous PROTACs utilising CI-994.

We took inspiration from work by Ibrahim *et al.*^16^ and L. Schäker-Hübner *et al.*^17^ in improving the physicochemical properties of benzamide HDAC inhibitors. Ibrahim *et al.* reported compound 1 with IC_50_ values of 0.16 μM, 0.34 μM and 6.7 μM respectively for HDAC1, HDAC2 and HDAC3,^16^ with the HDAC1/2 selectivity over HDAC3 hypothesised to arise from occupation of the larger foot pocket present in HDAC1 and HDAC2 by the *para*-fluorine-phenyl group of 1 (Fig. 2B). We also wanted to synthesise analogues without the *para*-fluorine benzene group for direct comparison (3–9).

We synthesised linkers from 7 atoms to 12 atoms in length, extruding from the terminal nitrogen atom on the piperazine ring (3–15), (Fig. 2C). We hypothesized that the piperazine ring, compared to the acetyl amide group in CI-994, would protrude further from the HDAC active site and would also contribute as a pseudo-linker component. We also investigated linker conjugation *via* amide bond to the piperazine ring or by alkylation. Taking inspiration from current PROTACs in clinical trials such as ARV-110 we also incorporated additional rigid components in some of the PROTAC linkers.

We screened these compounds side-by side with JPS004 a VHL based HDAC1–3 targeting PROTAC and the HDAC1–3 inhibitor CI-994 as control compounds for degradation and inhibition, respectively. PROTACs such as JPS004 with the VHL ligand degrade HDAC1, HDAC2 and HDAC3, however HDAC3 degradation is compromised at concentrations >1 μM due to the hook effect.^11^ HCT116 cells were incubated for 24 hours with the compounds and HDAC1, HDAC2 and HDAC3 abundance was quantified by fluorescence western blotting. We also determined the effects on Histone 3 Lysine 56 acetylation (H3K56ac) levels as a secondary assay, for in-cell HDAC inhibition or degradation. For full synthesis protocols, blots and compound characterisation data see the ESI.† Compounds 3–8, without the *para*-fluorine phenyl group, reduced HDAC1 abundance, with compound 7 causing the greatest HDAC1 degradation at 1 μM (Fig. 3 and Fig. S1, ESI†). Similar to our previous observations with VHL recruiting PROTACs, HDAC2 reduction was less affected. It was noteworthy that 7 also incorporated a 12 carbon alkyl linker, which has been an effective linker in our previous studies.^10,11^ Pleasingly, compounds 3–8 also had more modest effects on HDAC3 abundance compared to previously reported VHL recruiting PROTACs.^6–13^ All these compounds increased H3K56ac levels to greater levels than CI-994 and JPS004 with the exception of 7 (Fig. 3 and Fig. S2, ESI†), which was a surprising result given 7 exhibited the greatest HDAC1 degradation. However, while surprising, it is not unprecedented as we and others have observed similar effects on histone acetylation markers with HDAC3 targeting PROTACs.^11,13,18^ Compounds 9 and 10 had no effects on HDAC1, HDAC2 and HDAC3 levels, demonstrating these compounds do not act as degraders, however they did increase H3K56ac levels to greater levels than DMSO controls (9 greater than 10) indicating they do act as inhibitors in cells. Compounds 11 and 12, that contain the *para*-fluorine-phenyl group, reduced HDAC1 and HDAC2 abundance at 0.1 μM and 1 μM and exhibited no HDAC3 degradation, compound 13 also exhibited a similar degradation selectivity to compounds 11 and 12. Compounds 14 and 15 were less effective at HDAC1 degradation compared to 11 and 12. In general, the presence of the *para*-fluorine phenyl group in 10–15 did reduce HDAC3 degradation; however, nearly all the compounds exhibited minimal HDAC3 degradation perhaps highlighting that the cereblon E3-ligand is the more important factor.

**Fig. 3:**
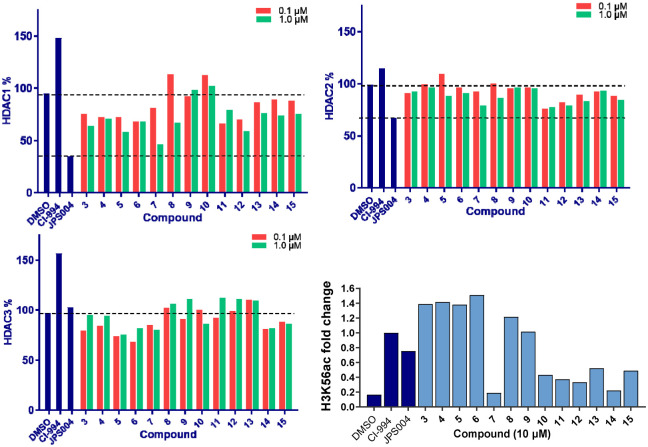
HDAC1, HDAC2, HDAC3 abundance were determined by quantitative western blotting with antibodies for HDAC1, HDAC2, HDAC3 in HCT116 cells after 24 hours. H3K56 acetylation levels were also determined by quantitative western blotting with an antibody for H3K56ac, and the fold change was compared between compounds at 10 μM, normalising treatment with inhibitor CI 994 = 1.0.

We next investigated the effects of 5, 7 and 12 on dose dependent degradation of HDAC1, HDAC2 and HDAC3 (Fig. 4A and Fig. S3, ESI†). 12 was chosen as it exhibited little or no HDAC3 degradation, 5 as a direct comparison to 12 without the *para*-fluorine phenyl group, and 7 was chosen as it exhibited the greatest degradation of HDAC1. 5 exhibited dose dependent degradation of HDAC1 and HDAC2, however a hook effect was observed for HDAC3. HDAC3 abundance was reduced with lower concentrations of 5 but at higher concentrations HDAC3 levels recovered. Introduction of the *para*-fluorine phenyl group in 12 resulted in very similar dose dependent degradation of HDAC1 and HDAC2 to 5, however HDAC3 degradation was abolished and HDAC3 abundance marginally increased in the presence of 12 (an artefact also observed regards HDAC1–3 abundance with the HDAC1–3 inhibitor CI-994, Fig. 3). Surprisingly 7, without the *para*-fluorine phenyl group, also exhibited minimal HDAC3 degradation with clear dose dependent HDAC1 degradation observed. Importantly, unlike VHL recruiting PROTACs, 7 and 12 exhibited minimal degradation of HDAC3 at all concentrations tested.

**Fig. 4:**
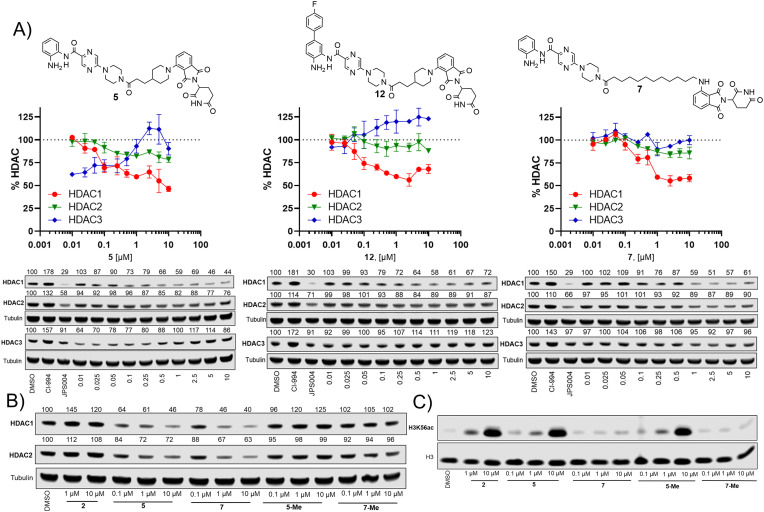
(A) Dose response degradation of HDAC1, HDAC2, HDAC3 in the presence of 5, 7 and 12 in HCT116 cells after 24 h. HDAC1, HDAC2, HDAC3 abundance were determined by quantitative western blotting with antibodies for HDAC1, HDAC2, HDAC3. Error bars represent the variation between two independent biological replicates. (B) HDAC1 and HDAC2 degradation is compromised in the presence of 5-Me and 7-Me that do not bind the cereblon E3-ligase. (C) H3K56 acetylation levels were determined in the presence of 2, 5, 7, 5-Me and 7-Me by quantitative western blotting with an antibody for H3K56ac.

We also synthesised the methylated thalidomide analogues of 5 and 7, 5-Me and 7-Me which should compromise binding affinity for the cereblon E3-ligase and degradation (see ESI† for structures and synthesis), we were pleased to observe no HDAC1 or HDAC2 degradation with 5-Me and 7-Me (Fig. 4B), providing evidence that these PROTACs are recruiting the cereblon E3-ligase for degradation. We also screened these compounds again for their effects on H3K56 acetylation (Fig. 4C), with 5 increasing H3K56ac levels as observed in the primary screen.

Interestingly, the 5-Me analogue also increased H3K56ac despite no HDAC1 or HDAC2 degradation. 5-Me would still be expected to act as a HDAC1–3 inhibitor and this finding suggests that the increases observed in H3K56ac levels are more influenced by HDAC inhibitory effects than the effects of HDAC degradation. 7 did not increase H3K56 acetylation levels as observed previously and neither did the 7-Me analogue as expected.

Here, we report the first PROTACs 7 and 12 that exhibit the selective degradation of HDAC1 over HDAC3 by utilising the cereblon E3-ligand. Given HDAC3 has been reported to be more susceptible to proteasome mediated degradation by PROTACs over other HDAC isoforms this is a significant finding. Intriguingly, despite clear HDAC1 degradation 7 did not increase H3K56ac levels, however we and others have also observed similar effects with HDAC3 selective PROTACs.^11,13,18^5-Me which does not degrade HDAC1/2 also increased H3K56ac levels similar to 5, suggesting the observed increases in the H3K56ac with 5 and potentially other PROTACs are due to HDAC inhibition rather than PROTAC-mediated degradation. Hence, we speculate degraders such as 7 and other selective HDAC targeting PROTACs may result in less pronounced effects on certain histone acetylation markers due to their selectivity of degradation and/or are potentially poor inhibitors but effective degraders. These observed effects on H3K56ac are also important findings because as we and others develop more selective PROTACs for individual HDAC isoforms and potentially each of the seven HDAC1–3 containing corepressor complexes we may expect to observe that certain select histone acetylation markers are more prone to modification over others within the cell. In this vein, the PROTACs reported here aid design principles for HDAC1 selective degraders and provide starting points towards the development of potential new HDAC1 targeting PROTAC therapeutics.

J. T. H. was supported by the EPSRC (EP/S030492/1), thanks EPSRC for grant (EP/W02151X/1) and BBSRC IAA funding. S. M. C. was supported by the MRC (MR/J009202/1) and BBSRC (BB/N002954/1), (BB/P021689/1). A. R. P. was funded by the Fundação de Amparo à Pesquisa do Estado de São Paulo FAPESP (2018/19523-7) (2021/10059-9) (2023/05739-6).

## Conflicts of interest

There are no conflicts to declare.

## Supplementary Material

CC-060-D4CC05138F-s001

## Data Availability

The data supporting this article have been included as part of the ESI.†

## References

[cit1] Békés M., Langley D. R., Crews C. M. (2022). Nat. Rev. Drug Discovery.

[cit2] Roy M. J., Winkler S., Hughes S. J., Whitworth C., Galant M., Farnaby W., Rumpel K., Ciulli A. (2019). ACS Chem. Biol..

[cit3] Lai A. C., Crews C. M. (2017). Nat. Rev. Drug Discovery.

[cit4] Fang Y., Wang S., Han S., Zhao Y., Yu C., Liu H., Li N. (2023). Trends Pharmacol. Sci..

[cit5] Patel U., Smalley J. P., Hodgkinson J. T. (2023). RSC Chem. Biol..

[cit6] Xiao Y., Wang J., Zhao L. Y., Chen X., Zheng G., Zhang X., Liao D. (2020). Chem. Commun..

[cit7] Cao F., de Weerd S., Chen D., Zwinderman M. R. H., Van Der Wouden P. E., Dekker F. J. (2020). Eur. J. Med. Chem..

[cit8] Xiong Y., Donovan K. A., Eleuteri N. A., Kirmani N., Yue H., Razov A., Krupnick N. M., Nowak R. P., Fischer E. S. (2021). Cell Chem. Biol..

[cit9] Zhao C., Chen S., Chen D., Río-Bergé C., Zhang J., Van Der Wouden P. E., Daemen T., Dekker F. J. (2023). Angew. Chem., Int. Ed..

[cit10] Smalley J. P., Adams G. E., Millard C. J., Song Y., Norris J. K. S., Schwabe J. W. R., Cowley S. M., Hodgkinson J. T. (2020). Chem. Commun..

[cit11] Smalley J. P., Baker I. M., Pytel W. A., Lin L. Y., Bowman K. J., Schwabe J. W. R., Cowley S. M., Hodgkinson J. T. (2022). J. Med. Chem..

[cit12] Cross J. M., Coulson M. E., Smalley J. P., Pytel W. A., Ismail O., Trory J. S., Cowley S. M., Hodgkinson J. T. (2022). RSC Med. Chem..

[cit13] Baker I. M., Smalley J. P., Sabat K. A., Hodgkinson J. T., Cowley S. M. (2023). Biochemistry.

[cit14] Millard C. J., Watson P. J., Fairall L., Schwabe J. W. R. (2017). Trends Pharmacol. Sci..

[cit15] Fuller N. O., Pirone A., Lynch B. A., Hewitt M. C., Quinton M. S., McKee T. D., Ivarsson M. (2019). ACS Chem. Neurosci..

[cit16] Ibrahim H. S., Abdelsalam M., Zeyn Y., Zessin M., Mustafa A.-H. M., Fischer M. A., Zeyen P., Sun P., Bülbül E. F., Vecchio A., Erdmann F., Schmidt M., Robaa D., Barinka C., Romier C., Schutkowski M., Krämer O. H., Sippl W. (2021). Int. J. Mol. Sci..

[cit17] Schäker-Hübner L., Haschemi R., Büch T., Kraft F. B., Brumme B., Schöler A., Jenke R., Meiler J., Aigner A., Bendas G., Hansen F. K. (2022). ChemMedChem.

[cit18] Xiao Y., Hale S., Awasthee N., Meng C., Zhang X., Liu Y., Ding H., Huo Z., Lv D., Zhang W., He M., Zheng G., Liao D. (2023). Cell Chem. Biol..

