# Supplementary material for: Cereblon-recruiting proteolysis targeting chimeras (PROTACs) can determine the selective degradation of HDAC1 over HDAC3
Source: Chem Commun (Camb). Author manuscript; Available in PMC 2025 Dec 15. (PMC11563196; doi:10.1039/d4cc05138f)
Supplement: Supplementary Information [file EMS211468-supplement-Supplementary_Information.pdf]

Supplementary Information (SI) for ChemComm.
This journal is © The Royal Society of Chemistry 2024

Cereblon-recruiting proteolysis targeting chimeras can determine the selective degradation of HDAC1 over HDAC3

Aline R. Pavan,[a,b†] Joshua P. Smalley,[a†] Urvashi Patel,[a] Wiktoria A. Pytel,[a] Jean Lenardo dos Santos,[b] Shaun M. Cowley,[c*] John W.R. Schwabe,[d*] and James T. Hodgkinson[a*]

Supplementary Information

Summary

Supplementary Information: Biology 3

1. Cell Culture, Compound Handling and Western Blotting Protocols 3
2. Western Blotting Results 4

2.1. Western Blotting Analysis of Compounds **3-15** from Initial Screen 4

2.2. Representative Blots from Dose Response Experiment with **5**,**7**, and **12** 8

Supplementary Information: Chemistry 10

3. General Methods 10

3.1. Synthesis of HDAC inhibitor **(1)** 11

3.2. Synthesis of HDAC inhibitor **(2)** 16

3.3. Synthesis of Pomalidomide-linker conjugates (7 atoms length) 19

3.4. Synthesis of Pomalidomide-linker conjugates (12 atoms length) 23

3.5. Synthesis of the PROTACs **3-6** and **10-13** 29

3.6. Synthesis of the PROTACs **7-9** and **14-15** 42

3.7. Synthesis of **5-Me** and **7-Me** 49

Appendix: ^{1}H NMR and ^{13}C NMR of the Compounds 57

Appendix: Analytical HPLC 113

Appendix: Full Western Blots 116

Supplementary Information: Biology

1. Cell Culture, Compound Handling and Western Blotting Protocols

The HCT116 cell line used in this study was obtained from ATCC (CCL-247). HCT116 human colon carcinoma cells were grown in a Dulbecco's modified Eagle medium (GIBCO, 41965-039) supplemented with 10% FBS (Sigma) and 1X glutamine/penicillin/streptomycin (GIBCO, 10378−016). Cells were incubated at 37 °C with 5% CO_2. Cells were treated with compounds at indicated concentrations (0.01−10 μM). All compounds were made up to a standard solution of 10 mM in DMSO, serially diluted in DMSO, then added to fresh medium (5 mL total) to afford the concentrations stated (0.01−10 μM), with a final DMSO volume of 0.1%.

HCT116 cells were seeded into 6-well plates (4×10^5 cells/well) for 24 hours and then treated with DMSO or compounds at the indicated concentrations in fresh medium (5 mL total). After 24 hours treatment time, the cells were harvested and then lysed in lysis buffer (50 mM Tris-HCl, 150 mM NaCl, 0.5% NP-40, 0.5% Triton X-100) supplemented with a protease inhibitor (Sigma, P8340). The suspension was incubated on ice for 30 min, then centrifuged (18,000 rcf, 15 min, 4 °C) and the supernatant collected. Protein concentrations were quantified via Bradford Assay using Protein Assay Dye Reagent Concentrate (BIO-RAD). For histone extraction, an equal volume of 0.4 N H_2SO_4 was added to the pellets, and the extracts were placed at 4 °C overnight, centrifuged (18,000 rcf, 15 min, 4 °C), then the supernatant (histone extract) collected.

Western blots were run on NuPAGE 4−12% bis−Tris gels with 30 μg of protein or 10 μL of acid-extracted histone loaded per lane, using NuPAGE LDS sample buffer (4X). PageRuler Plus Prestained Ladder was used for size standards. After gel electrophoresis at 140 V for 90 min, the separated proteins were transferred onto a nitrocellulose membrane at 30 V for 60 min. The membranes were probed with primary antibodies (as indicated below) for 60−90 min. Blots were developed with complimentary IRDye®-conjugated secondary antibodies, and the bands were visualized using an Odyssey infrared imaging system. Image processing and band intensity quantification were performed using Image Studio Lite.

<u>Antibody Information</u>

Primary Antibodies;

α-tubulin - Sigma, t5168 (1:10,000 dilution)

HDAC1 - Abcam, 109411 (1:2,000 dilution)

HDAC2 - Merck Millipore, 05-814 (1:2,000 dilution)

HDAC3 - Abcam, 32369 (1:2,000 dilution)

H3 - Merck Millipore, 05-499 (1:1,000 dilution)

H3K56Ac - Active Motif, 39082 (1:1,000 dilution)

Secondary Antibodies;

IRDye® 680LT - LI-COR Biosciences, 926-68023 (1:10,000 dilution)

IRDye® 800CW - LI-COR Biosciences, 926-32210 (1:10,000 dilution)

2. Western Blotting Results

2.1. Western Blotting Analysis of Compounds **3-15** from Initial Screen

Figure S1. Western blotting of HDAC1/2/3 proteins in HCT116 cells treated with compounds 3-15.

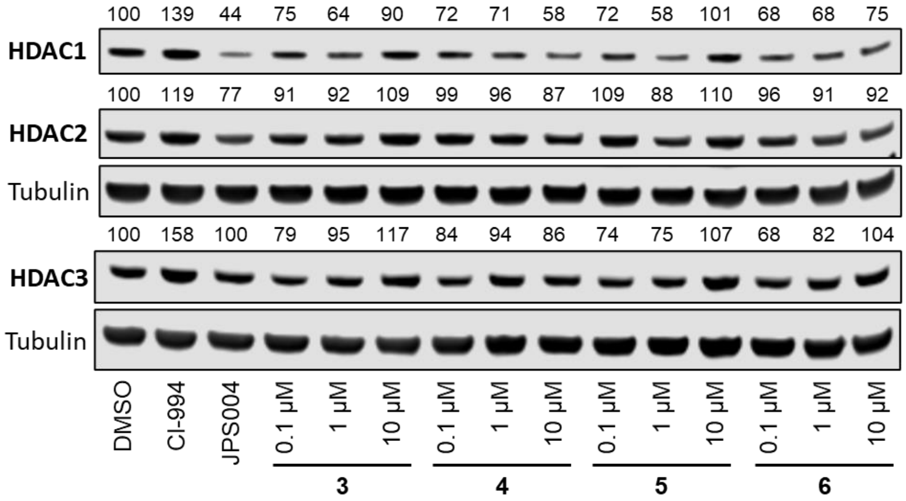

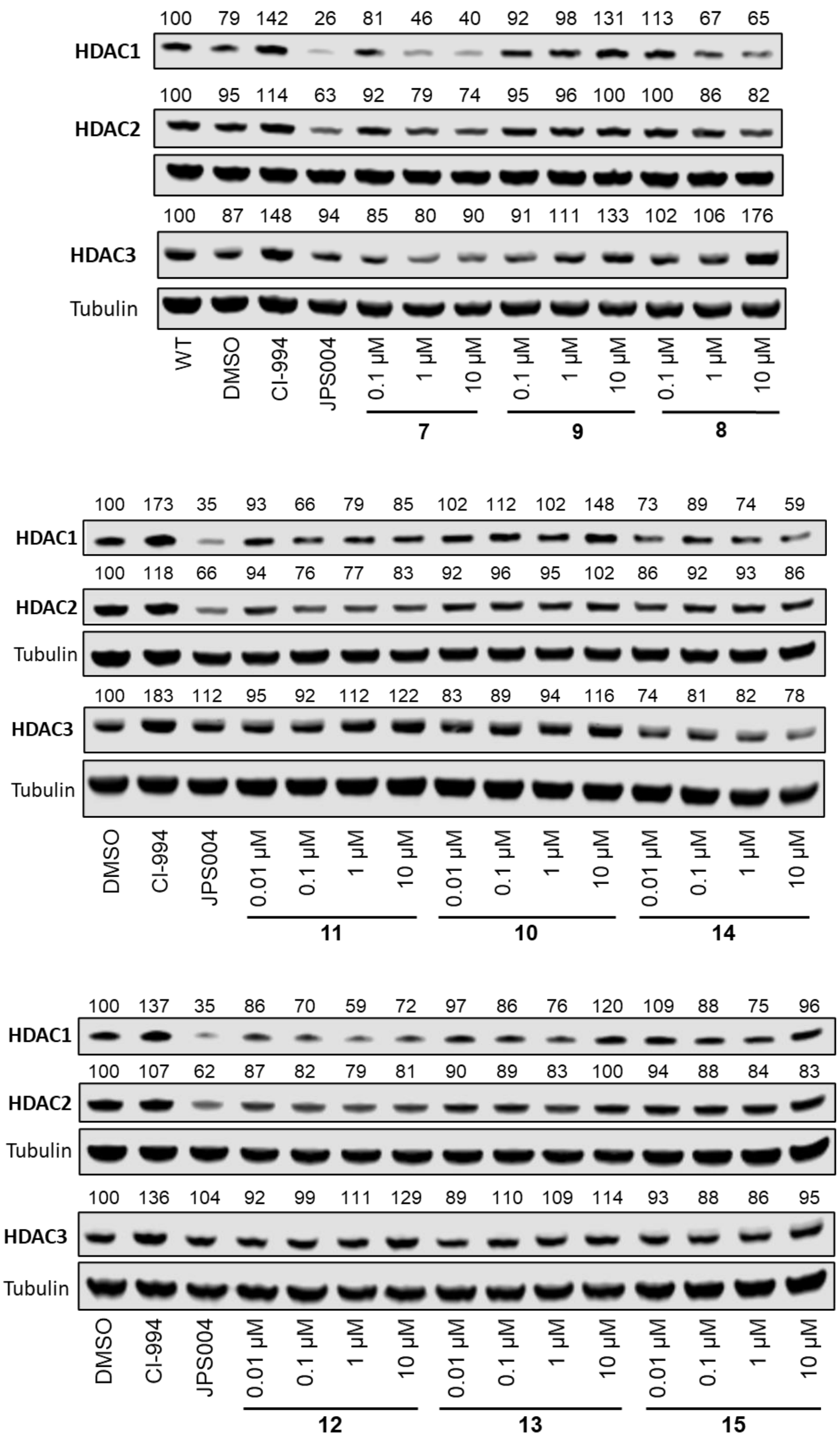
100 79 142 26 81 46 40 92 98 131 113 67 65
HDAC1
100 95 114 63 92 79 74 95 96 100 100 86 82
HDAC2
100 87 148 94 85 80 90 91 111 133 102 106 176
HDAC3
Tubulin
WT DMSO CI-994 JPS004 0.1 µM 1 µM 10 µM 0.1 µM 1 µM 10 µM 0.1 µM 1 µM 10 µM
7 9 8
100 173 35 93 66 79 85 102 112 102 148 73 89 74 59
HDAC1
100 118 66 94 76 77 83 92 96 95 102 86 92 93 86
HDAC2
Tubulin
100 183 112 95 92 112 122 83 89 94 116 74 81 82 78
HDAC3
Tubulin
DMSO CI-994 JPS004 0.01 µM 0.1 µM 1 µM 10 µM 0.01 µM 0.1 µM 1 µM 10 µM 0.01 µM 0.1 µM 1 µM 10 µM
11 10 14
100 137 35 86 70 59 72 97 86 76 120 109 88 75 96
HDAC1
100 107 62 87 82 79 81 90 89 83 100 94 88 84 83
HDAC2
Tubulin
100 136 104 92 99 111 129 89 110 109 114 93 88 86 95
HDAC3
Tubulin
DMSO CI-994 JPS004 0.01 µM 0.1 µM 1 µM 10 µM 0.01 µM 0.1 µM 1 µM 10 µM 0.01 µM 0.1 µM 1 µM 10 µM
12 13 15

Figure S2. Western blotting of H3/H3K56ac in HCT116 cells treated with compounds 3-15, complete with representative graphs showing fold-change relative to DMSO.

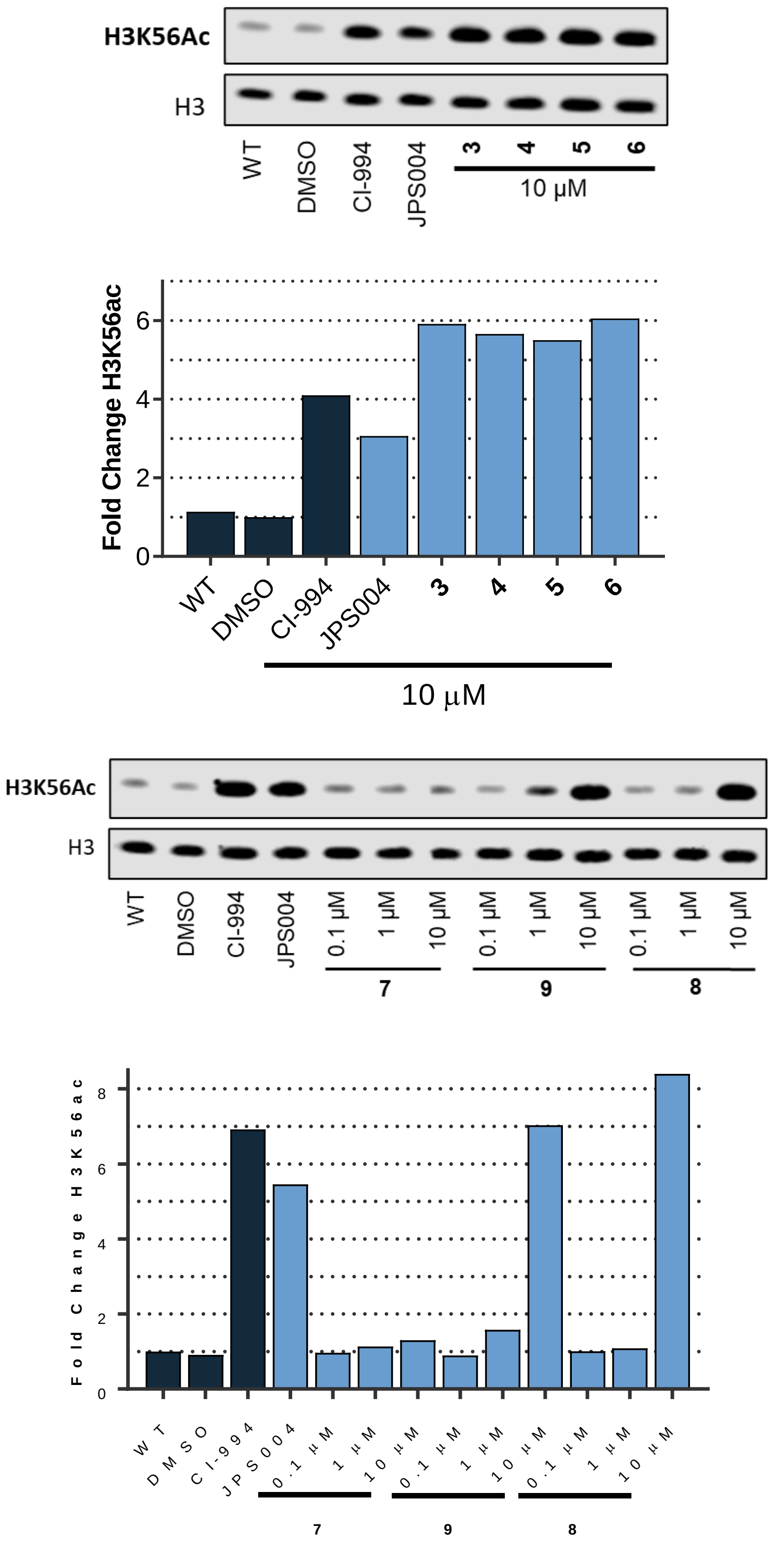

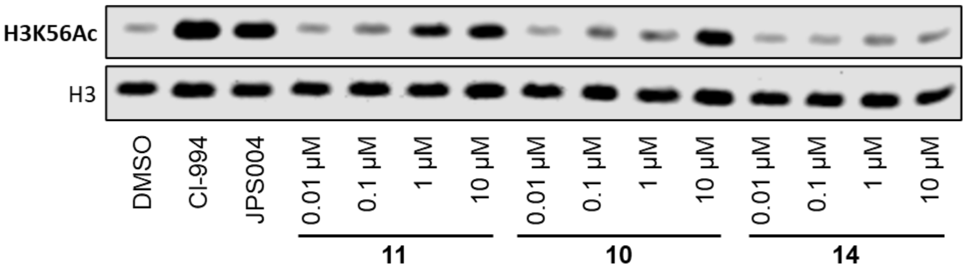
H3K56Ac
H3
DMSO
CI-994
JPS004
0.01 µM
0.1 µM
1 µM
10 µM
0.01 µM
0.1 µM
1 µM
10 µM
0.01 µM
0.1 µM
1 µM
10 µM
11
10
14

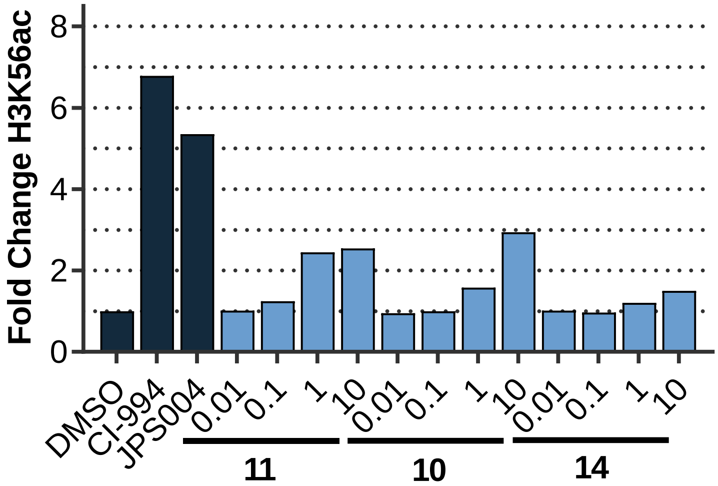
Fold Change H3K56ac
0
2
4
6
8
DMSO
CI-994
JPS004
0.01
0.1
1
10
0.01
0.1
1
10
0.01
0.1
1
10
11
10
14

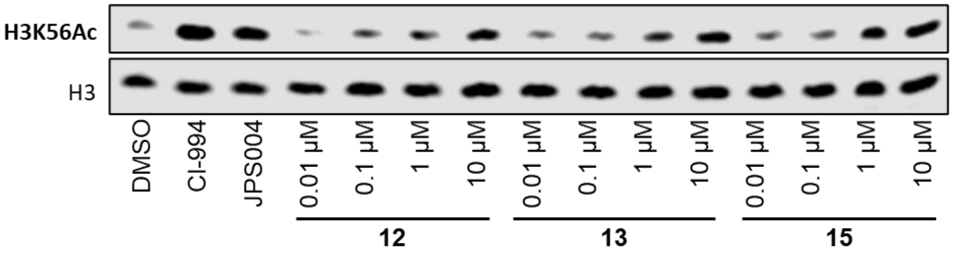
H3K56Ac
H3
DMSO
CI-994
JPS004
0.01 µM
0.1 µM
1 µM
10 µM
0.01 µM
0.1 µM
1 µM
10 µM
0.01 µM
0.1 µM
1 µM
10 µM
12
13
15

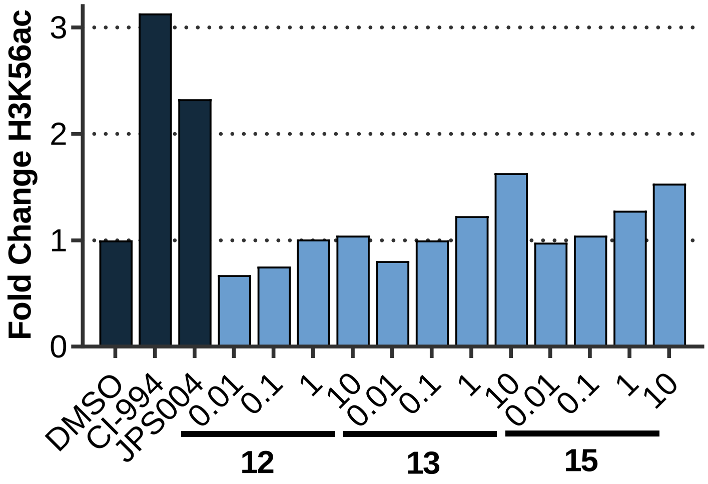
Fold Change H3K56ac
0
1
2
3
DMSO
CI-994
JPS004
0.01
0.1
1
10
0.01
0.1
1
10
0.01
0.1
1
10
12
13
15

2.2. Representative Blots from Dose Response Experiment with **5**,**7**, and **12**

Figure S3. Western blotting of HDAC1/2/3 proteins in HCT116 cells treated with compounds 5,7 and 12 at concentration range from 0.01-10 µM. Blots of two independent biological replicates.

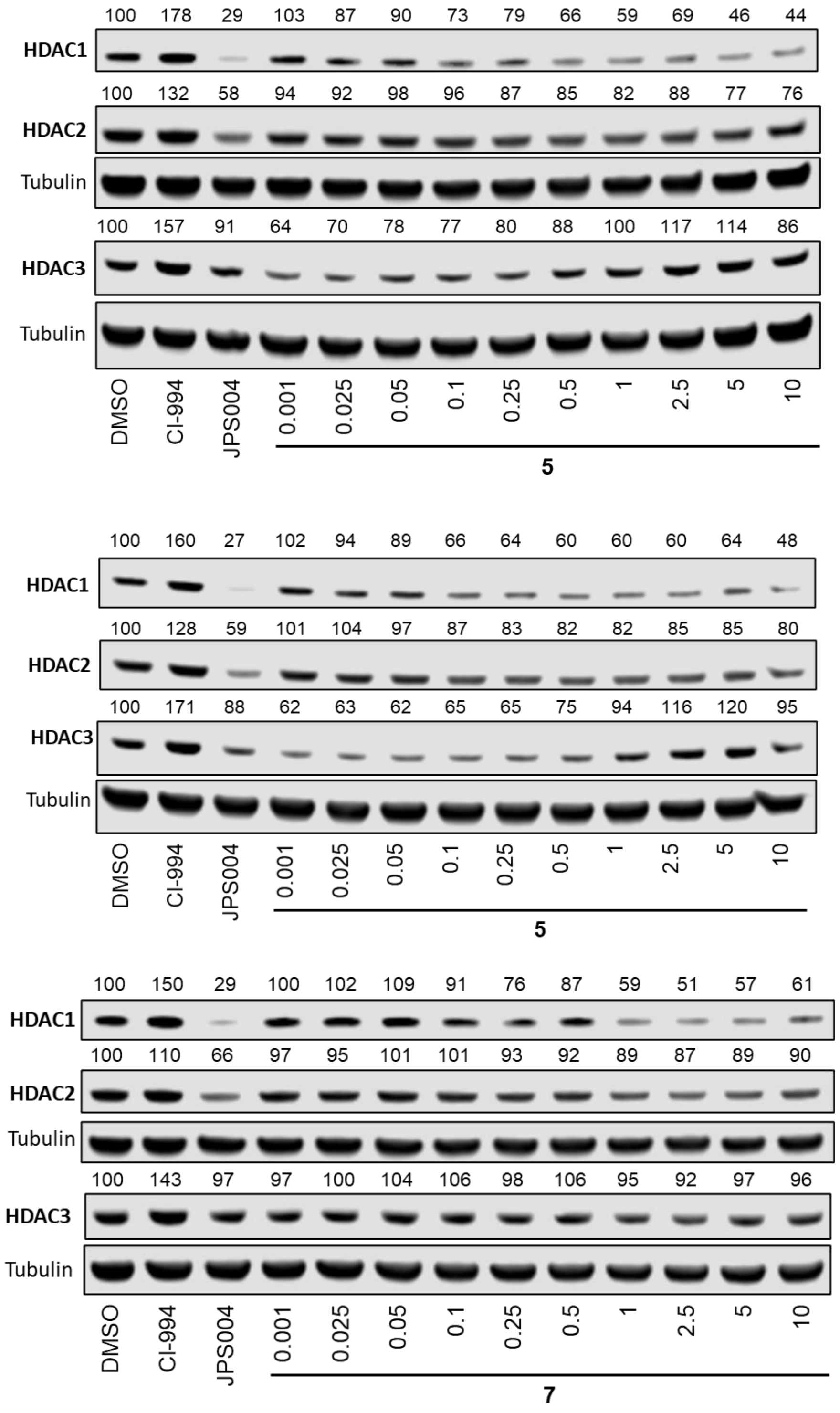

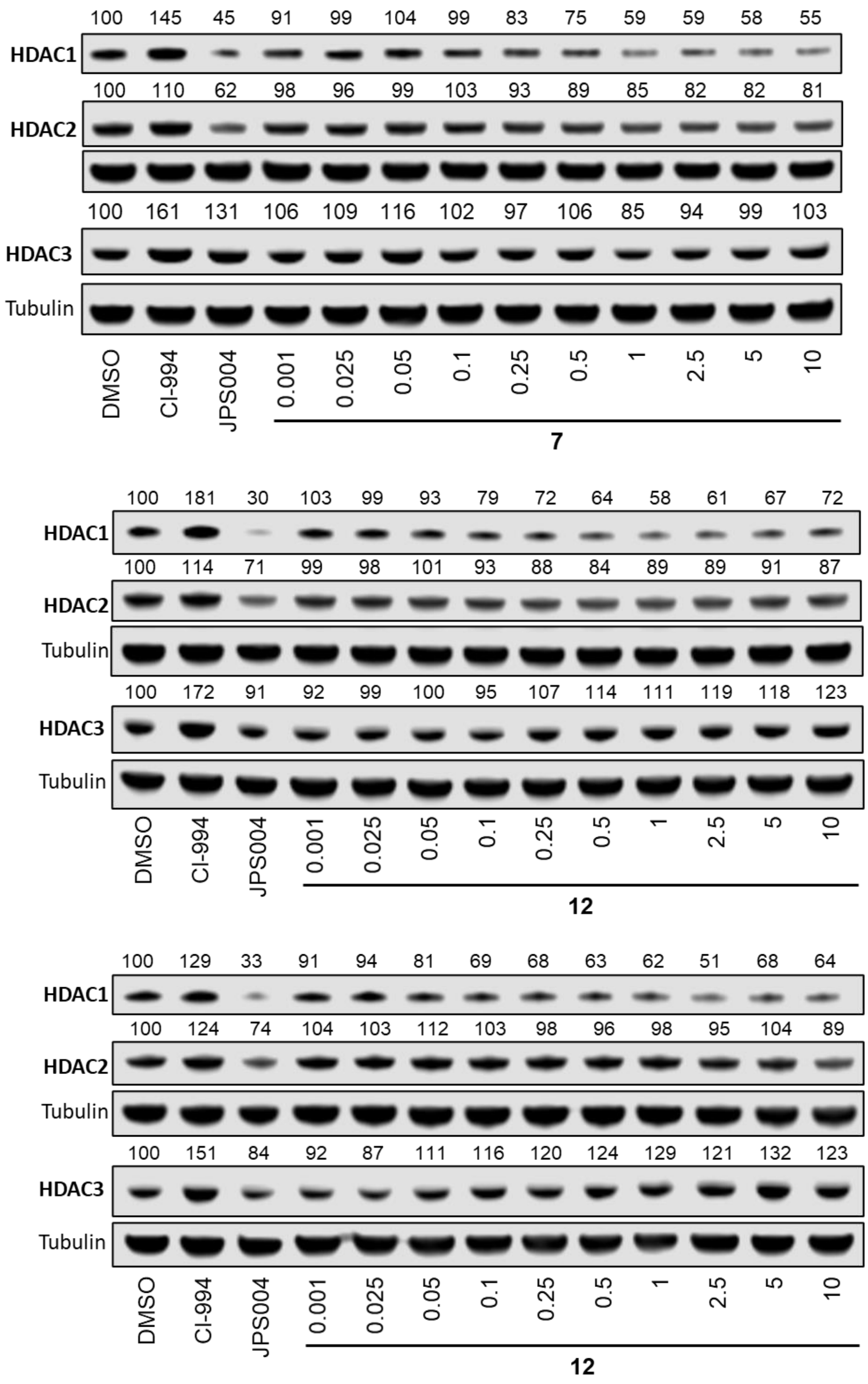
100 145 45 91 99 104 99 83 75 59 59 58 55
HDAC1
100 110 62 98 96 99 103 93 89 85 82 82 81
HDAC2
100 161 131 106 109 116 102 97 106 85 94 99 103
HDAC3
Tubulin
DMSO CI-994 JPS004 0.001 0.025 0.05 0.1 0.25 0.5 1 2.5 5 10
7
100 181 30 103 99 93 79 72 64 58 61 67 72
HDAC1
100 114 71 99 98 101 93 88 84 89 89 91 87
HDAC2
Tubulin
100 172 91 92 99 100 95 107 114 111 119 118 123
HDAC3
Tubulin
DMSO CI-994 JPS004 0.001 0.025 0.05 0.1 0.25 0.5 1 2.5 5 10
12
100 129 33 91 94 81 69 68 63 62 51 68 64
HDAC1
100 124 74 104 103 112 103 98 96 98 95 104 89
HDAC2
Tubulin
100 151 84 92 87 111 116 120 124 129 121 132 123
HDAC3
Tubulin
DMSO CI-994 JPS004 0.001 0.025 0.05 0.1 0.25 0.5 1 2.5 5 10
12

Supplementary Information: Chemistry

3. General Methods

All reagents and solvents were obtained from Sigma Aldrich, Acros Organics, Fluorochem, Fisher Scientific and were used as supplied unless stated otherwise. Biotage® Macroporous polystyrene-co-divinylbenzene (MP) carbonate resin (3.02 mmol/g loading capacity) was used for neutralising amine TFA salts and scavenging excess TFA during tert-butoxycarbonyl deprotection reactions. Room temperature refers to ambient temperature. Temperatures of 0 °C were maintained using an ice-water bath. The reactions were monitored by thin-layer chromatography (TLC) on aluminum backed silica gel. Unless otherwise stated Flash column chromatography was carried out with Silica Gel 60 using commercial solvents. All evaporations *in vacuo* were performed under reduced pressure using a Büchi rotary evaporator. All chemical names have been generated using ChemDraw Professional.

Nuclear magnetic resonance (NMR) spectra were acquired using a Bruker 400 (^{1}H, 400 MHz; ^{13}C 101 MHz) instrument at ambient temperature using deuterated solvent as reference - $CDCl_3$ (δ_H = 7.26 ppm, δ_C = 77.00 ppm), DMSO-d_6 (δ_H = 2.50 ppm, δ_C = 39.51 ppm) or CD_3OD (δ_H = 3.31 ppm, δ_C = 49.15 ppm). ^{1}H NMR data are reported as: chemical shift, multiplicity [b, broad; s, singlet; d, doublet; t, triplet; q, quartet; quin, quintet m for multiplet; or as a combination (e.g., dd, dt, etc.)], coupling constant(s) and integration. ^{13}C NMR spectra were recorded by broadband proton decoupling. Where ^{1}H and ^{13}C NMR's have been fully assigned, 2D NMR including ^{1}H-^{1}H COSY (correlated spectroscopy), ^{1}H-^{13}C HSQC (heteronuclear single quantum coherence) and ^{1}H-^{13}C HMBC (heteronuclear multiple bond coherence) were used to aid assignment.

3.1. Synthesis of HDAC inhibitor **(1)**

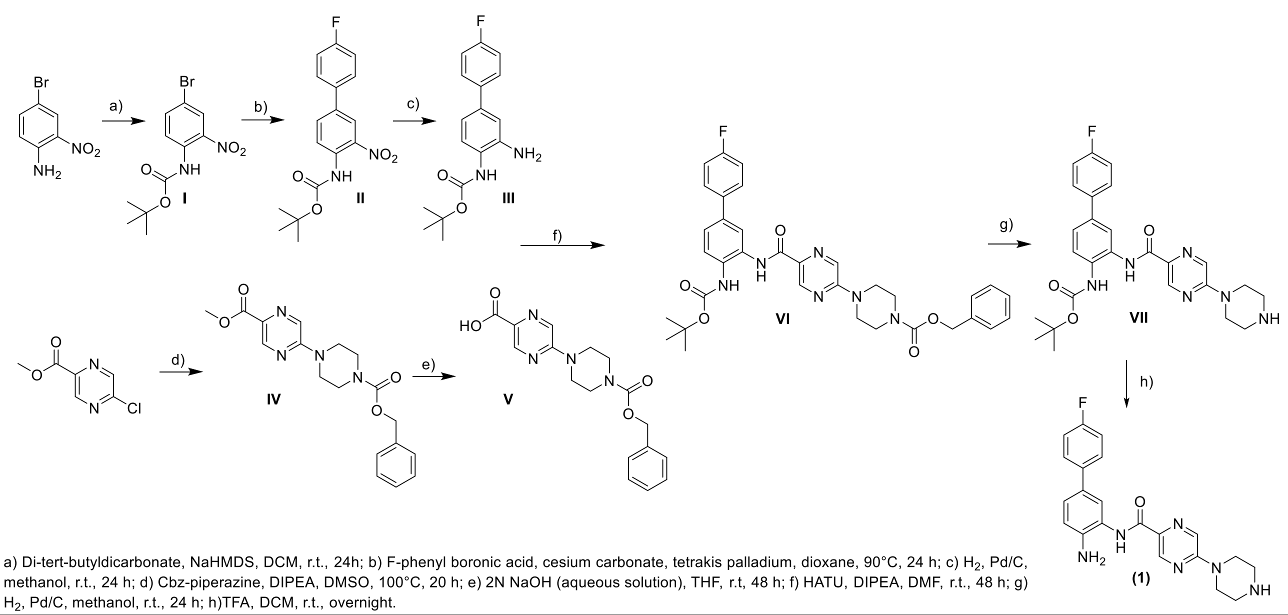

a) Di-tert-butyldicarbonate, NaHMDS, DCM, r.t., 24h; b) F-phenyl boronic acid, cesium carbonate, tetrakis palladium, dioxane, 90°C, 24 h; c) H_2, Pd/C, methanol, r.t., 24 h; d) Cbz-piperazine, DIPEA, DMSO, 100°C, 20 h; e) 2N NaOH (aqueous solution), THF, r.t, 48 h; f) HATU, DIPEA, DMF, r.t., 48 h; g) H_2, Pd/C, methanol, r.t., 24 h; h)TFA, DCM, r.t., overnight.

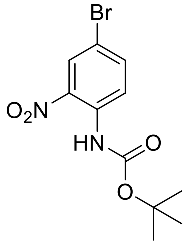

***tert*-butyl (4-bromo-2-nitrophenyl)carbamate, I[1]:** A solution of di-tert-butyldicarbonate (2.7 mmol, 1.2 eq) in anhydrous DCM (10 mL) was added dropwise to a solution of 4-bromo-2-nitroaniline (2.3 mmol, 1.0 eq) and sodium bis(trhimethylsilyl)amide (NaHMDS) (5 mL) in anhydrous DCM (15 mL) at 0 °C, then the mixture was stirred for 24 h. The reaction mixture was then concentrated *in vacuo* to yield a black/dark red sticky solid/oil, which was re-dissolved in ethyl acetate (100 mL) and washed with distilled H_2O (2 x 50 mL) and sat. NaCl (1 x 50 mL). The organic layer was collected, dried over $MgSO_4$, filtered and concentrated *in vacuo*. The crude product was purified by column chromatography (25% EtOAc in hexanes) to afford compound **I** as a yellow/orange solid in a yield of 33%. ^{1}H NMR (400 MHz, $CDCl_3$) δ 9.60 (s, 1H), 8.51 (d, J = 9.2 Hz, 1H), 8.33 (d, J = 2.4 Hz, 1H), 7.68 (dd, J=9.2, 1.4Hz, 1H), 1.54 (s, 9H). ^{13}C NMR (101 MHz, $CDCl_3$) δ 152.10 (s), 138.68 (s), 136.32 (s), 135.28 (s), 128.41 (s), 122.35 (s), 113.88 (s), 82.46 (s), 28.32 (s). Spectroscopic data is consistent with previous literature.[1]

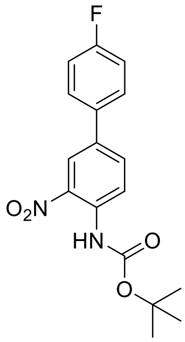

***tert*-butyl (4'-fluoro-3-nitro-[1,1'-biphenyl]-4-yl)carbamate, II:** A solution of 4-fluorophenyl boronic acid (2,05 mmol, 1.3 eq), cesium carbonate (4.1 mmol, 2.6 eq - aqueous solution 50:50 m/v) and Tetrakis(triphenylphosphine)-palladium(0) (0.079 mmol, 5 mol%) were added to a solution of **I** in anhydrous1,4-dioxane (20 mL), then the reaction was stirred at 90 °C for 24 h. The reaction mixture was cooled to room temperature and concentrated *in vacuo* to afford a yellow crude mixture. The crude mixture was resuspended in ethyl acetate (50 mL), filtered in Celite (washed with 50-100mL of ethyl acetate, or until the Celite was clear), then washed with distilled water (4 x 50 mL). The crude product was purified by column chromatography (10% EtOAc in hexane) to afford compound **II** as a yellow solid with 54% yield. ^{1}H NMR (400 MHz, DMSO-d_6) δ 9.65 (s, 1H), 8.17 (d, J = 2.2 Hz, 1H), 7.97 (dd, J = 8.6, 2.3 Hz, 1H), 7.78 (dd, J = 8.9, 5.4 Hz, 2H), 7.73 (d, J = 8.6 Hz, 1H), 7.32 (t, J = 8.9 Hz, 2H), 1.46 (s, 9H). ^{13}C NMR (101 MHz, DMSO-d_6) δ 162.22 (d, J = 245.4 Hz), 152.40 (s), 141.47 (s), 134.74 (s), 133.99 (d, J = 3.0 Hz), 131.94 (s), 131.64 (s), 128.76 (d, J = 8.3 Hz), 124.44 (s), 122.77 (s), 115.96 (d, J = 21.5 Hz), 80.60 (s), 27.90 (s). ^{19}F NMR (376 MHz, DMSO-d_6) δ -114.41 (s). Spectroscopic data is consistent with previous literature.[2]

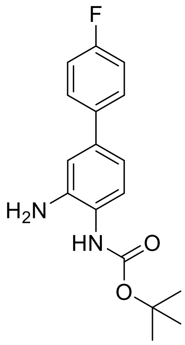

***tert*-butyl (3-amino-4'-fluoro-[1,1'-biphenyl]-4-yl)carbamate, III:** To a solution of compound **II** (0.64 mmol, 1.0 eq) in methanol (15 mL), 10% Pd/C was added. The flask was evacuated using a Schlenk line (3 times) and filled with nitrogen. A balloon filled with H_2 was added to the reaction, that was then stirred at room temperature for 24 h. The reaction mixture was filtered through celite, and the solvent was removed *in vacuo* to afford compound **III** as a white solid in a yield of 93%. ^{1}H NMR (400 MHz, DMSO-d_6) δ 8.35 (s, 1H), 7.56 (dd, J = 8.9, 5.5 Hz, 2H), 7.30 (d, J = 8.2 Hz, 1H), 7.24 (t, J = 8.9 Hz, 2H), 6.95 (d, J = 2.1 Hz, 1H), 6.80 (dd, J = 8.2, 2.2 Hz,

1H), 4.96 (s, 2H), 1.47 (s, 9H). ^{13}C NMR (101 MHz, DMSO-*d$_6$*) δ 161.50 (d, *J* = 243.4 Hz), 153.56 (s), 141.22 (s), 136.97 (s), 135.63 (s), 128.05 (d, *J* = 8.0 Hz), 124.52 (s), 123.42 (s), 115.52 (d, *J* = 21.2 Hz), 114.71 (s), 113.65 (s), 78.80 (s), 28.17 (s). ^{19}F NMR (376 MHz, DMSO-*d$_6$*) δ -116.36 (s). HRMS (ESI) m/z: $[M+H]^+$ calculated for $C_{17}H_{20}N_2O_2F$: 303.1509, found 303.1511.

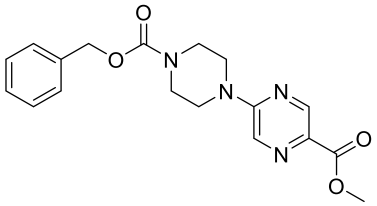

methyl 5-(4-((benzyloxy)carbonyl)piperazin-1-yl)pyrazine-2-carboxylate, IV: A solution of methyl 5-chloro-2-pyrazinecarboxylate (0.87 mmol, 1.5 eq) in DMSO (5 mL) was added to a solution of Cbz-piperazine (0.57 mmol, 1.0 eq) and DIPEA (0.87 mmol, 1.5 eq) in DMSO (10 mL) and stirred at 100°C for 20 h. Upon cooling, 100 mL of DCM was added to the reaction and washed with distilled water (2 x 50mL) and brine (1 x 50mL). The organic layer was dried over $MgSO_4$ and concentrated *in vacuo*. The crude product was purified by column chromatography (80-100% EtOAc in hexane) to afford compound **IV** as a brown solid in a yield of 78.3%. ^{1}H NMR (400 MHz, $CDCl_3$) δ 8.80 (d, *J* = 1.3 Hz, 1H), 8.13 (d, *J* = 1.4 Hz, 1H), 7.38 – 7.30 (m, 5H), 5.17 (s, 2H), 3.95 (s, 3H), 3.79 – 3.74 (m, 4H), 3.66 (dd, *J* = 6.5, 3.9 Hz, 4H). ^{13}C NMR (101 MHz, $CDCl_3$) δ 165.17 (s), 155.28 (s), 155.06 (s), 145.35 (s), 136.45 (s), 131.51 (s), 129.46 (s), 128.71 (s), 128.38 (s), 128.18 (s), 67.67 (s), 52.49 (s), 44.04 (s), 43.34 (s). HRMS (ESI) m/z: $[M+H]^+$ calculated for $C_{18}H_{21}N_4O_4$: 357.1563, found 357.1559, $[M+Na]^+$ calculated for $C_{18}H_{20}N_4O_4Na$: 379.1382, found 379.1365.

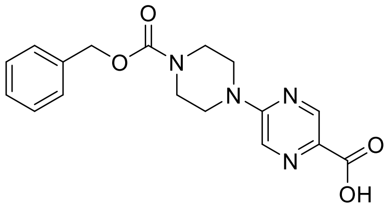

5-(4-((benzyloxy)carbonyl)piperazin-1-yl)pyrazine-2-carboxylic acid, V: A solution of sodium hydroxide (2.40 mL, 2 M, 3.3 eq) was added to a solution of compound **IV** (2.8 mmol, 1.0 eq) in THF (30 mL). The reaction was stirred for 48 h at room temperature. The solvent was concentrated *in vacuo*, dissolved in ethyl acetate (100-150 mL) and washed with saturated solution of potassium bissulfate (1 x 50mL or until pH <2) and distilled water (2 x 50 mL). The organic layer was dried over $MgSO_4$, filtered and the solvent was concentrated *in vacuo* to afford compound **V** as a yellow solid with yield of 82%. ^{1}H NMR (400 MHz, DMSO-*d$_6$*) δ 12.72 (s, 1H), 8.65 (d, *J* = 1.2 Hz, 1H), 8.35 (d, *J* = 1.2 Hz, 1H), 7.41 – 7.31 (m, 5H), 5.12 (s, 2H), 3.80 – 3.73 (m, 4H), 3.54 (s, 4H). ^{13}C NMR (101 MHz, DMSO-*d$_6$*) δ 165.45 (s), 154.53 (s), 154.48 (s), 144.34 (s), 136.77 (s), 130.93 (s), 130.29 (s), 128.44 (s), 127.90 (s), 127.64 (s), 66.41 (s), 43.38 (s), 42.84 (s). HRMS (ESI) m/z: $[M+H]^+$ calculated for $C_{17}H_{19}N_4O_4$: 343.1406, found 343.1405.

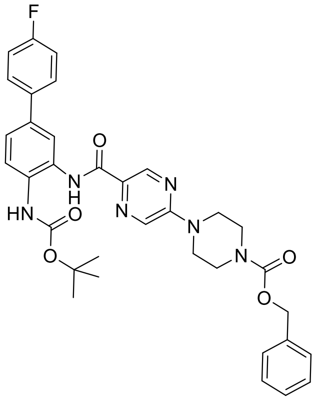

benzyl 4-(5-((4-((*tert*-butoxycarbonyl)amino)-4'-fluoro-[1,1'-biphenyl]-3-yl)carbamoyl)pyrazin-2-yl)piperazine-1-carboxylate, VI: A solution of DIPEA (1.39 mmol, 2.8 eq) and HATU (0.69 mmol, 1.4 eq) were added to a solution of compound **V** (0.59 mmol, 1.2 eq) in anhydrous DMF (10 mL) and stirred at room temperature for 15 minutes. A solution of compound **III** (0.49 mmol, 1.0 eq) in anhydrous DMF (3 mL) was added dropwise and the reaction was stirred at room temperature for 48 h. Then 100 mL of ethyl acetate was added to the reaction and washed with saturated solution of sodium bicarbonate (2 x 50mL) and distilled water (1 x 50mL). The organic layer was dried over $MgSO_4$, filtered and the solvent was concentrated *in vacuo*. The crude product was purified by column chromatography (100% EtOAc) to afford compound **VI** as a white solid in a yield of 67%. ^1H NMR (400 MHz, DMSO-d_6) δ 10.08 (s, 1H), 9.12 (s, 1H), 8.76 (d, J = 1.3 Hz, 1H), 8.27 (d, J = 1.0 Hz, 1H), 8.23 (d, J = 1.8 Hz, 1H), 7.68 (dd, J = 8.9, 5.4 Hz, 2H), 7.44 – 7.27 (m, 9H), 5.13 (s, 2H), 3.81 – 3.77 (m, 4H), 3.57 (s, 4H), 1.50 (s, 9H). ^{13}C NMR (101 MHz, DMSO-d_6) δ 161.87 (s), 161.85 (d, J = 244.8 Hz), 155.10 (s), 154.50 (s), 153.88 (s), 142.24 (s), 136.78 (s), 136.18 (s), 136.04 (d, J = 2.9 Hz), 132.25 (s), 132.11 (s), 129.06 (s), 128.83 (s), 128.52 (s), 128.45 (s), 127.92 (s), 127.64 (s), 125.85 (s), 122.80 (s), 121.91 (s), 115.79 (d, J = 21.4 Hz), 79.83 (s), 66.42 (s), 43.54 (s), 42.90 (s), 28.09 (s). ^{19}F NMR (376 MHz, DMSO-d_6) δ -115.50 (s). HRMS (ESI) m/z: $[M+H]^+$ calculated for $C_{34}H_{36}N_6O_5F$: 627.2731, found 627.2733; $[M+Na]^+$ calculated for $C_{34}H_{35}N_6O_5FNa$: 649.2551, found 649.2553.

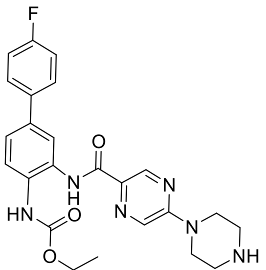

***tert*-butyl (4'-fluoro-3-(5-(piperazin-1-yl)pyrazine-2-carboxamido-[1,1'-biphenyl]-4-yl)carbamate, VII**: To a solution of compound **VI** (0.64 mmol, 1.0 eq) in methanol (15 mL), 10% Pd/C was added. The flask was evacuated using a Schlenk line (3 times) and filled with nitrogen. A balloon filled with H_2 was added to the reaction, that was then stirred at room temperature for 48 h. The reaction mixture was filtered through celite, and

the solvent was removed *in vacuo* to afford compound **VII** as a white solid in a yield of 89%. ^{1}H NMR (400 MHz, DMSO-d_6) δ 10.06 (s, 1H), 9.11 (s, 1H), 8.74 (d, *J* = 1.2 Hz, 1H), 8.24 (s, 2H), 7.68 (dd, *J* = 8.8, 5.4 Hz, 3H), 7.42 (dd, *J* = 8.3, 2.1 Hz, 1H), 7.35 (d, *J* = 8.3 Hz, 1H), 7.30 (t, *J* = 8.9 Hz, 3H), 3.71 – 3.67 (m, 4H), 2.88 – 2.80 (m, 5H), 1.50 (s, 9H). ^{13}C NMR (101 MHz, DMSO-d_6) δ 161.96 (s), 161.85 (d, *J* = 244.5 Hz), 155.27 (s), 153.88 (s), 142.33 (s), 136.20 (s), 136.06 (d, *J* = 3.0 Hz), 132.20 (s), 131.76 (s), 129.02 (s), 128.64 (s), 128.48 (d, *J* = 8.2 Hz), 125.88 (s), 122.75 (s), 121.86 (s), 115.79 (d, *J* = 21.4 Hz), 79.83 (s), 45.01 (s), 44.76 (s), 28.08 (s). ^{19}F NMR (376 MHz, DMSO-d_6) δ -115.51 (s). HRMS (ESI) m/z: $[M+H]^+$ calculated for $C_{26}H_{30}N_6O_3F$: 493.2363, found 493.2368; $[M+Na]^+$ calculated for $C_{26}H_{29}N_6O_3FNa$: 515.2183, found 515.2188.

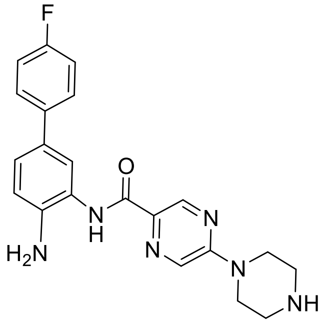

N-(4-amino-4'-fluoro-[1,1'-biphenyl]-3-yl)-5-(piperazin-1-yl)pyrazine-2-carboxamide, (1): TFA (0.5 mL) was added to a solution of compound **VII** (0.09 mmol, 1.0 eq) in DCM (2 mL) at 0°C, then the reaction was stirred at room temperature for 4 h. The solvent was concentrated *in vacuo* and the mixture re-suspended in methanol (10 mL). MP-carbonate resin was added to this solution and stirred at room temperature for 4 hours. The reaction was filtered, and the solvent was removed *in vacuo*. Purification was performed by column chromatography (alumina, 5% methanol in DCM) to afford compound (**1**) as a crystalline white solid in a yield of 68%. ^{1}H NMR (400 MHz, CD_3OD) δ ppm 8.80 (d, *J*=1.4 Hz, 1 H), 8.27 (d, *J*=1.4 Hz, 1 H), 7.67 (d, *J*=2.2 Hz, 1 H), 7.55 - 7.59 (m, 2 H), 7.31 (dd, J=8.3, 2.2 Hz, 1 H), 7.07 - 7.17 (m, 3 H), 6.97 (d, *J*=8.3 Hz, 1 H), 3.76 - 3.79 (m, 4 H), 2.93 - 2.96 (m, 4 H). ^{13}C NMR (101 MHz, CD_3OD) δ 165.1 (s), 163.5 (d, *J*=243.8 Hz), 157.1 (s), 143.8 (s), 142.5 (s), 138.6 (d, *J*=3.0 Hz), 133.9 (s), 132.1 (s), 130.1 (s), 129.1 (d, *J*=7.8 Hz, C15), 126.4 (s), 125.7 (s), 124.9 (s), 119.1 (s), 116.5 (d, J=21.6 Hz), 46.3 (s), 46.1 (s). ^{19}F NMR (376 MHz, CD_3OD) δ -119.4 (s). HRMS (ESI) m/z: [M+H]+ calculated for $C_{21}H_{22}FN_6O$: 393.1839, found 393.1836.

3.2. Synthesis of HDAC inhibitor **(2)**

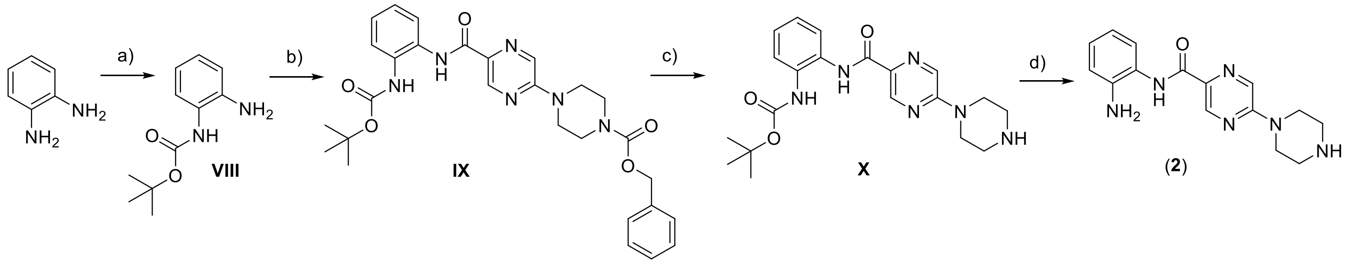

a) Di-tert-butyldicarbonate, NaHMDS, DCM, r.t., 24h; b) **V**, HATU, DIPEA, DMF, r.t., 48h; c) H_2, Pd/C, Methanol r.t., 24h; d)TFA, DCM, r.t., overnight.

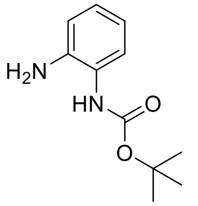

***tert*-butyl (2-aminophenyl)carbamate, VIII:** A solution of di-tert-butyl dicarbonate (2.8 mmol, 1.0 eq) in anhydrous THF (10 mL) was added dropwise to a solution of *o*-phenylenediamine (2.8 mmol, 1.0 eq) and triethylamine (4.7 mL) in anhydrous THF (15 mL) at 0°C, then the mixture was stirred for 24 hours. The reaction mixture was then concentrated *in vacuo* to yield a grey/white solid, which was re-dissolved in ethyl acetate (100 mL) and washed with distilled H_2O (2 x 50 mL) and sat. NaCl (1 x 50 mL). The organic layer was collected, dried over $MgSO_4$, filtered, and concentrated *in vacuo*. The crude was purified by column chromatography (10-90% EtOAc in hexane) to afford compound **VIII** as a slight pink solid in a yield of 69%. ^{1}H NMR (400 MHz, $CDCl_3$) δ 7.28 (d, 1H), 7.00 (td, J = 7.6, 1.5 Hz, 1H), 6.82 – 6.74 (m, 2H), 1.51 (s, 9H). ^{13}C NMR (101 MHz, $CDCl_3$) δ 153.96 (s), 140.06 (s), 126.27 (s), 124.93 (s), 124.79 (s), 119.77 (s), 117.76 (s), 80.66 (s), 28.47 (s). HRMS (ESI) m/z: $[M+Na]^+$ calculated for $C_{11}H_{16}N_2O_2Na$: 231.1109, found 231.1111.

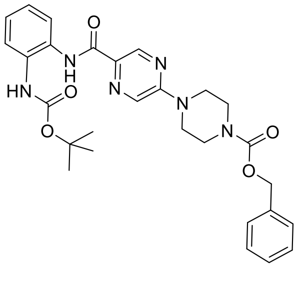

benzyl 4-(5-((2-((*tert*-butoxycarbonyl)amino)phenyl)carbamoyl)pyrazin-2-yl)piperazine-1-carboxylate, IX: A solution of DIPEA (2.1 mmol, 1.5 eq) and HATU (1.8 mmol, 1.3 eq) were added to a solution of compound **V** (1.7 mmol, 1.2 eq) in anhydrous DMF (10 mL) and stirred at room temperature for 15 minutes. Later, a solution

of compound **VIII** (1.4 mmol, 1.0 eq) in anhydrous DMF (3 mL) was added dropwise and the reaction was stirred at room temperature for 48 hours. Then 100 mL of ethyl acetate was added to the reaction and washed with saturated solution of sodium bicarbonate (2 x 50 mL) and distilled water (1 x 50 mL). The product precipitated in the organic layer and was then filtered to afford compound **IX** as a yellow solid in a yield of 75%. ^{1}H NMR (400 MHz, DMSO-d_6) δ 10.00 (s, 1H), 9.03 (s, 1H), 8.75 (d, J = 1.3 Hz, 1H), 8.25 (d, J = 0.6 Hz, 1H), 7.96 (d, J = 7.4 Hz, 1H), 7.41 – 7.31 (m, 5H), 7.28 – 7.19 (m, 2H), 7.13 (td, J = 7.6, 1.5 Hz, 1H), 5.13 (s, 2H), 3.82 – 3.75 (m, 4H), 3.56 (s, 4H), 1.48 (s, 8H). ^{13}C NMR (101 MHz, DMSO-d_6) δ 161.65 (s), 155.08 (s), 154.50 (s), 153.92 (s), 142.15 (s), 136.78 (s), 132.32 (s), 132.05 (s), 129.56 (s), 128.79 (s), 128.45 (s), 127.91 (s), 127.63 (s), 125.64 (s), 125.45 (s), 124.54 (s), 123.54 (s), 79.67 (s), 66.41 (s), 43.53 (s), 42.83 (s), 28.08 (s). HRMS (ESI) m/z: $[M+H]^+$ calculated for $C_{28}H_{33}N_6O_5$: 533.2512, found 533.2512; $[M+Na]^+$ calculated for $C_{28}H_{32}N_6O_5Na$: 555.2332, found 555.2325.

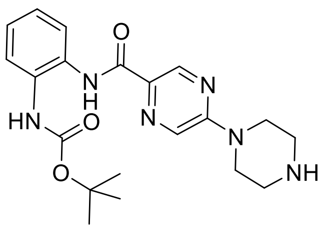

***tert*-butyl (2-(5-(piperazin-1-yl)pyrazine-2-carboxamido)phenyl)carbamate, X:** To a solution of compound **IX** (0.14 mmol, 1.0 eq) in methanol (15 mL), 10% Pd/C was added. The flask was evacuated using a Schlenk line (3 times) and filled with nitrogen. A balloon filled with H_2 was added to the reaction, that was then stirred at room temperature for 48 h. The reaction mixture was filtered through celite, and the solvent was removed *in vacuo* to afford compound **X** as a white solid in a yield of 85%. ^{1}H NMR (400 MHz, DMSO-d_6) δ 9.97 (s, 1H), 9.02 (s, 1H), 8.71 (d, J = 1.3 Hz, 1H), 8.21 (d, J = 0.9 Hz, 1H), 7.96 (d, J = 7.9 Hz, 1H), 7.27 – 7.19 (m, 2H), 7.12 (td, J = 7.6, 1.5 Hz, 1H), 3.65 (t, 4H), 2.79 (t, 4H), 1.48 (s, 9H). ^{13}C NMR (101 MHz, DMSO-d_6) δ 161.79 (s), 155.33 (s), 153.95 (s), 142.31 (s), 132.15 (s), 131.69 (s), 129.54 (s), 128.59 (s), 125.71 (s), 125.51 (s), 124.53 (s), 123.57 (s), 79.71 (s), 45.35 (s), 45.19 (s), 28.11 (s). HRMS (ESI) m/z: $[M+H]^+$ calculated for $C_{20}H_{26}N_6O_3$: 399.2145, found 399.2143; $[M+Na]^+$ calculated for $C_{20}H_{25}N_6O_3Na$: 421.1964, found 421.1960.

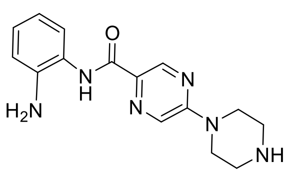

***N*-(2-aminophenyl)-5-(piperazin-1-yl)pyrazine-2-carboxamide**, (**2**): TFA was added to a solution of compound **X** (0.12 mmol, 1.0 eq) in DCM (7 mL) at 0 °C, then the reaction was stirred at room temperature for 24 h. The solvent was concentrated *in vacuo* and re-suspended in methanol (20 mL). MP-carbonate resin was added to this solution and stirred at room temperature for 4 hours. The reaction was filtered, and the solvent was removed *in*

vacuo. Purification was performed by column chromatography (alumina, 5% methanol in DCM) to afford compound (**2**) as a crystalline white solid in a yield of 85%. ^{1}H NMR (400 MHz, CD_3OD) δ 8.77 (d, *J* = 1.3 Hz, 1H), 8.24 (d, *J* = 1.4 Hz, 1H), 7.39 (dd, *J* = 7.9, 1.4 Hz, 1H), 7.05 (ddd, *J* = 7.9, 7.4, 1.5 Hz, 1H), 6.91 (dd, *J* = 8.0, 1.4 Hz, 1H), 6.79 (td, *J* = 7.8, 1.4 Hz, 1H), 3.75 (t, 4H), 2.94 (t, 4H). ^{13}C NMR (101 MHz, CD_3OD) δ 164.91 (s), 156.93 (s), 143.54 (s), 142.86 (s), 133.72 (s), 130.00 (s), 127.94 (s), 126.48 (s), 125.36 (s), 119.84 (s), 118.74 (s), 46.15 (s), 45.95 (s). HRMS (ESI) m/z: $[M+H]^+$ calculated for $C_{15}H_{19}N_6O$: 299.1621, found 299.1620; $[M+Na]^+$ calculated for $C_{15}H_{18}N_6ONa$: 321.1442, found 321.1440.

3.3. Synthesis of Pomalidomide-linker conjugates (7 atoms length)

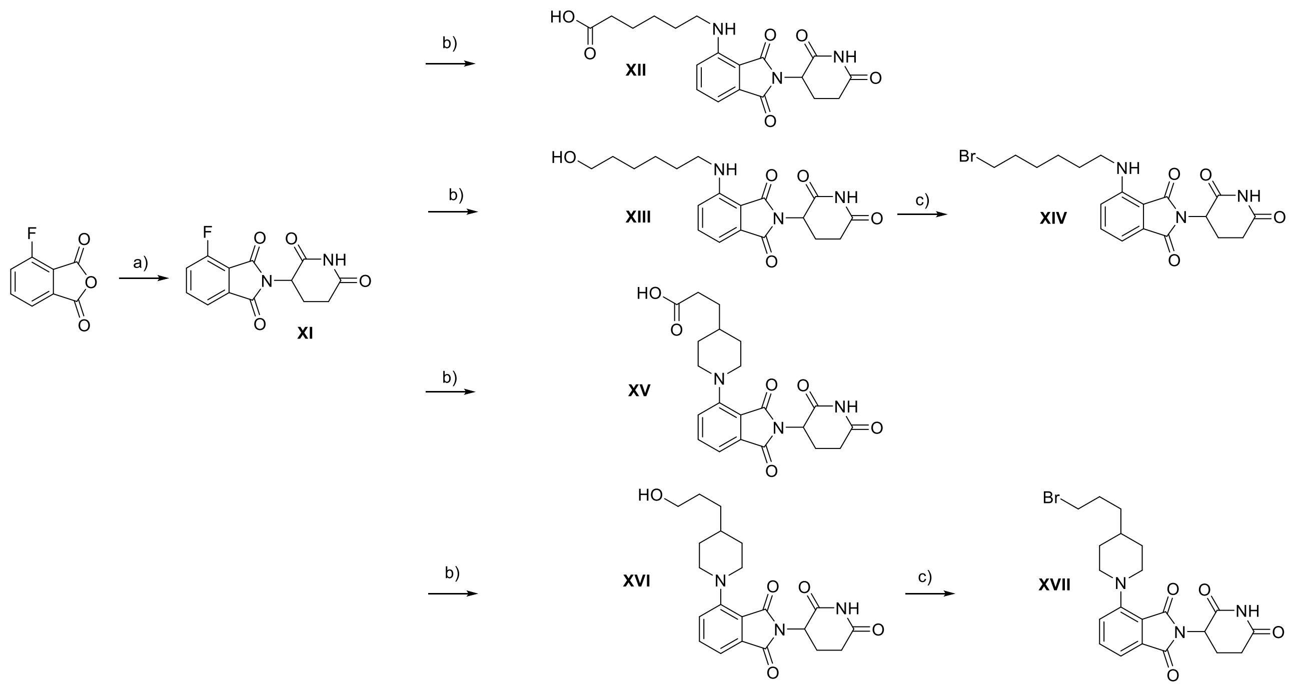

a) 3-aminopiperidine-2,6-dione hydrochloride, sodium acetate, acetic acid, 140°C, 24h; b) 6-aminocapric acid or 6-aminohexan-1-ol or 3-(iperidin-4-yl)propanoic acid or 3-(piperidin-4-yl)propan-1-ol, DIPEA, DMSO, 90°C, 24h; c) Thionyl bromide, DCM, r.t., overnight.

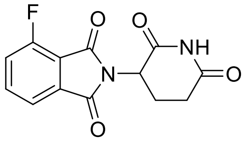

2-(2,6-dioxopiperidin-3-yl)-4-fluoroisoindoline-1,3-dione, XI: A solution of 3-aminopiperidine-2,6-dione hydrochloride (1.8 mmol, 1.0 eq), and sodium acetate (2.1 mmol, 1.2 eq) in acetic acid (10 mL) was added to a solution of 3-flurophtalic anhydride (1.8 mmol, 1.0 eq) in acetic acid (5 mL), then the resultant mixture was stirred at 140 °C for 24 h. The reaction mixture was cooled to room temperature and then the volume of acetic acid was reduced *in vacuo* to afford a brown crude mixture. The crude product was purified by column chromatography (2.5-4% of methanol in DCM), to afford compound **XI** as a white solid in a yield of 77.7%. ^{1}H NMR (400 MHz, DMSO-d_6) δ 11.14 (s, 1H), 7.95 (ddd, J = 8.4, 7.4, 4.6 Hz, 1H), 7.79 (d, J = 7.4 Hz, 1H), 7.73 (dd, J = 8.4 Hz, 1H), 5.16 (dd, J = 12.9, 5.4 Hz, 1H), 2.89 (ddd, J = 17.1, 13.9, 5.5 Hz, 1H), 2.65 – 2.56 (m, 1H), 2.55 – 2.45 (m, 1H), 2.10 – 2.02 (m, 1H). ^{13}C NMR (101 MHz, DMSO-d_6) δ 172.73 (s), 169.68 (s), 166.10 (d, J = 2.8 Hz), 163.97 (s), 156.81 (d, J = 262.4 Hz), 138.05 (d, J = 7.9 Hz), 133.45 (d, J = 1.3 Hz), 123.00 (d, J = 19.5 Hz), 120.04 (d, J = 3.2 Hz), 117.03 (d, J = 12.6 Hz), 49.09 (s), 30.90 (s), 21.84 (s). ^{19}F NMR (376 MHz, DMSO-d_6) δ -114.67 (s). HRMS (ESI) m/z: $[M+H]^+$ calculated for $C_{13}H_{10}N_2O_4F$: 277.0625, found 277.0626.

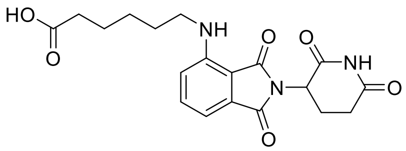

6-((2-(2,6-dioxopiperidin-3-yl)-1,3-dioxoisoindolin-4-yl)amino)hexanoic acid, XII: To a solution of **XI** (0.7 mmol, 1.0 eq) in anhydrous DMSO (10mL), DIPEA (1.4 mmol, 2.0 eq) and 6-aminocapric acid (0.86 mmol, 1.2 eq) were added and the resultant mixture was stirred at 90 °C for 24 h. The reaction mixture was cooled to room temperature then poured in water (30 mL) and washed with EtOAc (2 x 50 mL). The organic layers were combined, washed with sat. brine (2 x 50 mL), dried over $MgSO_4$, filtered, and concentrated *in vacuo* to afford a yellow/orange tar. The crude product was purified by column chromatography (50-70% EtOAc in hexane) to afford compound **XII** as yellow solid in a yield of 20 %. ^{1}H NMR (400 MHz, DMSO-d_6) δ 11.09 (s, 1H), 7.57 (dd, J = 8.5, 7.2 Hz, 1H), 7.08 (d, J = 8.6 Hz, 1H), 7.01 (d, J = 6.9 Hz, 1H), 6.53 (t, J = 5.9 Hz, 1H), 5.04 (dd, J = 12.8, 5.4 Hz, 1H), 3.28 (dd, J = 13.3, 6.7 Hz, 2H), 2.88 (ddd, J = 17.2, 14.0, 5.4 Hz, 1H), 2.63 – 2.56 (m, 1H), 2.55 – 2.51 (m, 1H), 2.21 (t, J = 7.3 Hz, 2H), 2.06 – 1.99 (m, 1H), 1.55 (tt, J = 14.8, 7.4 Hz, 4H), 1.40 – 1.30 (m, 2H). ^{13}C NMR (101 MHz, DMSO-d_6) δ 174.52 (s), 172.85 (s), 170.14 (s), 168.96 (s), 167.34 (s), 146.44 (s), 136.31 (s), 132.22 (s), 117.21 (s), 110.41 (s), 109.03 (s), 48.56 (s), 41.76 (s), 33.68 (s), 31.01 (s), 28.48 (s), 25.93 (s), 24.29 (s), 22.18 (s). HRMS (ESI) m/z: $[M+H]^+$ calculated for $C_{19}H_{22}N_3O_6$: 388.1509, found 388.1515, $[M+Na]^+$ calculated for $C_{19}H_{21}N_3O_6Na$: 410.1328, found 410.1336.

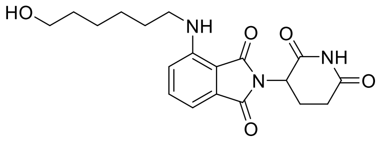

2-(2,6-dioxopiperidin-3-yl)-4-((6-hydroxyhexyl)amino)isoindoline-1,3-dione, XIII: To a solution of **XI** (0.7 mmol, 1.0 eq) in anhydrous DMSO (10 mL), DIPEA (1.4 mmol, 2.0 eq) and 6-aminohexan-1-ol (0.86 mmol, 1.2 eq) were added and the resultant mixture was stirred at 90 °C for 24h. The reaction mixture was cooled to room temperature then poured in water (30 mL) and washed with EtOAc (2 x 50 mL). The organic layers were combined, washed with brine (2 x 50 mL), dried over $MgSO_4$, filtered and concentrated *in vacuo* to afford a yellow/orange tar. The crude product was purified by column chromatography (70% EtOAc in hexane) to afford compound **XIII** as yellow solid in a yield of 60%. ^{1}H NMR (400 MHz, $CDCl_3$) δ 8.16 (s, 1H), 7.48 (dd, J = 8.1, 7.2 Hz, 1H), 7.08 (dd, J = 7.1, 0.5 Hz, 1H), 6.88 (d, J = 8.5 Hz, 1H), 6.23 (t, J = 5.5 Hz, 1H), 4.91 (dd, J = 12.1, 5.4 Hz, 1H), 3.65 (t, J = 4.9 Hz, 2H), 3.27 (dd, J = 12.7, 7.0 Hz, 2H), 2.92 – 2.67 (m, 3H), 2.18 – 2.09 (m, 1H), 1.72 – 1.64 (m, 2H), 1.58 (dd, J = 13.4, 6.8 Hz, 2H), 1.48 – 1.41 (m, 4H). ^{13}C NMR (101 MHz, $CDCl_3$) δ 171.13 (s), 169.67 (s), 168.48 (s), 167.76 (s), 147.14 (s), 136.27 (s), 132.63 (s), 116.79 (s), 111.56 (s), 110.00 (s), 62.91 (s), 49.00 (s), 42.70 (s), 32.68 (s), 31.55 (s), 29.30 (s), 26.86 (s), 25.62 (s), 22.95 (s). HRMS (ESI) m/z: $[M+H]^+$ calculated for $C_{19}H_{24}N_3O_5$: 374.1716, found 374.1716, $[M+Na]^+$ calculated for $C_{19}H_{23}N_3O_5Na$: 396.1535, found 396.1541.

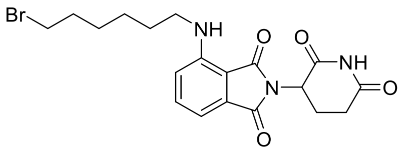

4-((6-bromohexyl)amino)-2-(2,6-dioxopiperidin-3-yl)isoindoline-1,3-dione, XIV: Thionyl bromide (1.6 mmol, 3.0 eq) was added to a solution of **XIII** (0.53 mmol, 1.0 eq) in DCM (3 mL) at 0 °C. The reaction was stirred at room temperature overnight. Then DCM (50-100 mL) was added to the reaction and washed with saturated solution of sodium bicarbonate (2 x 50 mL) and distilled water (2 x 50 mL). The organic layer was dried over $MgSO_4$ and the solvent was concentrated *in vacuo*. The crude was purified by column chromatography (50% EtOAc in hexane) to afford compound **XIV** as yellow solid in a yield of 89%. ^{1}H NMR (400 MHz, DMSO-d_6) δ 7.58 (dd, *J* = 8.5, 7.2 Hz, 1H), 7.09 (d, *J* = 8.6 Hz, 1H), 7.02 (d, *J* = 6.7 Hz, 1H), 6.54 (t, *J* = 5.9 Hz, 1H), 5.05 (dd, *J* = 12.8, 5.4 Hz, 1H), 3.53 (t, *J* = 6.7 Hz, 2H), 3.31 – 3.26 (m, 2H), 2.88 (ddd, *J* = 17.3, 14.0, 5.4 Hz, 1H), 2.62-2.52 (m, 2H), 2.07 – 1.98 (m, 1H), 1.85 – 1.77 (m, 2H), 1.63 – 1.53 (m, 2H), 1.47 – 1.33 (m, 4H). ^{13}C NMR (101 MHz, DMSO-d_6) δ 172.81 (s), 170.09 (s), 168.93 (s), 167.30 (s), 146.41 (s), 136.28 (s), 132.20 (s), 117.19 (s), 110.38 (s), 109.03 (s), 48.53 (s), 41.74 (s), 35.11 (s), 32.16 (s), 30.97 (s), 28.50 (s), 27.26 (s), 25.41 (s), 22.15 (s). HRMS (ESI) m/z: $[M+H]^+$ calculated for $C_{19}H_{23}N_3O_4{}^{79}Br$: 436.0872, found 436.0871, $[M+H]^+$ calculated for $C_{19}H_{23}N_3O_4{}^{81}Br$: 438.0851, found 438.0853.

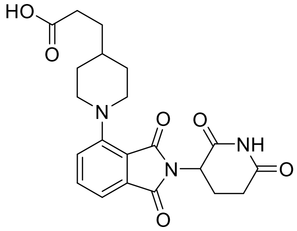

3-(1-(2-(2,6-dioxopiperidin-3-yl)-1,3-dioxoisoindolin-4-yl)piperidin-4-yl)propanoic acid, XV: To a solution of **XI** (0.7 mmol, 1.0 eq) in anhydrous DMSO (10 mL), DIPEA (1.4 mmol, 2.0 eq) and 3-(piperidin-4-yl)propanoic acid (0.86 mmol, 1.2 eq) were added. The resultant mixture was stirred at 90 °C for 24 h. The reaction mixture was cooled to room temperature then poured in water (30 mL) and washed with EtOAc (2 x 50 mL). The organic layers were combined, washed with sat. brine (2 x 50 mL), dried over $MgSO_4$, filtered and concentrated *in vacuo* to afford a yellow/orange tar. The crude product was purified by column chromatography (3% methanol in EtOAc) to afford compound **XV** as yellow solid in a yield of 81.6%. ^{1}H NMR (400 MHz, $CDCl_3$) δ 8.39 (s, 1H), 7.56 (dd, *J* = 8.4, 7.2 Hz, 1H), 7.36 (dd, *J* = 7.1, 0.5 Hz, 1H), 7.16 (d, *J* = 8.3 Hz, 1H), 4.96 (dd, *J* = 12.3, 5.3 Hz, 1H), 3.73 (t, *J* = 10.4 Hz, 2H), 2.93 – 2.70 (m, 5H), 2.43 (t, *J* = 7.6 Hz, 2H), 2.14 – 2.07 (m, 1H), 1.84 (d, *J* = 8.9 Hz, 2H), 1.68 (dd, *J* = 12.7, 7.5 Hz, 2H), 1.50 (s, 3H). ^{13}C NMR (101 MHz, $CDCl_3$) δ 178.69 (s), 171.40 (s), 168.57 (s), 167.57 (s), 166.83 (s), 150.99 (s), 135.64 (s), 134.26 (s), 123.80 (s), 117.34 (s), 115.48 (s), 52.11 (s), 51.76 (s), 49.23 (s), 34.98 (s), 32.11 (s), 31.55 (s), 31.27 (s), 31.24 (s), 31.08 (s), 22.82 (s). HRMS (ESI) m/z: $[M+H]^+$

calculated for $C_{21}H_{24}N_3O_6$: 414.1665, found 414.1666, $[M+Na]^+$ calculated for $C_{21}H_{23}N_3O_6Na$: 436.1485, found 436.1483.

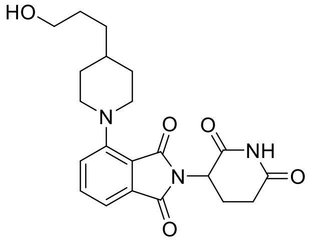

2-(2,6-dioxopiperidin-3-yl)-4-(4-(3-hydroxypropyl)piperidin-1-yl)isoindoline-1,3-dione, XVI: To a solution of **XI** (0.7 mmol, 1.0 eq) in anhydrous DMSO (10 mL), DIPEA (1.4 mmol, 2.0 eq) and 3-(piperidin-4-yl)propan-1-ol (0.86 mmol, 1.2 eq) were added and the resultant mixture was stirred at 90°C for 24h. The reaction mixture was cooled to room temperature then poured in water (30 mL) and washed with EtOAc (2 x 50 mL). The organic layers were combined, washed with sat. brine (2 x 50mL), dried over $MgSO_4$, filtered, and concentrated *in vacuo* to afford a yellow/orange tar. The crude product was purified by column chromatography (100% EtOAc) to afford compound **XVI** as yellow solid in a yield of 68.5%. ^1H NMR (400 MHz, $CDCl_3$) δ 8.13 (s, 1H), 7.55 (dd, J = 8.4, 7.2 Hz, 1H), 7.35 (dd, J = 7.1, 0.6 Hz, 1H), 7.17 (d, J = 8.0 Hz, 1H), 4.96 (dd, J = 12.2, 5.4 Hz, 1H), 3.74 (t, J = 10.2 Hz, 2H), 3.66 (t, J = 6.6 Hz, 2H), 2.92 – 2.67 (m, 5H), 2.15 – 2.06 (m, 1H), 1.84 (d, J = 9.5 Hz, 2H), 1.69 – 1.59 (m, 2H), 1.52 – 1.44 (m, 3H), 1.42 – 1.34 (m, 2H). ^{13}C NMR (101 MHz, $CDCl_3$) δ 171.12 (s), 168.36 (s), 167.59 (s), 166.82 (s), 151.08 (s), 135.58 (s), 134.27 (s), 123.79 (s), 117.21 (s), 115.33 (s), 63.29 (s), 52.28 (s), 51.94 (s), 49.23 (s), 35.47 (s), 32.64 (s), 32.50 (s), 32.48 (s), 31.55 (s), 30.04 (s), 22.82 (s). HRMS (ESI) m/z: $[M+H]^+$ calculated for $C_{21}H_{26}N_3O_5$: 400.1872, found 400.1873, $[M+Na]^+$ calculated for $C_{21}H_{25}N_3O_5Na$: 422.1692, found 422.1693.

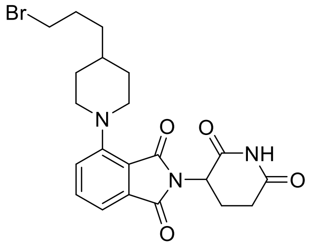

4-(4-(3-bromopropyl)piperidin-1-yl)-2-(2,6-dioxopiperidin-3-yl)isoindoline-1,3-dione, XVII: Thionyl bromide (1.6 mmol, 3.0 eq) was added to a solution of **XVI** (0.53 mmol, 1.0 eq) in DCM (3 mL) at 0°C. The reaction was stirred at room temperature overnight. Then DCM (50-100 mL) was added to the reaction and washed with saturated solution of sodium bicarbonate (2 x 50 mL) and distilled water (2 x 50 mL). The organic layer was dried over $MgSO_4$ and the solvent was concentrated *in vacuo*. The crude product was purified by column chromatography (40% EtOAc in hexane) to afford compound **XVII** as yellow solid in a yield of 64%. ^1H NMR (400 MHz, $CDCl_3$) δ 8.05 (s, 1H), 7.56 (dd, J = 8.4, 7.2 Hz, 1H), 7.36 (d, J = 7.0 Hz, 1H), 7.17 (d, J = 8.3 Hz,

1H), 4.96 (dd, J = 12.2, 5.4 Hz, 1H), 3.74 (t, J = 10.1 Hz, 2H), 3.42 (t, J = 6.8 Hz, 2H), 2.94 – 2.64 (m, 5H), 2.16 – 2.07 (m, 1H), 1.93 (dt, J = 12.1, 5.9 Hz, 2H), 1.86 – 1.80 (m, 2H), 1.55 – 1.43 (m, 5H). ^{13}C NMR (101 MHz, $CDCl_3$) δ 171.03 (s), 168.30 (s), 167.55 (s), 166.82 (s), 151.02 (s), 135.61 (s), 134.27 (s), 123.78 (s), 117.29 (s), 115.41 (s), 52.20 (s), 51.87 (s), 49.24 (s), 35.08 (s), 35.03 (s), 34.07 (s), 32.40 (s), 32.38 (s), 31.55 (s), 30.21 (s), 22.83 (s). HRMS (ESI) m/z: $[M+H]^+$ calculated for $C_{21}H_{25}N_3O_4{}^{79}Br$: 462.1028, found 462.1025, $[M+H]^+$ calculated for $C_{21}H_{25}N_3O_4{}^{81}Br$: 464.1008, found 464.1009.

3.4. Synthesis of Pomalidomide-linker conjugates (12 atoms length)

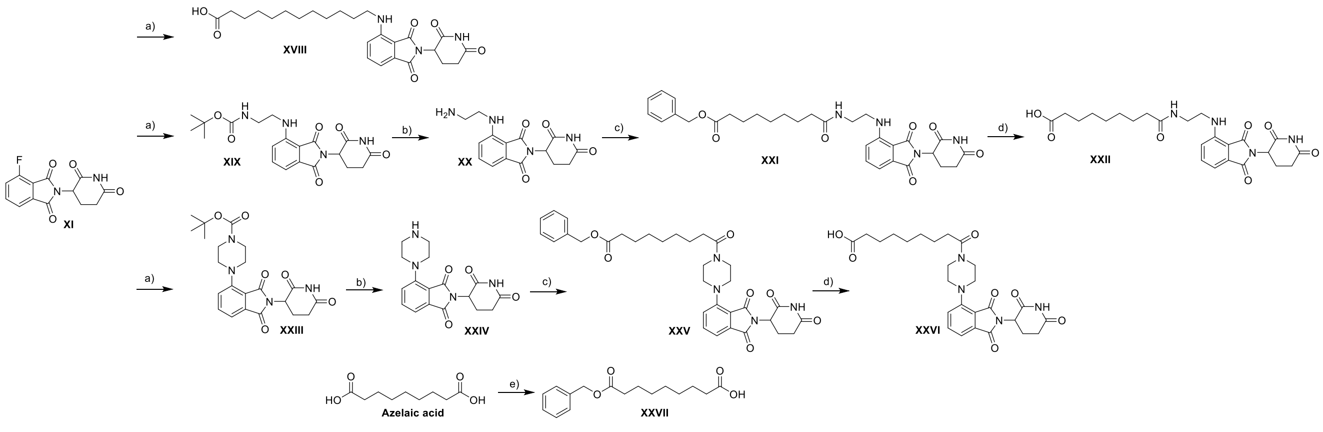

a) 12-aminododecanoic acid or tert-butyl-N-(2-aminoethylcarbamate) or boc-piperazine, DIPEA, DMSO, 90°C, 24h; b) TFA, DCM, r.t.., 24h; c) **XXVII**, HATU, DIPEA, r.t., 48h; d) Metanol, H_2, r.t., 24h; e) benzyl bromide, $NaHCO_3$, 1,4-dioxane/DMF, 90°C, 16h

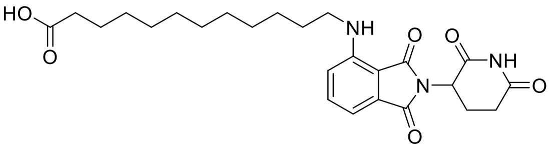

12-((2-(2,6-dioxopiperidin-3-yl)-1,3-dioxoisoindolin-4-yl)amino_dodecanoic acid, XVIII: To a solution of **XI** (0.7 mmol, 1.0 eq) in anhydrous DMSO (10 mL), DIPEA (1.4 mmol, 2.0 eq) and 12-aminododecanoic acid (0.86 mmol, 1.2 eq) were added and the resultant mixture was stirred at 90 °C for 24 h. The reaction mixture was cooled to room temperature then poured in water (30 mL) and washed with EtOAc (2 x 50 mL). The organic layers were combined, washed with sat. brine (2 x 50 mL), dried over $MgSO_4$, filtered, and concentrated *in vacuo* to afford a yellow/orange tar. The crude product was purified by column chromatography (50-100% EtOAc in hexane) to afford compound **XVIII** as yellow solid in a yield of 71%. ^{1}H NMR (400 MHz, DMSO-d_6) δ 11.94 (s, 1H), 11.08 (s, 1H), 7.57 (dd, J = 8.5, 7.2 Hz, 1H), 7.08 (d, J = 8.5 Hz, 1H), 7.01 (d, J = 7.2 Hz, 1H), 6.52 (t, J = 5.9 Hz, 1H), 5.05 (dd, J = 12.9, 5.4 Hz, 1H), 3.31 – 3.25 (m, 2H), 2.90 – 2.81 (m, 1H), 2.63 – 2.52 (m, 2H), 2.18 (t, J = 7.4 Hz, 2H), 2.06 – 1.98 (m, 1H), 1.56 (dt, J = 14.1, 7.1 Hz, 2H), 1.47 (dt, J = 12.5, 6.3 Hz, 2H), 1.33 – 1.20 (m, 16H). ^{13}C NMR (101 MHz, DMSO-d_6) δ 174.51 (s), 172.81 (s), 170.09 (s), 168.95 (s), 167.31 (s), 146.44 (s), 136.27 (s), 132.19 (s), 117.19 (s), 110.36 (s), 109.00 (s), 48.54 (s), 41.83 (s), 33.66 (s), 30.98 (s), 28.97 (s), 28.92 (s), 28.88 (s), 28.74 (s)*, 28.66 (s), 28.54 (s), 26.30 (s), 24.49 (s), 22.15 (s), * 2 x resonace signals coincide. HRMS

(ESI) m/z: $[M+H]^+$ calculated for $C_{25}H_{34}N_3O_6$: 472.2448, found 472.2442, $[M+Na]^+$ calculated for $C_{25}H_{33}N_3O_6Na$: 494.2267, found 494.2255.

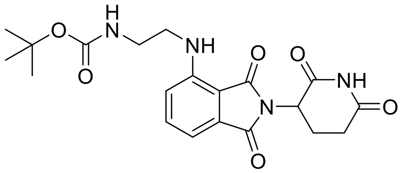

***tert*-butyl (2-((2-(2,6-dioxopiperidin-3-yl)-1,3-dioxoisoindolin-4-yl)amino_ethyl)carbamate, XIX**: To a solution of **XI** (0.7 mmol, 1.0 eq) in anhydrous DMSO (10mL), DIPEA (1.4 mmol, 2.0 eq) and *tert*-butyl-*N*-(2-aminoethylcarbamate) (0.86 mmol, 1.2 eq) were added and the resultant mixture was stirred at 90°C for 24h. The reaction mixture was cooled to room temperature then poured in water (30 mL) and washed with EtOAc (2 x 50 mL). The organic layers were combined, washed with sat. brine (2 x 50 mL), dried over $MgSO_4$, filtered, and concentrated *in vacuo* to afford a yellow/orange tar. The crude product was purified by column chromatography (50% EtOAc in hexane) to afford compound **XIX** as yellow solid in a yield of 28%. ^{1}H NMR (400 MHz, DMSO-*d₆*) δ 11.08 (s, 1H), 7.57 (dd, *J* = 8.3, 7.3 Hz, 1H), 7.14 (d, *J* = 8.6 Hz, 1H), 7.03-6.99 (m, 2H), 6.71 (t, *J* = 6.1 Hz, 1H), 5.05 (dd, *J* = 12.8, 5.4 Hz, 1H), 3.36 (q, *J* = 6.2 Hz, 2H), 3.12 (q, *J* = 6.0 Hz, 2H), 2.89 (ddd, *J* = 17.2, 13.9, 5.4 Hz, 1H), 2.63 – 2.52 (m, 2H), 2.06 – 1.97 (m, 1H), 1.36 (s, 9H). ^{13}C NMR (101 MHz, DMSO-d_6) δ 172.81 (s), 170.06 (s), 168.71 (s), 167.30 (s), 155.88 (s), 146.40 (s), 136.17 (s), 132.22 (s), 117.07 (s), 110.49 (s), 109.23 (s), 77.78 (s), 48.51 (s), 41.57 (s), 30.97 (s), 28.20 (s), 22.17 (s). HRMS (ESI) m/z: $[M+H]^+$ calculated for $C_{20}H_{25}N_4O_6$: 417.1774, found 417.1768, $[M+Na]^+$ calculated for $C_{20}H_{24}N_4O_6Na$: 439.1594, found 439.1584.

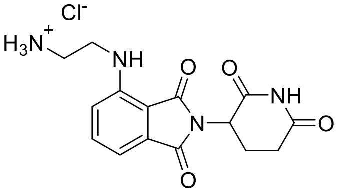

2-((2-(2,6-dioxopiperidin-3-yl)-1,3-dioxoisoindolin-4-yl)amino)ethan-1-aminium, XX: HCl in dioxane (4 N, 2 mL) was added to a stirring solution of compound **XIX** (0.18 mmol) in DCM (5 mL) and the resulting mixture was stirred at room temperature for 4 h. The reaction mixture was concentrated *in vacuo* to afford compound **XX** as a yellow solid in a yield of 95%. ^{1}H NMR (400 MHz, DMSO-d_6) δ 11.10 (s, 1H), 8.17 (s, 3H), 7.61 (dd, *J* = 8.5, 7.1 Hz, 1H), 7.26 (d, *J* = 8.5 Hz, 1H), 7.08 (d, *J* = 7.1 Hz, 1H), 6.83 (t, *J* = 6.3 Hz, 1H), 5.06 (dd, *J* = 12.8, 5.4 Hz, 1H), 3.63 (q, *J* = 6.2 Hz, 2H), 2.97 (dd, *J* = 6.2 Hz, 2H), 2.91 – 2.84 (m, 1H), 2.63 – 2.52 (m, 2H), 2.09 – 1.99 (m, 1H). ^{13}C NMR (101 MHz, DMSO-d_6) δ 172.86 (s), 170.08 (s), 168.60 (s), 167.29 (s), 145.85 (s), 136.32 (s), 132.28 (s), 117.34 (s), 111.05 (s), 110.02 (s), 66.36 (s), 48.57 (s), 37.66 (s), 31.00 (s), 22.20 (s). HRMS (ESI) m/z: $[M+H]^+$ calculated for $C_{15}H_{17}N_4O_4$: 317.1250, found 317.1248.

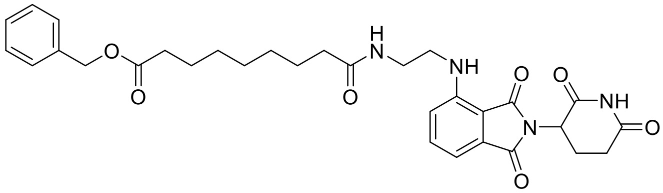

benzyl 9-((2-((2-(2,6-dioxopiperidin-3-yl)-1,3-dioxoisoindolin-4-yl)amino)ethyl)amino-9-oxononanoate, XXI: To a solution of compound **XXVII** (0.48 mmol, 1.1 eq) in anhydrous DMF (7 mL) at 0 °C, DIPEA (0.88 mmol, 2.0 eq) and HATU (0.53 mmol, 1.2 eq) were added. The reaction mixture was stirred for 15 minutes, after which a solution of compound **XX** (0.44 mmol, 1.0 eq) in DMF (3 mL) was added slowly and the resultant solution stirred at room temperature for 48 h. Then 100 mL of ethyl acetate was added to the reaction and washed with sat. solution of sodium bicarbonate (2 x 50 mL) and distilled water (2 x 50 mL). The organic layer was dried over $MgSO_4$ and the solvent was concentrated *in vacuo*. The crude product was purified by column chromatography (50-100% EtOAc in hexane) to afford compound **XXI** as yellow solid in a yield of 42%. ^{1}H NMR (400 MHz, CD_3OD) δ 7.55 (dd, *J* = 8.5, 7.1 Hz, 1H), 7.38 – 7.29 (m, 5H), 7.13 (d, *J* = 8.5 Hz, 1H), 7.06 (d, *J* = 6.9 Hz, 1H), 6.61 (t, *J* = 5.6 Hz, 1H), 5.11 (s, 2H), 5.06 (dd, *J* = 12.5, 5.4 Hz, 1H), 3.51 – 3.46 (m, 1H), 3.45 – 3.41 (m, 1H), 2.91 – 2.81 (m, 1H), 2.79 – 2.69 (m, 2H), 2.35 (t, *J* = 7.4 Hz, 2H), 2.19 – 2.15 (m, 3H), 2.14 – 2.05 (m, 1H), 1.64 – 1.54 (m, 4H), 1.31 – 1.25 (m, 6H). ^{13}C NMR (101 MHz, CD_3OD) δ 176.85 (s), 175.24 (s), 174.66 (s), 171.55 (s), 170.56 (s), 169.27 (s), 148.12 (s), 137.74 (s), 137.22 (s), 133.96 (s), 129.53 (s)*, 129.18 (s)*, 118.01 (s), 112.08 (s), 111.42 (s), 67.11 (s), 50.18 (s), 42.74 (s), 39.76 (s), 37.03 (s), 35.00 (s), 32.20 (s), 29.93, 26.83 (s), 25.95 (s), 23.79 (s), * 2 x resonace signals coincide at 129.53 and 3 x resonace signals coincide at 129.18. HRMS (ESI) m/z: $[M+H]^+$ calculated for $C_{31}H_{36}N_4O_7$: 577.2662, found 577.2661, $[M+Na]^+$ calculated for $C_{31}H_{35}N_4O_7Na$: 599.2482, found 599.2488.

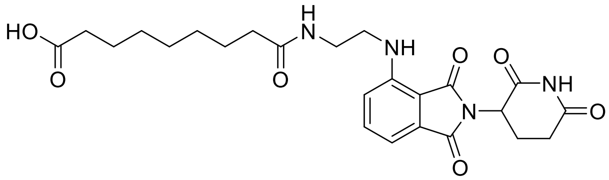

9-((2-((2-(2,6-dioxopiperidin-3-yl)-1,3-dioxoisoindolin-4-yl)amino(ethyl)amino)9-oxononanoic acid, XXII: To a solution of compound **XXI** (0.52 mmol) in methanol (15 mL), 10% Pd/C was added. The flask was evacuated using a Schlenk line (3 times) and filled with nitrogen. A balloon filled with H_2 was added to the reaction, that was then stirred at room temperature for 48 h. The reaction mixture was filtered through celite, and the solvent was removed *in vacuo* to afford compound **XXII** as a yellow solid in a yield of 93%. ^{1}H NMR (400 MHz, DMSO-*d$_6$*) δ 8.00 (t, *J* = 5.5 Hz, 1H), 7.58 (dd, *J* = 8.5, 7.2 Hz, 1H), 7.16 (d, *J* = 8.6 Hz, 1H), 7.02 (d, *J* = 6.9 Hz, 1H), 6.70 (t, *J* = 6.0 Hz, 1H), 5.04 (dd, *J* = 12.8, 5.4 Hz, 1H), 2.88 (ddd, *J* = 16.9, 13.9, 4.4 Hz, 1H), 2.63 – 2.52 (m, 2H), 2.15 (t, *J* = 7.4 Hz, 2H), 2.06 – 1.98 (m, 3H), 1.54 – 1.38 (m, 4H), 1.21 (s, 6H). ^{13}C NMR (101 MHz, DMSO-*d$_6$*) δ 174.67 (s), 172.86 (s), 171.87 (s), 170.11 (s), 168.74 (s), 167.35 (s), 146.42 (s), 136.24 (s), 132.23 (s), 117.20

(s), 110.59 (s), 109.26 (s), 48.56 (s), 41.52 (s), 35.40 (s), 33.86 (s), 31.01 (s), 28.53 (s), 28.48 (s), 25.18 (s), 24.57 (s), 22.22 (s). HRMS (ESI) m/z: $[M+H]^+$ calculated for $C_{24}H_{31}N_4O_7$: 487.2193, found 487.2186, $[M+Na]^+$ calculated for $C_{24}H_{30}N_4O_7Na$: 509.2012, found 509.1996.

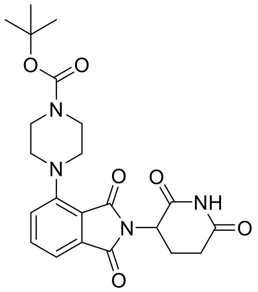

***tert*-butyl 4-(2-(2,6-dioxopiperidin-3-yl)-1,3-dioxoisoindolin-4-yl)piperazine-1-carboxylate, XXIII**: To a solution of **XI** (0.7 mmol, 1.0 eq) in anhydrous DMSO (10 mL), DIPEA (1.4 mmol, 2.0 eq) and boc-piperazine (0.86 mmol, 1.2 eq) were added and the resultant mixture was stirred at 90 °C for 24 h. The reaction mixture was cooled to room temperature then poured in water (30 mL) and washed with EtOAc (2 x 50 mL). The organic layers were combined, washed with sat. brine (2 x 50 mL), dried over $MgSO_4$, filtered, and concentrated *in vacuo* to afford a yellow/orange tar. The crude product was purified by column chromatography (60-80% EtOAc in hexane) to afford compound **XXIII** as yellow solid in a yield of 67%. ^1H NMR (400 MHz, $CDCl_3$) δ 8.03 (s, 1H), 7.61 (dd, J = 8.4, 7.2 Hz, 1H), 7.43 (d, J = 7.2, 0.7 Hz, 1H), 7.16 (d, J = 8.4, 0.6 Hz, 1H), 4.96 (dd, J = 12.4, 5.3 Hz, 1H), 3.65 (t, J = 4.9 Hz, 4H), 3.28 (dd, J = 9.3, 4.4 Hz, 4H), 2.94 – 2.67 (m, 3H), 2.13 (tdd, J = 11.3, 4.5, 1.7 Hz, 1H), 1.48 (s, 9H). ^{13}C NMR (101 MHz, $CDCl_3$) δ 170.91 (s), 168.20 (s), 167.37 (s), 166.72 (s), 154.89 (s), 150.45 (s), 135.91 (s), 134.31 (s), 123.54 (s), 118.05 (s), 116.34 (s), 80.20 (s), 51.42 (s), 49.33 (s), 43.40 (s), 31.56 (s), 28.57 (s), 22.81 (s). HRMS (ESI) m/z: $[M+H]^+$ calculated for $C_{22}H_{27}N_4O_6$: 443.1931, found 443.1930.

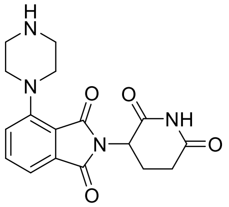

2-(2,6-dioxopiperidin-3-yl)-4-(piperazin-1-yl)isoindoline-1,3-dione, XXIV: TFA was added to a solution of compound **XXIII** (0.12 mmol, 1.0 eq) in DCM (7 mL) at 0°C, then the reaction was stirred at room temperature for 24 h. Then DCM (100 mL) was added to the reaction and washed with sat. solution of sodium bicarbonate (2 x 50 mL) and distilled water (1 x 50 mL). The organic layer was dried over $MgSO_4$, filtered, and the solvent was removed *in vacuo* to afford a yellow solid in a yield of 82% without further purification. ^1H NMR (400 MHz, DMSO) δ 11.09 (s, 1H), 7.69 (dd, J = 8.4, 7.2 Hz, 1H), 7.35 – 7.31 (m, 2H), 5.09 (dd, J = 12.9, 5.4 Hz, 1H), 3.23 – 3.16 (m, 4H), 2.92 – 2.82 (m, 5H), 2.63 – 2.53 (m, 2H), 2.06 – 1.98 (m, 1H). ^{13}C NMR (101 MHz, DMSO) δ 172.80 (s), 170.02 (s), 167.08 (s), 166.28 (s), 150.33 (s), 135.83 (s), 133.71 (s), 123.62 (s), 116.36 (s), 114.58 (s),

51.93 (s), 48.77 (s), 45.53 (s), 30.96 (s), 22.05 (s). HRMS (ESI) m/z: $[M+H]^+$ calculated for $C_{17}H_{19}N_4O_4$: 343.1408, found 343.1406.

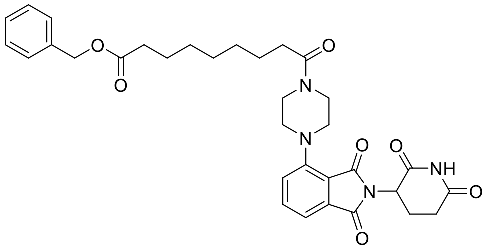

benzyl 9-(4-(2-(2,6-dioxopiperidin-3-yl)-1,3-dioxoisoindolin-4-yl)piperazin-1-yl)-9-oxonanoate, XXV: To a solution of compound **XXVII** (0.48 mmol, 1.1 eq) in anhydrous DMF (7 mL) at 0 °C, DIPEA (0.88 mmol, 2.0 eq) and HATU (0.53 mmol, 1.2 eq) were added. The reaction mixture was stirred for 15 minutes, after which a solution of compound **XXIV** (0.44 mmol, 1.0 eq) in DMF (3 mL) was added slowly and the resultant solution stirred at room temperature for 48 h. Then 100 mL of ethyl acetate was added to the reaction and washed with saturated solution of sodium bicarbonate (2 x 50 mL) and distilled water (2 x 50 mL). The organic layer was dried over $MgSO_4$ and the solvent was concentrated *in vacuo*. The crude product was purified by column chromatography (90% EtOAc in hexane) to afford compound **XXV** as yellow solid in a yield of 54%. ^1H NMR (400 MHz, $CDCl_3$) δ 8.14 (s, 1H), 7.62 (dd, J = 8.3, 7.3 Hz, 1H), 7.45 (d, J = 6.8 Hz, 1H), 7.39 – 7.29 (m, 5H), 7.16 (d, J = 8.0 Hz, 1H), 5.11 (s, 2H), 4.96 (dd, J = 12.3, 5.3 Hz, 1H), 3.91 – 3.67 (m, 4H), 3.41 – 3.24 (m, 4H), 2.94 – 2.68 (m, 3H), 2.35 (m, 4H), 2.15 – 2.09 (m, 1H), 1.69 – 1.59 (m, 4H), 1.39 – 1.28 (m, 6H). ^{13}C NMR (101 MHz, $CDCl_3$) δ 173.75 (s), 171.94 (s), 170.95 (s), 168.23 (s), 167.29 (s), 166.77 (s), 150.08 (s), 136.24 (s), 135.98 (s), 134.28 (s), 128.68 (s), 128.30 (s), 123.52 (s), 118.17 (s), 116.56 (s), 66.22 (s), 52.16 (s), 50.45 (s), 49.35 (s), 45.87 (s), 41.49 (s), 34.39 (s), 33.43 (s), 31.06 (s), 29.38 (s), 29.16 (s), 29.08 (s), 25.35 (s), 24.99 (s), 22.80 (s) HRMS (ESI) m/z: $[M+H]^+$ calculated for $C_{33}H_{39}N_4O_7$: 603.2819, found 603.2817; $[M+Na]^+$ calculated for $C_{33}H_{38}N_4O_7Na$: 625.2638, found 625.2636.

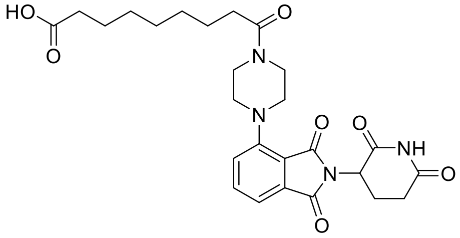

9-(4-(2-(2,6-dioxopiperidin-3-yl)-1,3-dioxoisoindolin-4-yl)piperazin-1-yl)-9-oxononanoic acid, XXVI: To a solution of compound **XXV** (0.52 mmol) in methanol (15mL), 10% Pd/C (0.052 mmol) was added. The flask was evacuated using a Schlenk line (3 times) and filled with nitrogen. A balloon filled with H_2 was added to the reaction, that was then stirred at room temperature for 48 h. The reaction mixture was filtered through celite, and the solvent was removed *in vacuo* to afford compound **XXVI** as a yellow solid in a yield of 95%. ^1H NMR (400 MHz, DMSO-d_6) δ 11.09 (s, 1H), 7.72 (dd, J = 8.3, 7.2 Hz, 1H), 7.39 (d, J = 7.1 Hz, 1H), 7.35 (d, J = 8.4 Hz,

1H), 5.11 (dd, J = 12.9, 5.4 Hz, 1H), 3.65-3.63 (m, 4H), 3.30-3.24 (m, 4H), 2.88 (ddd, J = 17.3, 14.0, 5.3 Hz, 1H), 2.64 – 2.53 (m, 2H), 2.34 (t, J = 7.4 Hz, 2H), 2.16 (t, J = 7.4 Hz, 2H), 2.07 – 1.99 (m, 1H), 1.55 – 1.44 (m, 4H), 1.31 – 1.23 (m, 6H). ^{13}C NMR (101 MHz, DMSO-d_6) δ 174.70 (s), 172.80 (s), 170.86 (s), 169.97 (s), 167.02 (s), 166.35 (s), 149.46 (s), 135.94 (s), 133.61 (s), 123.88 (s), 116.91 (s), 115.22 (s), 50.95 (s), 50.30 (s), 48.82 (s), 44.95 (s), 40.90 (s), 34.02 (s), 32.26 (s), 30.95 (s), 28.67 (s), 28.61 (s), 28.54 (s), 24.77 (s), 24.62 (s), 22.04 (s). HRMS (ESI) m/z: $[M+H]^+$ calculated for $C_{26}H_{33}N_4O_7$: 513.2349, found 513.2338, $[M+Na]^+$ calculated for $C_{26}H_{32}N_4O_7Na$: 535.2169, found 535.2151.

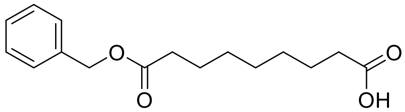

9-(benzyloxy)-9-oxononanoic acid, XXVII: To a solution of azelaic acid (6.36 mmol, 1.0 eq) in 1,4-Dioxane/DMF (1:1 – 8 mL), was added benzyl bromide (6.36 mmol, 1.0 eq), followed by the addition of $NaHCO_3$ (6.51 mmol, 1.02 eq). The resulting suspension was heated at 90 °C for 16 h. The reaction mixture was cool to room temperature and then concentrated *in vacuo* to afford an off-white oil. The crude residue was then suspended in EtOAc (50 mL) and washed with sat. NaCl (2 x 50 mL) and distilled water (2 x 50 mL). The organic layer was dried over $MgSO_4$, and the solvent was concentrated *in vacuo.* The crude product was purified by column chromatography (40% EtOAc in hexane) to afford compound **XXVII** as an off-white solid in a yield of 31%. ^{1}H NMR (400 MHz, $CDCl_3$) δ 7.40 – 7.30 (m, 5H), 5.12 (s, 2H), 2.34 (td, J = 7.5, 5.7 Hz, 4H), 1.68 – 1.57 (m, 4H), 1.32 (m, 6H). ^{13}C NMR (101 MHz, $CDCl_3$) δ 179.58 (s), 173.77 (s), 136.25 (s), 128.69 (s)*, 128.34 (s)*, 66.25 (s), 34.40 (s), 34.03 (s), 28.99 (s), 24.99 (s), 24.70 (s), * 2 x resonace signals coincide at 128.69 and 3 x resonace signals coincide at 128.34. HRMS (ESI) m/z: $[M+Na]^+$ calculated for $C_{16}H_{22}N_4O_4$: 301.1416, found 301.1422.

3.5. Synthesis of the PROTACs **3-6** and **10-13**

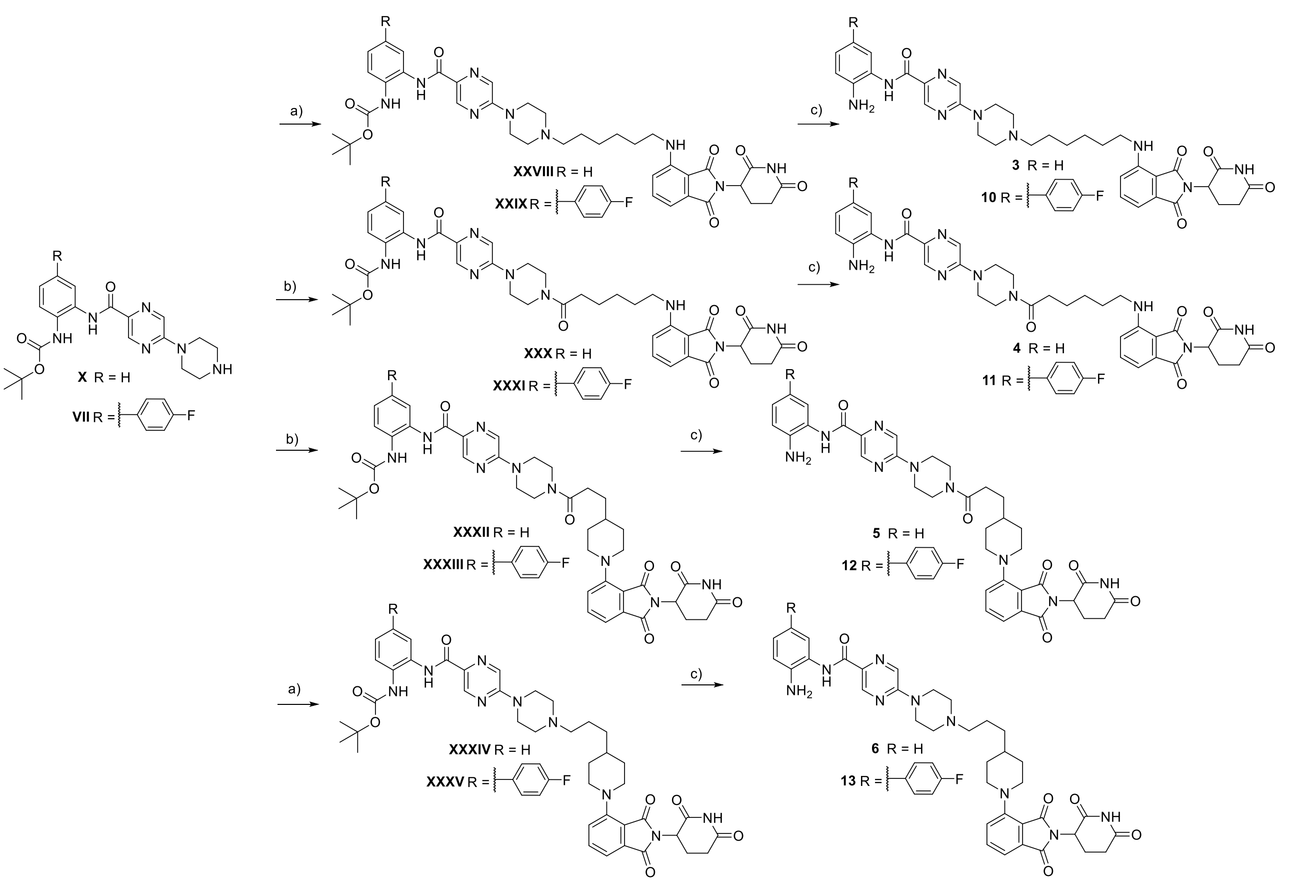

a) **XIV** or **XVII**, DIPEA, acetonitrile, 85°C, 24h; b) HATU, DIPEA, DMF, 48h, r.t.; c) TFA, DCM, r.t. overnight.

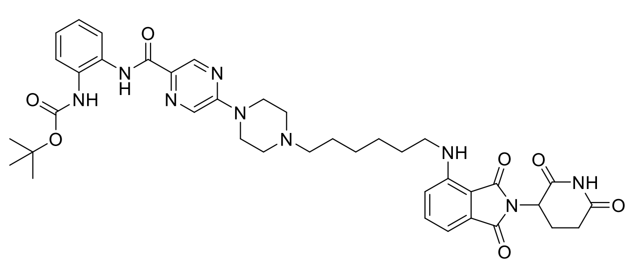

***tert*-butyl (2-(5-(4-(6-((2-(2,6-dioxopiperidin-3-yl)-1,3-dioxoisoindolin-4-yl)amino)hexyl)piperazin-1-yl)pyrazine-2-carboxamido)phenyl)carbamate, XXVIII:** DIPEA (0.18 mmol, 1.5 eq) was added to a solution of compounds **X** (0.12 mmol, 1.0 eq) and **XIV** (0.13 mmol, 1.1 eq) in acetonitrile (10 mL) and the reaction was stirred at 85°C for 24 hours. The solvent was concentrated *in vacuo* and 50 mL of ethyl acetate was added to the crude and washed with distilled water (3 x 50 mL). The organic layer was dried over $MgSO_4$, and the solvent was

concentrated *in vacuo*. The crude product was purified by column chromatography (50-100% EtOAc in hexane) to afford compound **XXVIII** as yellow solid in a yield of 53%. ^{1}H NMR (400 MHz, $CDCl_3$) δ 9.57 (s, 1H), 8.93 (d, *J* = 1.3 Hz, 1H), 8.33 (s, 1H), 7.98 (d, *J* = 1.3 Hz, 1H), 7.67 – 7.56 (m, 2H), 7.49 (dd, *J* = 8.4, 7.3 Hz, 1H), 7.24 – 7.13 (m, 2H), 7.08 (d, *J* = 7.0 Hz, 1H), 6.88 (d, *J* = 8.5 Hz, 1H), 6.23 (t, *J* = 5.4 Hz, 1H), 4.91 (dd, *J* = 12.1, 5.4 Hz, 1H), 3.78 – 3.71 (m, 4H), 3.27 (dd, *J* = 12.7, 6.8 Hz, 2H), 2.92 – 2.70 (m, 3H), 2.58 – 2.53 (m, 4H), 2.43 – 2.36 (m, 2H), 2.16 – 2.07 (m, 1H), 1.73 – 1.64 (m, 2H), 1.60 – 1.52 (m, 2H), 1.50 (s, 9H), 1.47 – 1.36 (m, 4H). ^{13}C NMR (101 MHz, $CDCl_3$) δ 171.15 (s), 169.67 (s), 168.53 (s), 167.72 (s), 162.98 (s), 155.59 (s), 154.00 (s), 147.12 (s), 143.39 (s), 136.27 (s), 132.64 (s), 132.56 (s), 127.72 (s), 126.10 (s), 125.46 (s), 125.01 (s), 124.51 (s), 116.75 (s), 111.57 (s), 110.02 (s), 80.71 (s), 58.53 (s), 52.83 (s), 49.01 (s), 44.44 (s), 42.70 (s), 31.06 (s), 29.32 (s), 28.45 (s), 27.30 (s), 26.97 (s), 26.73 (s), 22.96 (s). HRMS (ESI) m/z: $[M+H]^+$ calculated for $C_{39}H_{48}N_9O_7$: 754.3677, found 754.3684.

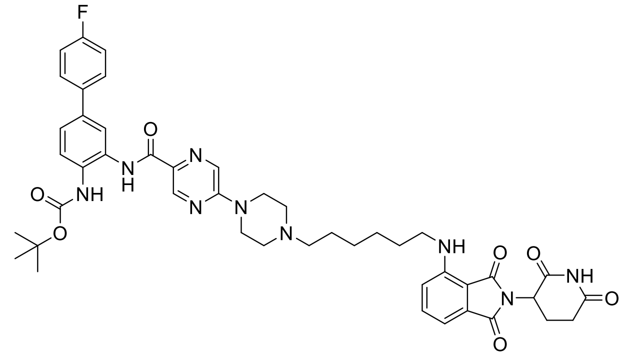

***tert*-butyl (3-(5-(4-(6-((2-(2,6-dioxopiperidin-3-yl)-1,3-dioxoisoindolin-4-yl)amino)hexyl)piperazin-1-yl)pyrazine-2-carboxamido)-4'-fluoro-[1,1'-biphenyl]-4-yl)carbamate, XXIX**: DIPEA (0.27 mmol, 1.5 eq) was added to a solution of compounds **VII** (0.18 mmol, 1.0 eq) and **XIV** (0.21 mmol, 1.2 eq) in acetonitrile (10 mL) and the reaction was stirred at 85 °C for 24 h. The solvent was concentrated *in vacuo* and 50 mL of ethyl acetate was added to the crude and washed with distilled water (3 x 50 mL). The organic layer was dried over $MgSO_4$, and the solvent was concentrated *in vacuo*. The crude product was purified by column chromatography (50-100% EtOAc in hexane) to afford compound **XXIX** as yellow solid in a yield of 37%. ^{1}H NMR (400 MHz, $CDCl_3$) δ 9.64 (s, 1H), 8.95 (d, *J* = 1.3 Hz, 1H), 8.22 (s, 1H), 7.99 (d, *J* = 1.3 Hz, 1H), 7.85 (s, 1H), 7.65 (d, *J* = 8.2 Hz, 1H), 7.56 – 7.52 (m, 3H), 7.37 (dd, *J* = 8.4, 2.1 Hz, 1H), 7.13 – 7.07 (m, 3H), 6.78 (d, *J* = 9.0 Hz, 1H), 6.38 (t, *J* = 5.6 Hz, 1H), 4.92 (dd, *J* = 12.3, 5.4 Hz, 1H), 3.79 – 3.73 (m, 4H), 3.31 – 3.22 (m, 3H), 2.93 – 2.72 (m, 3H), 2.59 – 2.54 (m, 4H), 2.43 – 2.38 (m, 2H), 2.16 – 2.10 (m, 1H), 1.70 – 1.65 (m, 2H), 1.60 – 1.55 (m, 2H), 1.52 (s, 9H), 1.48 – 1.39 (m, 4H). ^{13}C NMR (101 MHz, $CDCl_3$) δ 170.96 (s), 168.26 (s), 165.66 (s), 163.10 (s), 162.61 (d, *J* = 246.4 Hz), 155.62 (s), 154.00 (s), 146.52 (s), 143.46 (s), 140.68 (s), 136.47 (d, *J* = 3.0 Hz), 132.48 (s), 129.41 (s), 128.74 (d, *J* = 8.0 Hz), 127.74 (s), 125.31 (s), 124.49 (s), 122.78 (s), 118.43 (s), 115.73 (d, *J* = 21.5 Hz), 111.64 (s), 103.65 (s), 80.91 (s), 58.52 (s), 52.84 (s), 49.17 (s), 44.46 (s), 42.81 (s), 31.52 (s), 29.25 (s), 28.47

(s), 27.28 (s), 26.94 (s), 26.73 (s), 22.85 (s). HRMS (ESI) m/z: $[M+H]^+$ calculated for $C_{45}H_{51}N_9O_7$: 848.3895, found 848.3929; $[M+Na]^+$ calculated for $C_{45}H_{50}N_9O_7Na$: 870.3715, found 870.3749.

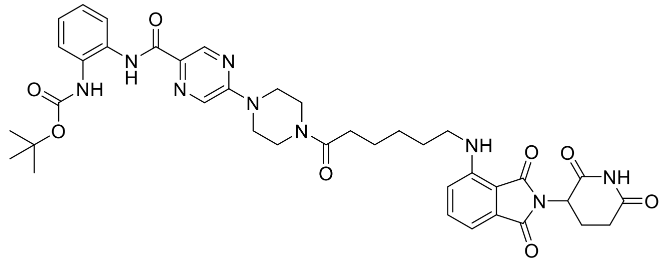

***N*-(2-(3-(tert-butyl)ureido)phenyl)-5-(4-(6-((2-(2,6-dioxopiperidin-3-yl)-1,3-dioxoisoindolin-4-yl)amino)hexanoyl)piperazin-1-yl)pyrazine-2-carboxamide, XXX**: A solution of DIPEA (0.4 mmol, 1.6 eq) and HATU (0.35 mmol, 1.4 eq) were added to a solution of compound **XII** (0.3 mmol, 1.2 eq) in anhydrous DMF (10 mL) and stirred at room temperature for 15 minutes. Later, a solution of compound **X** (0.25 mmol, 1.0 eq) in anhydrous DMF (3 mL) was added dropwise and the reaction was stirred at room temperature for 48 h. Then 100 mL of ethyl acetate was added to the reaction and washed with saturated solution of sodium bicarbonate (2 x 50 mL) and distilled water (1 x 50 mL). The crude product was purified by column chromatography (100% EtOAc) to afford compound **XXX** as yellow solid in a yield of 30%. ^{1}H NMR (400 MHz, $CDCl_3$) δ 9.62 (s, 1H), 8.95 (d, J = 1.3 Hz, 1H), 8.31 (s, 1H), 8.00 (d, J = 1.3 Hz, 1H), 7.67 (s, 1H), 7.56 (d, J = 7.3 Hz, 1H), 7.48 (dd, J = 8.4, 7.3 Hz, 1H), 7.24 – 7.14 (m, 3H), 7.07 (d, J = 6.9 Hz, 1H), 6.88 (d, J = 8.5 Hz, 1H), 6.23 (t, J = 5.5 Hz, 1H), 4.91 (dd, J = 12.1, 5.4 Hz, 1H), 3.83 – 3.77 (m, 4H), 3.73 – 3.59 (m, 4H), 3.29 (dd, J = 12.7, 6.7 Hz, 2H), 2.90 – 2.71 (m, 3H), 2.40 (t, J = 7.4 Hz, 2H), 2.16 – 2.09 (m, 1H), 1.78 – 1.66 (m, 6H), 1.50 (s, 9H). ^{13}C NMR (101 MHz, $CDCl_3$) δ 171.67 (s), 169.67 (s), 168.56 (s), 167.69 (s), 162.70 (s), 155.38 (s), 154.03 (s), 147.07 (s), 143.29 (s), 136.31 (s), 133.44 (s), 132.60 (s), 130.94 (s), 127.74 (s), 126.11 (s), 125.58 (s), 125.13 (s), 124.45 (s), 116.77 (s), 111.62 (s), 110.02 (s), 80.81 (s), 45.00 (s), 44.50 (s), 44.09 (s), 42.46 (s), 40.94 (s), 33.10 (s), 31.53 (s), 29.15 (s), 28.45 (s), 26.77 (s), 24.84 (s), 22.96 (s). HRMS (ESI) m/z: $[M+H]^+$ calculated for $C_{39}H_{46}N_9O_8$: 768.3469, found 768.3464.

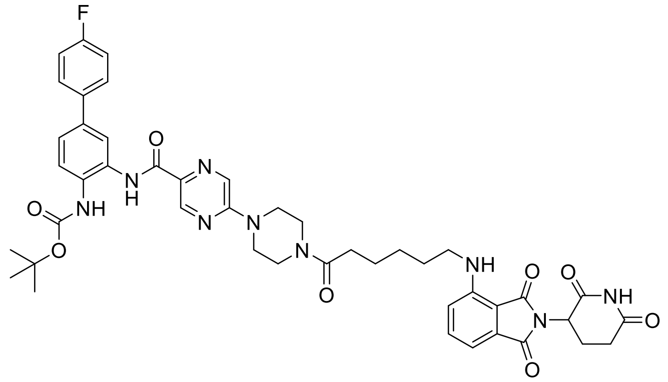

***tert*-butyl (3-(5-(4-(6-((2-(2,6-dioxopiperidin-3-yl)-1,3-dioxoisoindolin-4-yl)amino)hexanoyl)piperazin-1-yl)pyrazine-2-carboxamido)-4'-fluoro-[1,1'-biphenyl]-4-yl)carbamate, XXXI**: A solution of DIPEA (0.61 mmol, 2.6 eq) and HATU (0.3 mmol, 1.2 eq) were added to a solution of compound **XII** (0.25 mmol, 1.1 eq) in anhydrous DMF (10 mL) and stirred at room temperature for 15 minutes. Later, a solution of compound **VII** (0.23 mmol, 1.0 eq) in anhydrous DMF (3 mL) was added dropwise and the reaction was stirred at room temperature for 48 h. Then 100 mL of ethyl acetate was added to the reaction and washed with saturated solution of sodium bicarbonate (2 x 50 mL) and distilled water (1 x 50 mL). The crude product was purified by column chromatography (80-95% EtOAc in petroleum ether) to afford compound **XXXI** as yellow solid in a yield of 55.3%. ^{1}H NMR (400 MHz, $CDCl_3$) δ 9.69 (s, 1H), 8.97 (d, J = 1.3 Hz, 1H), 8.25 (s, 1H), 8.01 (d, J = 1.4 Hz, 1H), 7.90 (s, 1H), 7.63 (d, J = 8.2 Hz, 1H), 7.49 (dd, J = 8.3, 7.3 Hz, 1H), 7.38 (dd, J = 8.4, 2.1 Hz, 1H), 7.17 (s, 1H), 7.13 – 7.06 (m, 3H), 6.88 (d, J = 8.5 Hz, 1H), 6.23 (t, J = 5.5 Hz, 1H), 4.91 (dd, J = 12.1, 5.4 Hz, 1H), 3.85 – 3.77 (m, 4H), 3.74 – 3.60 (m, 4H), 3.30 (dd, J = 12.6, 6.7 Hz, 2H), 2.91 – 2.71 (m, 3H), 2.40 (t, J = 7.4 Hz, 2H), 2.16 – 2.10 (m, 1H), 1.79 – 1.68 (m, 4H), 1.57 – 1.47 (m, 11H). ^{13}C NMR (101 MHz, $CDCl_3$) δ 171.67 (s), 171.14 (s), 169.68 (s), 168.54 (s), 167.69 (s), 165.65 (s), 162.83 (s), 162.62 (d, J = 246.7 Hz), 155.41 (s), 154.02 (s), 147.08 (s), 143.36 (s), 136.44 (s), 136.32 (s), 133.36 (s), 132.61 (s), 128.74 (d, J = 8.1 Hz), 127.76 (s), 125.41 (s), 124.57 (s), 122.85 (s), 116.78 (s), 115.75 (d, J = 21.5 Hz), 111.65 (s), 110.03 (s), 81.00 (s), 49.01 (s), 45.02 (s), 44.51 (s), 44.10 (s), 42.47 (s), 40.98 (s), 33.11 (s), 31.54 (s), 29.15 (s), 28.46 (s), 26.78 (s), 24.85 (s), 22.97 (s). ^{19}F NMR (376 MHz, $CDCl_3$) δ -115.59 (s). HRMS (ESI) m/z: $[M+H]^+$ calculated for $C_{45}H_{49}N_9O_8F$: 862.3688, found 862.3690, $[M+Na]^+$ calculated for $C_{45}H_{48}N_9O_8FNa$: 884.3508, found 884.3509.

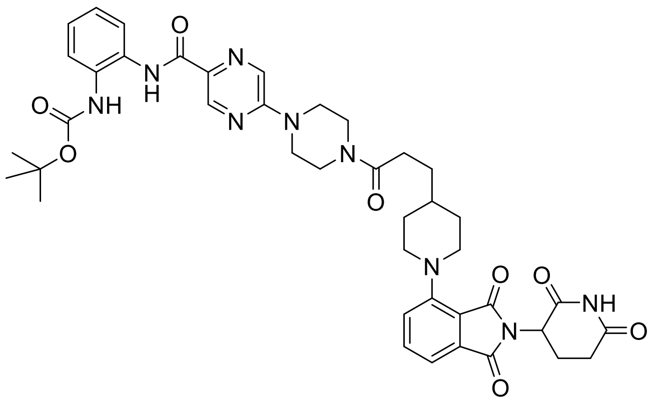

***N*-(2-(3-(tert-butyl)ureido)phenyl)-5-(4-(3-(1-(2-(2,6-dioxopiperidin-3-yl)-1,3-dioxoisoindolin-4-yl)piperidin-4-yl)propanoyl)piperazin-1-yl)pyrazine-2-carboxamide, XXXII**: A solution of DIPEA (0.33 mmol, 1.5 eq) and HATU (0.28 mmol, 1.3 eq) were added to a solution of compound **XV** (0.24 mmol, 1.1 eq) in anhydrous DMF (10 mL) and stirred at room temperature for 15 minutes. Later, a solution of compound **X** (0.22 mmol, 1.0 eq) in anhydrous DMF (3 mL) was added dropwise and the reaction was stirred at room temperature for 48 h. Then 100 mL of ethyl acetate was added to the reaction and washed with saturated solution of sodium

bicarbonate (2 x 50 mL) and distilled water (1 x 50 mL). The crude product was purified by column chromatography (100% EtOAc) to afford compound **XXXII** as yellow solid in a yield of 63%. ^{1}H NMR (400 MHz, $CDCl_3$) δ 9.63 (s, 1H), 8.96 (d, *J* = 1.3 Hz, 1H), 8.13 (s, 1H), 8.01 (d, *J* = 1.4 Hz, 1H), 7.67 (d, *J* = 3.6 Hz, 1H), 7.59 – 7.54 (m, 2H), 7.36 (dd, *J* = 8.8, 2.1 Hz, 1H), 7.24 – 7.11 (m, 3H), 4.96 (dd, *J* = 12.3, 5.3 Hz, 1H), 3.89 – 3.62 (m, 10H), 2.92 – 2.68 (m, 5H), 2.47 – 2.43 (m, 2H), 2.14 – 2.07 (m, 1H), 1.87 (d, *J* = 9.0 Hz, 2H), 1.74 – 1.68 (m, 2H), 1.57 – 1.53 (m, 3H), 1.51 (s, 9H). ^{13}C NMR (101 MHz, $CDCl_3$) δ 171.89 (s), 171.05 (s), 168.34 (s), 167.53 (s), 166.87 (s), 162.71 (s), 155.40 (s), 154.01 (s), 150.99 (s), 143.32 (s), 135.65 (s), 134.25 (s), 133.49 (s), 127.70 (s), 126.17 (s), 125.62 (s), 125.12 (s), 124.43 (s), 123.78 (s), 117.33 (s), 115.49 (s), 80.82 (s), 52.14 (s), 51.80 (s), 49.24 (s), 45.04 (s), 44.53 (s), 44.09 (s), 40.97 (s), 35.16 (s), 32.27 (s), 31.66 (s), 31.55 (s), 31.07 (s), 30.42 (s), 28.45 (s), 22.83 (s). HRMS (ESI) m/z: $[M+H]^+$ calculated for $C_{41}H_{48}N_9O_8$: 794.3626, found 794.3630, $[M+Na]^+$ calculated for $C_{41}H_{47}N_9O_8Na$: 816.3445, found 816.3453.

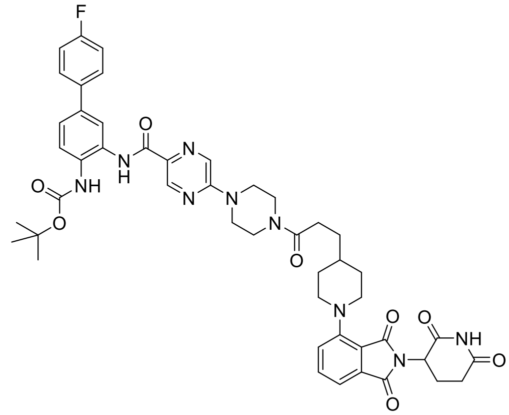

***N*-(4-(3-(tert-butyl)ureido)-4'-fluoro-[1,1'-biphenyl]-3-yl)-5-(4-(3-(1-(2-(2,6-dioxopiperidin-3-yl)-1,3-dioxoisoindolin-4-yl)piperidin-4-yl)propanoyl)piperazin-1-yl)pyrazine-2-carboxamide, XXXIII**: A solution of DIPEA (0.5 mmol, 2.8 eq) and HATU (0.25 mmol, 1.4 eq) were added to a solution of compound **XV** (0.21 mmol, 1.2 eq) in anhydrous DMF (10 mL) and stirred at room temperature for 15 minutes. Later, a solution of compound **VII** (0.18 mmol, 1.0 eq) in anhydrous DMF (3 mL) was added dropwise and the reaction was stirred at room temperature for 48 hours. Then 100 mL of ethyl acetate was added to the reaction and washed with sat. solution of sodium bicarbonate (2 x 50 mL) and distilled water (1 x 50 mL). The crude product was purified by column chromatography (100% EtOAc) to afford compound **XXXIII** as yellow solid in a yield of 53%. ^{1}H NMR (400 MHz, $CDCl_3$) δ 9.69 (s, 1H), 8.98 (d, *J* = 1.3 Hz, 1H), 8.11 (s, 1H), 8.02 (d, *J* = 1.4 Hz, 1H), 7.90 (s, 1H), 7.62 (d, *J* = 8.5 Hz, 1H), 7.58 – 7.52 (m, 3H), 7.40 – 7.35 (m, 2H), 7.17 (d, *J* = 8.1 Hz, 1H), 7.10 (t, *J* = 8.7 Hz, 2H), 4.96 (dd, *J* = 12.3, 5.3 Hz, 1H), 3.92 – 3.54 (m, 10H), 2.96 – 2.67 (m, 7H), 2.48 – 2.43 (m, 2H), 2.14 – 2.08 (m, 1H), 1.87 (d, *J* = 9.6 Hz, 2H), 1.71 (dd, *J* = 14.2, 7.4 Hz, 2H), 1.53 (s, 12H). ^{13}C NMR (101 MHz, $CDCl_3$) δ 171.89 (s), 171.03 (s), 168.32 (s), 167.52 (s), 166.88 (s), 162.83 (s), 162.63 (d, *J* = 246.3 Hz), 155.43 (s), 154.01 (s), 150.99 (s), 143.37 (s), 137.72 (s), 136.42 (d, *J* = 3.3 Hz), 135.66 (s), 134.25 (s), 133.39 (s), , 128.74 (d, *J* =

8.0 Hz), 127.71 (s), 125.38 (s), 124.61 (s), 123.79 (s), 122.93 (s), 117.34 (s), 115.75 (d, J = 21.4 Hz), 115.50 (s), 81.00 (s), 52.14 (s), 51.81 (s), 49.25 (s), 45.04 (s), 44.53 (s), 44.12 (s), 40.98 (s), 35.16 (s), 32.27 (s), 31.66 (s), 31.56 (s), 31.07 (s), 30.42 (s), 28.47 (s), 22.83 (s). ^{19}F NMR (376 MHz, $CDCl_3$) δ -115.57 (s).

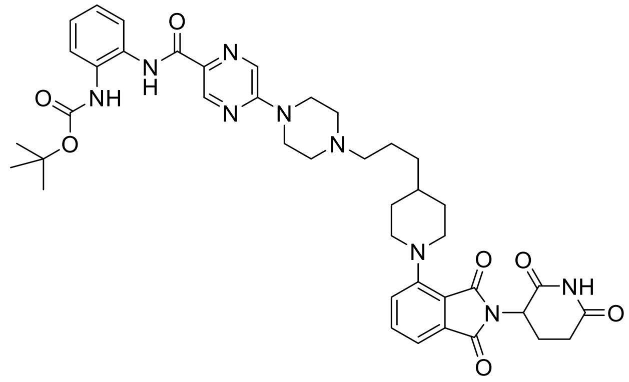

***tert*-butyl (2-(5-(4-(3-(1-(2-(2,6-dioxopiperidin-3-yl)-1,3-dioxoisoindolin-4-yl)piperidin-4-yl)propyl)piperazin-1-yl)pyrazine-2-carboxamido)phenyl)carbamate, XXXIV**: DIPEA (0.37 mmol, 1.5 eq) was added to a solution of compounds **X** (0.25 mmol, 1.0 eq) and **XVII** (0.30 mmol, 1.2 eq) in acetonitrile (10 mL) and the reaction was stirred at 85 °C for 24 h. The solvent was concentrated *in vacuo* and 50 mL of ethyl acetate was added to the crude and washed with distilled water (3 x 50 mL). The organic layer was dried over $MgSO_4$, and the solvent was concentrated *in vacuo*. The crude product was purified by column chromatography (2% methanol in EtOAc in hexane) to afford compound **XXXIV** as yellow solid in a yield of 40%. ^{1}H NMR (400 MHz, $CDCl_3$) δ 9.58 (s, 1H), 8.93 (d, J = 1.3 Hz, 1H), 8.24 (s, 1H), 7.99 (d, J = 1.3 Hz, 1H), 7.66 – 7.60 (m, 2H), 7.56 (dd, J = 8.4, 7.2 Hz, 1H), 7.35 (d, J = 6.7 Hz, 1H), 7.23 – 7.15 (m, 3H), 4.96 (dd, J = 12.2, 5.4 Hz, 1H), 3.81 – 3.70 (m, 6H), 2.93 – 2.70 (m, 5H), 2.59 (s, 4H), 2.47 – 2.34 (m, 2H), 2.14 – 2.07 (m, 1H), 1.84 (d, J = 9.6 Hz, 2H), 1.64 – 1.58 (m, 2H), 1.50 (s, 9H), 1.48 (s, 2H), 1.39 – 1.32 (m, 2H). ^{13}C NMR (101 MHz, $CDCl_3$) δ 171.11 (s), 168.39 (s), 167.56 (s), 166.85 (s), 162.96 (s), 155.56 (s), 155.56 (s), 154.00 (s), 154.00 (s), 151.06 (s), 151.06 (s), 143.40 (s), 135.60 (s), 134.26 (s), 132.64 (s), 127.71 (s), 126.12 (s), 125.48 (s), 125.01 (s), 124.47 (s), 123.78 (s), 117.25 (s), 115.37 (s), 80.72 (s), 58.87 (s), 52.79 (s), 52.27 (s), 51.94 (s), 49.24 (s), 44.35 (s), 35.56 (s), 34.35 (s), 32.51 (s), 32.48 (s), 31.56 (s), 28.46 (s), 23.95 (s), 22.83 (s). HRMS (ESI) m/z: $[M+H]^+$ calculated for $C_{41}H_{50}N_9O_7$: 780.3833, found 780.3835, $[M+Na]^+$ calculated for $C_{41}H_{49}N_9O_7Na$: 802.3653, found 802.3657.

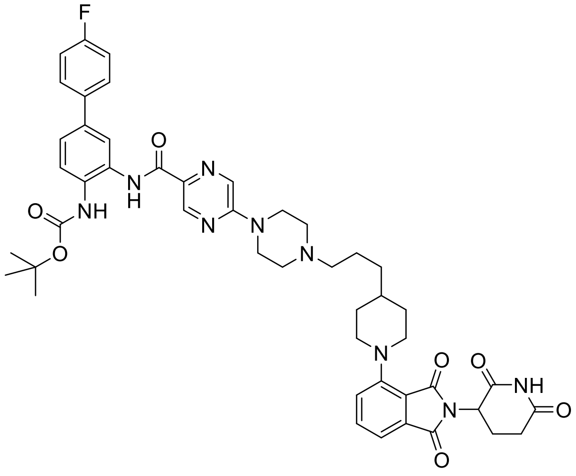

***tert*-butyl (3-(5-(4-(3-(1-(2-(2,6-dioxopiperidin-3-yl)-1,3-dioxoisoindolin-4-yl)piperidin-4-yl)propyl)piperazin-1-yl)pyrazine-2-carboxamido)-4'-fluoro-[1,1'-biphenyl]-4-yl)carbamate, XXXV**: DIPEA (0.27 mmol, 1.5 eq) was added to a solution of compounds **VII** (0.18 mmol, 1.0 eq) and **XVII** (0.21 mmol, 1.2 eq) in acetonitrile (10 mL) and the reaction was stirred at 85°C for 24 hours. The solvent was concentrated *in vacuo* and 50 mL of ethyl acetate was added to the crude mixture and washed with distilled water (3 x 50 mL). The organic layer was dried over $MgSO_4$, and the solvent was concentrated *in vacuo*. The crude product was purified by column chromatography (2% methanol in EtOAc in hexane) to afford compound **XXXV** as yellow solid in a yield of 36%. ^{1}H NMR (400 MHz, $CDCl_3$) δ 9.65 (s, 1H), 8.94 (d, J = 1.3 Hz, 1H), 8.24 (s, 1H), 7.99 (d, J = 1.3 Hz, 1H), 7.86 (s, 1H), 7.65 (d, J = 8.1 Hz, 1H), 7.58 – 7.51 (m, 3H), 7.39 – 7.34 (m, 2H), 7.17 (d, J = 8.1 Hz, 1H), 7.12 – 7.06 (m, 2H), 4.96 (dd, J = 12.2, 5.4 Hz, 1H), 3.80 – 3.71 (m, 6H), 2.92 – 2.70 (m, 5H), 2.60 – 2.53 (m, 4H), 2.44 – 2.38 (m, 2H), 2.18 – 2.08 (m, 1H), 1.89 – 1.80 (m, 2H), 1.63 – 1.56 (m, 2H), 1.52 (s, 9H), 1.49 – 1.46 (m, 3H), 1.39 – 1.32 (m, 2H). ^{13}C NMR (101 MHz, $CDCl_3$) δ 171.09 (s), 168.37 (s), 167.55 (s), 166.85 (s), 163.10 (s), 162.60 (d, J = 246.2 Hz), 155.62 (s), 153.99 (s), 151.07 (s), 143.46 (s), 137.56 (s), 136.46 (d, J = 3.1 Hz), 135.60 (s), 134.27 (s), 132.45 (s), 128.73 (d, J = 8.1 Hz), 127.72 (s), 125.34 (s), 124.51 (s), 123.78 (s), 122.82 (s), 117.25 (s), 115.72 (d, J = 21.4 Hz), 115.37 (s), 80.89 (s), 58.92 (s), 52.86 (s), 52.28 (s), 51.95 (s), 49.24 (s), 44.47 (s), 35.59 (s), 34.38 (s), 32.52 (s), 32.50 (s), 31.56 (s), 28.46 (s), 24.09 (s), 22.84 (s). HRMS (ESI) m/z: $[M+H]^+$ calculated for $C_{47}H_{53}N_9O_7F$: 874.4052, found 874.4051; $[M+Na]^+$ calculated for $C_{47}H_{52}N_9O_7FNa$: 896.3871, found 896.3870.

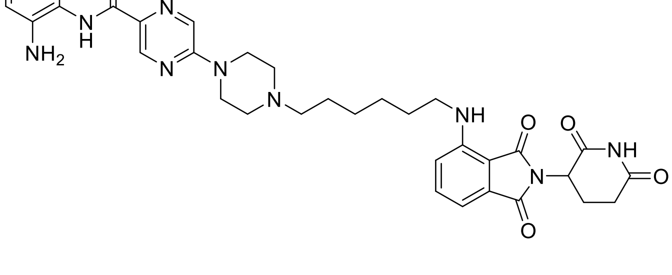

***N*-(2-aminophenyl)-5-(4-(6-((2-(2,6-dioxopiperidin-3-yl)-1,3-dioxoisoindolin-4-yl)amino)hexyl)piperazin-1-yl)pyrazine-2-carboxamide, 3**: TFA (0.16 mmol, 3.0 eq) was added to a solution of compound **XXVIII** (0.05 mmol, 1.0 eq) in DCM (10 mL) and the reaction was stirred at room temperature overnight. Then, 50 mL of ethyl acetate was added and washed with saturated solution of sodium bicarbonate (2 x 50 mL) and distilled water (1 x 50 mL). The organic layer was dried over $MgSO_4$, and the solvent was concentrated *in vacuo*. The crude product was purified by column chromatography (5% methanol in EtOAc) to afford compound **3** as yellow solid in a yield of 72%. ^{1}H NMR (400 MHz, $CDCl_3$) δ 9.25 (s, 1H), 8.91 (d, *J* = 1.3 Hz, 1H), 8.00 (d, *J* = 1.3 Hz, 1H), 7.48 (dd, *J* = 8.4, 7.2 Hz, 1H), 7.42 (dd, *J* = 8.2, 1.4 Hz, 1H), 7.10 – 7.04 (m, 2H), 6.88 (d, *J* = 8.5 Hz, 1H), 6.86 – 6.81 (m, 2H), 6.23 (t, *J* = 5.5 Hz, 1H), 4.90 (dd, *J* = 12.1, 5.4 Hz, 1H), 3.78 – 3.72 (m, 4H), 3.27 (dd, *J* = 12.7, 6.8 Hz, 2H), 2.91 – 2.70 (m, 3H), 2.61 – 2.54 (m, 4H), 2.45 – 2.39 (m, 2H), 2.18 – 2.09 (m, 1H), 1.68 (dt, *J* = 14.5, 7.1 Hz, 2H), 1.56 (dt, *J* = 14.8, 7.5 Hz, 2H), 1.50 – 1.37 (m, 4H). ^{13}C NMR (101 MHz, $CDCl_3$) δ 171.24 (s), 169.67 (s), 168.60 (s), 167.73 (s), 162.40 (s), 155.52 (s), 147.11 (s), 143.14 (s), 140.58 (s), 136.27 (s), 132.78 (s), 132.63 (s), 127.76 (s), 126.81 (s), 124.84 (s), 124.60 (s), 119.67 (s), 118.16 (s), 116.75 (s), 111.56 (s), 110.01 (s), 58.50 (s), 52.77 (s), 49.01 (s), 44.35 (s), 42.68 (s), 31.55 (s), 29.29 (s), 27.27 (s), 26.94 (s), 26.62 (s), 22.95 (s). HRMS (ESI) m/z: $[M+H]^+$ calculated for $C_{34}H_{40}N_9O_5$: 654.3152, found 654.3152; $[M+Na]^+$ calculated for $C_{34}H_{39}N_9O_5Na$: 676.2972, found 676.2970.

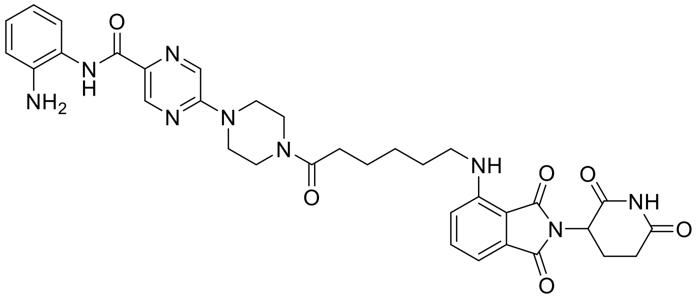

***N*-(2-aminophenyl)-5-(4-(6-((2-(2,6-dioxopiperidin-3-yl)-1,3-dioxoisoindolin-4- yl)amino) hexanoyl) piperazin-1-yl)pyrazine-2-carboxamide, 4**: TFA (1.2 mmol, 20 eq) was added to a solution of compound **XXX** (0.06 mmol, 1.0 eq) in DCM (10 mL) and the reaction was stirred at room temperature overnight. Then, 50 mL of ethyl acetate was added and washed with sat. sodium bicarbonate (2 x 50 mL) and distilled water (1 x 50 mL). The organic layer was dried over $MgSO_4$, and the solvent was concentrated *in vacuo*. The crude product was purified by column chromatography (100% EtOAc) to afford compound **4** as yellow solid in a yield of 49%. ^{1}H NMR (400 MHz, DMSO-d_6) δ 11.09 (s, 1H), 9.63 (s, 1H), 8.73 (d, *J* = 1.3 Hz, 1H), 8.36 (d, *J* = 1.3 Hz, 1H), 7.58 (dd, *J* = 8.4, 7.2 Hz, 1H), 7.47 (dd, *J* = 7.9, 1.3 Hz, 1H), 7.10 (d, *J* = 8.6 Hz, 1H), 7.01 (d, *J* = 6.9 Hz, 1H), 6.97 – 6.90 (m, 1H), 6.81 (dd, *J* = 8.0, 1.4 Hz, 1H), 6.64 (td, *J* = 7.7, 1.4 Hz, 1H), 6.54 (t, *J* = 4.8 Hz, 1H), 5.05 (dd, *J* = 12.8, 5.4 Hz, 1H), 4.85 (s, 1H), 3.79 – 3.70 (m, 3H), 3.63 – 3.58 (m, 3H), 3.31 – 3.22 (m, 2H), 2.93 – 2.83 (m, 1H), 2.64 – 2.52 (m, 2H), 2.39 (t, *J* = 7.3 Hz, 2H), 2.07 – 1.98 (m, 1H), 1.59 (tt, *J* = 14.7, 7.5 Hz, 4H), 1.43 – 1.34

(m, 2H). ^{13}C NMR (101 MHz, DMSO-d_6) δ 172.81 (s), 170.88 (s), 170.10 (s), 168.95 (s), 167.29 (s), 161.85 (s), 154.93 (s), 146.43 (s), 141.92 (s), 141.60 (s), 136.29 (s), 132.93 (s), 132.19 (s), 128.83 (s), 125.61 (s), 124.42 (s), 124.30 (s), 117.20 (s), 117.06 (s), 116.79 (s), 110.37 (s), 108.99 (s), 48.54 (s), 44.21 (s), 43.91 (s), 43.72 (s), 41.75 (s), 40.41 (s), 32.15 (s), 30.98 (s), 30.69 (s), 28.56 (s), 26.06 (s), 24.44 (s), 22.16 (s). HRMS (ESI) m/z: $[M+H]^+$ calculated for $C_{34}H_{38}N_9O_6$: 668.2945, found 668.2954.

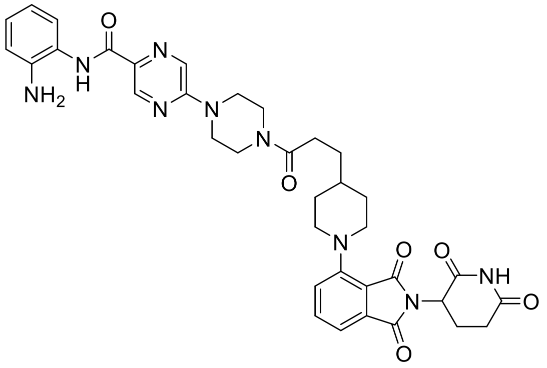

***N*-(2-aminophenyl)-5-(4-(3-(1-(2-(2,6-dioxopiperidin-3-yl)-1,3-dioxoisoindolin-4-yl)piperidin-4-yl)propanoyl)piperazin-1-yl)pyrazine-2-carboxamide, 5**: TFA (1.2 mmol, 20 eq) was added to a solution of compound **XXXII** (0.06 mmol, 1.0 eq) in DCM (10 mL) and the reaction was stirred at room temperature overnight. Then, 50 mL of ethyl acetate was added and washed with sat. sodium bicarbonate (2 x 50 mL) and distilled water (1 x 50 mL). The organic layer was dried over $MgSO_4$, and the solvent was concentrated *in vacuo*. The crude product was purified by column chromatography (5% methanol in EtOAc) to afford compound **5** as yellow solid in a yield of 82%. ^{1}H NMR (400 MHz, $CDCl_3$) δ 9.27 (s, 1H), 8.95 (d, *J* = 1.1 Hz, 1H), 8.22 (s, 1H), 8.04 (d, *J* = 1.2 Hz, 1H), 7.56 (dd, *J* = 8.3, 7.2 Hz, 1H), 7.43 (dd, *J* = 8.2, 1.3 Hz, 1H), 7.36 (d, *J* = 7.1 Hz, 1H), 7.16 (d, *J* = 8.4 Hz, 1H), 7.10 – 7.05 (m, 1H), 6.87 – 6.82 (m, 2H), 4.95 (dd, *J* = 12.2, 5.4 Hz, 1H), 3.99 (s, 2H), 3.86 – 3.62 (m, 10H), 2.94 – 2.66 (m, 5H), 2.48 – 2.43 (m, 2H), 2.15 – 2.07 (m, 1H), 1.86 (d, *J* = 9.3 Hz, 2H), 1.74 – 1.64 (m, 2H), 1.59 – 1.49 (m, 3H). ^{13}C NMR (101 MHz, $CDCl_3$) δ 171.89 (s), 171.11 (s), 168.38 (s), 167.53 (s), 166.86 (s), 162.16 (s), 155.35 (s), 150.98 (s), 143.09 (s), 140.53 (s), 135.64 (s), 134.24 (s), 133.59 (s), 127.74 (s), 126.92 (s), 124.80 (s), 124.51 (s), 123.78 (s), 119.74 (s), 118.24 (s), 117.32 (s), 115.48 (s), 52.13 (s), 51.80 (s), 49.23 (s), 45.05 (s), 44.50 (s), 44.09 (s), 40.97 (s), 35.15 (s), 32.25 (s), 31.66 (s), 31.54 (s)*, 30.41 (s), 22.82 (s), *2 x resonace signals coincide at 31.54. HRMS (ESI) m/z: $[M+H]^+$ calculated for $C_{36}H_{40}N_9O_6$: 694.3102, found 694.3102, $[M+Na]^+$ calculated for $C_{36}H_{39}N_9O_6Na$: 716.2921, found 716.2916.

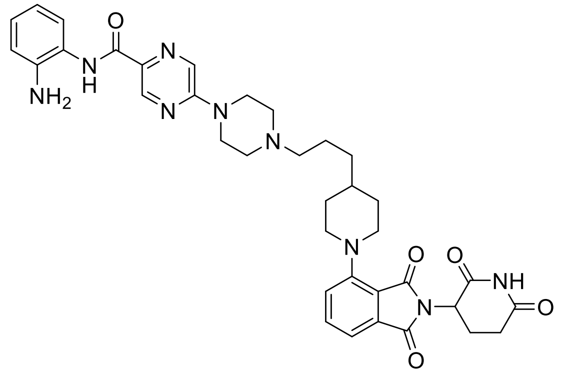

***N*-(2-aminophenyl)-5-(4-(3-(1-(2-(2,6-dioxopiperidin-3-yl)-1,3-dioxoisoindolin-4-yl)piperidin-4-yl)propyl)piperazin-1-yl)pyrazine-2-carboxamide, 6:** TFA (1.2 mmol, 20 eq) was added to a solution of compound **XXXIV** (0.06 mmol, 1.0 eq) in DCM (10 mL) and the reaction was stirred at room temperature overnight. Then, 50 mL of ethyl acetate was added and washed with sat. sodium bicarbonate (2 x 50 mL) and distilled water (1 x 50 mL). The organic layer was dried over $MgSO_4$, and the solvent was concentrated *in vacuo*. The crude product was purified by column chromatography (5% methanol in EtOAc) to afford compound **6** as yellow solid in a yield of 68%. ^{1}H NMR (400 MHz, $CDCl_3$) δ 9.26 (s, 1H), 8.92 (d, *J* = 1.4 Hz, 1H), 8.27 (s, 1H), 8.01 (d, *J* = 1.4 Hz, 1H), 7.56 (dd, *J* = 8.4, 7.2 Hz, 1H), 7.42 (dd, *J* = 8.3, 1.4 Hz, 1H), 7.35 (dd, *J* = 7.1, 0.6 Hz, 1H), 7.17 (d, *J* = 8.0 Hz, 1H), 7.09 – 7.04 (m, 1H), 6.86 – 6.81 (m, 2H), 4.96 (dd, *J* = 12.2, 5.4 Hz, 1H), 3.98 (s, 2H), 3.75 (dd, *J* = 14.3, 8.8 Hz, 6H), 2.93 – 2.67 (m, 5H), 2.60 – 2.55 (m, 4H), 2.44 – 2.38 (m, 2H), 2.15 – 2.06 (m, 1H), 1.84 (d, *J* = 9.3 Hz, 2H), 1.65 – 1.55 (m, 2H), 1.52 – 1.43 (m, 3H), 1.39 – 1.32 (m, 2H). ^{13}C NMR (101 MHz, $CDCl_3$) δ 171.12 (s), 168.39 (s), 167.56 (s), 166.85 (s), 162.40 (s), 155.56 (s), 151.07 (s), 143.16 (s), 140.57 (s), 135.59 (s), 134.27 (s), 132.76 (s), 127.73 (s), 126.80 (s), 124.82 (s), 124.62 (s), 123.78 (s), 119.68 (s), 118.18 (s), 117.24 (s), 115.36 (s), 58.92 (s), 52.86 (s), 52.28 (s), 51.96 (s), 49.24 (s), 44.45 (s), 35.58 (s), 34.39 (s), 32.52 (s), 32.49 (s), 31.56 (s), 24.08 (s), 22.83 (s). HRMS (ESI) m/z: $[M+H]^+$ calculated for $C_{36}H_{42}N_9O_5$: 680.3309, found 680.3314; $[M+Na]^+$ calculated for $C_{36}H_{41}N_9O_5Na$: 702.3128, found 702.3132.

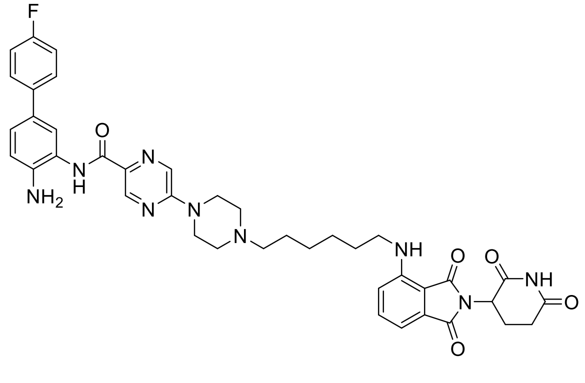

***N*-(4-amino-4'-fluoro-[1,1'-biphenyl]-3-yl)-5-(4-(6-((2-(2,6-dioxopiperidin-3-yl)-1,3-dioxoisoindolin-4-yl)amino)hexyl)piperazin-1-yl)pyrazine-2-carboxamide, 10**: TFA (1.2 mmol, 20 eq) was added to a solution of

compound **XXIX** (0.06 mmol, 1.0 eq) in DCM (10 mL) and the reaction was stirred at room temperature overnight. Then, 50 mL of ethyl acetate was added and washed with saturated solution of sodium bicarbonate (2 x 50mL) and distilled water (1 x 50 mL). The organic layer was dried over $MgSO_4$, and the solvent was concentrated *in vacuo*. The crude product was purified by column chromatography (2% methanol in DCM) to afford compound **10** as yellow solid in a yield of 13%. ^{1}H NMR (400 MHz, $CDCl_3$) δ 9.31 (s, 1H), 8.94 (d, *J* = 1.3 Hz, 1H), 8.14 (s, 1H), 8.02 (d, *J* = 1.4 Hz, 1H), 7.67 (d, *J* = 2.1 Hz, 1H), 7.54 (d, *J* = 9.2 Hz, 1H), 7.53 – 7.48 (m, 2H), 7.27 (d, *J* = 2.1 Hz, 1H), 7.10 – 7.04 (m, 3H), 6.89 (d, *J* = 8.2 Hz, 1H), 6.78 (d, *J* = 9.0 Hz, 1H), 6.38 (t, *J* = 5.5 Hz, 1H), 4.92 (dd, *J* = 12.2, 5.4 Hz, 1H), 4.03 (s, 2H), 3.79 – 3.73 (m, 4H), 3.31 – 3.23 (m, 2H), 2.93 – 2.72 (m, 3H), 2.61 – 2.52 (m, 4H), 2.44 – 2.37 (m, 2H), 2.18 – 2.10 (m, 1H), 1.74 – 1.65 (m, 2H), 1.61 – 1.52 (m, 2H), 1.49 – 1.38 (m, 4H). ^{13}C NMR (101 MHz, $CDCl_3$) δ 170.89 (s), 168.28 (s), 168.21 (s), 165.66 (s), 162.53 (s), 162.17 (d, *J* = 245.5 Hz), 155.60 (s), 146.53 (s), 143.23 (s), 140.69 (s), 139.81 (s), 136.95 (d, *J* = 3.2 Hz), 131.98 (s), 129.42 (s), 128.22 (d, *J* = 7.9 Hz), 127.76 (s), 125.25 (s), 123.27 (s), 118.52 (s), 118.43 (s), 115.59 (d, *J* = 21.4 Hz), 111.65 (s), 58.54 (s), 52.86 (s), 49.18 (s), 44.48 (s), 42.82 (s), 31.52 (s), 29.26 (s), 27.30 (s), 26.95 (s), 26.76 (s), 22.86 (s). ^{19}F NMR (376 MHz, $CDCl_3$) δ -116.90 (s). HRMS (ESI) m/z: $[M+H]^+$ calculated for $C_{40}H_{43}N_9O_5F$: 748.3371, found 748.3372; $[M+Na]^+$ calculated for $C_{40}H_{42}N_9O_5FNa$: 770.3191, found 770.3195.

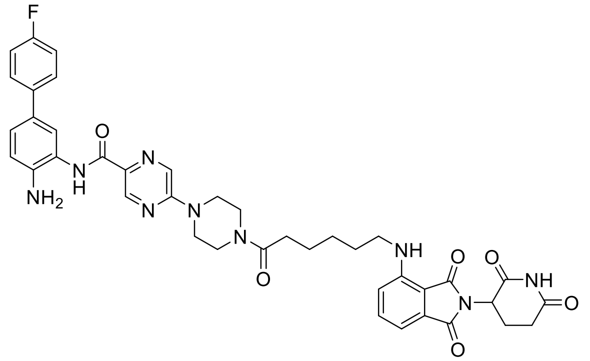

***N*-(4-amino-4'-fluoro-[1,1'-biphenyl]-3-yl)-5-(4-(6-((2-(2,6-dioxopiperidin-3-yl)-1,3-dioxoisoindolin-4-yl)amino)hexanoyl)piperazin-1-yl)pyrazine-2-carboxamide, 11**: TFA (0.9 mmol, 20 eq) was added to a solution of compound **XXXI** (0.046 mmol, 1.0 eq) in DCM (10 mL) and the reaction was stirred at room temperature overnight. Then, 50 mL of ethyl acetate was added and washed with saturated solution of sodium bicarbonate (2 x 50 mL) and distilled water (1 x 50 mL). The organic layer was dried over $MgSO_4$, and the solvent was concentrated *in vacuo*. The crude product was purified by column chromatography (3% methanol in EtOAc) to afford compound **11** as yellow solid in a yield of 68%. ^{1}H NMR (400 MHz, $CDCl_3$) δ 9.32 (s, 1H), 8.96 (d, *J* = 1.0 Hz, 1H), 8.27 (s, 1H), 8.04 (d, *J* = 1.0 Hz, 1H), 7.68 (d, *J* = 2.0 Hz, 1H), 7.53 – 7.46 (m, 3H), 7.28 (d, 1H), 7.10 – 7.04 (m, 3H), 6.89 (dd, *J* = 8.2, 7.0 Hz, 2H), 6.23 (t, *J* = 5.5 Hz, 1H), 4.90 (dd, *J* = 12.1, 5.4 Hz, 1H), 4.04 (s, 2H), 3.84 – 3.78 (m, 4H), 3.74 – 3.69 (m, 2H), 3.65 – 3.60 (m, 2H), 3.29 (dd, *J* = 12.7, 6.6 Hz, 2H), 2.92 – 2.67 (m, 3H), 2.41 (t, *J* = 7.4 Hz, 2H), 2.16 – 2.09 (m, 1H), 1.73 (dq, *J* = 15.1, 7.4 Hz, 4H), 1.49 (dt, *J* = 15.8, 7.9

Hz, 2H). ^{13}C NMR (101 MHz, $CDCl_3$) δ 171.66 (s), 171.12 (s), 169.67 (s), 168.53 (s), 167.69 (s), 162.30 (s), 162.17 (d, *J* = 245.4 Hz), 155.38 (s), 147.07 (s), 143.14 (s), 139.78 (s), 136.89 (d, *J* = 3.1 Hz), 136.32 (s), 133.47 (s), 132.61 (s), 131.98 (s), 128.20 (d, *J* = 7.9 Hz), 127.79 (s), 125.32 (s), 124.85 (s), 123.26 (s), 118.55 (s), 116.78 (s), 115.60 (d, *J* = 21.4 Hz), 111.63 (s), 110.03 (s), 49.01 (s), 45.02 (s), 44.51 (s), 44.09 (s), 42.46 (s), 40.96 (s), 33.11 (s), 31.54 (s), 29.15 (s), 26.77 (s), 24.85 (s), 22.96 (s). ^{19}F NMR (376 MHz, $CDCl_3$) δ -116.82 (s). HRMS (ESI) m/z: $[M+H]^+$ calculated for $C_{40}H_{41}N_9O_6F$: 762.3164, found 761.3162; $[M+Na]^+$ calculated for $C_{40}H_{40}N_9O_6FNa$: 784.2983, found 784.2987.

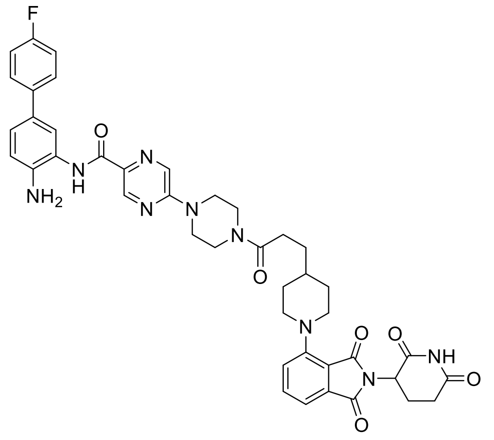

***N*-(4-amino-4'-fluoro-[1,1'-biphenyl]-3-yl)-5-(4-(3-(1-(2-(2,6-dioxopiperidin-3-yl)-1,3-dioxoisoindolin-4-yl)piperidin-4-yl)propanoyl)piperazin-1-yl)pyrazine-2-carboxamide, 12**: TFA (0.9 mmol, 20 eq) was added to a solution of compound **XXXIII** (0.046 mmol, 1.0 eq) in DCM (10 mL) and the reaction was stirred at room temperature overnight. Then, 50 mL of ethyl acetate was added and washed with saturated solution of sodium bicarbonate (2 x 50 mL) and distilled water (1 x 50 mL). The organic layer was dried over $MgSO_4$, and the solvent was concentrated *in vacuo*. The crude product was purified by column chromatography (3% methanol in EtOAc) to afford compound **12** as yellow solid in a yield of 75%. ^1H NMR (400 MHz, $CDCl_3$) δ 9.33 (s, 1H), 8.97 (d, *J* = 1.2 Hz, 1H), 8.14 (s, 1H), 8.05 (d, *J* = 1.3 Hz, 1H), 7.68 (d, *J* = 2.1 Hz, 1H), 7.56 (dd, *J* = 8.3, 7.2 Hz, 1H), 7.50 (dd, *J* = 8.8, 5.3 Hz, 2H), 7.36 (d, *J* = 7.1 Hz, 1H), 7.27 – 7.25 (m, 1H), 7.17 (d, *J* = 8.4 Hz, 1H), 7.07 (t, *J* = 8.7 Hz, 2H), 6.90 (d, *J* = 8.2 Hz, 1H), 4.96 (dd, *J* = 12.2, 5.4 Hz, 1H), 4.03 (s, 2H), 3.89 – 3.63 (m, 10H), 2.93 – 2.67 (m, 5H), 2.49 – 2.42 (m, 2H), 2.14 – 2.07 (m, 1H), 1.87 (d, *J* = 9.2 Hz, 2H), 1.71 (dd, *J* = 13.5, 7.0 Hz, 2H), 1.59 – 1.47 (m, 3H). ^{13}C NMR (101 MHz, $CDCl_3$) δ 171.88 (s), 171.04 (s), 168.34 (s), 167.52 (s), 166.87 (s), 162.29 (s), 162.17 (d, *J* = 245.5 Hz), 155.39 (s), 150.99 (s), 143.15 (s), 139.77 (s), 136.88 (d, *J* = 3.1 Hz), 135.65 (s), 134.25 (s), 133.49 (s), 132.00 (s), 128.20 (d, *J* = 7.9 Hz), 127.74 (s), 125.33 (s), 124.86 (s), 123.78 (s), 123.23 (s), 118.58 (s), 117.34 (s), 115.71 (s), 115.60 (d, *J*=21.5 Hz), 115.50 (s), 52.15 (s), 51.80 (s), 49.24 (s), 45.04 (s), 44.52 (s), 44.10 (s), 40.97 (s), 35.16 (s), 32.26 (s), 31.66 (s), 31.55 (s), 30.41 (s), 22.83 (s). ^{19}F NMR (376 MHz, $CDCl_3$) δ -116.80 (s). HRMS (ESI) m/z: $[M+H]^+$ calculated for $C_{42}H_{43}N_9O_6F$: 788.3320, found 788.3313, $[M+Na]^+$ calculated for $C_{42}H_{42}N_9O_6FNa$: 810.3140, found 810.3134.

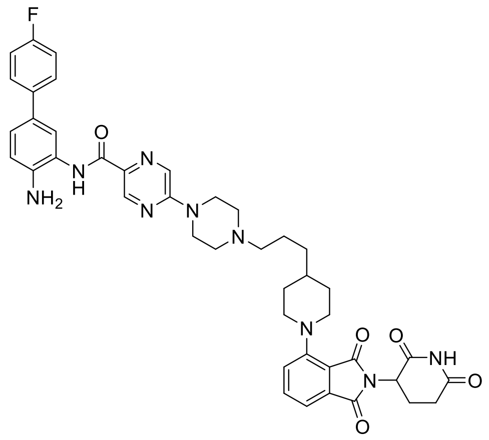

***N*-(4-amino-4'-fluoro-[1,1'-biphenyl]-3-yl)-5-(4-(3-(1-(2-(2,6-dioxopiperidin-3-yl)-1,3-dioxoisoindolin-4-yl)piperidin-4-yl)propyl)piperazin-1-yl)pyrazine-2-carboxamide, 13**: TFA (0.9 mmol, 20 eq) was added to a solution of compound **XXXV** (0.046 mmol, 1.0 eq) in DCM (10 mL) and the reaction was stirred at room temperature overnight. Then, 50 mL of ethyl acetate was added and washed with saturated solution of sodium bicarbonate (2 x 50 mL) and distilled water (1 x 50 mL). The organic layer was dried over $MgSO_4$, and the solvent was concentrated *in vacuo*. The crude product was purified by column chromatography (2% methanol in DCM) to afford compound **13** as yellow solid in a yield of 48%. ^{1}H NMR (400 MHz, $CDCl_3$) δ 9.32 (s, 1H), 8.94 (d, *J* = 1.3 Hz, 1H), 8.18 (s, 1H), 8.02 (d, *J* = 1.4 Hz, 1H), 7.67 (d, *J* = 2.1 Hz, 1H), 7.56 (dd, *J* = 8.4, 7.2 Hz, 1H), 7.50 (dd, *J* = 8.9, 5.3 Hz, 2H), 7.36 (d, *J* = 6.7 Hz, 1H), 7.26 (dd, *J*=2.1Hz, 1H), 7.17 (d, *J* = 8.2 Hz, 1H), 7.07 (t, *J* = 8.8 Hz, 2H), 6.89 (d, *J* = 8.2 Hz, 1H), 4.96 (dd, *J* = 12.2, 5.4 Hz, 1H), 4.05 (s, 2H), 3.80 – 3.69 (m, 4H), 2.92 – 2.66 (m, 5H), 2.60 – 2.55 (m, 4H), 2.44 – 2.38 (m, 2H), 2.16 – 2.07 (m, 1H), 1.85 (d, *J* = 9.2 Hz, 2H), 1.65 – 1.55 (m, 2H), 1.52 – 1.45 (m, 3H), 1.40 – 1.32 (m, 2H). ^{13}C NMR (101 MHz, $CDCl_3$) δ 171.06 (s), 168.35 (s), 167.56 (s), 166.86 (s), 162.54 (s), 162.16 (d, *J* = 245.3 Hz), 155.59 (s), 151.08 (s), 143.23 (s), 139.81 (s), 136.94 (d, *J* = 3.2 Hz), 135.60 (s), 134.27 (s), 132.63 (s), 131.95 (s), 128.20 (d, *J* = 7.9 Hz), 127.74 (s), 125.23 (s), 124.96 (s), 123.78 (s), 123.26 (s), 118.51 (s), 117.25 (s), 115.58 (d, *J* = 21.4 Hz), 115.37 (s), 58.93 (s), 52.87 (s), 52.29 (s), 51.96 (s), 49.24 (s), 44.48 (s), 35.59 (s), 34.39 (s), 32.52 (s), 32.50 (s), 31.56 (s), 24.10 (s), 22.84 (s). ^{19}F NMR (376 MHz, $CDCl_3$) δ -116.90 (s).

3.6. Synthesis of the PROTACs **7-9** and **14-15**

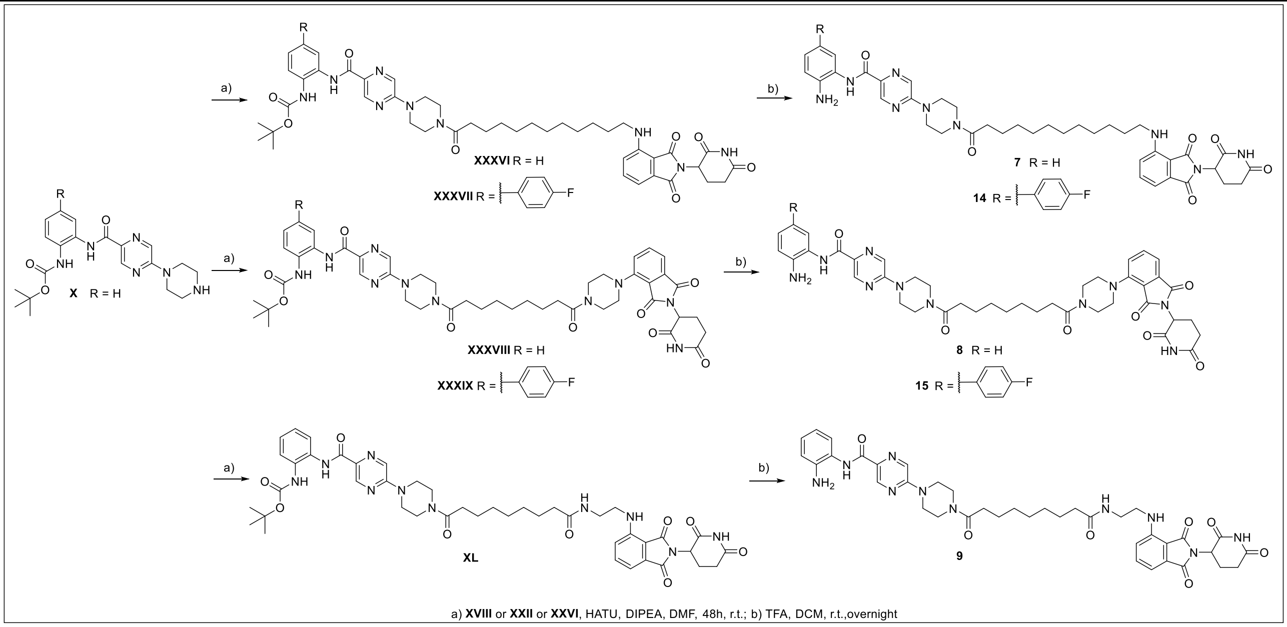

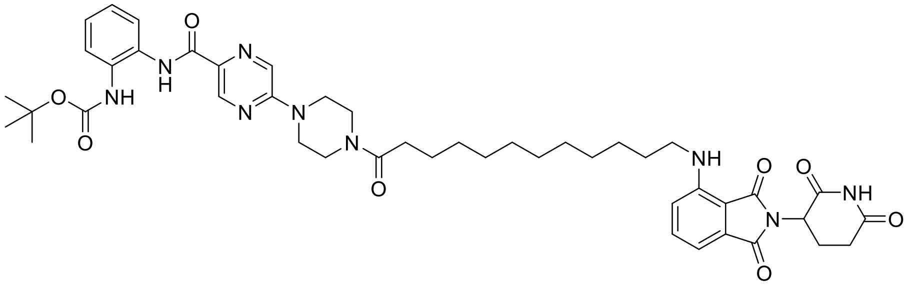

***tert*-butyl (2-(5-(4-(12-((2-(2,6-dioxopiperidin-3-yl)-1,3-dioxoisoindolin-4-yl)amino)dodecanoyl)piperazin-1-yl)pyrazine-2-carboxamido)phenyl)carbamate, XXXVI**: A solution of DIPEA (0.28 mmol, 1.5 eq) and HATU (0.25 mmol, 1.3 eq) were added to a solution of compound **XVIII** (0.21 mmol, 1.1 eq) in anhydrous DMF (10 mL) and stirred at room temperature for 15 minutes. Later, a solution of compound **X** (0.19 mmol, 1.0 eq) in anhydrous DMF (3 mL) was added dropwise and the reaction was stirred at room temperature for 48 hours. Then 100 mL of ethyl acetate was added to the reaction and washed with saturated solution of sodium bicarbonate (2 x 50 mL) and distilled water (1 x 50 mL). The crude product was purified by column chromatography (90% EtOAc in hexane) to afford compound **XXXVI** as yellow solid in a yield of 65%. ^{1}H NMR (400 MHz, $CDCl_3$) δ 9.62 (s, 1H), 8.96 (d, *J* = 1.3 Hz, 1H), 8.28 (s, 1H), 8.00 (d, *J* = 1.3 Hz, 1H), 7.71 – 7.63 (m, 1H), 7.57 (d, *J* = 5.6 Hz, 1H), 7.48 (dd, *J* = 8.4, 7.2 Hz, 1H), 7.24 – 7.16 (m, 2H), 7.07 (d, *J* = 6.8 Hz, 1H), 6.87 (d, *J* = 8.5 Hz, 1H), 6.22 (t, *J* = 5.4 Hz, 1H), 4.91 (dd, *J* = 12.1, 5.4 Hz, 1H), 3.85 – 3.77 (m, 4H), 3.73 – 3.60 (m, 4H), 3.25 (dd, *J* = 12.7, 6.9 Hz, 2H), 2.92 – 2.67 (m, 3H), 2.40 – 2.35 (m, 2H), 2.12 (ddd, *J* = 9.5, 5.6, 2.7 Hz, 1H), 1.72 – 1.61 (m, 5H), 1.50 (s, 9H), 1.40-1.27 (m, 14H). ^{13}C NMR (101 MHz, $CDCl_3$) δ 172.23 (s), 171.18 (s), 169.65 (s), 168.50 (s), 167.78 (s), 162.75 (s), 155.42 (s), 154.03 (s), 147.17 (s), 143.33 (s), 136.24 (s), 133.41 (s), 133.38 (s), 132.63 (s), 127.72 (s),

126.15 (s), 125.61 (s), 125.09 (s), 124.47 (s), 116.78 (s), 111.47 (s), 109.95 (s), 80.81 (s), 49.00 (s), 45.07 (s), 44.56 (s), 44.13 (s), 42.78 (s), 40.91 (s), 33.46 (s), 31.56 (s), 29.57 (s), 29.54 (s), 29.51 (s), 29.38 (s), 29.34 (s), 28.45 (s), 27.03 (s), 25.37 (s), 22.95 (s). HRMS (ESI) m/z: $[M+H]^+$ calculated for $C_{45}H_{58}N_9O_8$: 852.4408, found 852.4407; : $[M+Na]^+$ calculated for $C_{45}H_{57}N_9O_8Na$: 874.4228, found 874.4227.

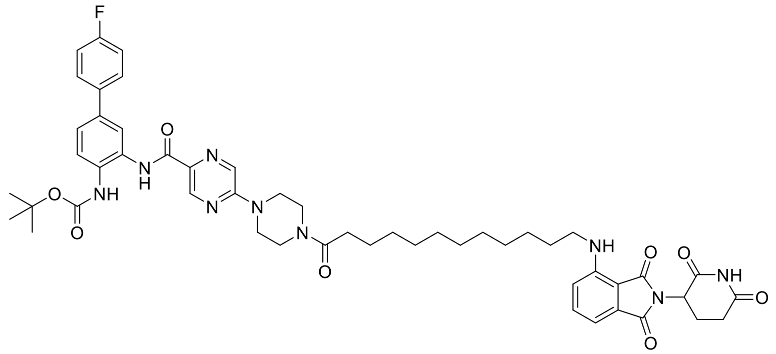

***tert*-butyl (3-(5-(4-(12-((2-(2,6-dioxopiperidin-3-yl)-1,3-dioxoisoindolin-4-yl)amino)dodecanoyl)piperazin-1-yl)pyrazine-2-carboxamido)-4'-fluoro-[1,1'-biphenyl]-4-yl)carbamate, XXXVII**: A solution of DIPEA (0.44 mmol, 2.8 eq) and HATU (0.22 mmol, 1.4 eq) were added to a solution of compound **XVIII** (0.19 mmol, 1.2 eq) in anhydrous DMF (10 mL) and stirred at room temperature for 15 minutes. Later, a solution of compound **VII** (0.15 mmol, 1.0 eq) in anhydrous DMF (3 mL) was added dropwise and the reaction was stirred at room temperature for 48 hours. Then 100 mL of ethyl acetate was added to the reaction and washed with saturated solution of sodium bicarbonate (2 x 50 mL) and distilled water (1 x 50 mL). The crude product was purified by column chromatography (90% EtOAc in hexane) to afford compound **XXXVII** as yellow solid in a yield of 50%. ^{1}H NMR (400 MHz, $CDCl_3$) δ 9.69 (s, 1H), 8.98 (d, J = 1.4 Hz, 1H), 8.19 (s, 1H), 8.02 (d, J = 1.4 Hz, 1H), 7.89 (s, 1H), 7.63 (d, J = 8.2 Hz, 1H), 7.55 (dd, J = 8.8, 5.3 Hz, 2H), 7.49 (dd, J = 8.4, 7.3 Hz, 1H), 7.38 (dd, J = 8.4, 2.1 Hz, 1H), 7.14 – 7.05 (m, 4H), 6.88 (d, J = 8.5 Hz, 1H), 6.22 (t, J = 5.4 Hz, 1H), 4.91 (dd, J = 12.1, 5.3 Hz, 1H), 3.88 – 3.77 (m, 4H), 3.76 – 3.62 (m, 4H), 3.26 (dd, J = 12.6, 6.7 Hz, 2H), 2.89 – 2.70 (m, 3H), 2.40 – 2.35 (m, 2H), 2.16 – 2.09 (m, 1H), 1.71 – 1.61 (m, 4H), 1.53 (s, 9H), 1.34 – 1.28 (m, 14H). ^{13}C NMR (101 MHz, $CDCl_3$) δ 172.23 (s), 171.10 (s), 169.66 (s), 168.45 (s), 167.77 (s), 162.87 (s), 162.64 (d, J = 246.5 Hz), 155.47 (s), 154.00 (s), 147.19 (s), 143.40 (s), 136.25 (s), 133.34 (s), 132.64 (s), 128.76 (d, J = 8.1 Hz), 127.72 (s), 125.87 (s), 124.22 (s), 123.22 (s), 116.79 (s), 115.75 (d, J = 21.4 Hz), 111.49 (s), 109.97 (s), 80.98 (s), 49.01 (s), 45.08 (s), 44.58 (s), 44.14 (s), 42.79 (s), 40.92 (s), 33.46 (s), 31.57 (s), 29.67 (s), 29.64 (s), 29.62 (s), 29.60 (s), 29.57 (s), 29.55 (s), 29.52 (s), 29.39 (s), 29.34 (s), 28.47 (s), 22.96 (s). HRMS (ESI) m/z: $[M+H]^+$ calculated for $C_{51}H_{61}N_9O_8F$: 946.4627, found 946.4660; $[M+Na]^+$ calculated for $C_{51}H_{60}N_9O_8FNa$: 968.4447, found 968.4481.

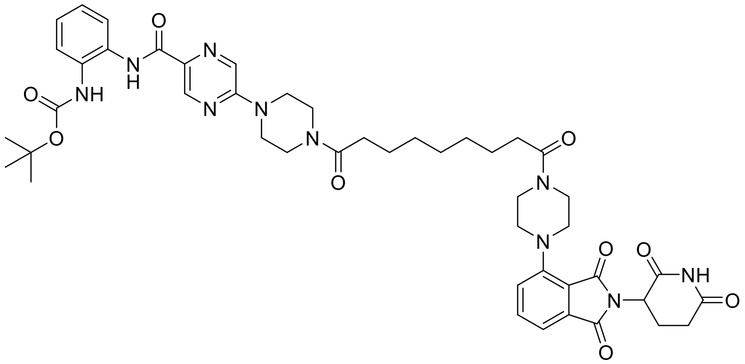

***tert*-butyl (2-(5-(4-(9-(4-(2-(2,6-dioxopiperidin-3-yl)-1,3-dioxoisoindolin-4-yl)piperazin-1-yl)-9-oxononanoyl)piperazin-1-yl)pyrazine-2-carboxamido)phenyl)carbamate, XXXVIII**: : A solution of DIPEA (0.32 mmol, 1.6 eq) and HATU (0.28 mmol, 1.4 eq) was added to a solution of compound **XXVI** (0.24 mmol, 1.2 eq) in anhydrous DMF (10 mL) and stirred at room temperature for 15 minutes. Later, a solution of compound **X** (0.2 mmol, 1.0 eq) in anhydrous DMF (3 mL) was added dropwise and the reaction was stirred at room temperature for 48 hours. Then 100 mL of ethyl acetate was added to the reaction and washed with saturated solution of sodium bicarbonate (2 x 50 mL) and distilled water (1 x 50 mL). The crude product was purified by column chromatography (60-80% EtOAc in hexane) to afford compound **XXXVIII** as yellow solid in a yield of 69%. ^{1}H NMR (400 MHz, $CDCl_3$) δ 9.62 (s, 1H), 8.96 (d, *J* = 1.4 Hz, 1H), 8.24 (s, 1H), 8.00 (d, *J* = 1.4 Hz, 1H), 7.67 (s, 1H), 7.62 (dd, *J* = 8.3, 7.2 Hz, 1H), 7.56 (d, *J* = 7.6 Hz, 1H), 7.44 (d, *J* = 7.1 Hz, 1H), 7.21 – 7.13 (m, 3H), 4.96 (dd, *J* = 12.3, 5.3 Hz, 1H), 3.91 – 3.61 (m, 12H), 3.41 – 3.25 (m, 4H), 2.91 – 2.71 (m, 3H), 2.37 (td, *J* = 7.7, 3.0 Hz, 4H), 2.16 – 2.09 (m, 1H), 1.66 (s, 4H), 1.50 (s, 9H), 1.37 (s, 6H). ^{13}C NMR (101 MHz, $CDCl_3$) δ 172.09 (s), 171.93 (s), 171.00 (s), 168.28 (s), 167.28 (s), 166.79 (s), 162.73 (s), 155.42 (s), 154.02 (s), 150.04 (s), 143.32 (s), 135.99 (s), 134.27 (s), 133.41 (s), 130.84 (s), 127.71 (s), 126.13 (s), 125.59 (s), 125.06 (s), 124.47 (s), 123.51 (s), 118.16 (s), 116.57 (s), 80.81 (s), 52.15 (s), 50.43 (s), 49.35 (s), 45.88 (s), 45.05 (s), 44.53 (s), 44.14 (s), 41.50 (s), 40.91 (s), 33.39 (s), 33.36 (s), 31.54 (s), 29.36 (s), 29.33 (s), 29.29 (s), 28.45 (s), 25.32 (s), 25.22 (s), 22.80 (s). HRMS (ESI) m/z: $[M+H]^+$ calculated for $C_{46}H_{57}N_{10}O_9$: 893.4310, found 893.4305, $[M+Na]^+$ calculated for $C_{46}H_{56}N_{10}O_9Na$: 915.4129, found 915.4128.

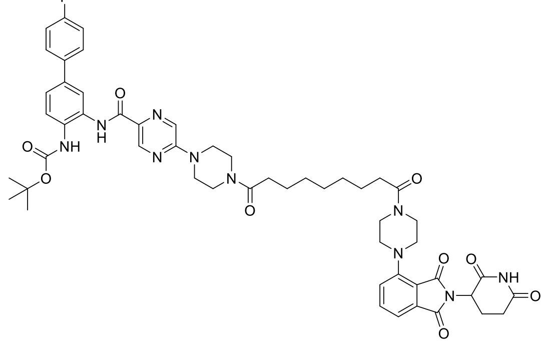

***tert*-butyl (3-(5-(4-(9-(4-(2-(2,6-dioxopiperidin-3-yl)-1,3-dioxoisoindolin-4-yl)piperazin-1-yl)-9-oxononanoyl)piperazin-1-yl)pyrazine-2-carboxamido)-4'-fluoro-[1,1'-biphenyl]-4-yl)carbamate, XXXIX:** A solution of DIPEA (0.52 mmol, 4.4 eq) and HATU (0.29 mmol, 2.2 eq) was added to a solution of compound **XXVI** (0.26 mmol, 2 eq) in anhydrous DMF (10 mL) and stirred at room temperature for 15 minutes. Later, a solution of compound **VII** (0.13 mmol, 1.0 eq) in anhydrous DMF (3 mL) was added dropwise and the reaction was stirred at room temperature for 48 hours. Then 100 mL of ethyl acetate was added to the reaction and washed with saturated solution of sodium bicarbonate (2 x 50 mL) and distilled water (1 x 50 mL). The crude product was purified by column chromatography (60-80% EtOAc in hexane) to afford compound **XXXIX** as yellow solid in a yield of 58%. ^{1}H NMR (400 MHz, $CDCl_3$) δ 9.69 (s, 1H), 8.97 (d, *J* = 1.3 Hz, 1H), 8.22 (s, 1H), 8.01 (d, *J* = 1.3 Hz, 1H), 7.90 (s, 1H), 7.65 – 7.59 (m, 2H), 7.54 (dd, *J* = 8.8, 5.3 Hz, 2H), 7.44 (d, *J* = 6.9 Hz, 1H), 7.37 (dd, *J* = 8.4, 2.1 Hz, 1H), 7.16 (d, *J* = 8.0 Hz, 1H), 7.10 (t, *J* = 8.7 Hz, 2H), 4.96 (dd, *J* = 12.3, 5.3 Hz, 1H), 3.92 – 3.61 (m, 12H), 3.42 – 3.25 (m, 4H), 2.93 – 2.71 (m, 3H), 2.42 – 2.32 (m, 4H), 2.16 – 2.09 (m, 1H), 1.72 – 1.62 (m, 4H), 1.52 (s, 9H), 1.37 (s, 6H). ^{13}C NMR (101 MHz, $CDCl_3$) δ 172.11 (s), 171.95 (s), 171.00 (s), 168.28 (s), 167.28 (s), 166.79 (s), 162.86 (s), 162.62 (d, *J* = 246.7 Hz), 155.45 (s), 154.02 (s), 150.04 (s), 143.37 (s), 137.70 (s), 136.41 (d, *J* = 3.2 Hz), 136.00 (s), 134.27 (s), 133.31 (s), 128.74 (d, *J* = 8.1 Hz), 127.73 (s), 125.39 (s), 124.60 (s), 123.51 (s), 122.86 (s), 118.16 (s), 116.58 (s), 115.74 (d, *J* = 21.5 Hz), 80.99 (s), 52.16 (s), 50.43 (s), 49.35 (s), 45.89 (s), 45.05 (s), 44.54 (s), 44.14 (s), 41.51 (s), 40.91 (s), 33.40 (s), 33.36 (s), 31.54 (s), 29.36 (s), 29.33 (s), 29.29 (s), 28.46 (s), 25.32 (s), 25.22 (s), 22.80 (s). HRMS (ESI) m/z: $[M+H]^+$ calculated for $C_{52}H_{60}N_{10}O_9F$: 987.4529, found 987.4548; $[M+Na]^+$ calculated for $C_{52}H_{59}N_{10}O_9FNa$: 1009.4348, found 1009.4363.

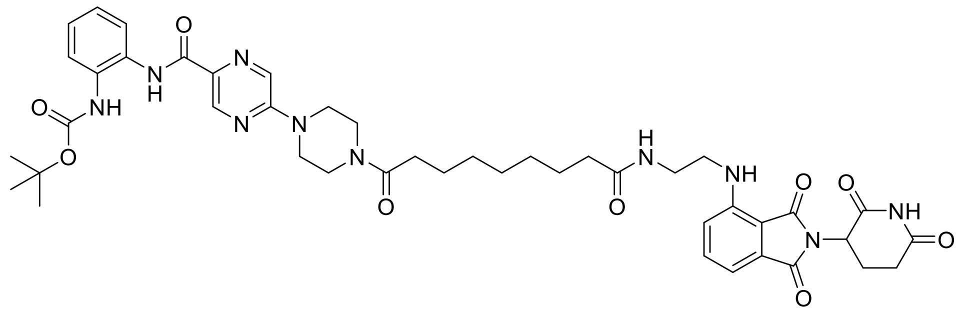

***tert*-butyl (2-(5-(4-(9-((2-((2-(2,6-dioxopiperidin-3-yl)-1,3-dioxoisoindolin-4-yl)amino)ethyl)amino)-9-oxononanoyl)piperazin-1-yl)pyrazine-2-carboxamido)phenyl)carbamate, XL**: A solution of DIPEA (0.4 mmol, 1.6 eq) and HATU (0.35 mmol, 1.4 eq) were added to a solution of compound **XXII** (0.3 mmol, 1.2 eq) in anhydrous DMF (10 mL) and stirred at room temperature for 15 minutes. Later, a solution of compound **X** (0.25 mmol, 1.0 eq) in anhydrous DMF (3 mL) was added dropwise and the reaction was stirred at room temperature for 48 hours. Then 100 mL of ethyl acetate was added to the reaction and washed with saturated solution of sodium bicarbonate (2 x 50 mL) and distilled water (1 x 50 mL). The crude product was purified by column chromatography (6% methanol in EtOAc) to afford compound **XL** as yellow solid in a yield of 24%. ^{1}H NMR (400 MHz, $CDCl_3$) δ 9.63 (s, 1H), 8.95

(d, J = 1.3 Hz, 1H), 8.62 (s, 1H), 8.00 (d, J = 1.3 Hz, 1H), 7.67 (s, 1H), 7.56 (d, J = 8.0 Hz, 1H), 7.50 (dd, J = 8.4, 7.3 Hz, 1H), 7.23 – 7.15 (m, 2H), 7.10 (d, J = 7.0 Hz, 1H), 6.99 (d, J = 8.5 Hz, 1H), 6.39 (t, J = 5.6 Hz, 1H), 6.08 (t, J = 4.9 Hz, 1H), 4.92 (dd, J = 12.1, 5.5 Hz, 1H), 3.84 – 3.76 (m, 4H), 3.74 – 3.60 (m, 4H), 3.50 – 3.43 (m, 4H), 2.91 – 2.68 (m, 3H), 2.39 – 2.33 (m, 2H), 2.19 – 2.14 (m, 2H), 2.14 – 2.08 (m, 1H), 1.65 – 1.58 (m, 4H), 1.50 (s, 9H), 1.38 – 1.22 (m, 6H). ^{13}C NMR (101 MHz, $CDCl_3$) δ 174.06 (s), 172.22 (s), 171.38 (s), 169.56 (s), 168.72 (s), 167.65 (s), 162.76 (s), 155.41 (s), 154.04 (s), 146.92 (s), 143.31 (s), 136.42 (s), 133.40 (s), 132.61 (s), 127.75 (s), 125.62 (s), 125.07 (s), 124.81 (s), 124.53 (s), 116.92 (s), 112.07 (s), 110.48 (s), 80.83 (s), 49.08 (s), 45.05 (s), 44.50 (s), 44.11 (s), 42.22 (s), 40.92 (s), 39.15 (s), 36.60 (s), 33.26 (s), 31.59 (s), 29.13 (s), 29.02 (s), 28.97 (s), 28.46 (s), 25.57 (s), 25.12 (s), 22.90 (s). HRMS (ESI) m/z: $[M+H]^+$ calculated for $C_{44}H_{55}N_{10}O_9$: 867.4153, found 867.4145.

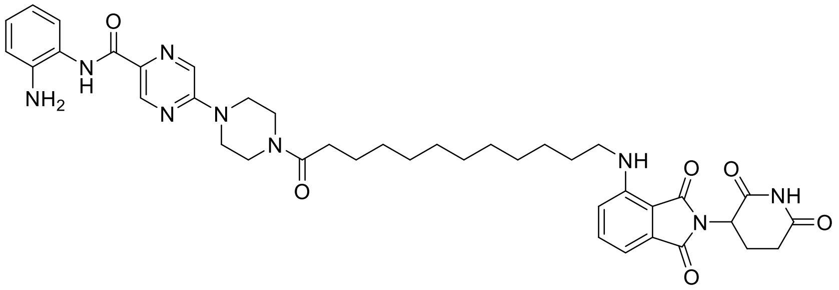

***N*-(2-aminophenyl)-5-(4-(12-((2-(2,6-dioxopiperidin-3-yl)-1,3-dioxoisoindolin-4-yl)amino)dodecanoyl)piperazin-1-yl)pyrazine-2-carboxamide, 7**: TFA (0.9 mmol, 20 eq) was added to a solution of compound **XXXVI** (0.046 mmol, 1.0 eq) in DCM (10 mL) and the reaction was stirred at room temperature overnight. Then, 50 mL of ethyl acetate was added and washed with saturated solution of sodium bicarbonate (2 x 50 mL) and distilled water (1 x 50mL). The organic layer was dried over $MgSO_4$, and the solvent was concentrated *in vacuo*. The crude product was purified by column chromatography (100% EtOAc) to afford compound **7** as yellow solid in a yield of 62%. ^{1}H NMR (400 MHz, $CDCl_3$) δ 9.27 (s, 1H), 8.95 (d, J = 1.1 Hz, 1H), 8.26 (s, 1H), 8.04 (d, J = 1.2 Hz, 1H), 7.48 (dd, J = 8.4, 7.2 Hz, 1H), 7.43 (dd, J = 8.2, 1.2 Hz, 1H), 7.10 – 7.04 (m, 2H), 6.90 – 6.82 (m, 3H), 6.22 (t, J = 5.4 Hz, 1H), 4.91 (dd, J = 12.1, 5.3 Hz, 1H), 3.86 – 3.77 (m, 4H), 3.74 – 3.58 (m, 4H), 3.25 (dd, J = 12.7, 6.8 Hz, 2H), 2.93 – 2.69 (m, 3H), 2.41 – 2.35 (m, 2H), 2.18 – 2.08 (m, 1H), 1.73 – 1.59 (m, 4H), 1.41 – 1.24 (m, 14H). ^{13}C NMR (101 MHz, $CDCl_3$) δ 172.24 (s), 171.16 (s), 169.65 (s), 168.50 (s), 167.78 (s), 162.20 (s), 155.38 (s), 147.18 (s), 143.11 (s), 140.53 (s), 136.25 (s), 133.54 (s), 132.63 (s), 127.75 (s), 126.93 (s), 124.84 (s), 124.53 (s), 119.77 (s), 118.27 (s), 116.79 (s), 111.48 (s), 109.95 (s), 49.01 (s), 45.09 (s), 44.55 (s), 44.13 (s), 42.78 (s), 40.93 (s), 33.46 (s), 31.56 (s), 29.84 (s), 29.57 (s), 29.55 (s), 29.51 (s), 29.39 (s), 29.34 (s), 27.03 (s), 25.38 (s), 22.95 (s). HRMS (ESI) m/z: $[M+H]^+$ calculated for $C_{40}H_{50}N_9O_6$: 752.3884, found 752.3878; : $[M+Na]^+$ calculated for $C_{40}H_{49}N_9O_6Na$: 774.3704, found 774.3691.

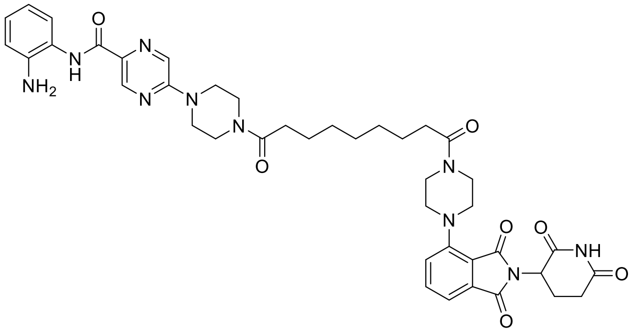

***N*-(2-aminophenyl)-5-(4-(9-(4-(2-(2,6-dioxopiperidin-3-yl)-1,3-dioxoisoindolin-4-yl)piperazin-1-yl)-9-oxononanoyl)piperazin-1-yl)pyrazine-2-carboxamide, 8:** TFA (0.9 mmol, 20 eq) was added to a solution of compound **XXXVIII** (0.046 mmol, 1.0 eq) in DCM (10 mL) and the reaction was stirred at room temperature overnight. Then, 50 mL of ethyl acetate was added and washed with saturated solution of sodium bicarbonate (2 x 50 mL) and distilled water (1 x 50 mL). The organic layer was dried over $MgSO_4$, and the solvent was concentrated *in vacuo*. The crude product was purified by column chromatography (5% methanol in DCM) to afford compound **8** as yellow solid in a yield of 35%. ^{1}H NMR (400 MHz, $CDCl_3$) δ 9.26 (s, 1H), 8.94 (d, *J* = 1.3 Hz, 1H), 8.26 (s, 1H), 8.04 (d, *J* = 1.4 Hz, 1H), 7.62 (dd, *J* = 8.3, 7.2 Hz, 1H), 7.46 – 7.41 (m, 2H), 7.16 (d, *J* = 8.0 Hz, 1H), 7.09 – 7.05 (m, 1H), 6.86 – 6.81 (m, 2H), 4.96 (dd, *J* = 12.3, 5.3 Hz, 1H), 3.98 (s, 2H), 3.92 – 3.62 (m, 12H), 3.42 – 3.24 (m, 4H), 2.93 – 2.69 (m, 3H), 2.41 – 2.33 (m, 4H), 2.15 – 2.08 (m, 1H), 1.67 (s, 4H), 1.37 (s, 6H). ^{13}C NMR (101 MHz, $CDCl_3$) δ 172.09 (s), 171.92 (s), 171.00 (s), 168.29 (s), 167.28 (s), 166.78 (s), 162.18 (s), 155.38 (s), 150.04 (s), 143.09 (s), 140.54 (s), 135.98 (s), 134.27 (s), 133.53 (s), 127.75 (s), 126.90 (s), 124.80 (s), 124.52 (s), 123.50 (s), 119.72 (s), 118.22 (s), 118.16 (s), 116.56 (s), 52.14 (s), 50.44 (s), 49.35 (s), 45.87 (s), 45.06 (s), 44.53 (s), 44.15 (s), 41.49 (s), 40.91 (s), 33.39 (s), 33.36 (s), 31.53 (s), 29.35 (s), 29.32 (s), 29.28 (s), 25.31 (s), 25.22 (s), 22.80 (s). HRMS (ESI) m/z: $[M+H]^+$ calculated for $C_{41}H_{49}N_{10}O_7$: 793.3786, found 793.3782; $[M+Na]^+$ calculated for $C_{41}H_{48}N_{10}O_7Na$: 815.3605, found 815.3600.

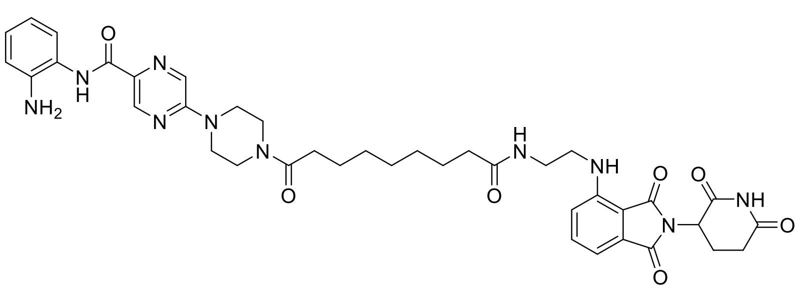

***N*-(2-aminophenyl)-5-(4-(9-((2-((2-(2,6-dioxopiperidin-3-yl)-1,3-dioxoisoindolin-4-yl)amino)ethyl)amino)-9-oxononanoyl)piperazin-1-yl)pyrazine-2-carboxamide, 9**: TFA (1.0 mmol, 20 eq) was added to a solution of compound **XL** (0.05 mmol, 1.0 eq) in DCM (10 mL) and the reaction was stirred at room temperature overnight. Then, 50 mL of ethyl acetate was added and washed with saturated solution of sodium bicarbonate (2 x 50 mL) and distilled water (1 x 50 mL). The organic layer was dried over $MgSO_4$, and the solvent was concentrated *in*

vacuo. The crude product was purified by column chromatography (8% methanol in DCM) to afford compound **9** as yellow solid in a yield of 67%. ^{1}H NMR (400 MHz, $CDCl_3$) δ 9.27 (s, 1H), 8.94 (d, *J* = 1.3 Hz, 1H), 8.53 (s, 1H), 8.03 (d, *J* = 1.3 Hz, 1H), 7.50 (dd, *J* = 8.4, 7.2 Hz, 1H), 7.43 (dd, *J* = 8.2, 1.4 Hz, 1H), 7.12 – 7.05 (m, 2H), 6.99 (d, *J* = 8.5 Hz, 1H), 6.88 – 6.82 (m, 2H), 6.40 (t, *J* = 5.1 Hz, 1H), 6.01 (t, *J* = 5.9 Hz, 1H), 4.92 (dd, *J* = 12.1, 5.4 Hz, 1H), 4.00 (s, 2H), 3.85 – 3.76 (m, 4H), 3.73 – 3.61 (m, 4H), 3.51 – 3.44 (m, 4H), 2.91 – 2.68 (m, 3H), 2.39 – 2.33 (m, 2H), 2.19 – 2.09 (m, 3H), 1.68 – 1.58 (m, 4H), 1.37 – 1.28 (m, 6H). ^{13}C NMR (101 MHz, $CDCl_3$) δ 174.04 (s), 172.21 (s), 171.29 (s), 169.56 (s), 168.65 (s), 167.65 (s), 162.21 (s), 155.38 (s), 146.92 (s), 143.10 (s), 140.57 (s), 136.44 (s), 133.55 (s), 132.63 (s), 127.78 (s), 126.94 (s), 124.85 (s), 124.51 (s), 119.75 (s), 118.24 (s), 116.92 (s), 112.10 (s), 110.52 (s), 49.08 (s), 45.06 (s), 44.50 (s), 44.11 (s), 42.23 (s), 40.93 (s), 39.17 (s), 36.62 (s), 33.27 (s), 31.60 (s), 29.14 (s), 29.02 (s), 28.97 (s), 25.58 (s), 25.13 (s), 22.91 (s). HRMS (ESI) m/z: $[M+H]^+$ calculated for $C_{39}H_{47}N_{10}O_7$: 767.3629, found 767.3632.

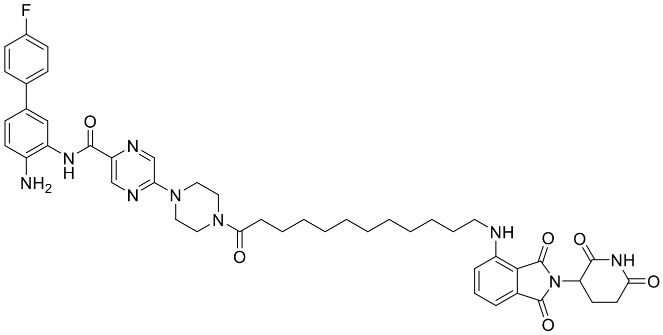

***N*-(4-amino-4'-fluoro-[1,1'-biphenyl]-3-yl)-5-(4-(12-((2-(2,6-dioxopiperidin-3-yl)-1,3-dioxoisoindolin-4-yl)amino)dodecanoyl)piperazin-1-yl)pyrazine-2-carboxamide, 14**: TFA (0.9 mmol, 20 eq) was added to a solution of compound **XXXVII** (0.046 mmol, 1.0 eq) in DCM (10 mL) and the reaction was stirred at room temperature overnight. Then, 50 mL of ethyl acetate was added and washed with saturated solution of sodium bicarbonate (2 x 50 mL) and distilled water (1 x 50 mL). The organic layer was dried over $MgSO_4$, and the solvent was concentrated *in vacuo*. The crude product was purified by column chromatography (2% methanol in DCM) to afford compound **14** as yellow solid in a yield of 59%. ^{1}H NMR (400 MHz, $CDCl_3$) δ 9.33 (s, 1H), 8.96 (d, *J* = 1.3 Hz, 1H), 8.25 (s, 1H), 8.04 (d, *J* = 1.4 Hz, 1H), 7.68 (d, *J* = 2.1 Hz, 1H), 7.54 – 7.45 (m, 3H), 7.28 (d, *J* = 2.1 Hz, 1H), 7.10 – 7.04 (m, 3H), 6.89 (t, 2H), 6.22 (t, *J* = 5.5 Hz, 1H), 4.90 (dd, *J* = 12.1, 5.3 Hz, 1H), 4.03 (s, 2H), 3.86 – 3.78 (m, 4H), 3.76 – 3.61 (m, 4H), 3.25 (dd, *J* = 12.7, 6.9 Hz, 2H), 2.92 – 2.69 (m, 3H), 2.41 – 2.34 (m, 2H), 2.15 – 2.09 (m, 1H), 1.73 – 1.63 (m, 4H), 1.43 – 1.27 (m, 14H). ^{13}C NMR (101 MHz, $CDCl_3$) δ 172.22 (s), 171.13 (s), 169.65 (s), 168.48 (s), 167.77 (s), 162.31 (s), 162.17 (d, *J* = 245.3 Hz), 155.42 (s), 147.17 (s), 143.17 (s), 139.79 (s), 136.89 (d, *J* = 3.1 Hz), 136.24 (s), 133.43 (s), 132.63 (s), 131.99 (s), 128.20 (d, *J* = 7.9 Hz), 127.75 (s), 125.33 (s), 124.85 (s), 123.27 (s), 118.56 (s), 116.78 (s), 115.60 (d, *J* = 21.4 Hz), 111.48 (s), 109.96 (s), 49.00 (s), 45.08 (s), 44.55 (s), 44.14 (s), 42.78 (s), 40.91 (s), 33.46 (s), 31.56 (s), 29.56 (s), 29.54 (s), 29.51 (s), 29.39 (s), 29.34 (s), 25.37 (s), 22.95 (s). ^{19}F NMR (376 MHz, $CDCl_3$) δ -116.83 (s). HRMS (ESI) m/z:

$[M+H]^+$ calculated for $C_{46}H_{53}N_9O_6F$: 846.4103, found 846.4122; $[M+Na]^+$ calculated for $C_{46}H_{52}N_9O_6FNa$: 868.3922, found 868.3941.

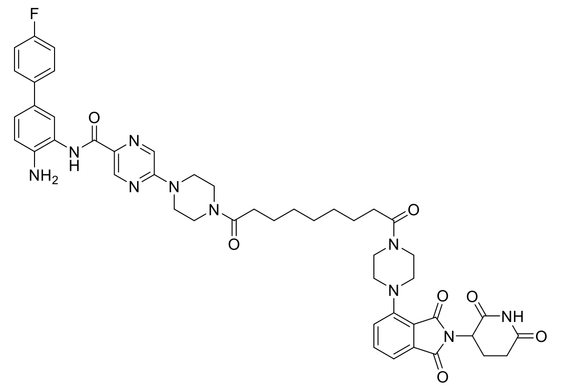

***N*-(4-amino-4'-fluoro-[1,1'-biphenyl]-3-yl)-5-(4-(9-(4-(2-(2,6-dioxopiperidin-3-yl)-1,3-dioxoisoindolin-4-yl)piperazin-1-yl)-9-oxononanoyl)piperazin-1-yl)pyrazine-2-carboxamide, 15**: TFA (1.0 mmol, 20 eq) was added to a solution of compound **XXXIX** (0.05 mmol, 1.0 eq) in DCM (10 mL) and the reaction was stirred at room temperature overnight. Then, 50 mL of ethyl acetate was added and washed with saturated solution of sodium bicarbonate (2 x 50 mL) and distilled water (1 x 50 mL). The organic layer was dried over $MgSO_4$, and the solvent was concentrated *in vacuo*. The crude product was purified by column chromatography (2% methanol in DCM) to afford compound **15** as yellow solid in a yield of 61%. ^{1}H NMR (400 MHz, $CDCl_3$) δ 9.32 (s, 1H), 8.96 (d, J = 1.3 Hz, 1H), 8.21 (s, 1H), 8.05 (d, J = 1.3 Hz, 1H), 7.68 (d, J = 2.1 Hz, 1H), 7.62 (dd, J = 8.3, 7.3 Hz, 1H), 7.50 (dd, J = 8.9, 5.3 Hz, 2H), 7.44 (dd, J = 7.2, 0.5 Hz, 1H), 7.28 (d, J = 2.1 Hz, 1H), 7.16 (d, J = 7.9 Hz, 1H), 7.07 (t, J = 8.8 Hz, 2H), 6.89 (d, J = 8.2 Hz, 1H), 4.96 (dd, J = 12.3, 5.3 Hz, 1H), 4.06 (s, 2H), 3.92 – 3.62 (m, 12H), 3.43 – 3.22 (m, 4H), 2.94 – 2.69 (m, 3H), 2.41 – 2.34 (m, 4H), 2.16 – 2.09 (m, 1H), 1.70 – 1.61 (m, 4H), 1.38 (s, 6H). ^{13}C NMR (101 MHz, $CDCl_3$) δ 172.10 (s), 171.93 (s), 170.96 (s), 168.27 (s), 167.28 (s), 166.79 (s), 162.32 (s), 162.17 (d, J = 245.5 Hz), 155.42 (s), 150.05 (s), 143.15 (s), 139.79 (s), 136.88 (d, J = 3.1 Hz), 135.99 (s), 134.28 (s), 133.42 (s), 131.98 (s), 128.20 (d, J = 7.9 Hz), 127.76 (s), 125.33 (s), 124.85 (s), 123.51 (s), 123.25 (s), 118.56 (s), 118.16 (s), 116.57 (s), 115.60 (d, J = 21.4 Hz), 52.16 (s), 50.43 (s), 49.35 (s), 45.88 (s), 45.07 (s), 44.54 (s), 44.14 (s), 41.50 (s), 40.92 (s), 33.40 (s), 33.36 (s), 31.54 (s), 29.36 (s), 29.32 (s), 29.29 (s), 25.32 (s), 25.23 (s), 22.80 (s). ^{19}F NMR (376 MHz, $CDCl_3$) δ -116.82 (s). HRMS (ESI) m/z: $[M+H]^+$ calculated for $C_{47}H_{52}N_{10}O_7F$: 887.4004, found 887.4005; $[M+Na]^+$ calculated for $C_{47}H_{51}N_{10}O_7FNa$: 909.3824, found 909.3821.

3.7. Synthesis of **5-Me** and **7-Me**

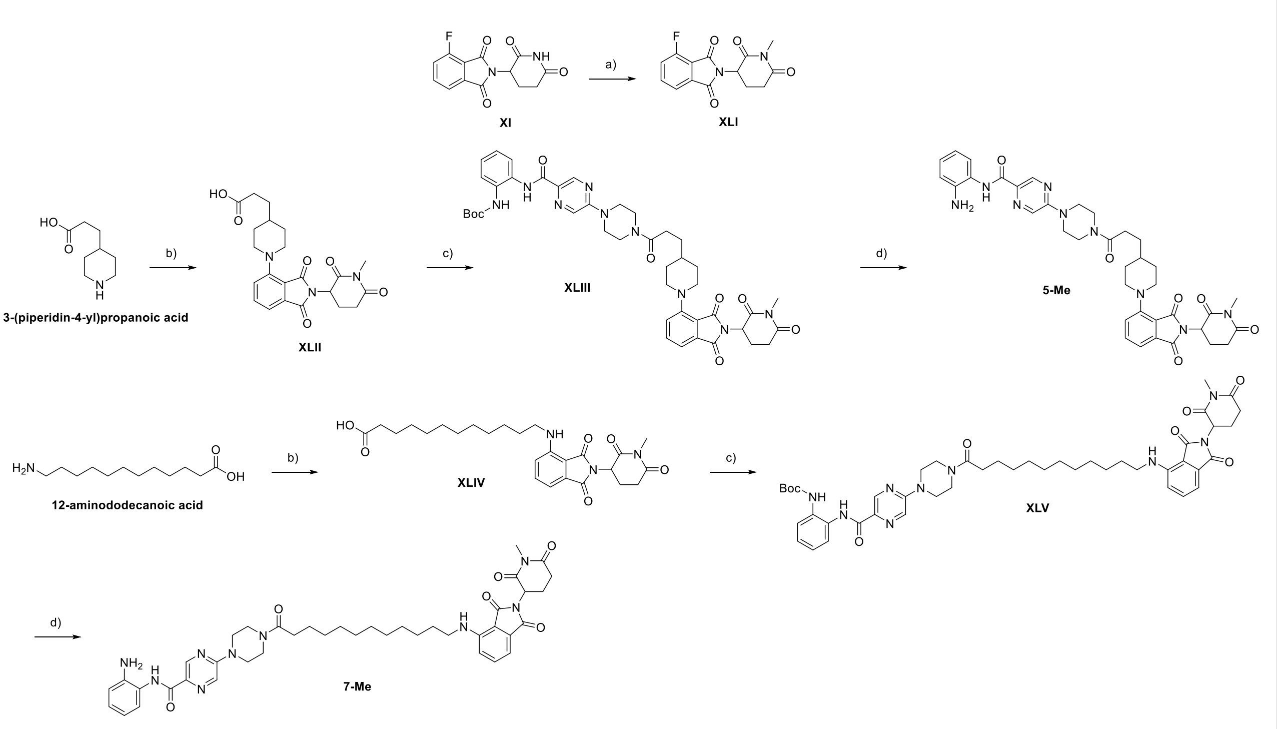

a) MeI, Cs_2CO_3, DMF, r.t. 16h; b) **XLI**, DIPEA, DMSO,100°C, 16h; c) **x**, HATU, DIPEA, DMF, r.t., 16h; d) TFA, DCM, r.t., 4h

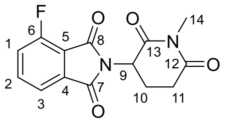

4-fluoro-2-(1-methyl-2,6-dioxopiperidin-3-yl)isoindoline-1,3-dione, XLI: A mixture of **XI** (0.500 g, 1.81 mmol), and Cs_2CO_3 (0.885 g, 2.72 mmol) in DMF (10 mL) was stirred at room temperature for 10 minutes, then methyl iodide (0.135 mL, 2.17 mmol) added and the reaction mixture stirred at room temperature for 16 hours. Acetic acid (1 mL) was added, then the reaction mixture concentrated *in vacuo*. The crude residue was triturated in water, filtered and then dried under vacuum to afford **XLI** (0.433 g, 1.49 mmol, 82% yield) as an off-white solid. ^1H NMR (400 MHz, DMSO-d_6) δ ppm 7.96 (ddd, J=8.4, 7.4, 4.6 Hz, 1 H, 2-CH), 7.79 (d, J=7.3 Hz, 1 H, 3-CH), 7.74 (t, $J_{CH/CF}$ =8.8 Hz, 1 H, 1-CH), 5.22 (dd, J=13.1, 5.4 Hz, 1 H, 9-CH), 3.02 (s, 3 H, 14-CH_3), 2.91 - 3.00 (m, 1 H, 11-CH), 2.73 - 2.82 (m, 1 H, 11-CH), 2.51 - 2.60 (m, 1 H, 10-CH), 2.08 (dtd, J=12.8, 5.4, 2.5 Hz, 1 H, 10-CH). ^{13}C NMR (101 MHz, DMSO-d_6) δ 171.7 (C12), 169.4 (C13), 166.1 (d, J=2.9 Hz, C8), 163.9 (d, J=0.8 Hz, C7), 156.8 (d, J=262.5 Hz, C6), 138.1 (d, J=8.0 Hz, C2), 133.4 (d, J=1.5 Hz, C4), 123.0 (d, J=19.6 Hz, C1), 120.0 (d, J=3.2 Hz, C3), 117.0 (d, J=12.4 Hz, C5), 49.6 (C9), 31.0 (C11), 26.6 (C14), 21.0 (C10). MS (ESI) m/z: 291 $[M+H]^+$. Spectroscopic data consistent with previous literature.[3]

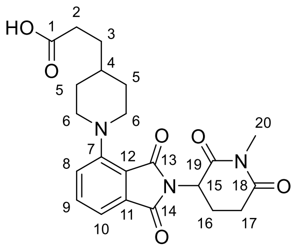

3-(1-(2-(1-methyl-2,6-dioxopiperidin-3-yl)-1,3-dioxoisoindolin-4-yl)piperidin-4-yl)propanoic acid, XLII: To a solution of **XLI** (120.0 mg, 0.413 mmol) in dry DMSO (7 mL), 3-(piperidin-4-yl)propanoic acid (72.8 mg, 0.376 mmol) and DIPEA (0.196 mL, 1.13 mmol) were added and the resultant solution stirred 100 °C under a N_2 atmosphere for 16 hours. The reaction mixture was cooled to room temperature, then water (30 mL) added and the product extracted with EtOAc (2 x 50 mL). The organic layers were combined, washed with saturated brine, dried over Na_2SO_4, then concentrated *in vacuo* to afford a green/brown tar. The crude product was purified by column chromatography (1-9% MeOH in DCM) to afford **XLII** (89.6 mg, 0.199 mmol, 53% yield) as a yellow solid. ^1H NMR (400 MHz, $CDCl_3$) δ ppm 7.56 (dd, *J*=8.3, 7.1 Hz, 1 H, 9-CH), 7.35 (d, *J*=7.1 Hz, 1 H, 8-CH), 7.16 (d, *J*=8.3 Hz, 1 H, 10-CH), 4.94 - 4.99 (m, 1 H, 15-CH), 3.74 (t, *J*=8.4 Hz, 2 H, 6-CH), 3.20 (s, 3 H, 20-CH_3), 2.93 - 2.99 (m, 1 H, 17-CH), 2.82 - 2.91 (m, 2 H, 6-CH), 2.71 - 2.81 (m, 2 H, (16,17)-CH), 2.43 (t, *J*=7.7 Hz, 2 H, 2-CH_3), 2.04 - 2.10 (m, 1 H, 16-CH), 1.78 - 1.89 (m, 2 H, 5-CH), 1.63 - 1.72 (m, 2 H, 3-CH_2), 1.42 - 1.54 (m, 3 H, 4-CH,5-CH). ^{13}C NMR (101 MHz, $CDCl_3$) δ ppm 179.1 (C1), 171.3 (C18), 168.9 (C19), 167.6 (C14), 166.8 (C13), 150.7 (C7), 135.4 (C9), 134.1 (C11), 123.5 (C10), 117.2 (C12), 115.2 (C8), 51.9 (C6), 51.6 (C6*), 49.8 (C15), 34.8 (C4), 31.95 (C5), 31.9 (C5*), 31.85 (C17), 31.2 (C2), 31.1 (C3), 27.2 (C20), 21.9 (C16). HRMS (ESI) m/z: $[M+H]^+$ calculated for $C_{22}H_{26}N_3O_6$: 428.1822, found 428.1818.

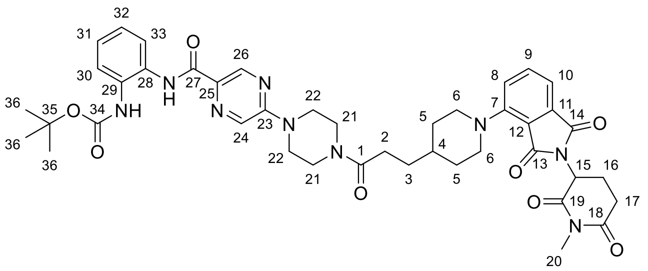

tert-butyl (2-(5-(4-(3-(1-(2-(1-methyl-2,6-dioxopiperidin-3-yl)-1,3-dioxoisoindolin-4-yl)piperidin-4-yl)propanoyl)piperazin-1-yl)pyrazine-2-carboxamido)phenyl)carbamate, XLIII: To a solution of **XLII** (17.3 mg, 0.041 mmol) in dry DMF (1 mL) at 0 °C, DIPEA (19.3 µL, 0.111 mmol) and HATU (18.2 mg, 0.048 mmol) were added. The reaction mixture was stirred for 15 minutes, after which a solution of **X** (14.7 mg, 0.037 mmol) in DMF (1 mL) was added slowly and the resultant solution stirred at room temperature for 16 hours.

The reaction mixture was diluted in EtOAc (10 mL), then washed with saturated $NaHCO_3$ (2 x 5 mL) and brine (2 x 5 mL). The organic layer was dried over $MgSO_4$, filtered and concentrated *in vacuo* to afford a yellow tar (55 mg). The crude product was purified by column chromatography (0-100% EtOAC in hexane) to afford **XLIII**

(19.9 mg, 0.025 mmol, 67% yield) as a yellow solid. ^{1}H NMR (400 MHz, $CDCl_3$) δ ppm 9.64 (s, 1 H, 28-NH), 8.97 (d, *J*=1.2 Hz, 1 H, 26-CH), 8.01 (d, *J*=1.3 Hz, 1 H, 24-CH), 7.61 - 7.75 (m, 1 H, 30/33-CH), 7.52 - 7.61 (m, 2 H, 30/33-CH,9-CH), 7.37 (d, *J*=6.9 Hz, 1 H, 8-CH), 7.09 - 7.24 (m, 4 H, 10,31,32-CH,29-NH), 4.97 (dd, *J*=12.5, 5.2 Hz, 1 H, 15-CH), 3.78 - 3.87 (m, 4 H, 21/22-CH), 3.69 - 3.78 (m, 4 H, 6,21/22-CH), 3.61 - 3.68 (m, 2 H, 21/22-CH), 3.21 (s, 3 H, 20-CH_3), 2.94 - 3.03 (m, 1 H, 17-CH), 2.85 - 2.94 (m, 2 H, 6-CH), 2.72 - 2.84 (m, 2 H, 16-CH, 17-CH), 2.46 (t, *J*=7.4 Hz, 2 H, 2-CH_2), 2.05 - 2.12 (m, 1 H, 16-CH), 1.83 - 1.93 (m, 2 H, 5-CH), 1.68 - 1.76 (m, 2 H, 3-CH_2), 1.46 - 1.60 (m, 12 H, 36-CH_3,(4,5)-CH). ^{13}C NMR (101 MHz, $CDCl_3$) δ ppm 171.7 (C1), 171.2 (C18), 168.9 (C14), 167.5 (C19), 166.9 (C13), 162.5 (C27), 155.2 (C23), 153.8 (C34), 150.8 (C7), 143.1 (C26), 135.4 (C9), 134.1 (C25), 133.3 (C11), 130.7 (C28/29), 130.1 (C28/29), 127.5 (C24), 126.0 (C31/32), 125.4 (C31/32), 124.9 (C30/33), 124.3 (C30/33), 123.5 (C10), 117.3 (C12), 115.3 (C8), 80.7 (C35), 51.9 (C6), 51.7 (C6), 49.9 (C15), 44.9 (C21/22), 44.4 (C21/22), 43.9 (C21/22), 40.8 (C21/22), 35.0 (C4), 32.15 (C5), 32.1 (C5), 31.9 (C17), 31.5 (C3), 30.3 (C2), 28.3 (C36), 27.2 (C20), 22.0 (C16). HRMS (ESI) m/z: $[M+H]^+$ calculated for $C_{42}H_{50}N_9O_8$: 808.3782, found 808.3770.

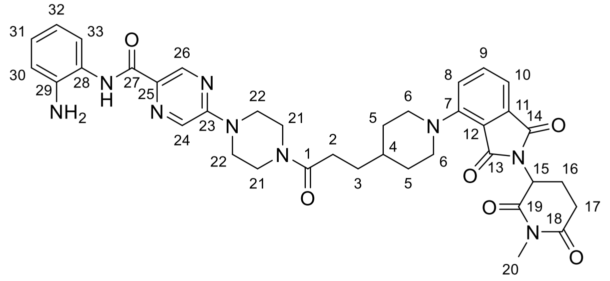

N-(2-aminophenyl)-5-(4-(3-(1-(2-(1-methyl-2,6-dioxopiperidin-3-yl)-1,3-dioxoisoindolin-4-yl)piperidin-4-yl)propanoyl)piperazin-1-yl)pyrazine-2-carboxamide, 5-Me: TFA (0.5 mL) was added to a stirring solution of **XLIII** (19.9 mg, 0.025 mmol) in DCM (2 mL) and the resulting reaction mixture stirred at room temperature for 4 hours. The reaction mixture was concentrated *in vacuo* (60.9 mg crude), then redissolved in EtOAc (25 mL) and washed with sat. $NaHCO_3$ (2 x 25mL) and water (1 x 25 mL). The organic layer was dried over Na_2SO_4, filtered and concentrated *in vacuo* to afford **5-Me** (17.4 mg, 0.025 mmol, 100% yield) as a yellow solid. The product was further purified by column chromatography (Biotage, 0-9% MeOH in DCM) and lyophilised to afford **5-Me** (16.1 mg) as a fluffy yellow solid for biological evaluation. ^{1}H NMR (400 MHz, $CDCl_3$) δ ppm 9.30 (s, 1 H, 28-NH), 8.95 (s, 1 H, 26-CH), 8.04 (s, 1 H, 24-CH), 7.57 (dd, *J*=8.3, 7.1 Hz, 1 H, 9-CH), 7.43 (d, *J*=7.9 Hz, 1 H, 33-CH), 7.37 (d, *J*=7.1 Hz, 1 H, 8-CH), 7.17 (d, *J*=8.3 Hz, 1 H, 10-CH), 7.05 - 7.12 (m, 1 H, 31-CH), 6.83 - 6.92 (m, 2 H, (30,32)-CH), 4.92 - 5.00 (m, 1 H, 15-CH), 3.78 - 3.88 (m, 4 H, 21/22-CH), 3.69 - 3.78 (m, 4 H, 6,21/22-CH), 3.62 - 3.68 (m, 2 H, 21/22-CH), 3.52 (br s, 2 H, 29-NH_2), 3.21 (s, 3 H, 20-CH_3), 2.94 - 3.04 (m, 1 H, 17-CH), 2.85 - 2.93 (m, 2 H, 6-CH), 2.70 - 2.83 (m, 2 H, (16,17)-CH), 2.46 (t, *J*=7.7 Hz, 2 H, 2-CH2), 2.05 - 2.11 (m, 1 H, 16-CH), 1.83 - 1.92 (m, 2 H, 5-CH), 1.67 - 1.75 (m, 2 H, 3-CH_2), 1.49 - 1.60 (m, 3 H, (4,5)-CH). ^{13}C NMR (101

MHz, $CDCl_3$) δ 171.7 (C1), 171.2 (C18), 168.9 (C14), 167.5 (C19), 166.9 (C13), 162.1 (C27), 155.2 (C23), 150.8 (C7), 142.9 (C26), 139.6 (C29), 135.4 (C9), 134.1 (C25), 133.3 (C11), 127.6 (C24), 126.8 (C31), 124.6 (C28), 124.6 (C33), 123.5 (C10), 120.1 (C30/32), 118.4 (C30/32), 117.3 (C12), 115.3 (C8), 51.9 (C6), 51.7 (C6), 49.9 (C15), 44.9 (C21/22), 44.3 (C21/22), 43.9 (C21/22), 40.8 (C21/22), 35.0 (C4), 32.1 (C5), 31.9 (C17), 31.5 (C3), 30.3 (C2), 27.2 (C20), 22.0 (C16). HRMS (ESI) m/z: [M+H]+ calculated for $C_{37}H_{42}N_9O_6$: 708.3258, found 708.3254.

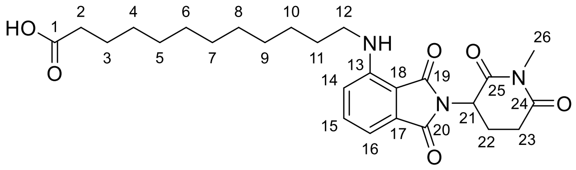

12-((2-(1-methyl-2,6-dioxopiperidin-3-yl)-1,3-dioxoisoindolin-4-yl)amino)dodecanoic acid, XLIV: To a solution of **XLI** (120.0 mg, 0.413 mmol) in dry DMSO (7 mL), 12-aminododecanoic acid (106.8 mg, 0.496 mmol) and DIPEA (0.216 mL, 1.24 mmol) were added and the resultant solution stirred 100 °C under a N_2 atmosphere for 16 hours. The reaction mixture was cooled to room temperature, then water (30 mL) added and the product extracted with EtOAc (2 x 50 mL). The organic layers were combined, washed with saturated brine, dried over Na_2SO_4, then concentrated *in vacuo* to afford a green/brown tar. The crude product was purified by column chromatography (0-100% EtOAc in hexane) to afford **XLIV** (76.0 mg, 0.157 mmol, 38% yield) as a green tar. ^{1}H NMR (400 MHz, DMSO-d_6) δ ppm 11.94 (s, 1 H, 1-CO_2H), 7.58 (dd, *J*=8.4, 7.2 Hz, 1 H, 15-CH), 7.09 (d, *J*=8.4 Hz, 1 H, 14-CH), 7.02 (d, *J*=7.2 Hz, 1 H, 16-CH), 6.52 (t, *J*=6.3 Hz, 1 H, 13-NH), 5.12 (dd, *J*=13.0, 5.4 Hz, 1 H, 21-CH), 3.28 (q, *J*=6.3 Hz, 2 H, 12-CH_2), 3.01 (s, 3 H, 26-CH_3), 2.94 (ddd, *J*=17.4, 13.8, 5.3 Hz, 1 H, 23-CH), 2.71 - 2.79 (m, 1 H, 23-CH), 2.51 - 2.59 (m, 1 H, 22-CH), 2.17 (t, *J*=7.3 Hz, 2 H, 2-CH_2), 2.00 - 2.08 (m, 1 H, 22-CH), 1.56 (quin, *J*=7.0 Hz, 2 H, 11-CH_2), 1.47 (quin, *J*=7.2 Hz, 2 H, 3-CH_2), 1.22 - 1.33 (m, 14 H, (4-10)-CH_2). ^{13}C NMR (101 MHz, DMSO-d_6) δ ppm 174.5 (C1), 171.8 (C24), 169.8 (C25), 168.9 (C19), 167.3 (C20), 146.4 (C13), 136.3 (C15), 132.2 (C17), 117.2 (C14), 110.4 (C18), 108.9 (C16), 49.1 (C21), 41.8 (C12), 33.6 (C2), 31.1 (C23), 28.95 (alkyl CH_2), 28.9 (alkyl CH_2), 28.85 (alkyl CH_2), 28.7 (2 x alkyl CH_2), 28.6 (alkyl CH_2), 28.5 (alkyl CH_2), 26.6 (C26), 26.3 (C10), 24.5 (C3), 21.4 (C22). HRMS (ESI) m/z: $[M+H]^+$ calculated for $C_{26}H_{36}N_3O_6$: 486.2604, found 486.2604.

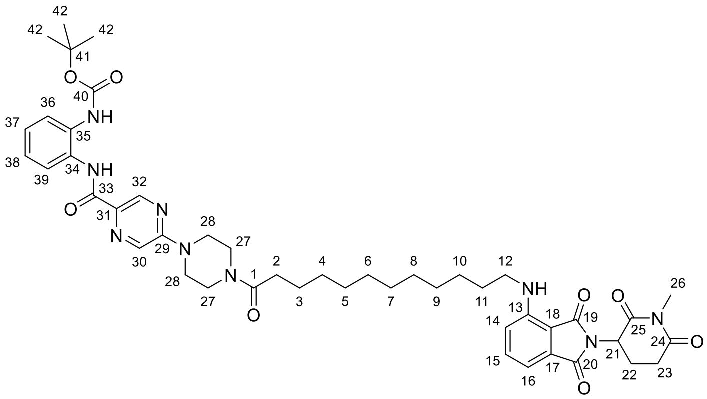

tert-butyl (2-(5-(4-(12-((2-(1-methyl-2,6-dioxopiperidin-3-yl)-1,3-dioxoisoindolin-4-yl)amino)dodecanoyl)piperazin-1-yl)pyrazine-2-carboxamido)phenyl)carbamate, XLV: To a solution of **XLIV** (21.2 mg, 0.044 mmol) in dry DMF (1 mL) at 0 °C, DIPEA (20.7 µL, 0.115 mmol) and HATU (19.6 mg, 0.052 mmol) were added. The reaction mixture was stirred for 15 minutes, after which a solution of **X** (15.8 mg, 0.040 mmol) in DMF (1 mL) was added slowly and the resultant solution stirred at room temperature for 16 hours. The reaction mixture was diluted in EtOAc (10 mL), then washed with saturated $NaHCO_3$ (2 x 5 mL) and brine (2 x 5 mL). The organic layer was dried over $MgSO_4$, filtered and concentrated *in vacuo* to afford a yellow tar. The crude product was purified by column chromatography (1-9% MeOH in DCM) to afford **XLV** (35.8 mg, 0.039 mmol, 98% yield) as a yellow solid. ^{1}H NMR (400 MHz, $CDCl_3$) δ_H ppm 9.63 (s, 1 H, 34-NH), 8.96 (d, *J*=1.4 Hz, 1 H, 32-CH), 8.02 (s, 1 H, 35-NH), 8.00 (d, *J*=1.4 Hz, 1 H, 30-CH), 7.67 (br d, *J*=7.2 Hz, 1 H, 36/39-CH), 7.57 (br d, *J*=7.2 Hz, 1 H, 36/39-CH), 7.49 (dd, *J*=8.3, 7.3 Hz, 1 H, 15-CH), 7.17 - 7.23 (m, 2 H, (37,38)-CH), 7.08 (d, *J*=7.2 Hz, 1 H, 14-CH), 6.88 (d, *J*=8.5 Hz, 1 H, 16-CH), 6.22 (br t, *J*=6.3 Hz, 1 H), 4.89 - 4.94 (m, 1 H, 21-CH), 3.77 - 3.85 (m, 4 H, 27/28-CH_2), 3.67 - 3.74 (m, 2 H, 27/28-CH_2), 3.60 - 3.67 (m, 2 H, 27/28-CH_2), 3.25 (q, *J*=6.3 Hz, 2 H, 12-CH_2), 3.21 (s, 3 H, 26-CH_3), 2.96 - 3.01 (m, 1H, 23-CH), 2.74 - 2.80 (m, 2 H, 22,23-CH), 2.38 (t, *J*=7.8 Hz, 2 H, 2-CH_2), 2.05 - 2.12 (m, 1 H, 22-CH), 1.64 - 1.68 (m, 4 H, (3,11)-CH_2), 1.51 (s, 9 H, 42-CH_3), 1.39 - 1.43 (m, 2 H, 10-CH_2), 1.29 - 1.36 (m, 12 H, (4-9)-CH_2). ^{13}C NMR (101 MHz, $CDCl_3$) δ_C ppm 172.0 (C1), 171.2 (C24), 169.7 (C20), 169.0 (C25), 167.8 (C19), 162.6 (C33), 155.2 (C29/31), 153.8 (C40), 147.0 (C13), 143.1 (C32), 136.0 (C15), 133.2 (C29/31), 132.5 (C17), 130.7 (C34/35), 130.1 (C34/35), 127.5 (C30), 126.0 (C37/38), 125.4 (C37/38), 124.9 (C36/39), 124.3 (C36/39), 116.5 (C16), 111.2 (C14), 109.9 (C18), 80.6 (C41), 49.6 (C21), 44.9 (C27/28), 44.4 (C27/28), 44.0 (C27/28), 42.6 (C12), 40.7 (C27/28), 33.3 (C2), 31.9 (C23), 29.5 (alkyl CH_2), 29.4 (alkyl CH_2), 29.4 (alkyl CH_2), 29.4 (alkyl CH_2), 29.4 (alkyl CH_2), 29.3 (alkyl CH_2), 29.2 (C11), 28.3 (C42), 27.2 (C26), 26.9 (C10), 25.2 (C3), 22.1 (C22). HRMS (ESI) m/z: $[M+H]^+$ calculated for $C_{46}H_{60}N_9O_8$: 866.4565, found 866.4559.

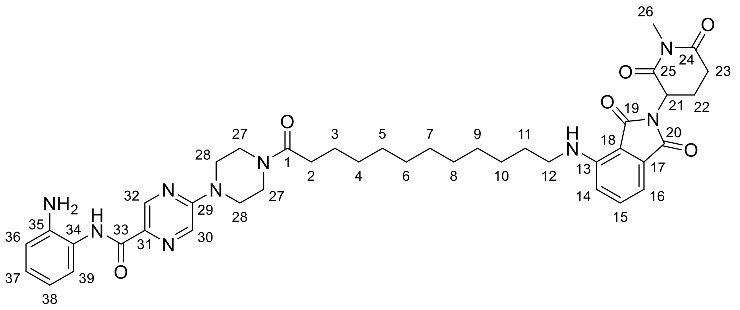

N-(2-aminophenyl)-5-(4-(12-((2-(1-methyl-2,6-dioxopiperidin-3-yl)-1,3-dioxoisoindolin-4-yl)amino)dodecanoyl)piperazin-1-yl)pyrazine-2-carboxamide, 7-Me: TFA (0.5 mL) was added to a stirring solution of **XLV** (35.8 mg, 0.039 mmol) in DCM (2 mL) and the resulting reaction mixture stirred at room temperature for 4 hours. The reaction mixture was concentrated *in vacuo*, then redissolved in EtOAc (25 mL) and washed with sat. $NaHCO_3$ (2 x 25mL) and water (1 x 25 mL). The organic layer was dried over Na_2SO_4, filtered and concentrated *in vacuo* to afford **7-Me** (28.6 mg, 0.012 mmol, 90% yield) as a yellow solid. The product was further purified by column chromatography (0-9% MeOH in DCM) and lyophilised to afford **7-Me** (26.7 mg) as a fluffy yellow solid for biological evaluation. ^{1}H NMR (400 MHz, $CDCl_3$) δ_H ppm 9.27 (s, 1 H, 34-NH), 8.95 (d, *J*=1.1 Hz, 1 H, 32-CH), 8.04 (d, *J*=1.1 Hz, 1 H, 30-CH), 7.42 - 7.52 (m, 2 H, (15,39)-CH), 7.04 - 7.12 (m, 2 H, (14,37)-CH), 6.79 - 6.93 (m, 3 H, (16,36,38)-CH), 6.22 (br t, *J*=6.0 Hz, 1 H, 13-NH), 4.89 - 4.94 (m, 1 H, 21-CH), 3.76 - 3.87 (m, 4 H, 27/28-CH), 3.68 - 3.76 (m, 2 H, 27/28-CH), 3.61 - 3.68 (m, 2 H, 27/28-CH), 3.25 (q, *J*=6.0 Hz, 2 H, 12-CH_2), 3.21 (s, 3 H, 26-CH_3), 2.94 - 3.02 (m, 1 H, 23-CH), 2.73 - 2.83 (m, 2 H, 22,23-CH), 2.38 (t, *J*=7.5 Hz, 2 H, 2-CH_2), 2.06 - 2.12 (m, 1 H, 22-CH), 1.62 - 1.69 (m, 4 H, (3,11)-CH_2), 1.29 - 1.40 (m, 14 H, (4-10)-CH_2). NH_2 not visible. ^{13}C NMR (101 MHz, $CDCl_3$) δ_C 172.0 (C1), 171.2 (C24), 169.7 (C20), 169.0 (C25), 167.8 (C19), 162.0 (C33), 155.2 (C29/31), 147.0 (C13), 142.9 (C32), 140.3 (C35), 136.0 (C15), 133.4 (C29/31), 132.5 (C17), 127.6 (C30), 126.7 (C39), 124.6 (C37), 124.4 (C34), 119.6 (C38), 118.1 (C36), 116.5 (C16), 111.2 (C14), 109.9 (C18), 49.6 (C21), 44.9 (C27/28), 44.4 (C27/28), 44.0 (C27/28), 42.6 (C12), 40.7 (C27/28), 33.3 (C2), 31.9 (C23), 29.5 (alkyl CH_2), 29.4 (alkyl CH_2), 29.4 (alkyl CH_2), 29.4 (alkyl CH_2), 29.4 (alkyl CH_2), 29.4 (alkyl CH_2), 29.3 (alkyl CH_2), 29.2 (C11), 27.2 (C26), 26.9 (C10), 25.2 (C3), 22.1 (C22). HRMS (ESI) m/z: $[M+H]^+$ calculated for $C_{41}H_{52}N_9O_6$: 766.4041, found 766.4031.

REFERENCES:

1 Y. Itoh, P. Zhan, T. Tojo, P. Jaikhan, Y. Ota, M. Sukuzi, Y. Li, Z. Hui, Y. Moriyama, Y. Takada, Y. Yamashita, M. Oba, S. Uchida, M. Masuda, S. Ito, Y. Sowa, T. Sakai, T. Suzuki, *J. Med. Chem*. 2023, **66**, 15171.

2 F. F. Wagner, Y. L. Zhang, D. M. Fass, N. Joseph, J. P. Gale, M. Weïwer, P. McCarren, S. L. Fisher, T. Kaya, W. N. Zhao, S. A. Reis, K. M. Hennig, M. Thomas, B. C. Lemercier, M. C. Lewis, J. S. Guan, M. P. Moyer, E. Scolnick, S. J. Haggarty, L. H. Tsai, E. B. Holson, *Chem. Sci*. 2015, **6**, 804.

3 C. Steinebach, S. Lindner, N. D. Udeshi, D. C. Mani, H. Kehm,S. Köpff, S. A. Carr, M. Gütschow, J. Krönke, *ACS Chem. Biol.* 2018, **13**, 2771.

Appendix: ^{1}H NMR and ^{13}C NMR of the Compounds

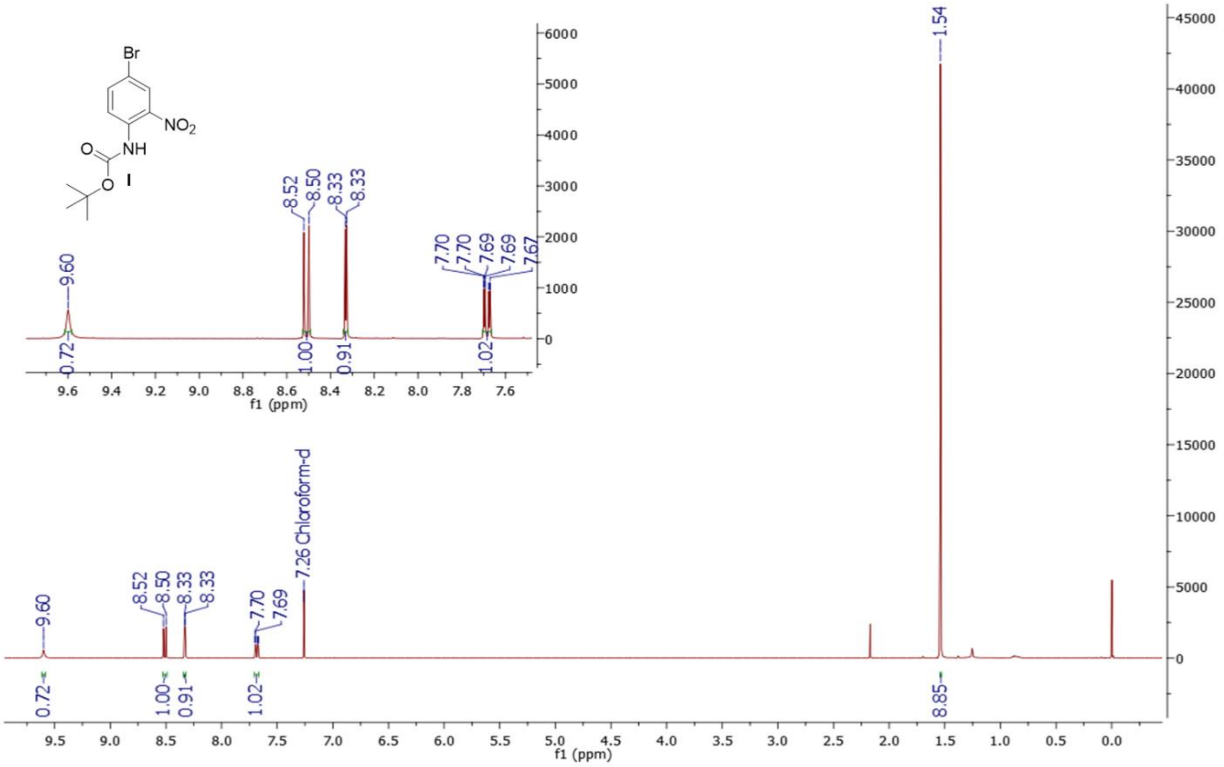

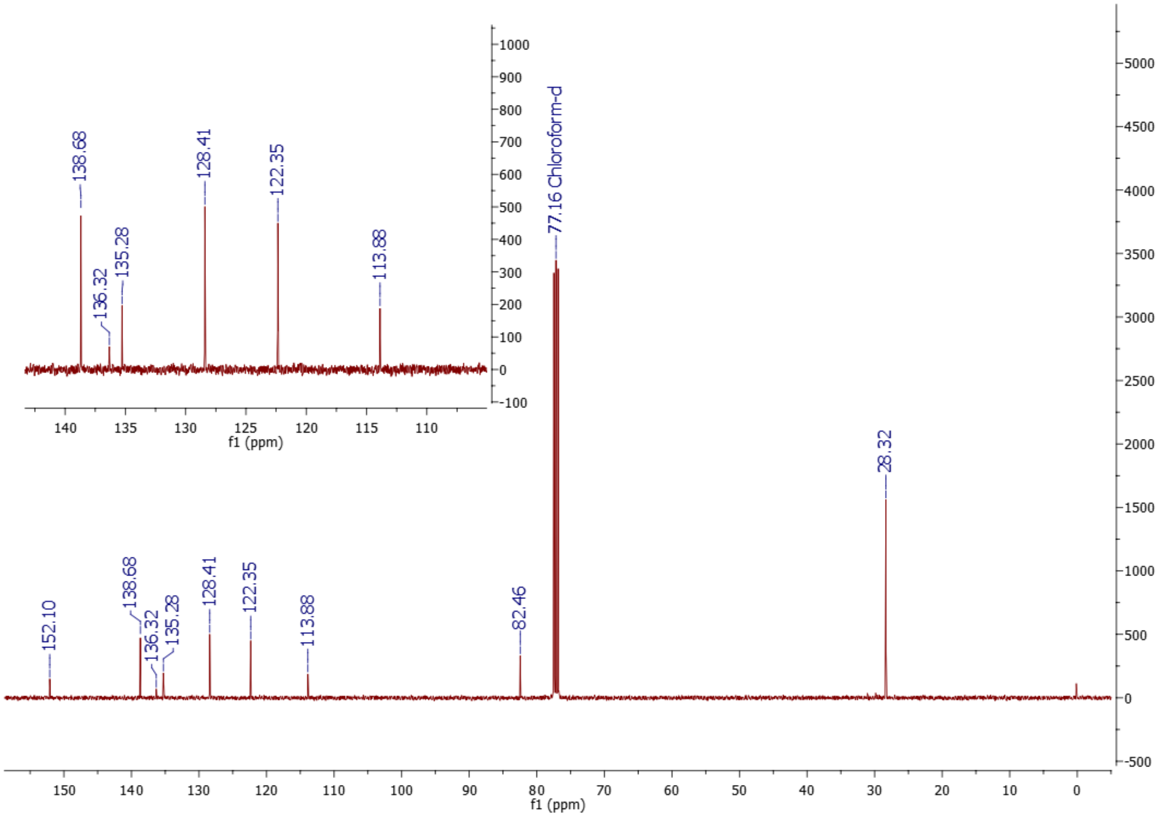

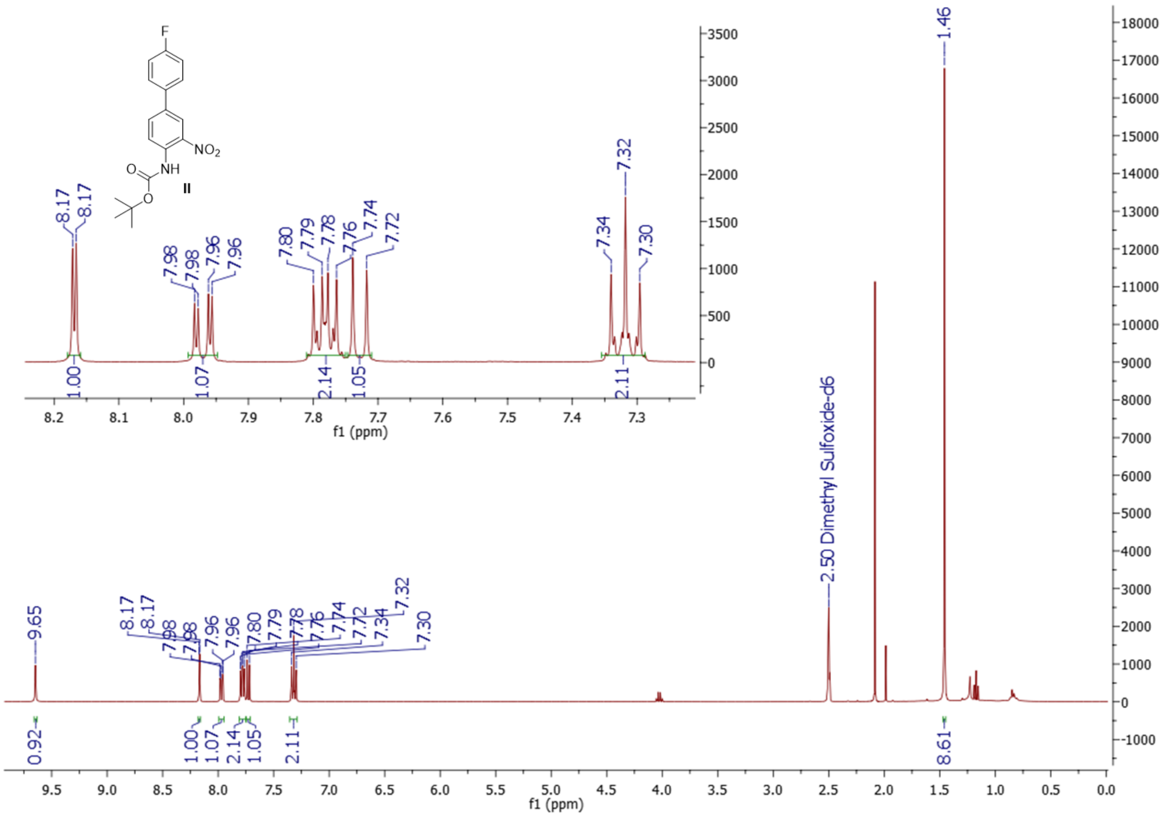
F
NO_2
NH
O
O
II
8.17
8.17
7.98
7.98
7.96
7.96
7.80
7.79
7.78
7.76
7.74
7.72
7.34
7.32
7.30
1.00
1.07
2.14
1.05
2.11
f1 (ppm)
9.65
2.50 Dimethyl Sulfoxide-d6
1.46
0.92
8.61

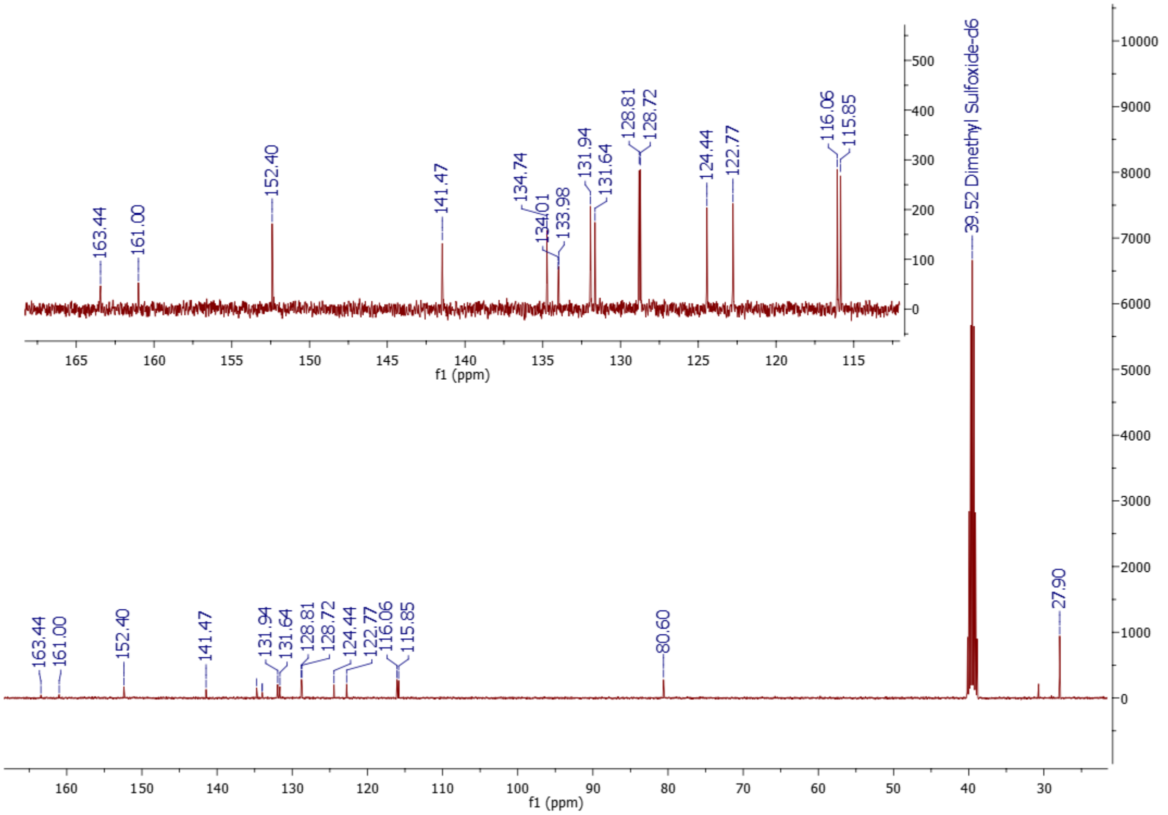
163.44
161.00
152.40
141.47
134.74
134.01
133.98
131.94
131.64
128.81
128.72
124.44
122.77
116.06
115.85
39.52 Dimethyl Sulfoxide-d6
80.60
27.90
f1 (ppm)

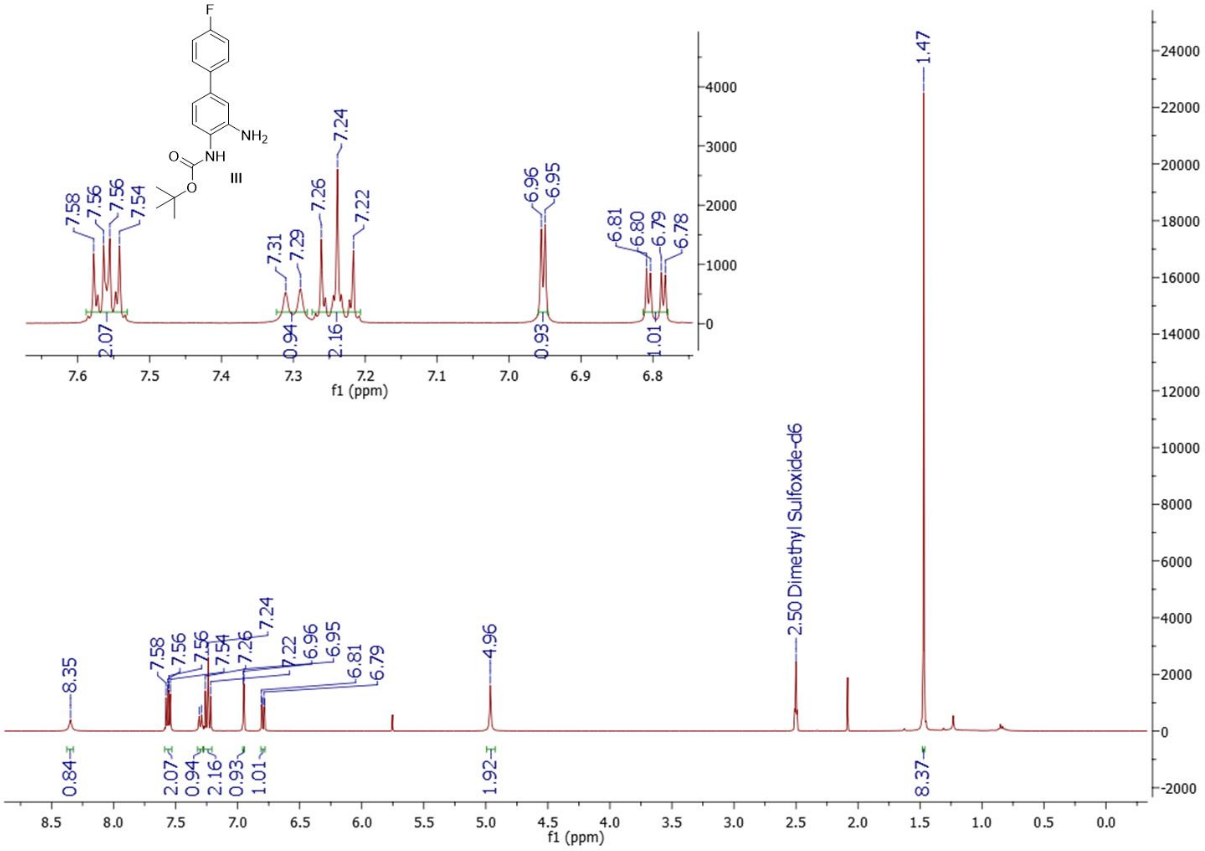
F
NH2
NH
O
O
III
2.50 Dimethyl Sulfoxide-d6
8.35
4.96
1.47
0.84
2.07
0.94
2.16
0.93
1.01
1.92
8.37
f1 (ppm)

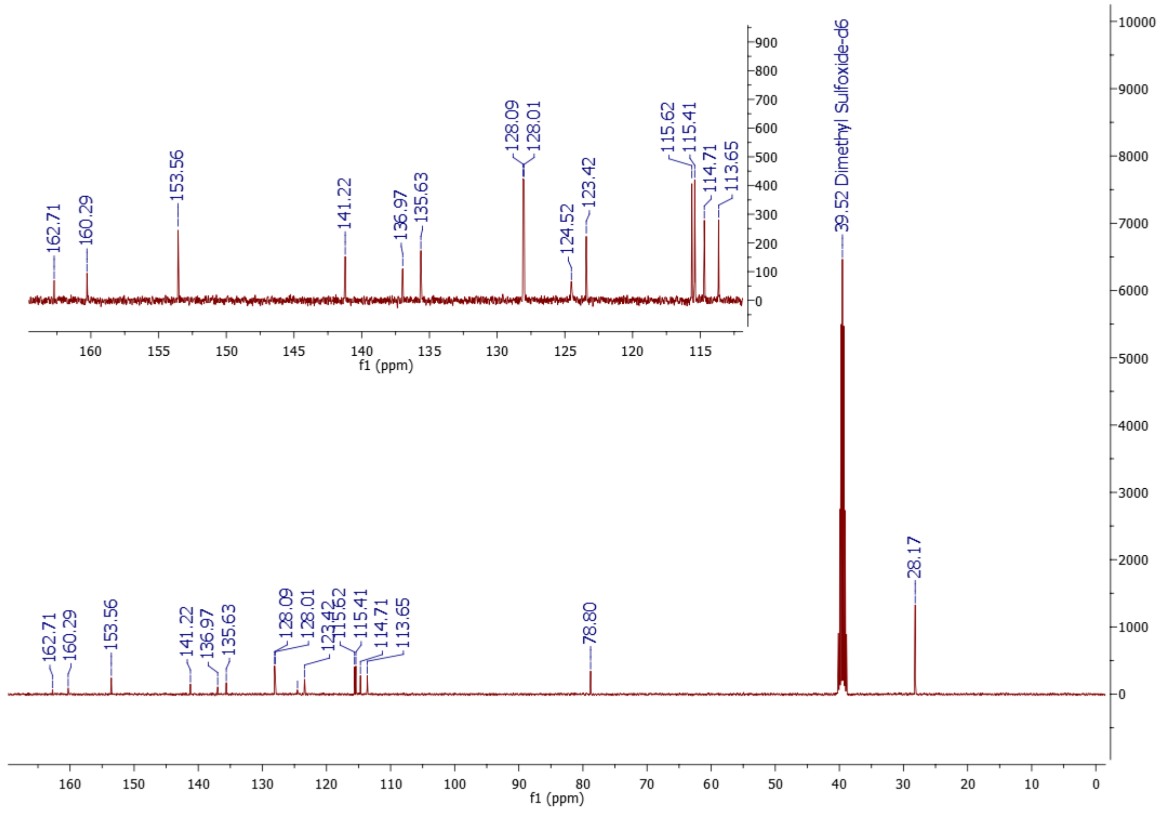
39.52 Dimethyl Sulfoxide-d6
162.71
160.29
153.56
141.22
136.97
135.63
128.09
128.01
124.52
123.42
115.62
115.41
114.71
113.65
78.80
28.17
f1 (ppm)

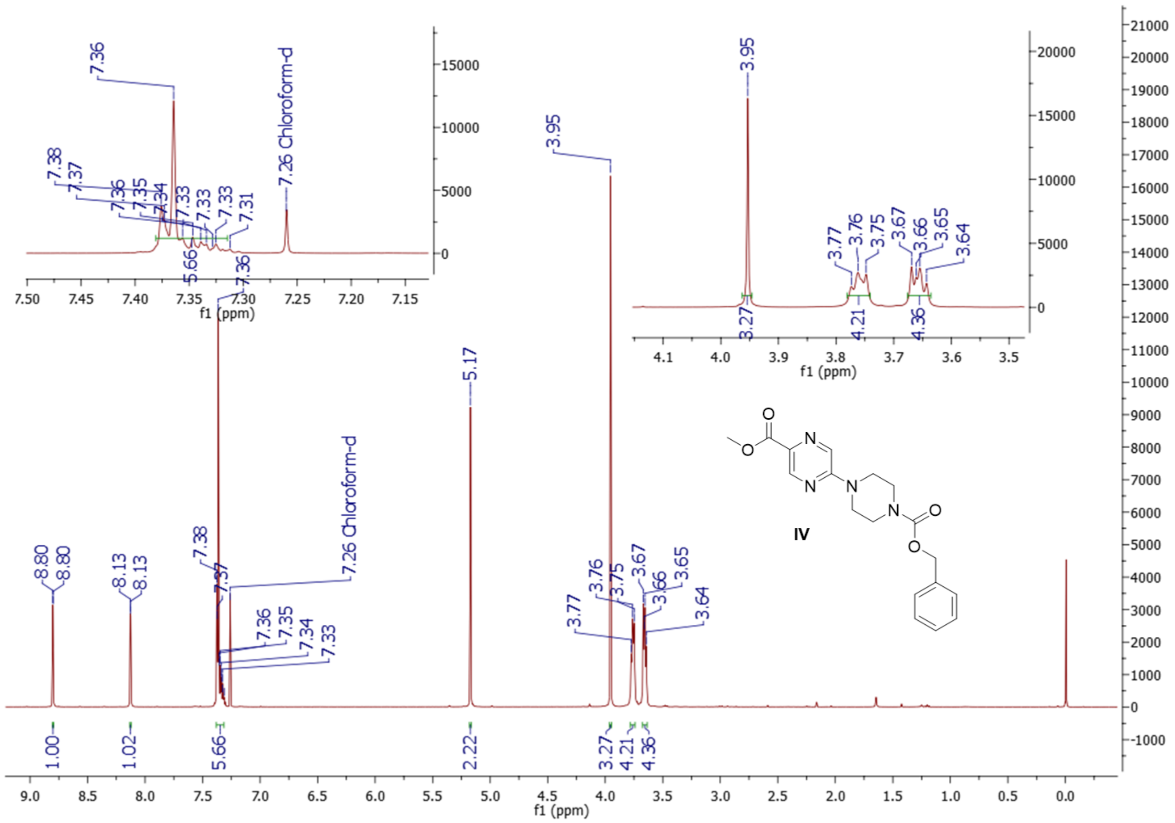
IV
8.80
8.80
8.13
8.13
7.38
7.37
7.36
7.35
7.34
7.33
7.26 Chloroform-d
5.17
3.95
3.77
3.76
3.75
3.67
3.66
3.65
3.64
1.00
1.02
5.66
2.22
3.27
4.21
4.36
f1 (ppm)

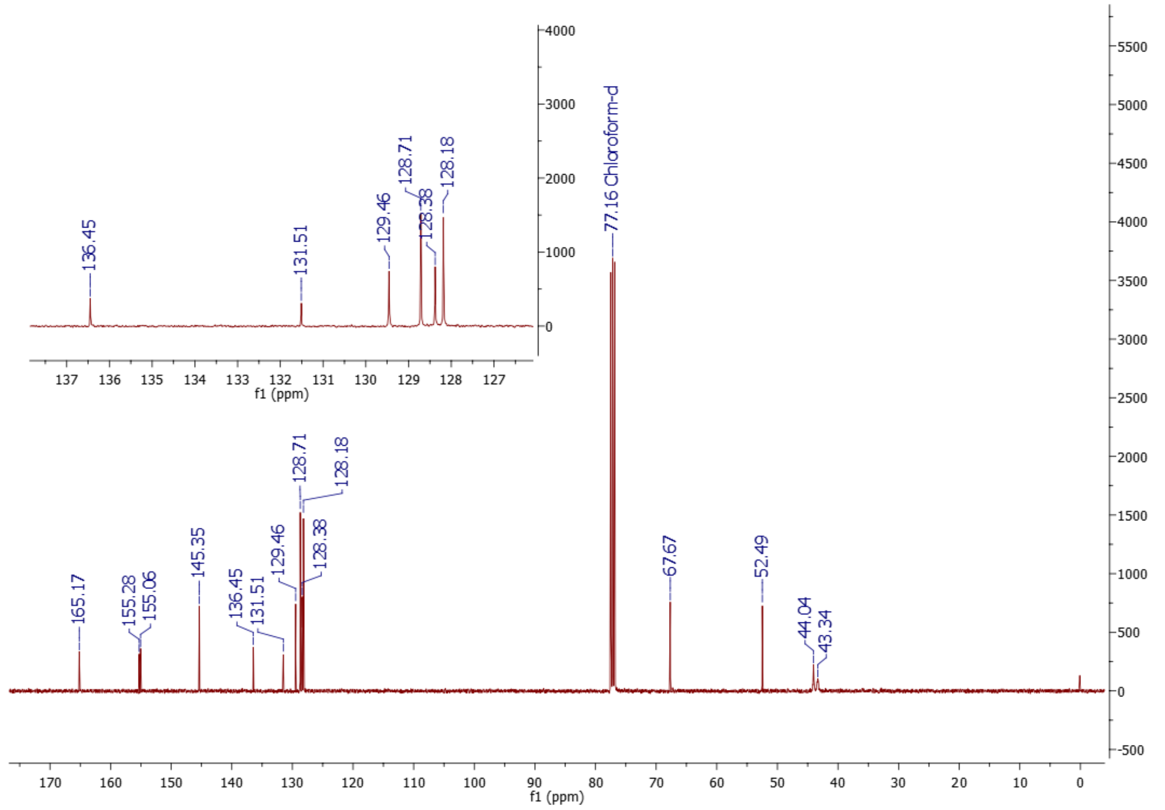
165.17
155.28
155.06
145.35
136.45
131.51
129.46
128.71
128.38
128.18
77.16 Chloroform-d
67.67
52.49
44.04
43.34
f1 (ppm)

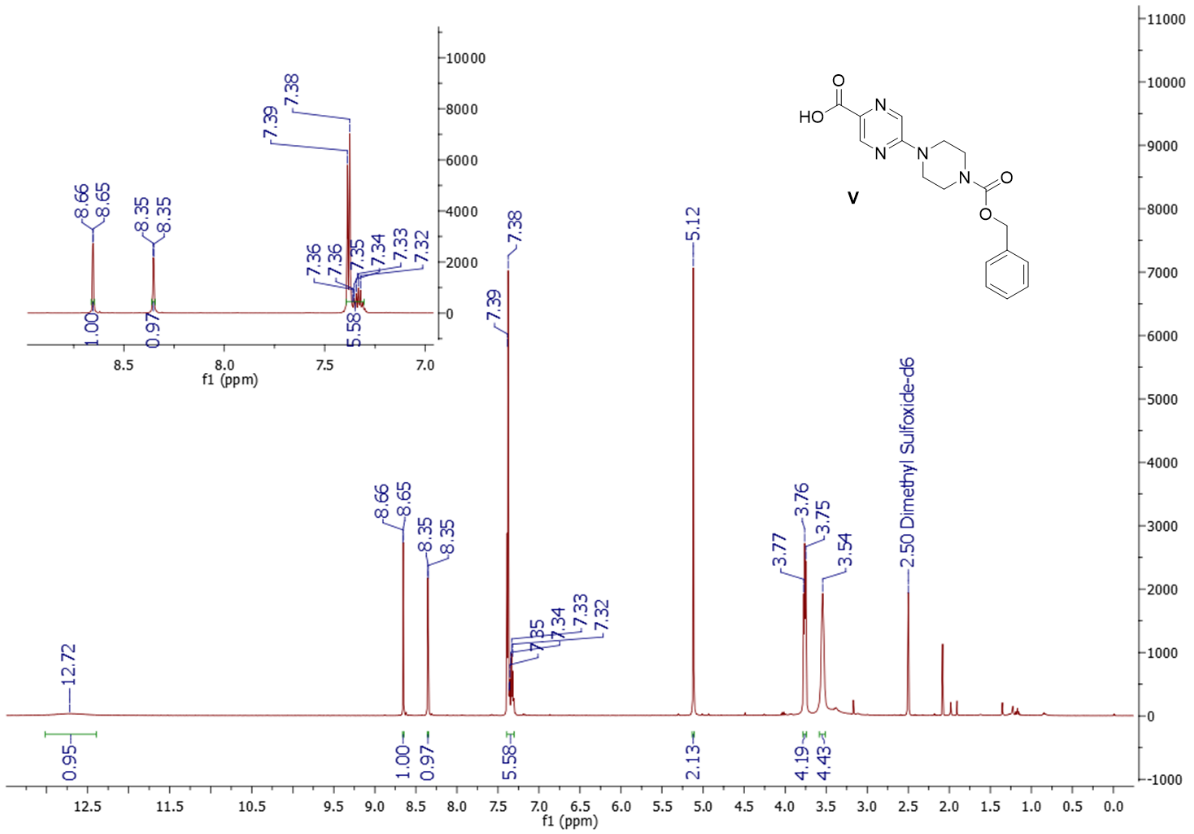

V
12.72
8.66
8.65
8.35
8.35
7.39
7.38
7.36
7.36
7.35
7.34
7.33
7.32
5.12
3.77
3.76
3.75
3.54
2.50 Dimethyl Sulfoxide-d6
0.95
1.00
0.97
5.58
2.13
4.19
4.43
f1 (ppm)

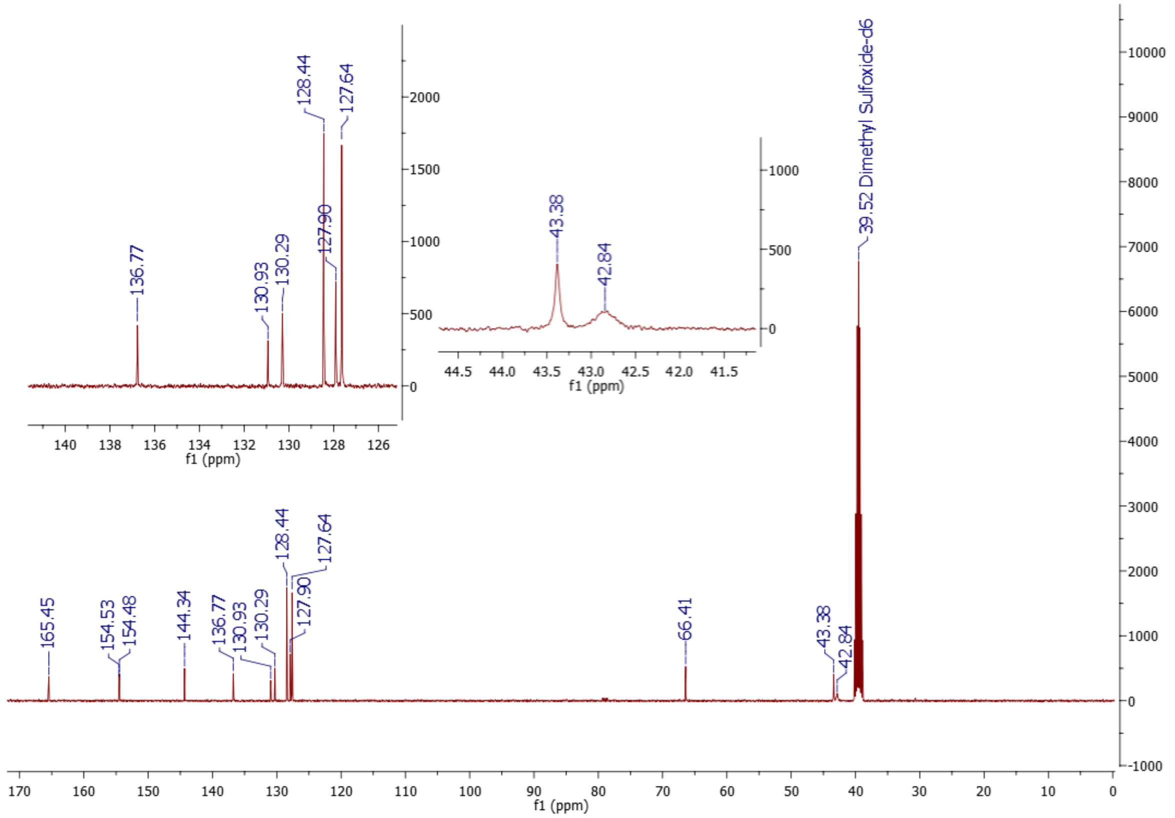

165.45
154.53
154.48
144.34
136.77
130.93
130.29
128.44
127.90
127.64
66.41
43.38
42.84
39.52 Dimethyl Sulfoxide-d6
f1 (ppm)

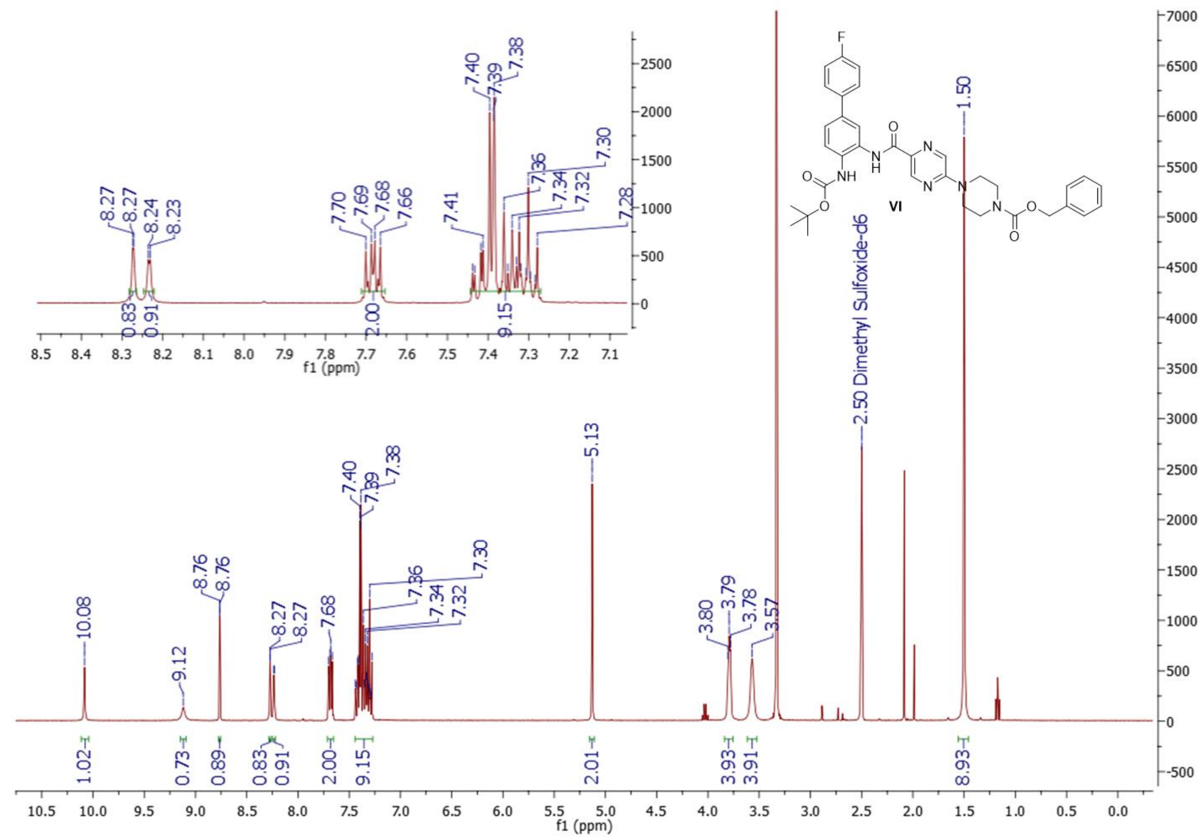
VI
2.50 Dimethyl Sulfoxide-d6
10.08
9.12
8.76
8.76
8.27
8.27
8.24
8.23
7.70
7.69
7.68
7.66
7.41
7.40
7.39
7.38
7.36
7.34
7.32
7.30
7.28
5.13
3.80
3.79
3.78
3.57
1.50
1.02
0.73
0.89
0.83
0.91
2.00
9.15
2.01
3.93
3.91
8.93
f1 (ppm)

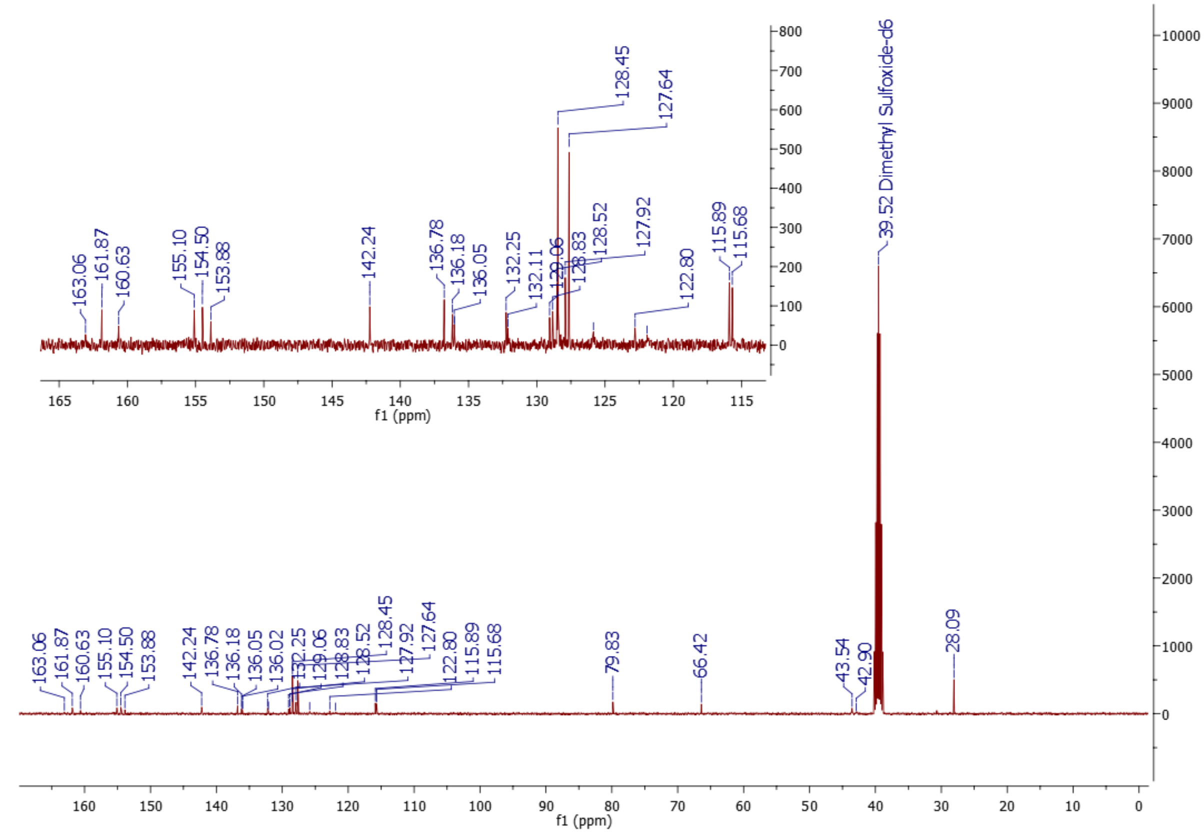
39.52 Dimethyl Sulfoxide-d6
163.06
161.87
160.63
155.10
154.50
153.88
142.24
136.78
136.18
136.05
136.02
132.25
132.11
129.06
128.83
128.52
128.45
127.92
127.64
122.80
115.89
115.68
79.83
66.42
43.54
42.90
28.09
f1 (ppm)

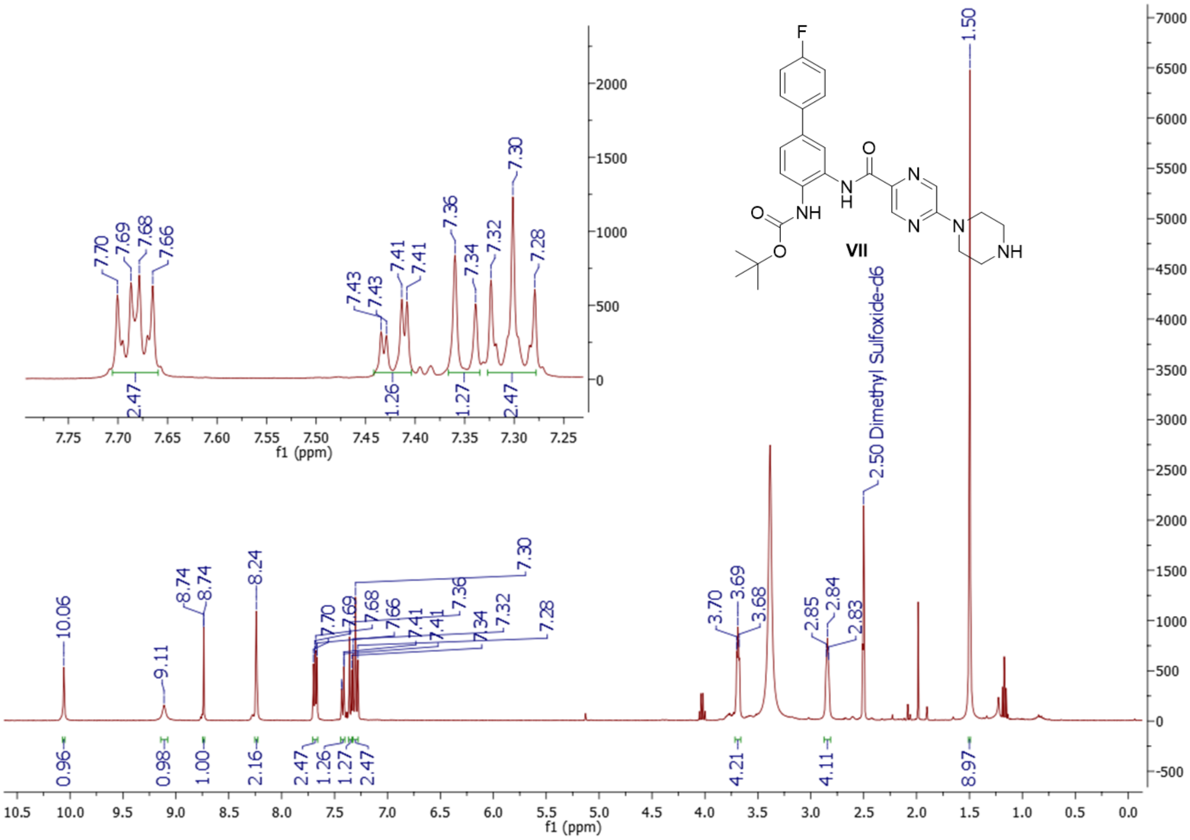
VII
F
NH
O
O
N
H
N
N
N
NH
10.06
9.11
8.74
8.74
8.24
7.70
7.69
7.68
7.66
7.43
7.43
7.41
7.41
7.36
7.34
7.32
7.30
7.28
3.70
3.69
3.68
2.85
2.84
2.83
2.50 Dimethyl Sulfoxide-d6
1.50
0.96
0.98
1.00
2.16
2.47
1.26
1.27
2.47
4.21
4.11
8.97
f1 (ppm)

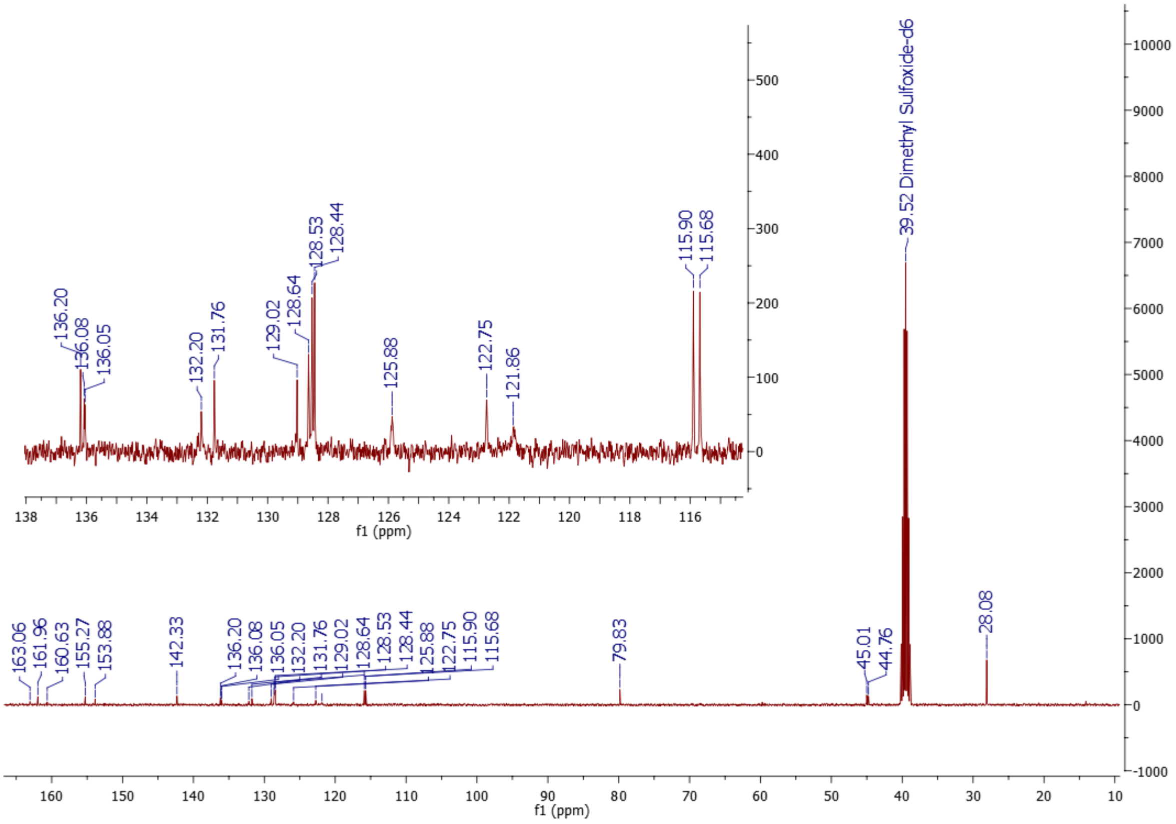
163.06
161.96
160.63
155.27
153.88
142.33
136.20
136.08
136.05
132.20
131.76
129.02
128.64
128.53
128.44
125.88
122.75
121.86
115.90
115.68
79.83
45.01
44.76
39.52 Dimethyl Sulfoxide-d6
28.08
f1 (ppm)

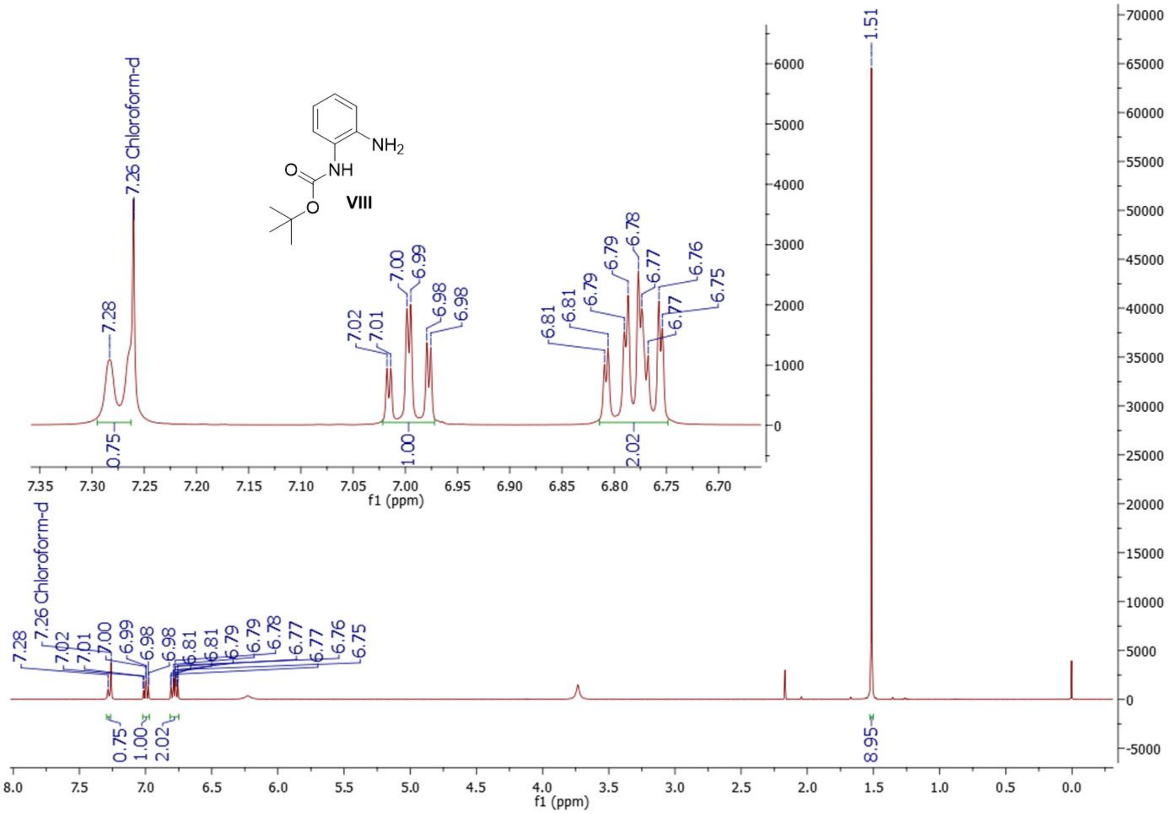
NH2
O
NH
O
VIII
7.26 Chloroform-d
1.51
0.75
1.00
2.02
8.95
f1 (ppm)

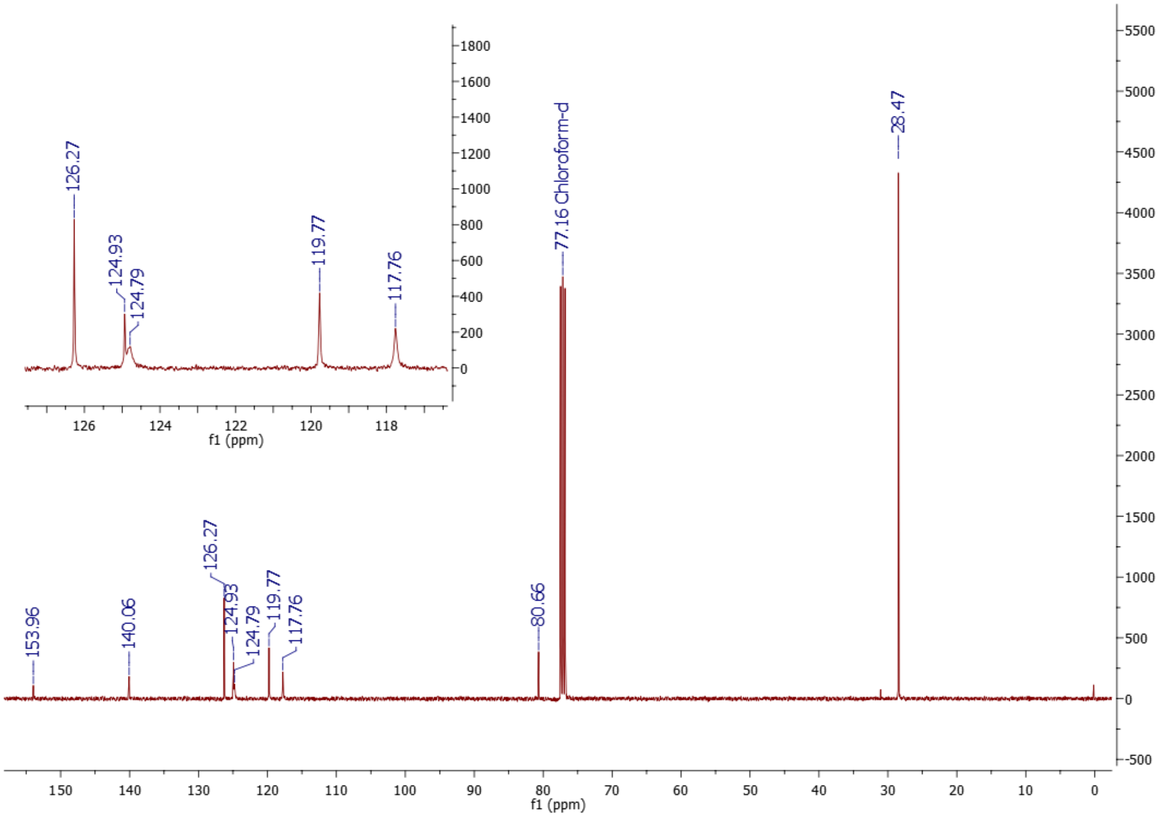
153.96
140.06
126.27
124.93
124.79
119.77
117.76
80.66
77.16 Chloroform-d
28.47
f1 (ppm)

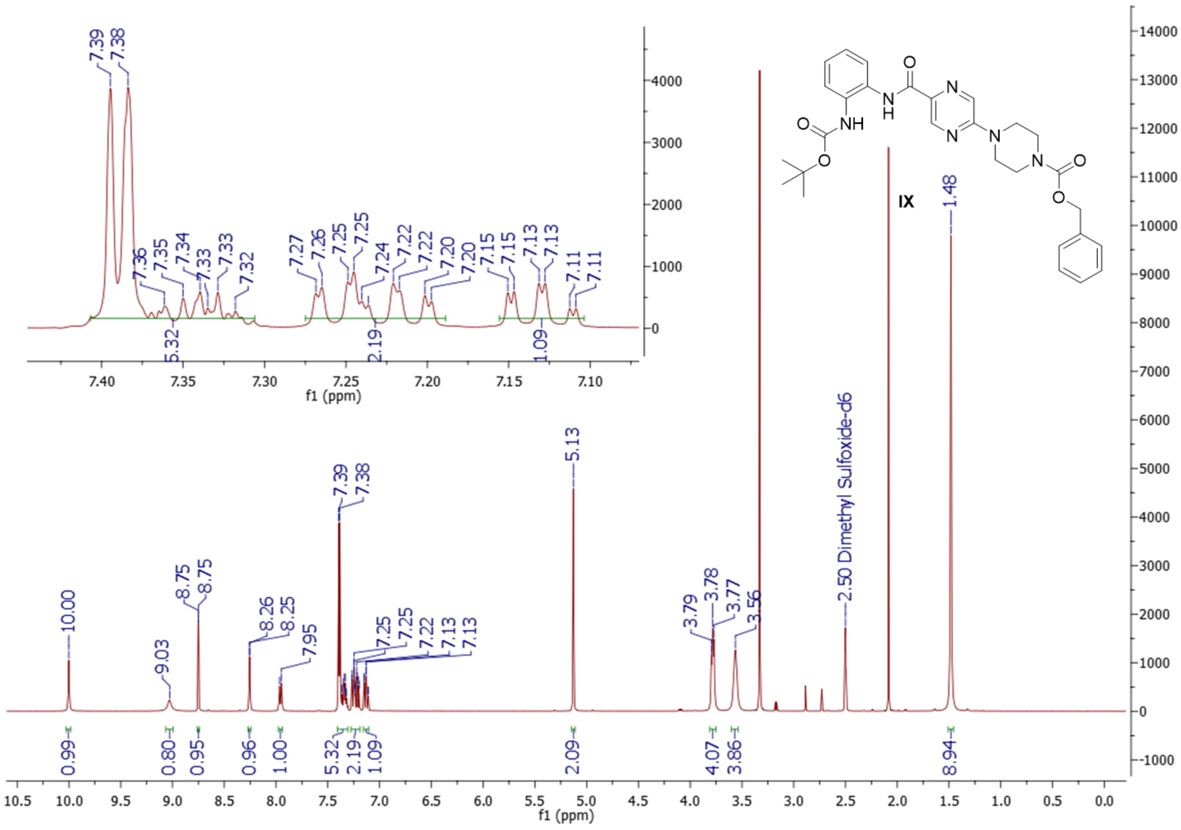
IX
2.50 Dimethyl Sulfoxide-d6
f1 (ppm)

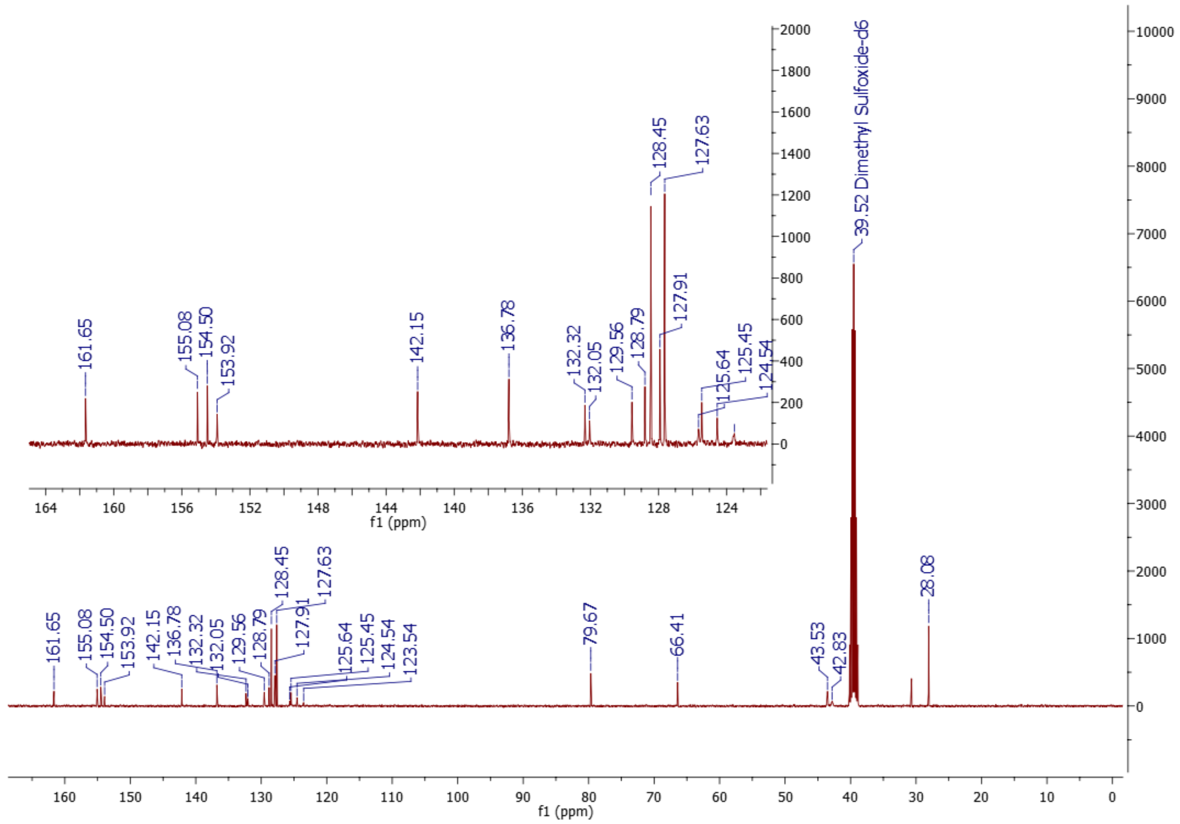
39.52 Dimethyl Sulfoxide-d6
f1 (ppm)

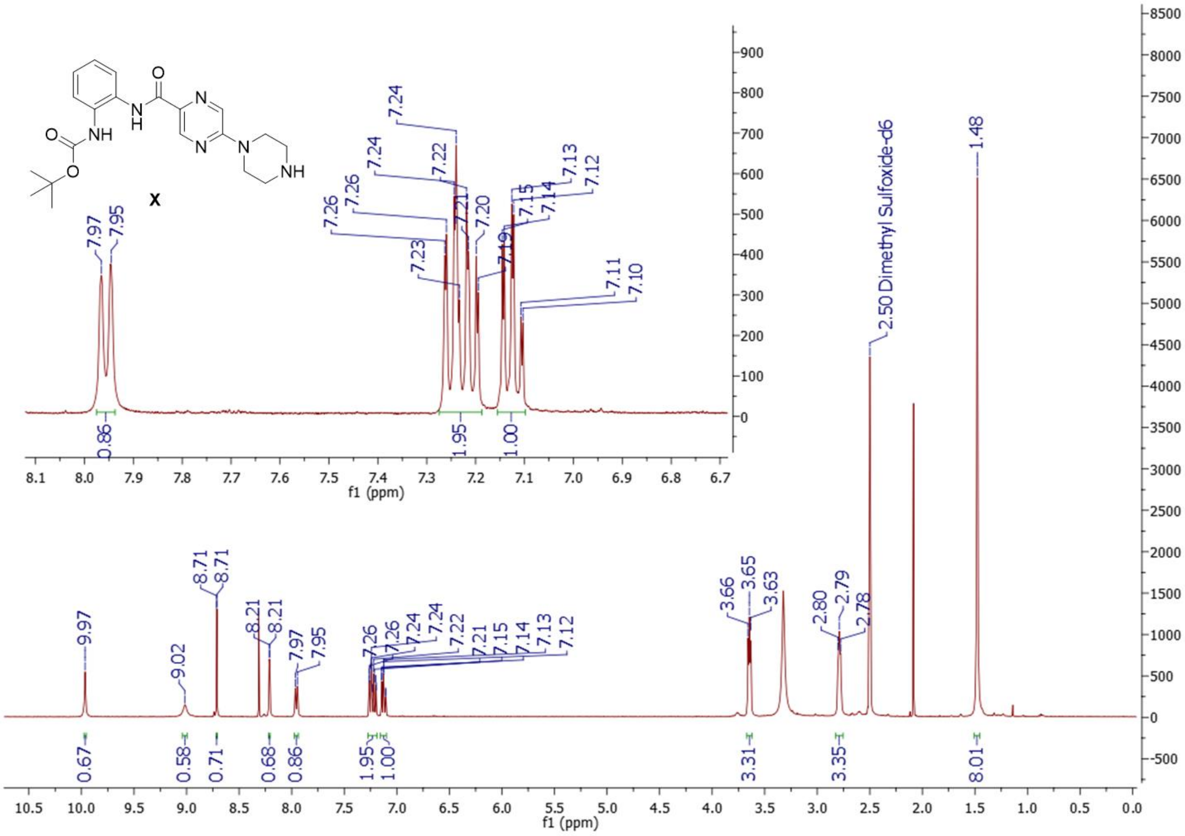

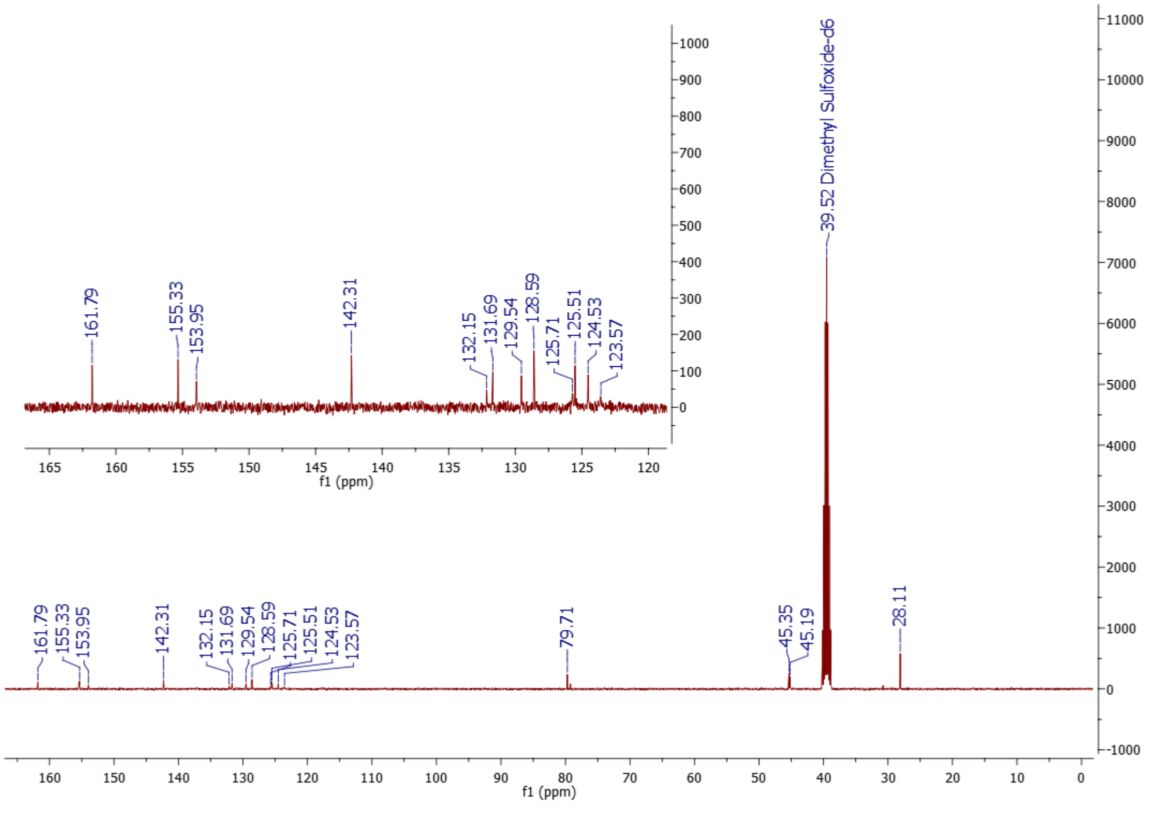

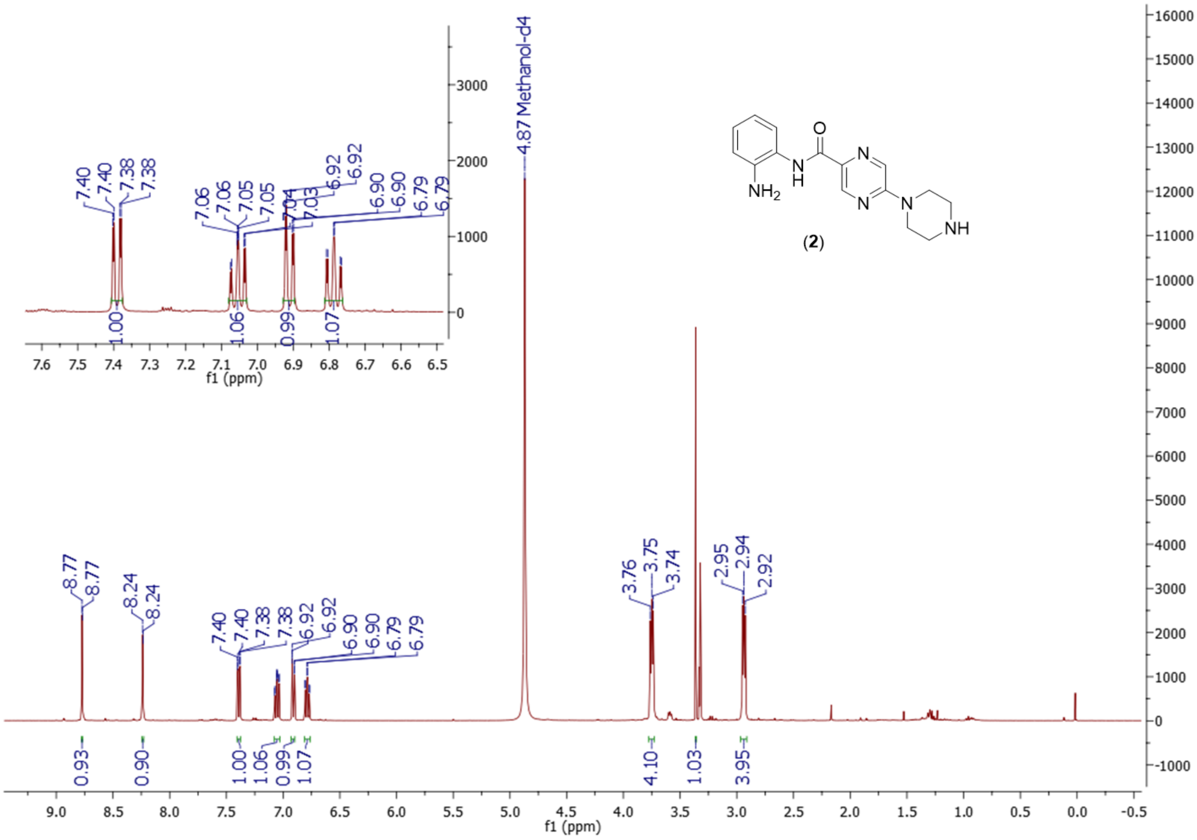
4.87 Methanol-d4
(2)
NH2
N
H
O
N
N
N
NH
8.77
8.77
8.24
8.24
7.40
7.40
7.38
7.38
7.06
7.06
7.05
7.05
7.04
7.03
6.92
6.92
6.90
6.90
6.79
6.79
3.76
3.75
3.74
2.95
2.94
2.92
0.93
0.90
1.00
1.06
0.99
1.07
4.10
1.03
3.95
f1 (ppm)

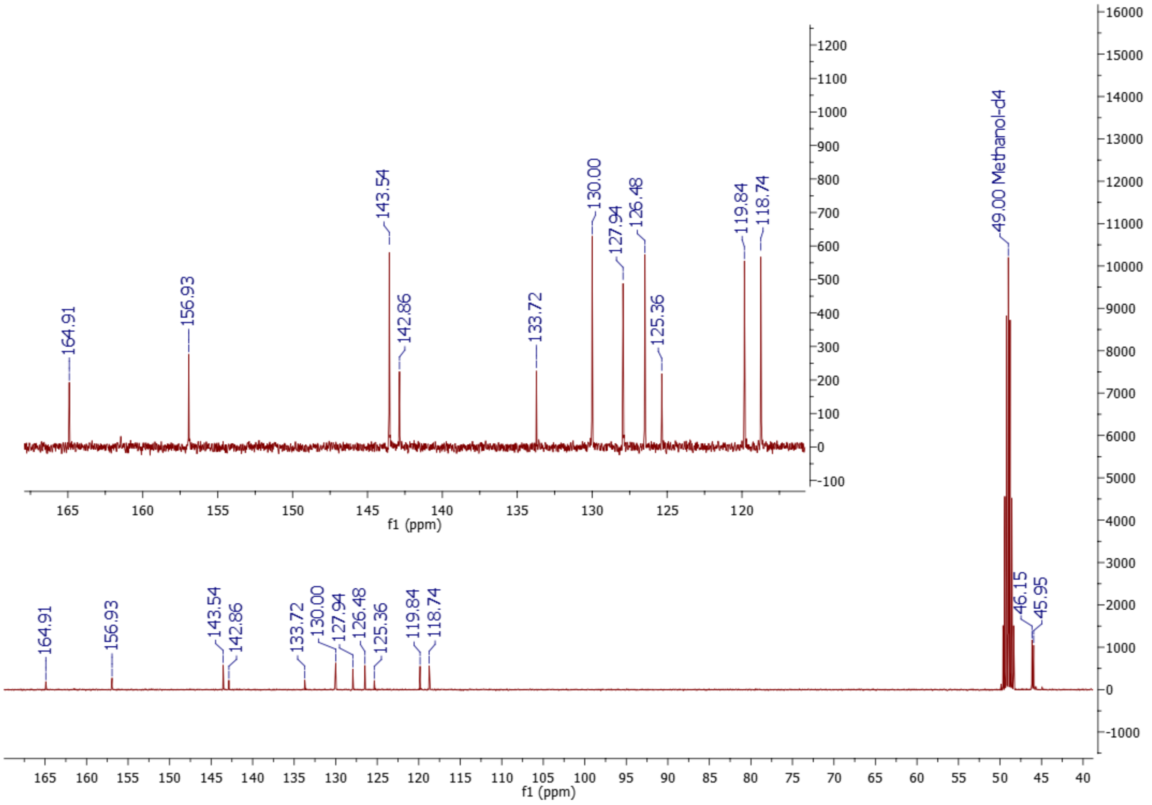
164.91
156.93
143.54
142.86
133.72
130.00
127.94
126.48
125.36
119.84
118.74
49.00 Methanol-d4
46.15
45.95
f1 (ppm)

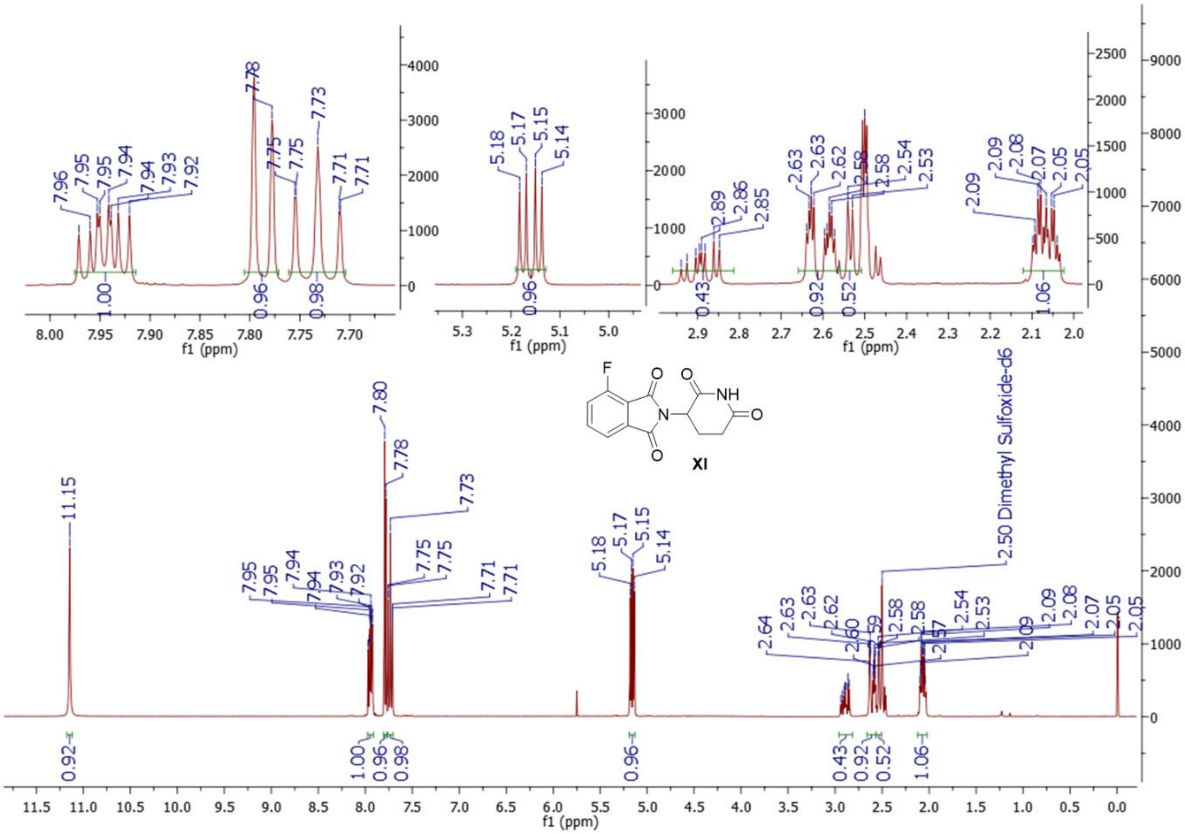
XI
2.50 Dimethyl Sulfoxide-d6
f1 (ppm)

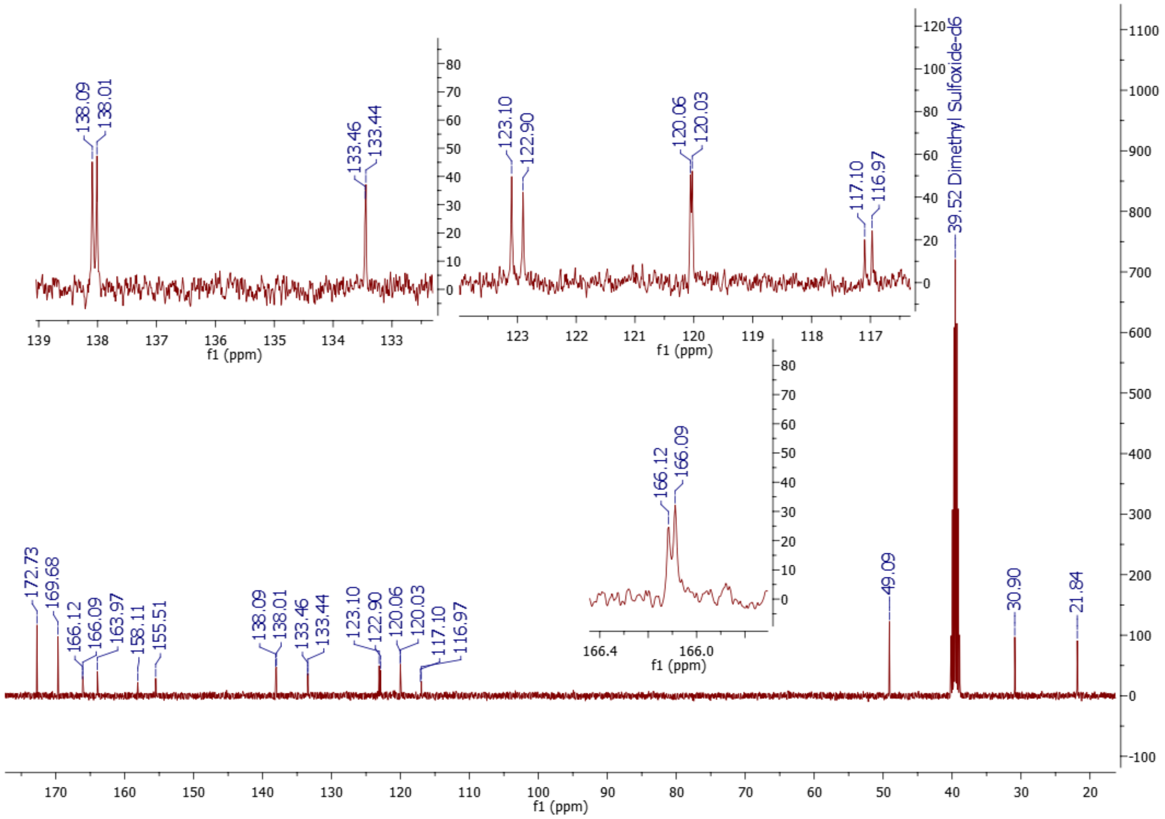
39.52 Dimethyl Sulfoxide-d6
f1 (ppm)

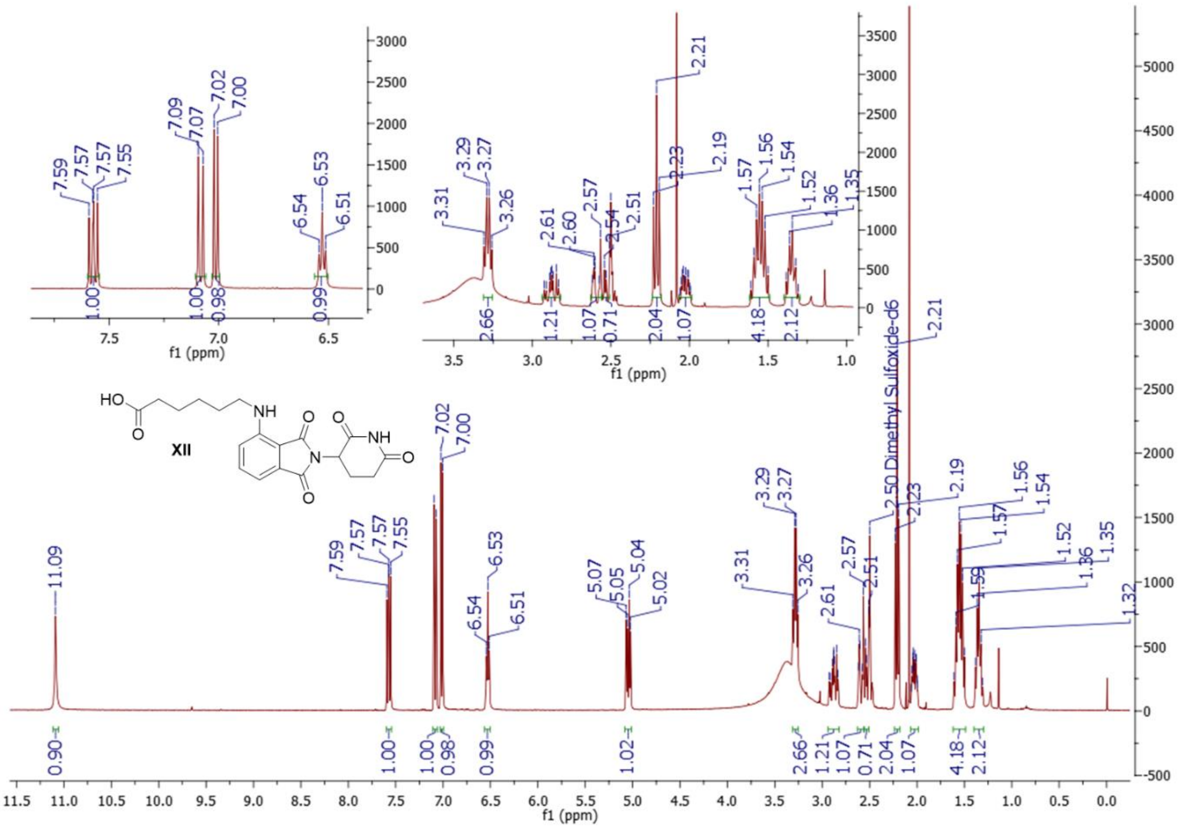
XII
f1 (ppm)
2.50 Dimethyl Sulfoxide-d6

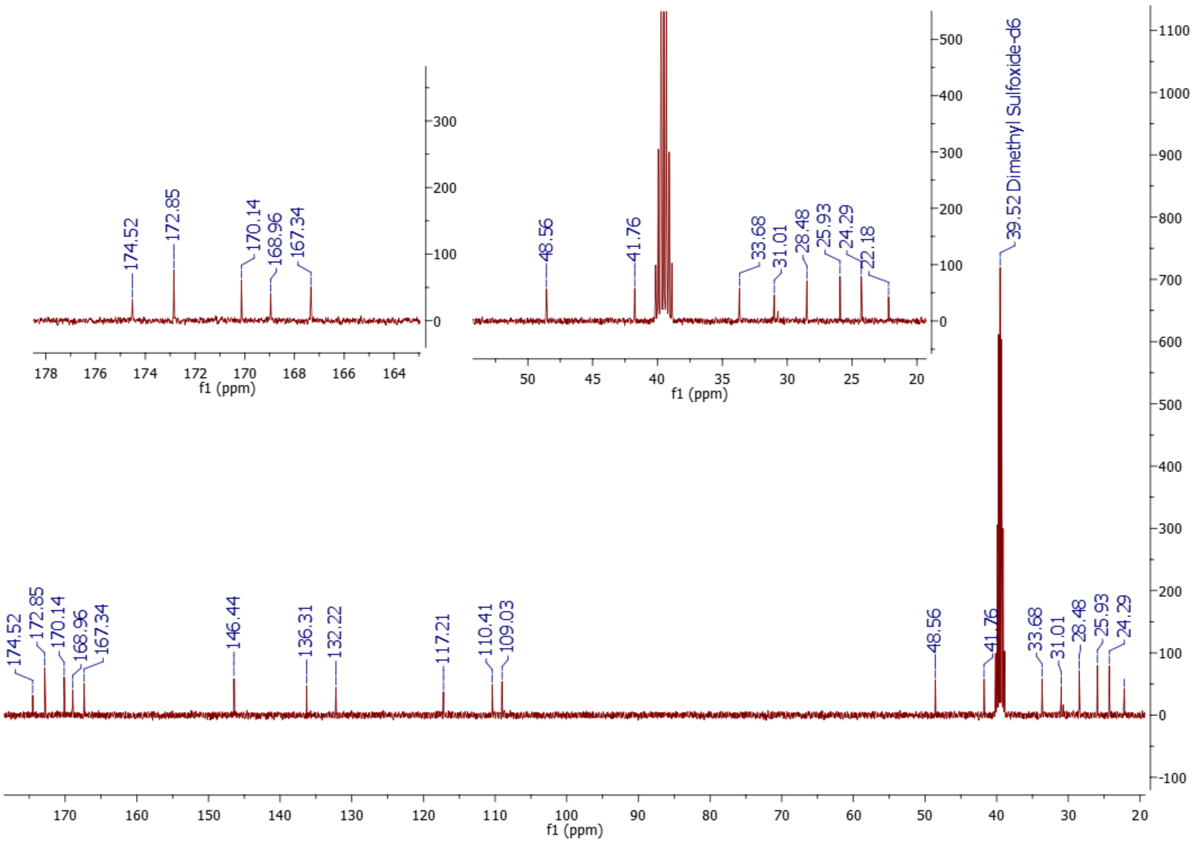
f1 (ppm)
39.52 Dimethyl Sulfoxide-d6

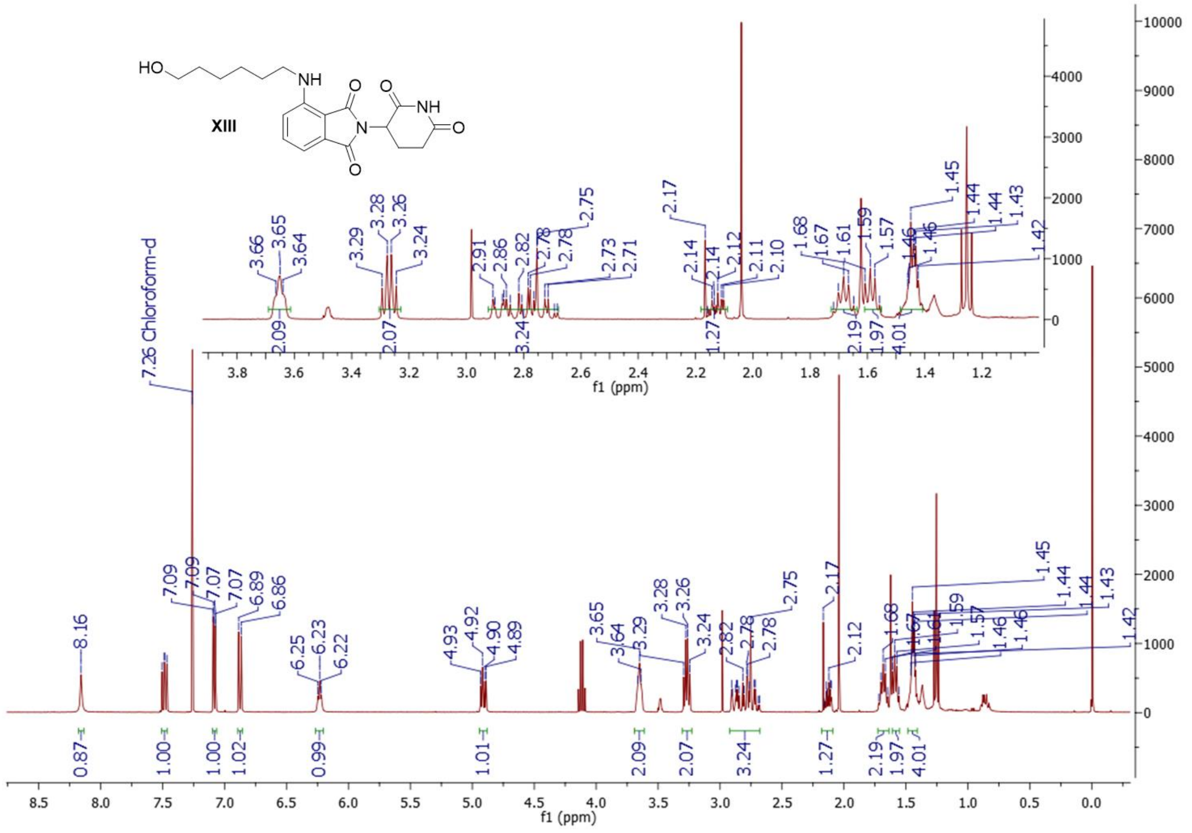

HO
NH
XIII
7.26 Chloroform-d
f1 (ppm)

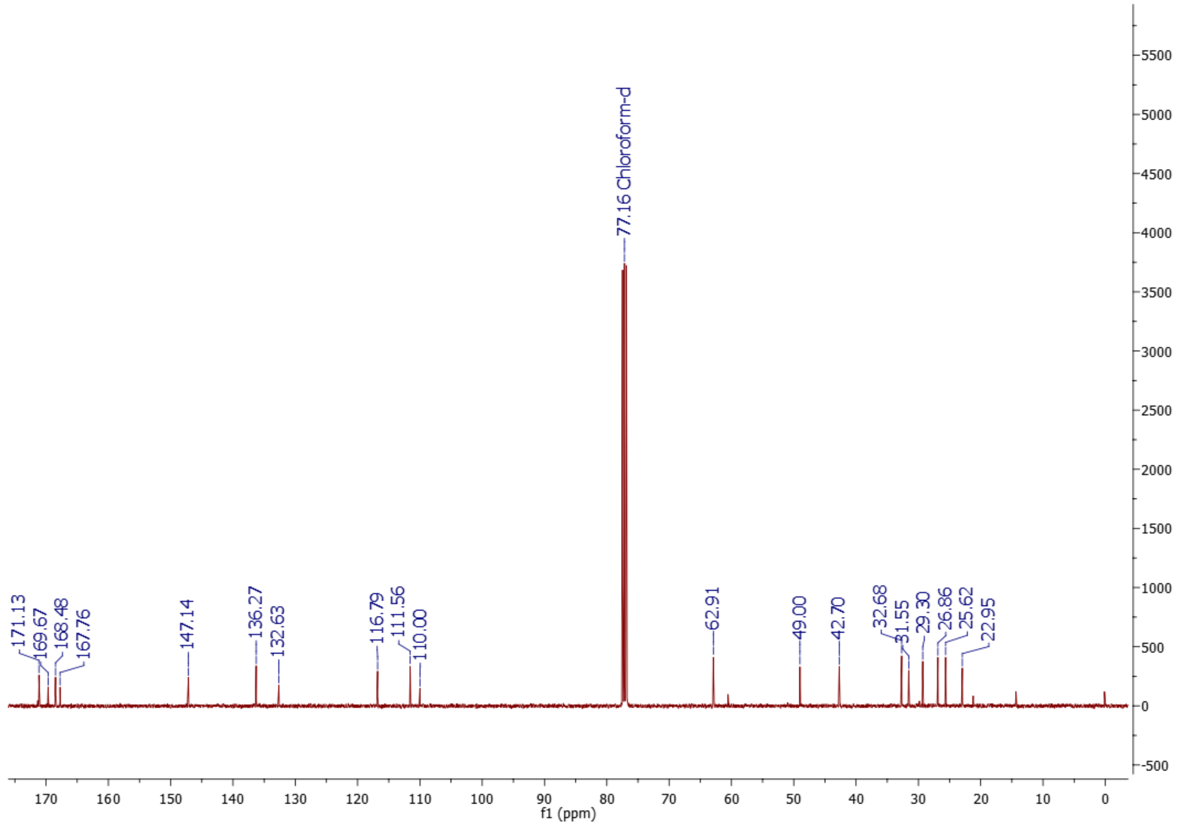

77.16 Chloroform-d
171.13
169.67
168.48
167.76
147.14
136.27
132.63
116.79
111.56
110.00
62.91
49.00
42.70
32.68
31.55
29.30
26.86
25.62
22.95
f1 (ppm)

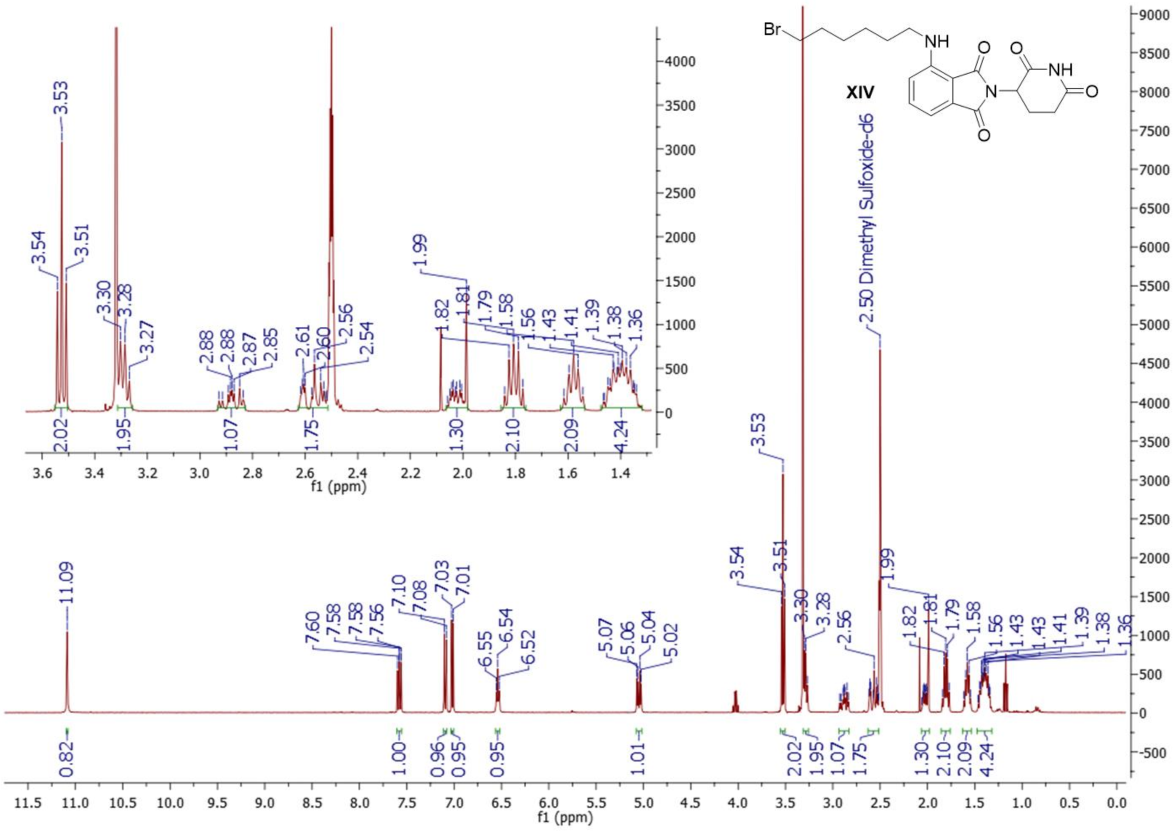
XIV
2.50 Dimethyl Sulfoxide-d6
f1 (ppm)

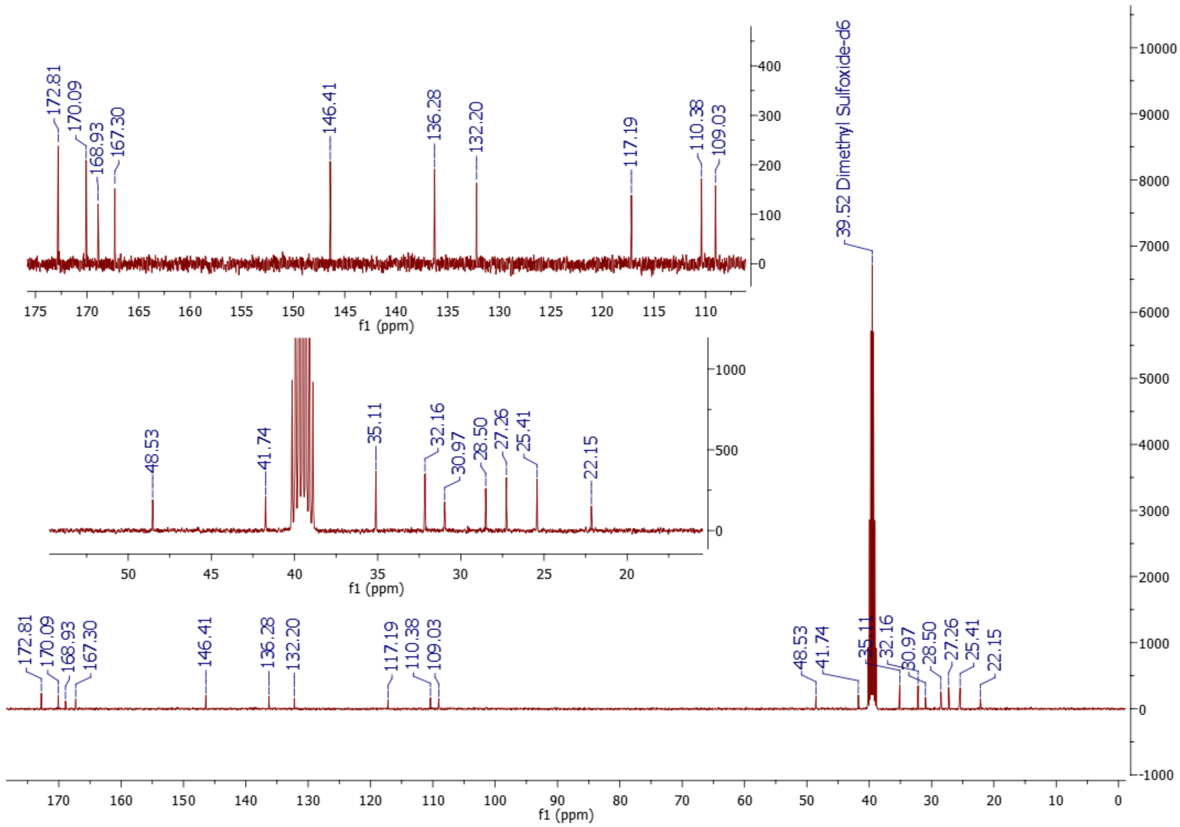
39.52 Dimethyl Sulfoxide-d6
f1 (ppm)

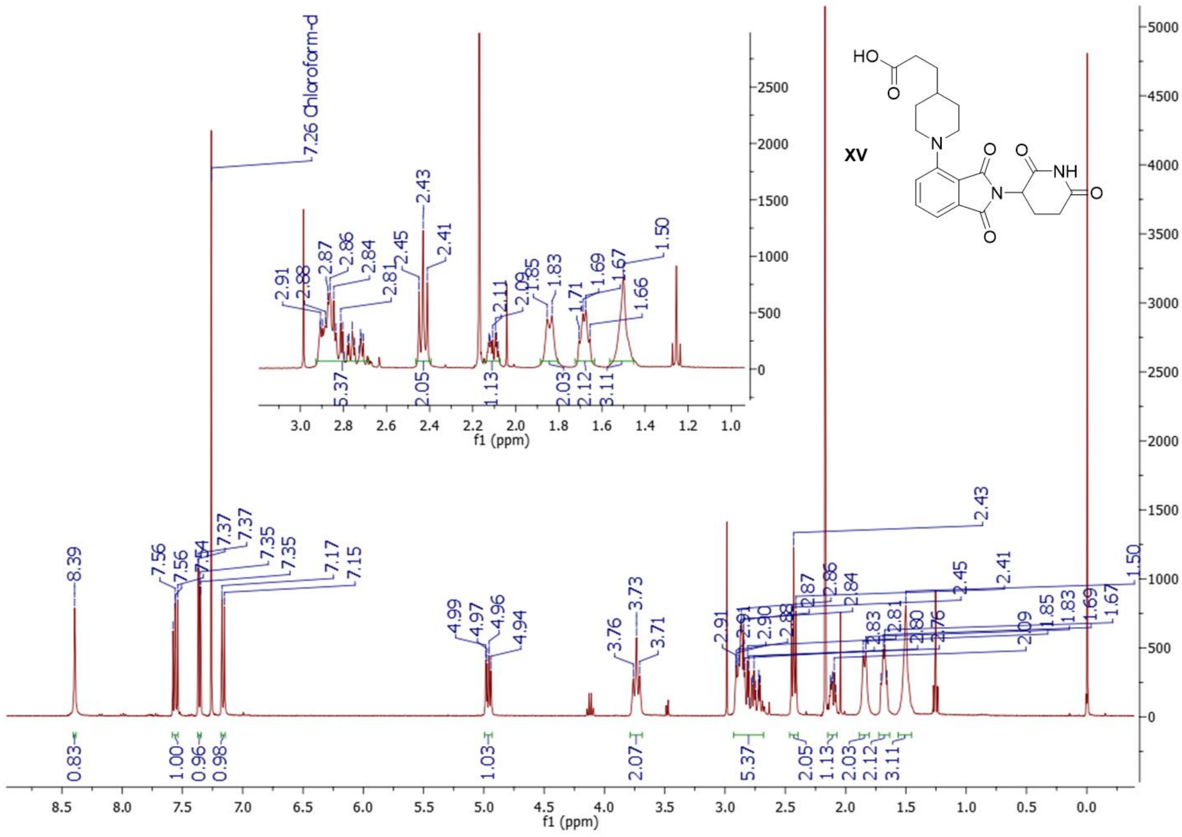

XV

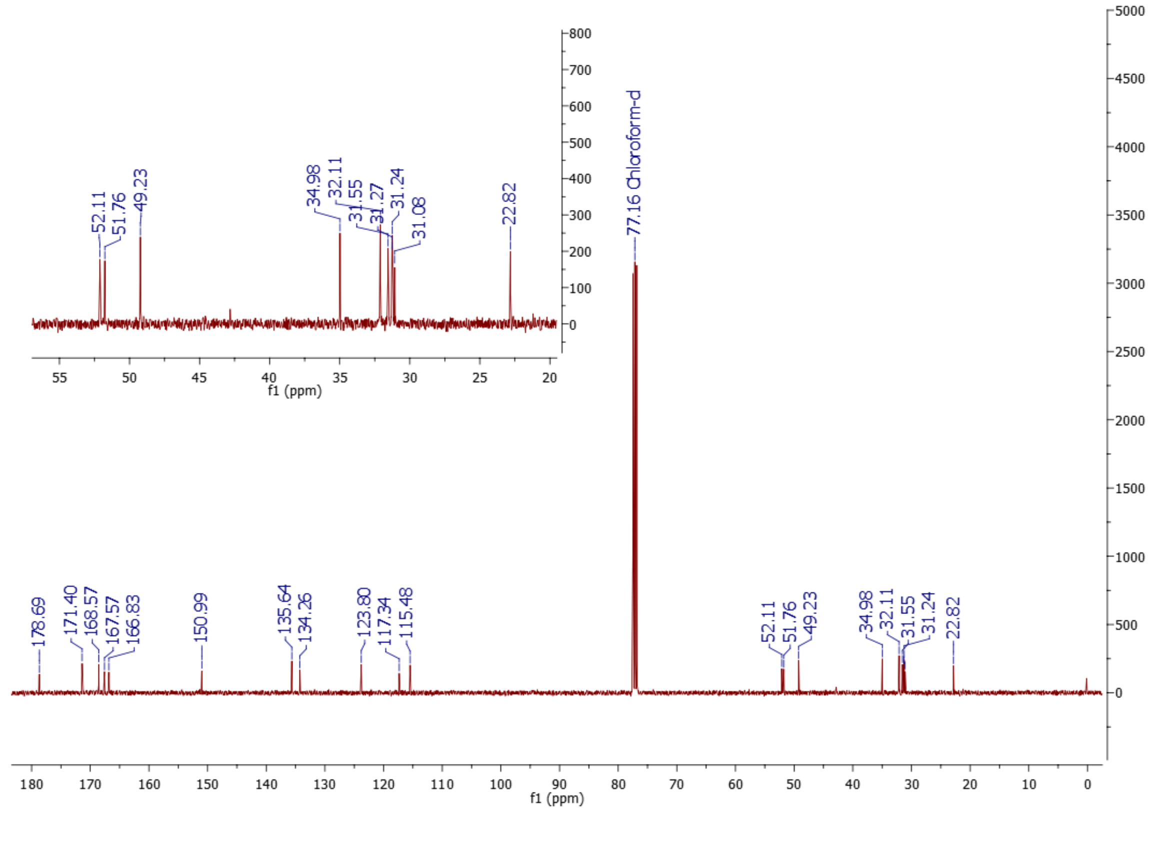

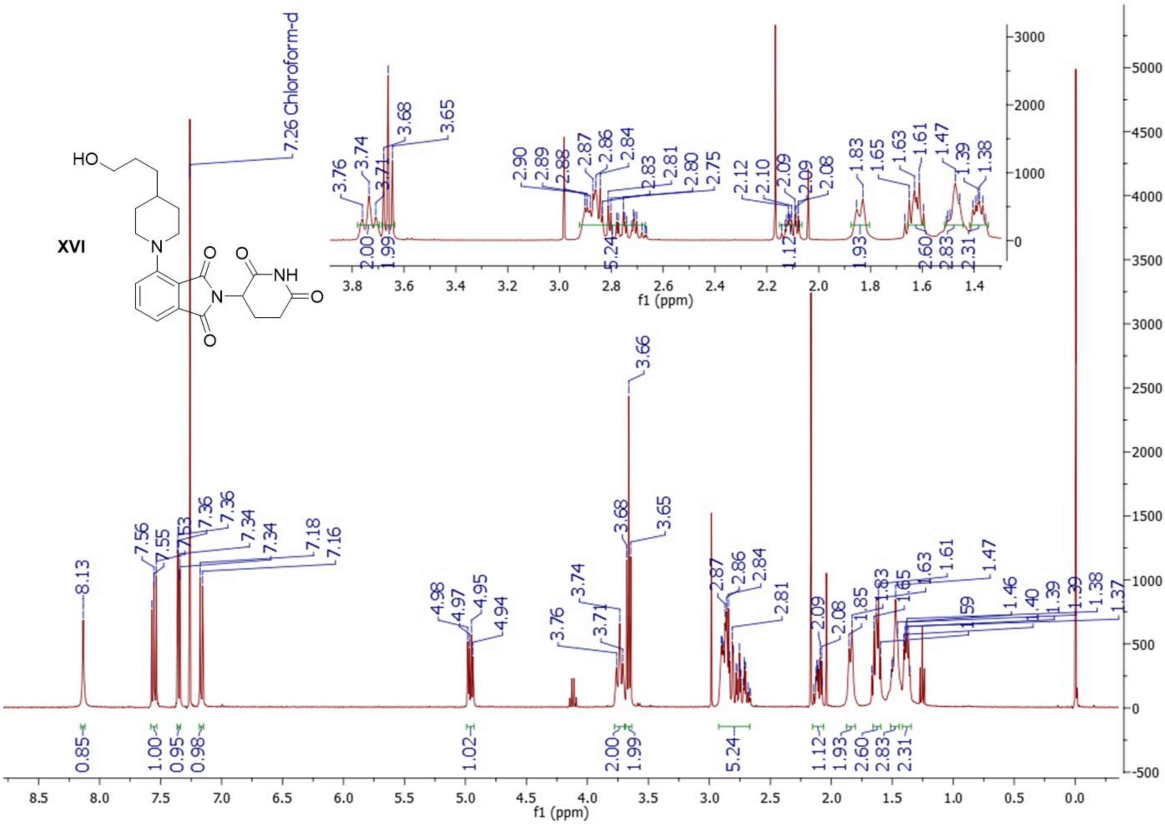
7.26 Chloroform-d
HO
N
O
NH
XVI
3.76 3.74 3.71 3.68 3.65
2.90 2.89 2.88 2.87 2.86 2.84 2.83 2.81 2.80 2.75
2.12 2.10 2.09 2.09 2.08
1.83 1.65 1.63 1.61 1.47 1.39 1.38
2.00 1.99 5.24 1.12 1.93 2.60 2.83 2.31
8.13
7.56 7.55 7.53 7.36 7.36 7.34 7.34 7.18 7.16
4.98 4.97 4.95 4.94
3.66
1.46 1.40 1.39 1.37
0.85 1.00 0.95 0.98 1.02
f1 (ppm)

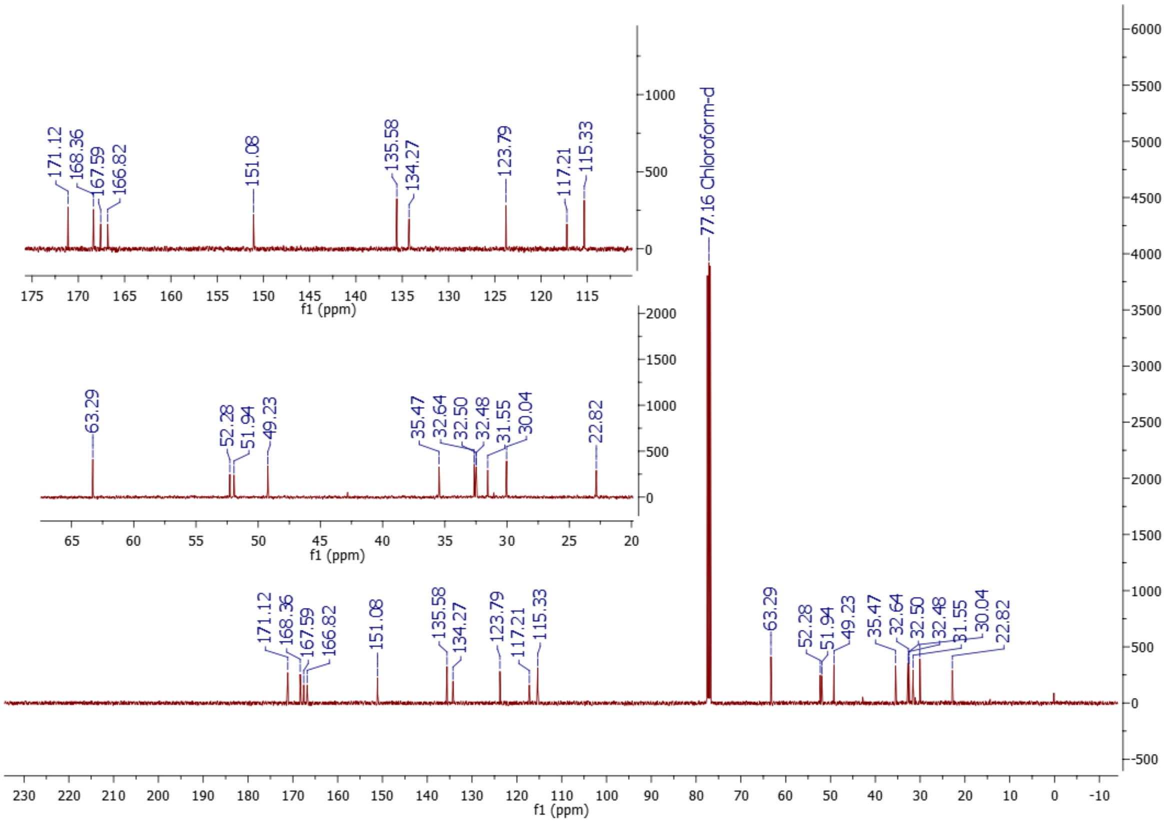
77.16 Chloroform-d
171.12 168.36 167.59 166.82
151.08
135.58 134.27
123.79
117.21 115.33
63.29
52.28 51.94 49.23
35.47 32.64 32.50 32.48 31.55 30.04
22.82
f1 (ppm)

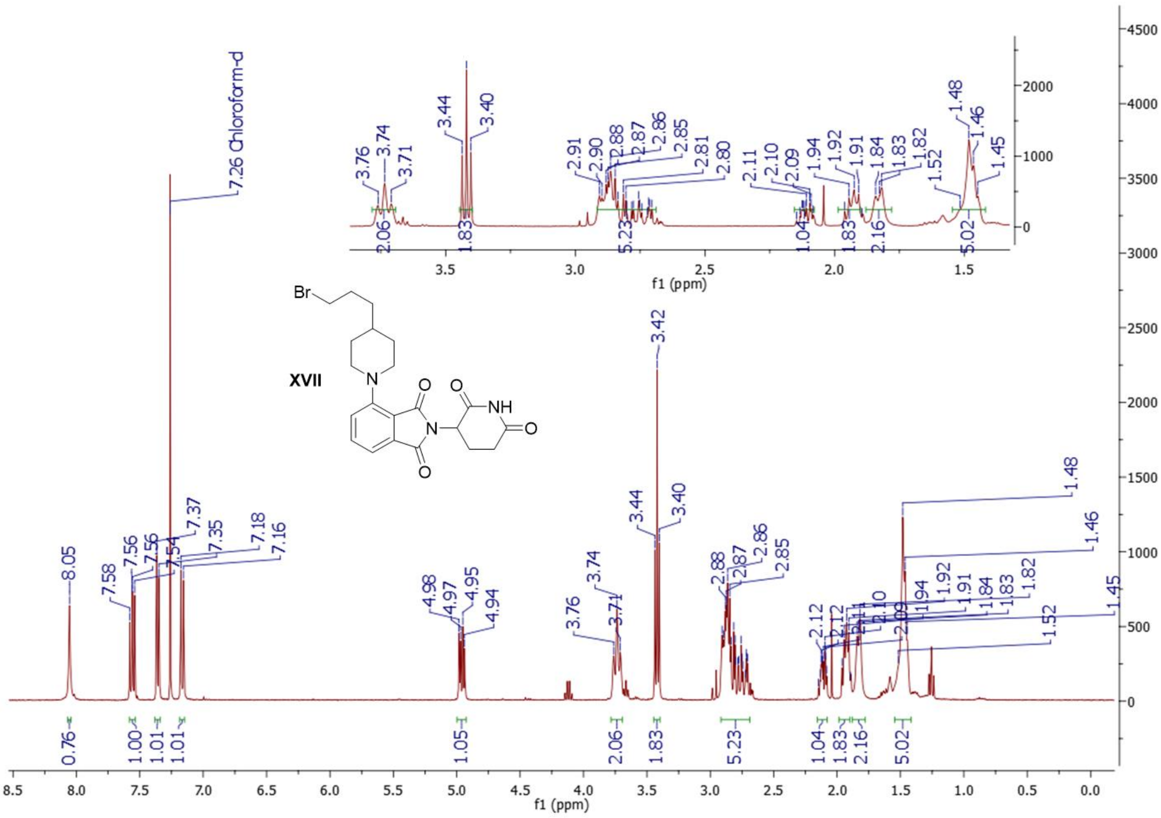
XVII
7.26 Chloroform-d
f1 (ppm)

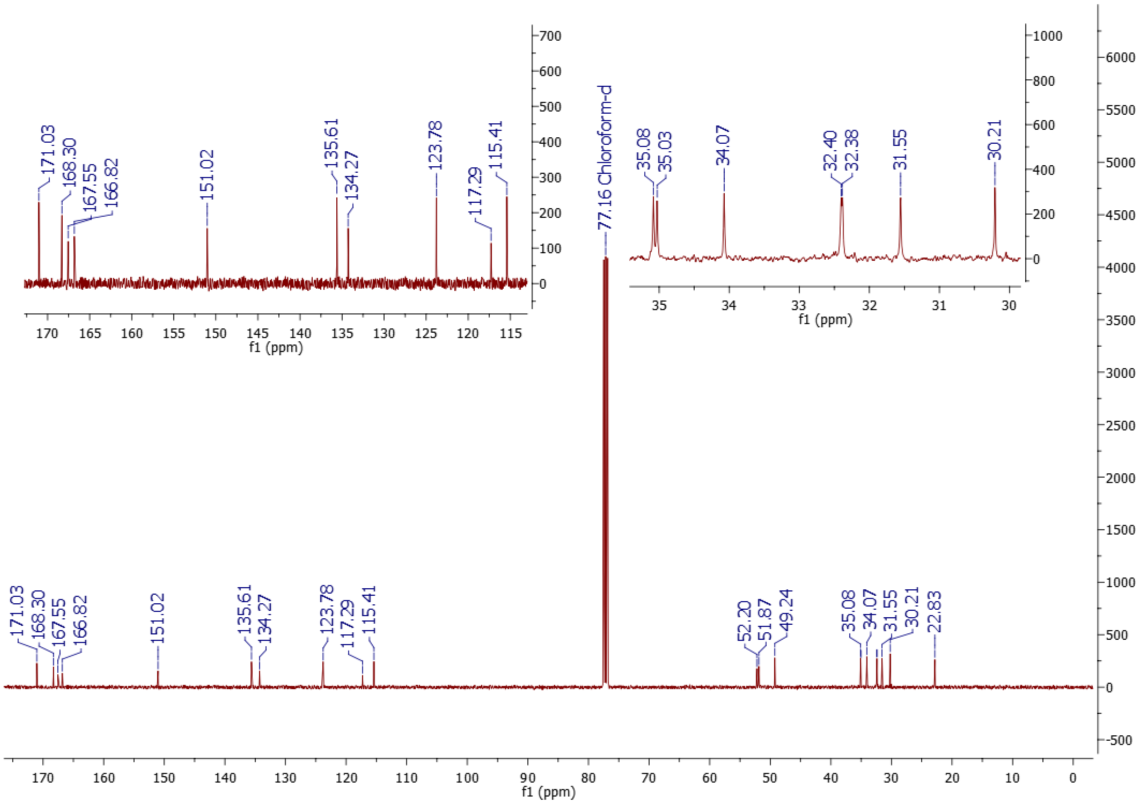
77.16 Chloroform-d
f1 (ppm)

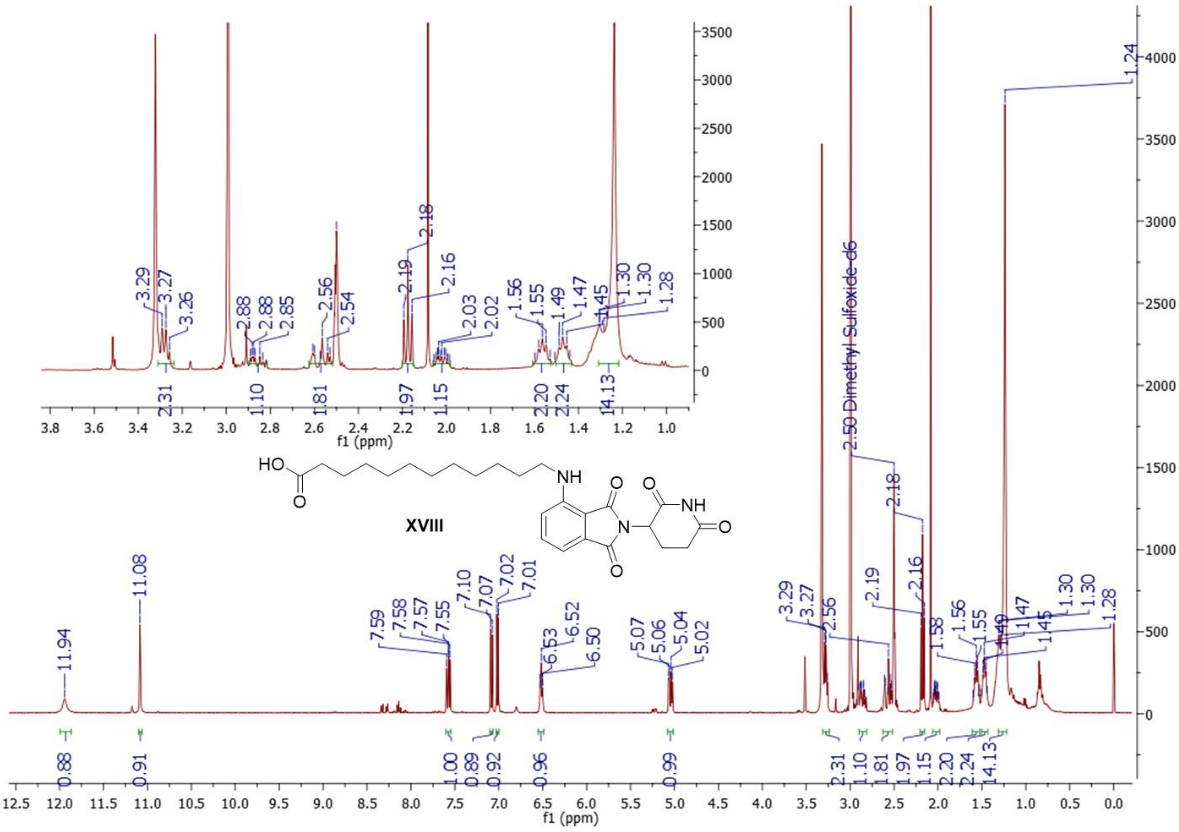
HO
O
NH
O
O
NH
N
O
O
XVIII
11.94
11.08
7.59
7.58
7.57
7.55
7.10
7.07
7.02
7.01
6.53
6.52
6.50
5.07
5.06
5.04
5.02
3.29
3.27
3.26
2.88
2.88
2.85
2.56
2.54
2.50 Dimethyl Sulfoxide-d6
2.19
2.18
2.16
2.03
2.02
1.58
1.56
1.55
1.49
1.47
1.45
1.30
1.30
1.28
1.24
0.88
0.91
1.00
0.89
0.92
0.96
0.99
2.31
1.10
1.81
1.97
1.15
2.20
2.24
14.13
f1 (ppm)

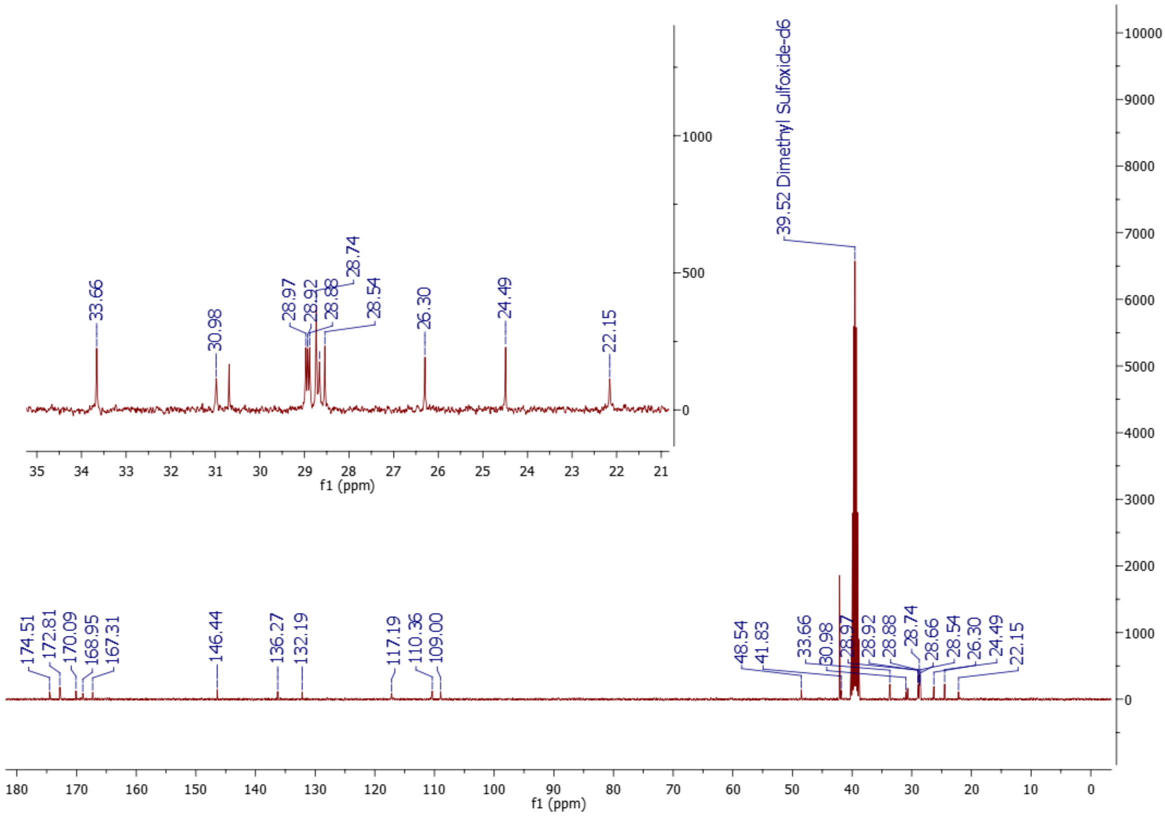
39.52 Dimethyl Sulfoxide-d6
174.51
172.81
170.09
168.95
167.31
146.44
136.27
132.19
117.19
110.36
109.00
48.54
41.83
33.66
30.98
28.97
28.92
28.88
28.74
28.66
28.54
26.30
24.49
22.15
f1 (ppm)

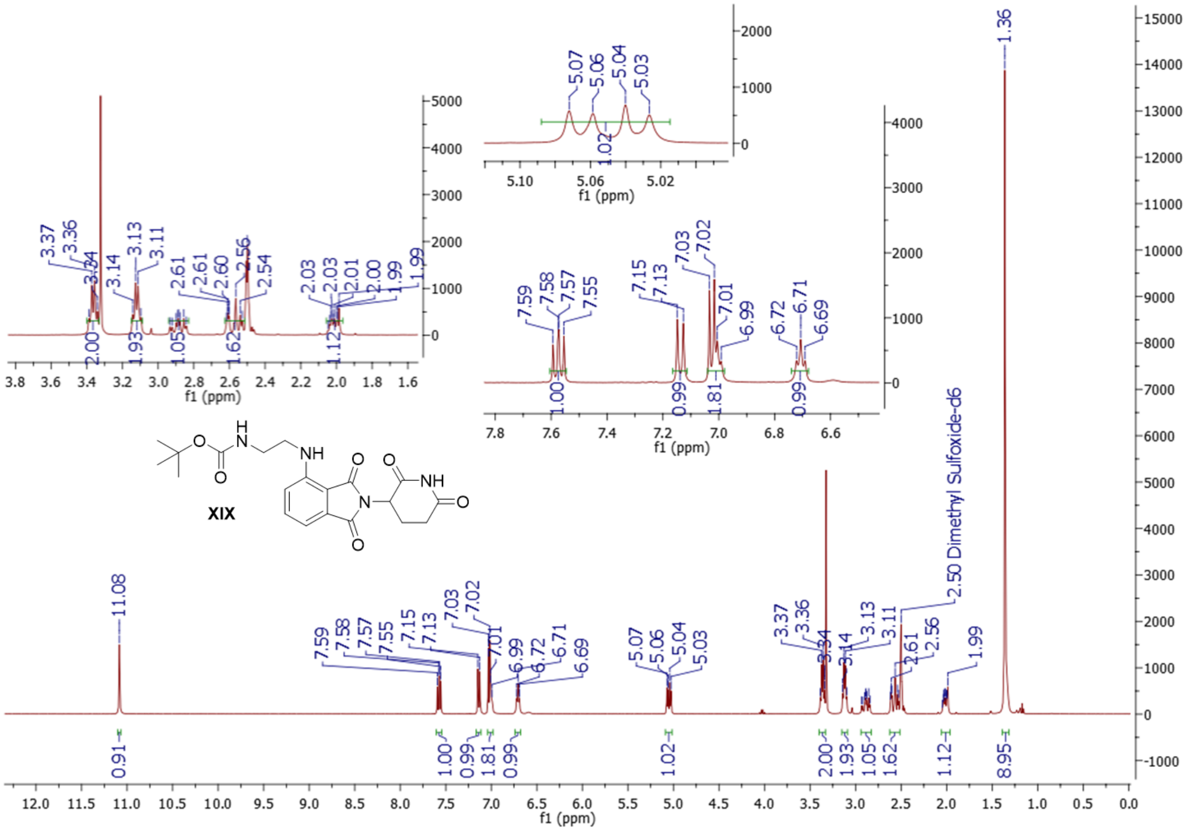

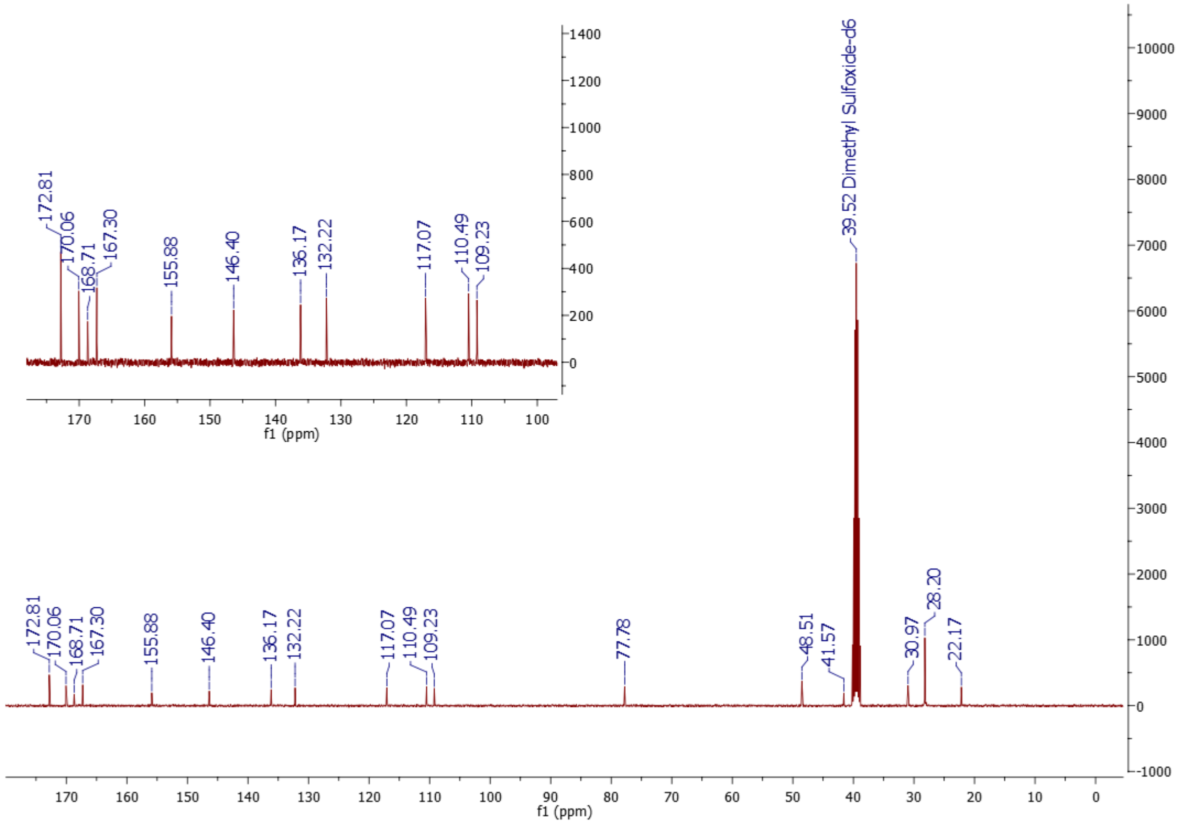

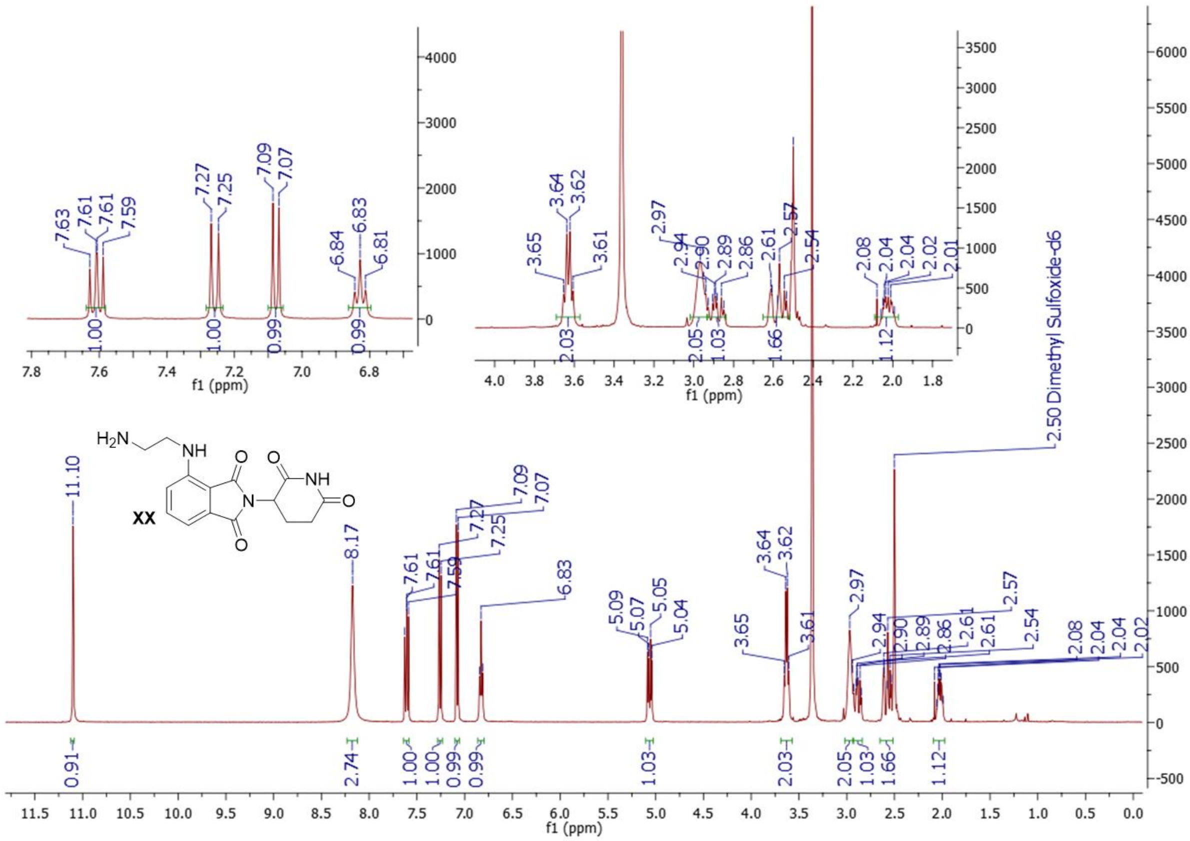
XX
11.10
8.17
7.61
7.61
7.59
7.27
7.25
7.09
7.07
6.83
5.09
5.07
5.05
5.04
3.65
3.64
3.62
3.61
2.97
2.94
2.90
2.89
2.86
2.61
2.61
2.57
2.54
2.08
2.04
2.04
2.02
2.50 Dimethyl Sulfoxide-d6
0.91
2.74
1.00
1.00
0.99
0.99
1.03
2.03
2.05
1.03
1.66
1.12
f1 (ppm)

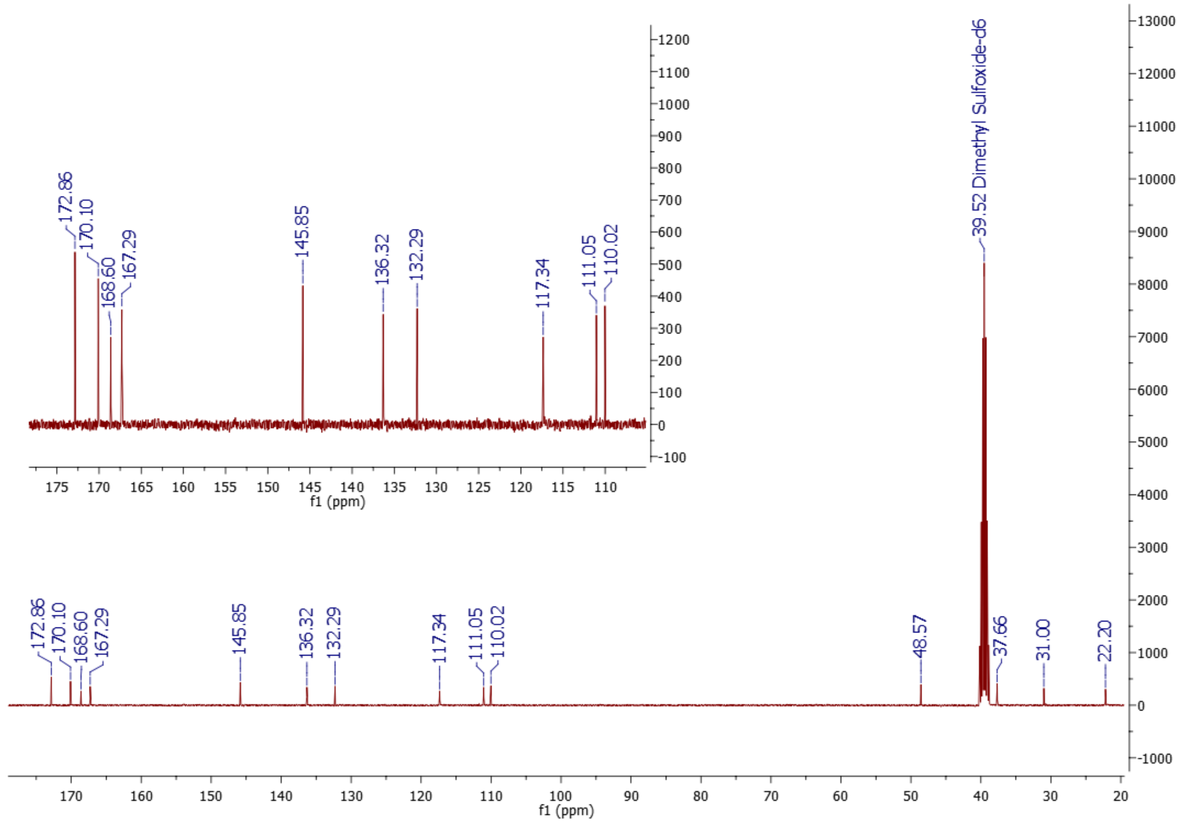
172.86
170.10
168.60
167.29
145.85
136.32
132.29
117.34
111.05
110.02
48.57
39.52 Dimethyl Sulfoxide-d6
37.66
31.00
22.20
f1 (ppm)

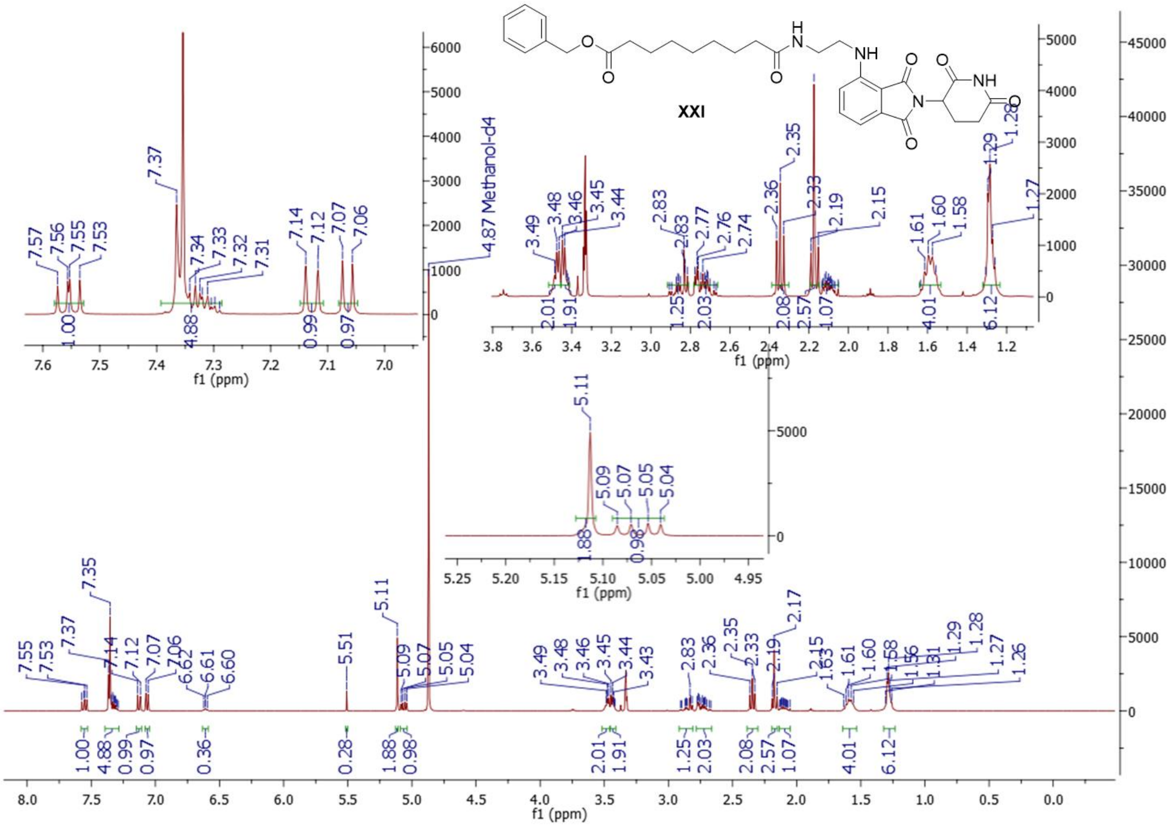
XXI

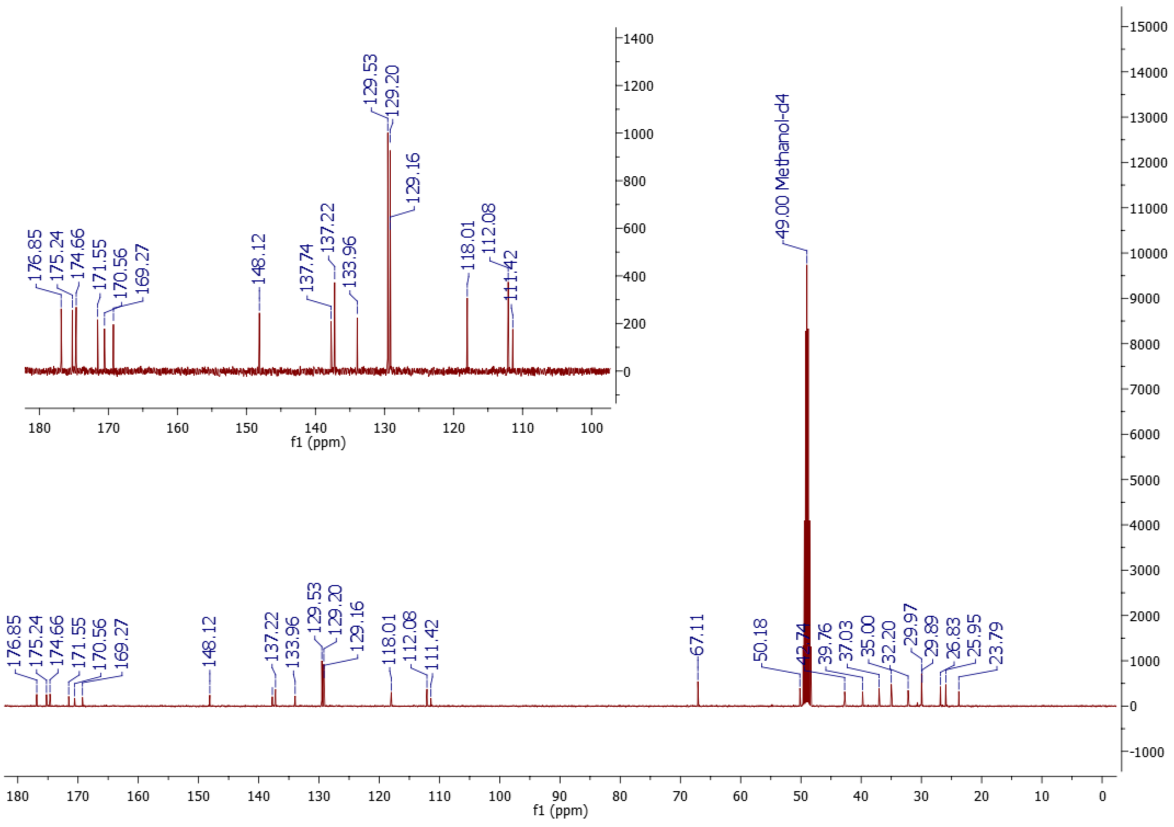

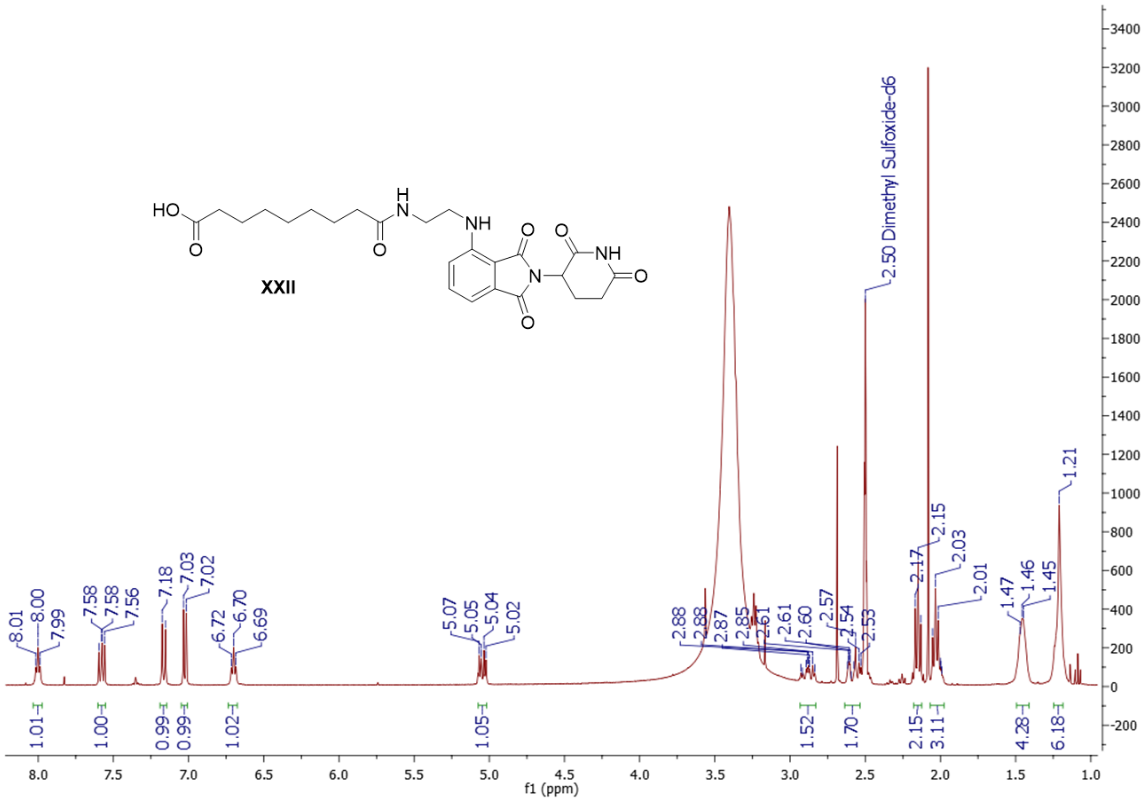
XXII
2.50 Dimethyl Sulfoxide-d6
f1 (ppm)

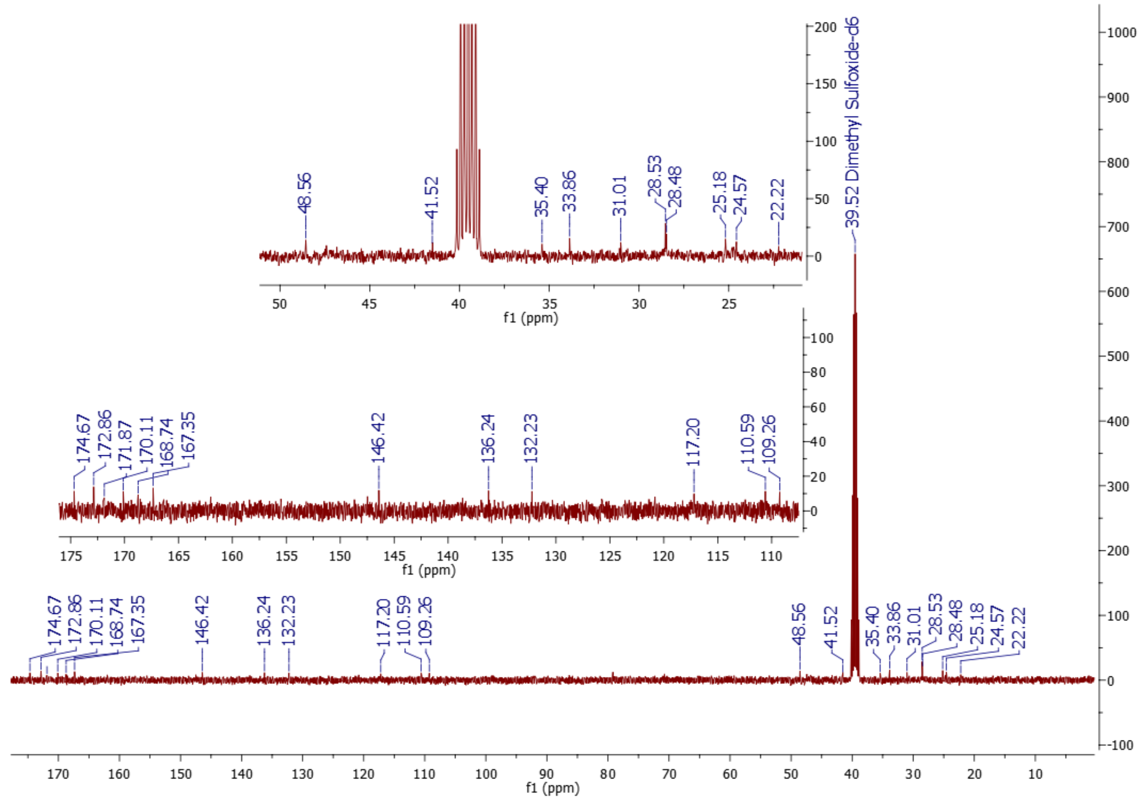
39.52 Dimethyl Sulfoxide-d6
f1 (ppm)

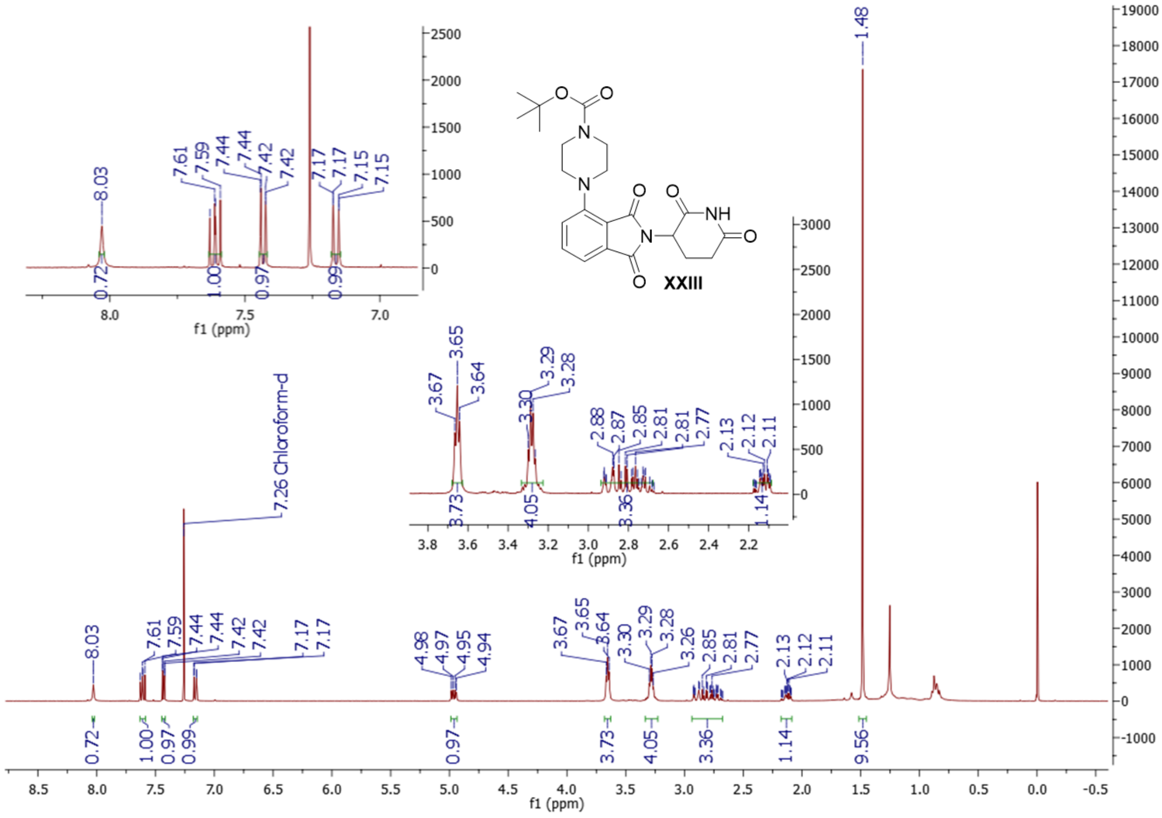
8.03
7.61
7.59
7.44
7.44
7.42
7.42
7.17
7.17
7.15
7.15
7.26 Chloroform-d
4.98
4.97
4.95
4.94
3.67
3.65
3.64
3.30
3.29
3.28
3.26
2.88
2.87
2.85
2.81
2.81
2.77
2.13
2.12
2.11
1.48
0.72
1.00
0.97
0.99
0.97
3.73
4.05
3.36
1.14
9.56
XXIII
O
NH
N
f1 (ppm)

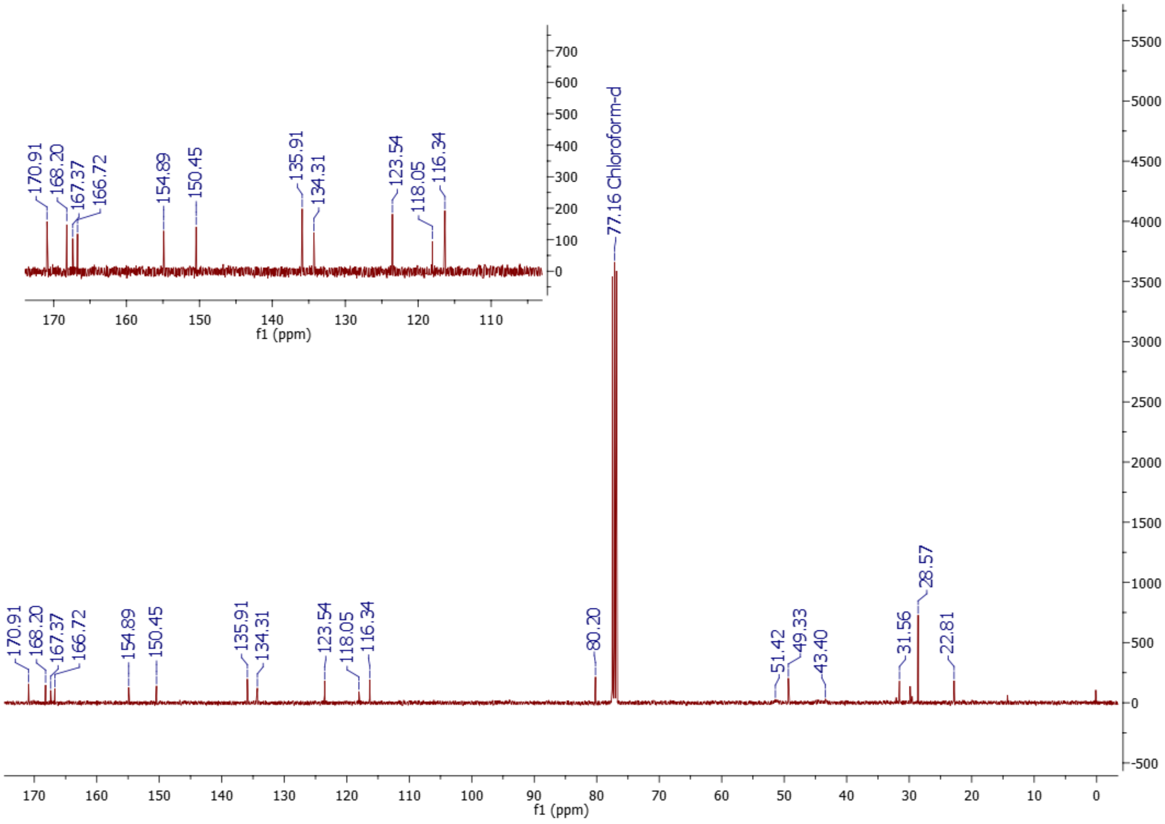
170.91
168.20
167.37
166.72
154.89
150.45
135.91
134.31
123.54
118.05
116.34
80.20
77.16 Chloroform-d
51.42
49.33
43.40
31.56
28.57
22.81
f1 (ppm)

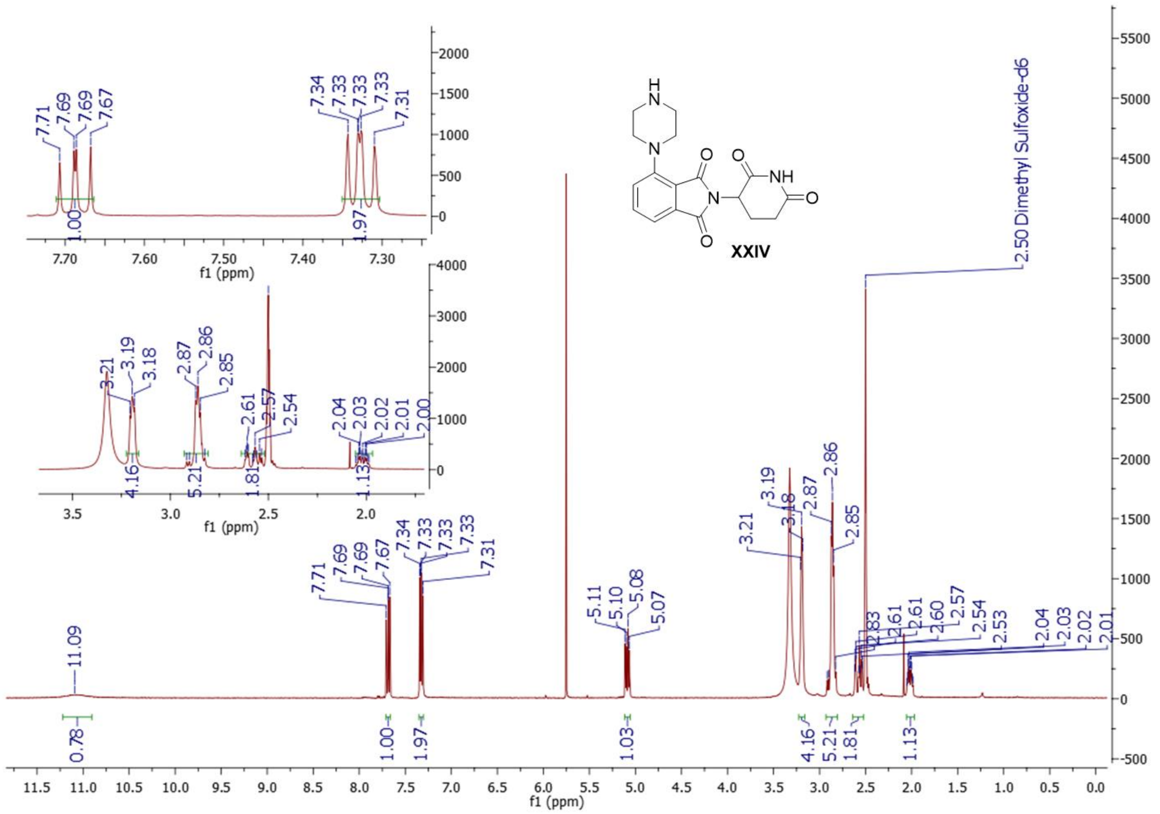
XXIV
2.50 Dimethyl Sulfoxide-d6
11.09
7.71 7.69 7.69 7.67 7.34 7.33 7.33 7.33 7.31
5.11 5.10 5.08 5.07
3.21 3.19 3.18 2.87 2.86 2.85
2.83 2.61 2.61 2.60 2.57 2.54 2.53
2.04 2.03 2.02 2.01
0.78 1.00 1.97 1.03 4.16 5.21 1.81 1.13
f1 (ppm)

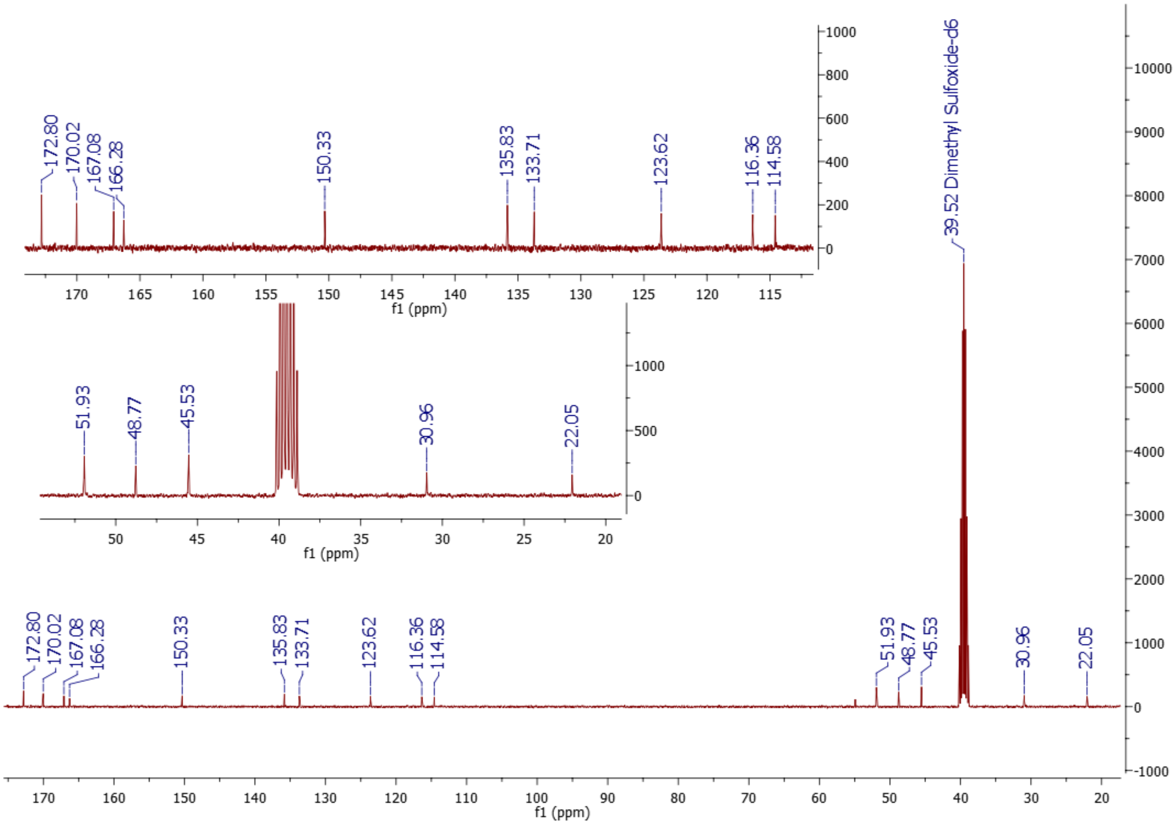
39.52 Dimethyl Sulfoxide-d6
172.80 170.02 167.08 166.28 150.33 135.83 133.71 123.62 116.36 114.58
51.93 48.77 45.53 30.96 22.05
f1 (ppm)

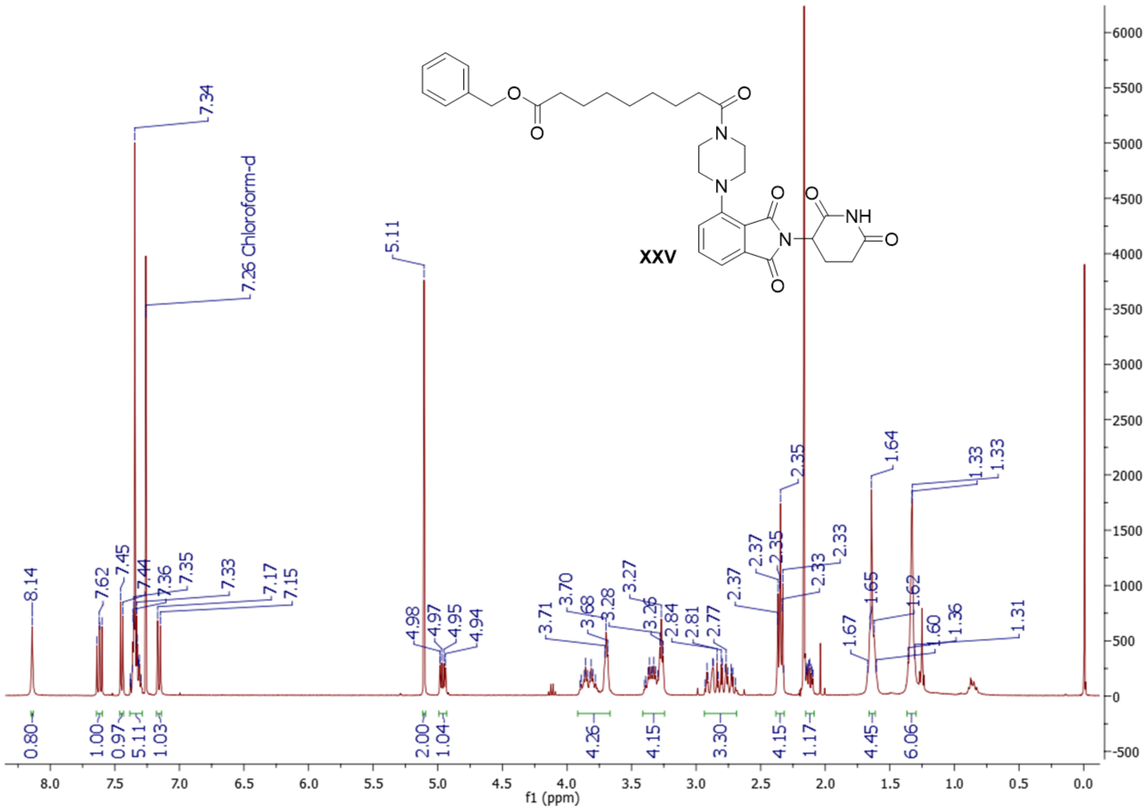

XXV
O
NH
N
7.34
7.26 Chloroform-d
5.11
8.14
7.62
7.45
7.44
7.36
7.35
7.33
7.17
7.15
4.98
4.97
4.95
4.94
3.71
3.70
3.68
3.28
3.27
3.26
2.84
2.81
2.77
2.37
2.37
2.35
2.35
2.33
2.33
1.67
1.65
1.64
1.62
1.60
1.36
1.33
1.33
1.31
0.80
1.00
0.97
5.11
1.03
2.00
1.04
4.26
4.15
3.30
4.15
1.17
4.45
6.06
f1 (ppm)

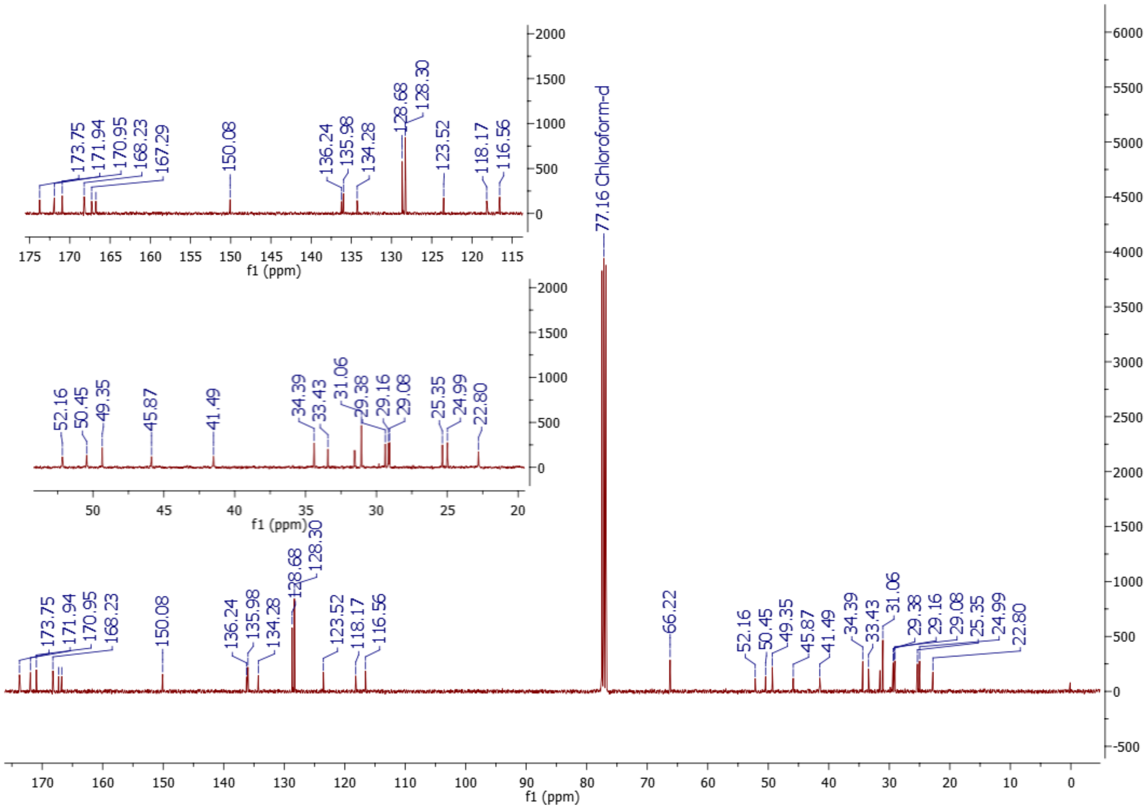

77.16 Chloroform-d
173.75
171.94
170.95
168.23
167.29
150.08
136.24
135.98
134.28
128.68
128.30
123.52
118.17
116.56
66.22
52.16
50.45
49.35
45.87
41.49
34.39
33.43
31.06
29.38
29.16
29.08
25.35
24.99
22.80
f1 (ppm)

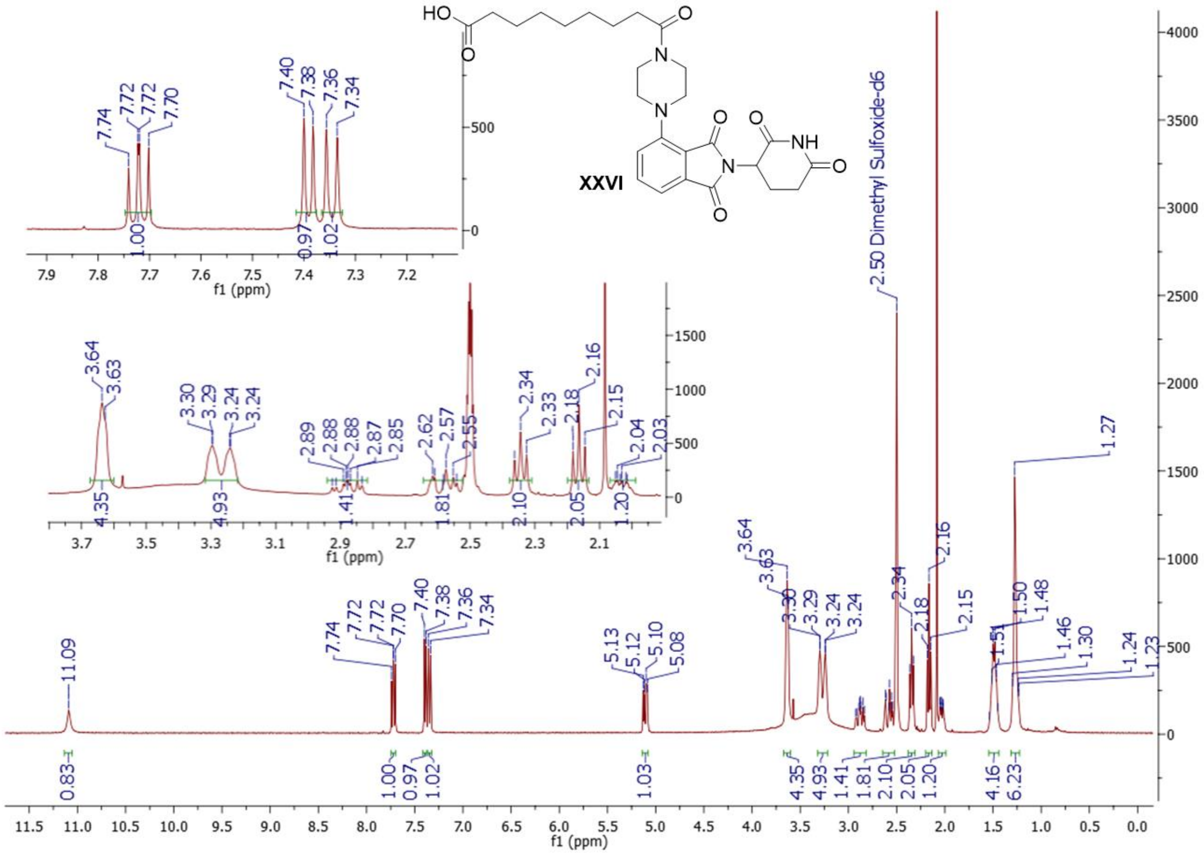
XXVI
HO
O
N
NH
2.50 Dimethyl Sulfoxide-d6
11.09
7.74 7.72 7.72 7.70 7.40 7.38 7.36 7.34
5.13 5.12 5.10 5.08
3.64 3.63 3.30 3.29 3.24 3.24
2.89 2.88 2.88 2.87 2.85 2.62 2.57 2.55 2.34 2.33 2.18 2.16 2.15 2.04 2.03
1.51 1.50 1.48 1.46 1.30 1.27 1.24 1.23
0.83 1.00 0.97 1.02 1.03 4.35 4.93 1.41 1.81 2.10 2.05 1.20 4.16 6.23
f1 (ppm)

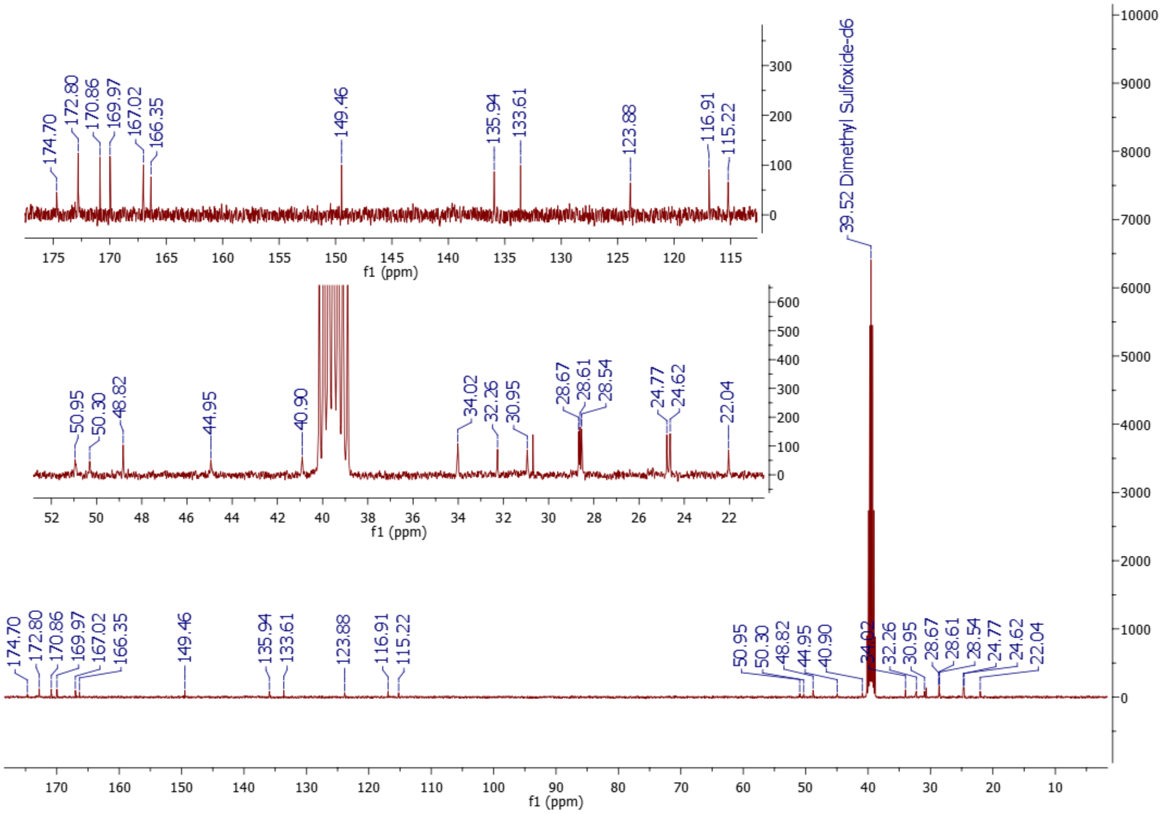
39.52 Dimethyl Sulfoxide-d6
174.70 172.80 170.86 169.97 167.02 166.35 149.46 135.94 133.61 123.88 116.91 115.22
50.95 50.30 48.82 44.95 40.90 34.02 32.26 30.95 28.67 28.61 28.54 24.77 24.62 22.04
f1 (ppm)

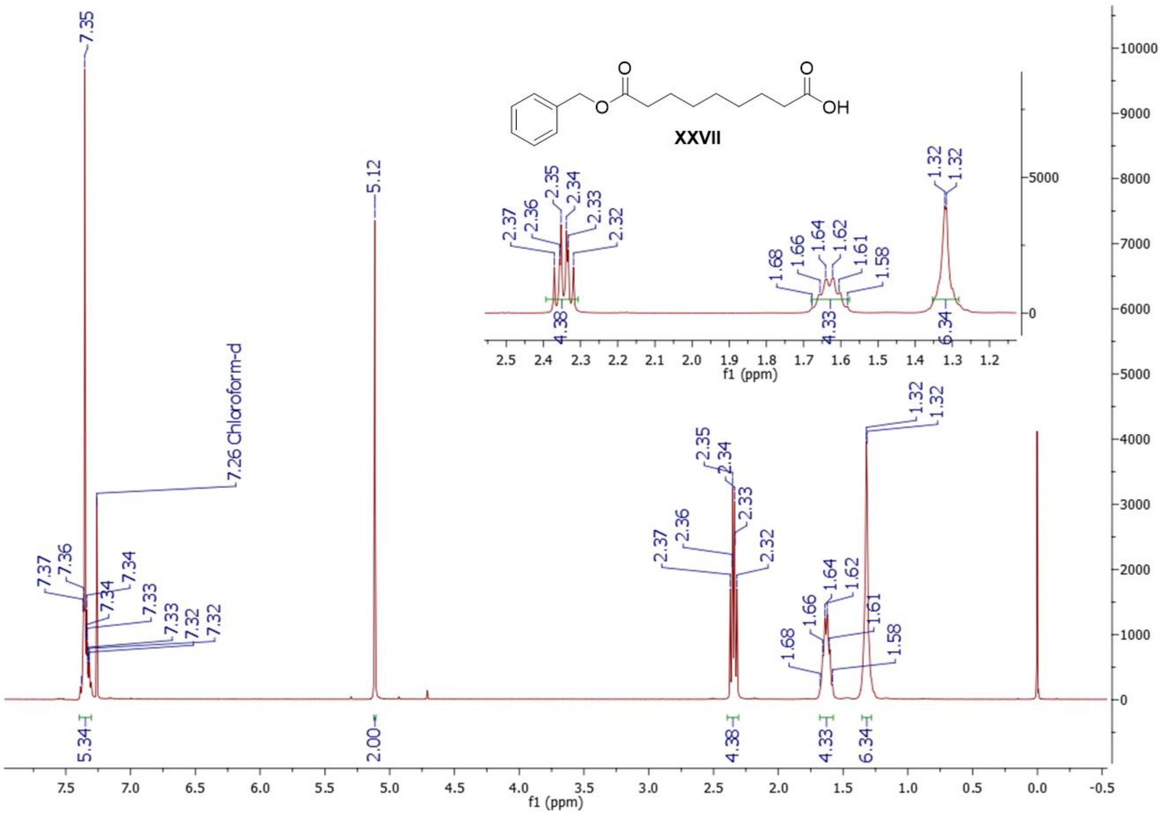
XXVII
OH
O
7.35
5.12
7.26 Chloroform-d
2.37
2.36
2.35
2.34
2.33
2.32
1.68
1.66
1.64
1.62
1.61
1.58
1.32
1.32
5.34
2.00
4.38
4.33
6.34
f1 (ppm)

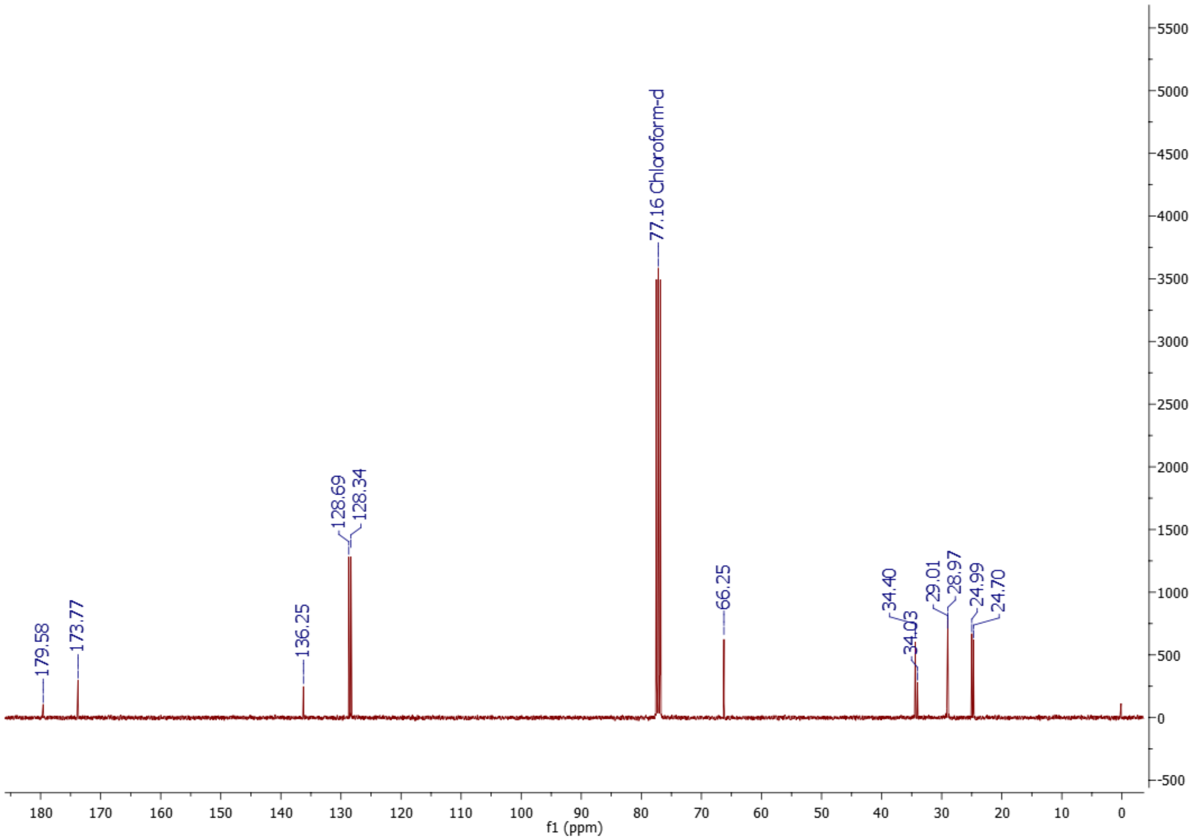
179.58
173.77
136.25
128.69
128.34
77.16 Chloroform-d
66.25
34.40
34.03
29.01
28.97
24.99
24.70
f1 (ppm)

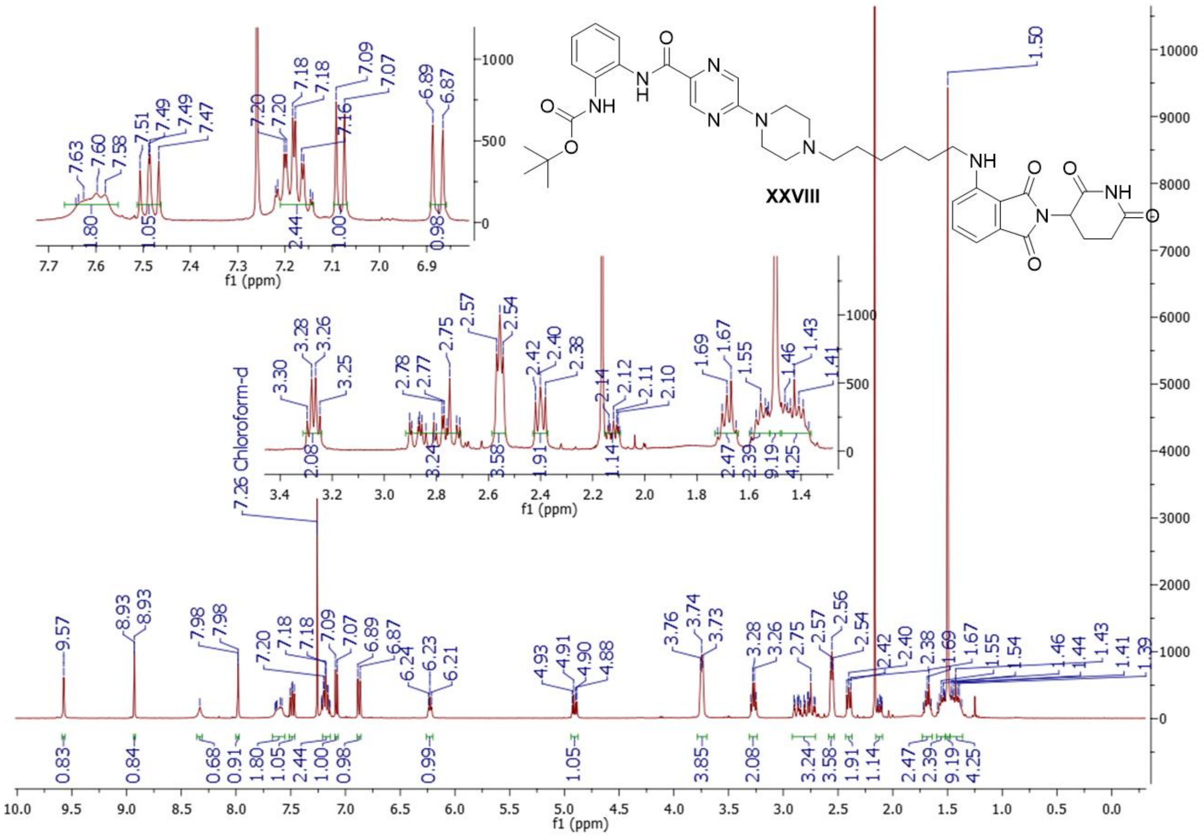

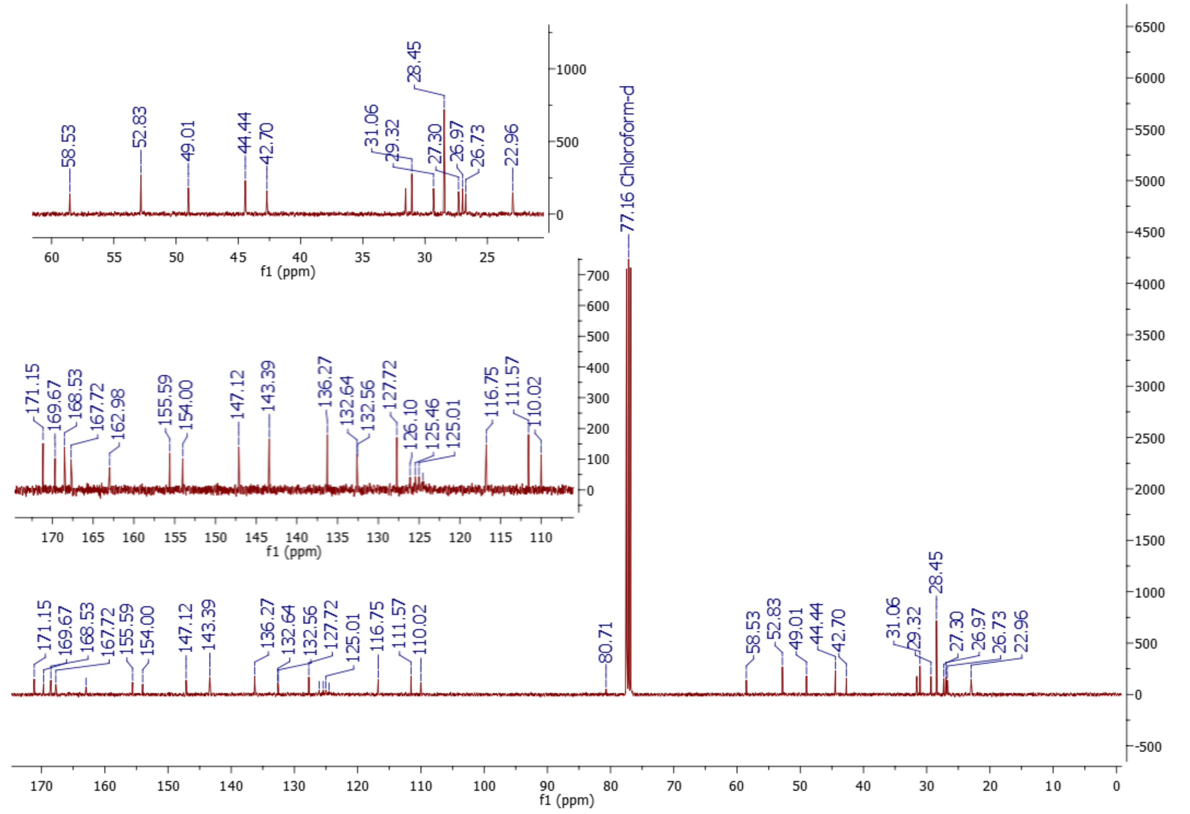

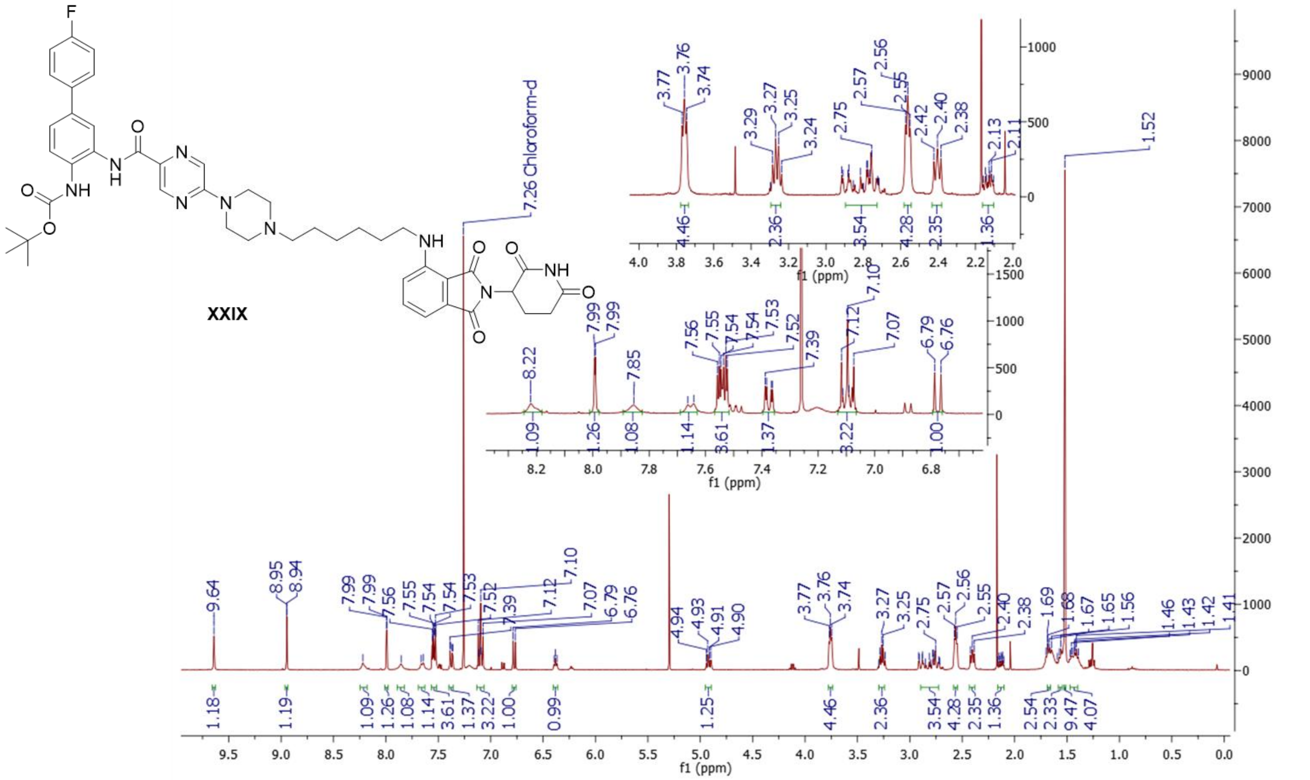

XXIX

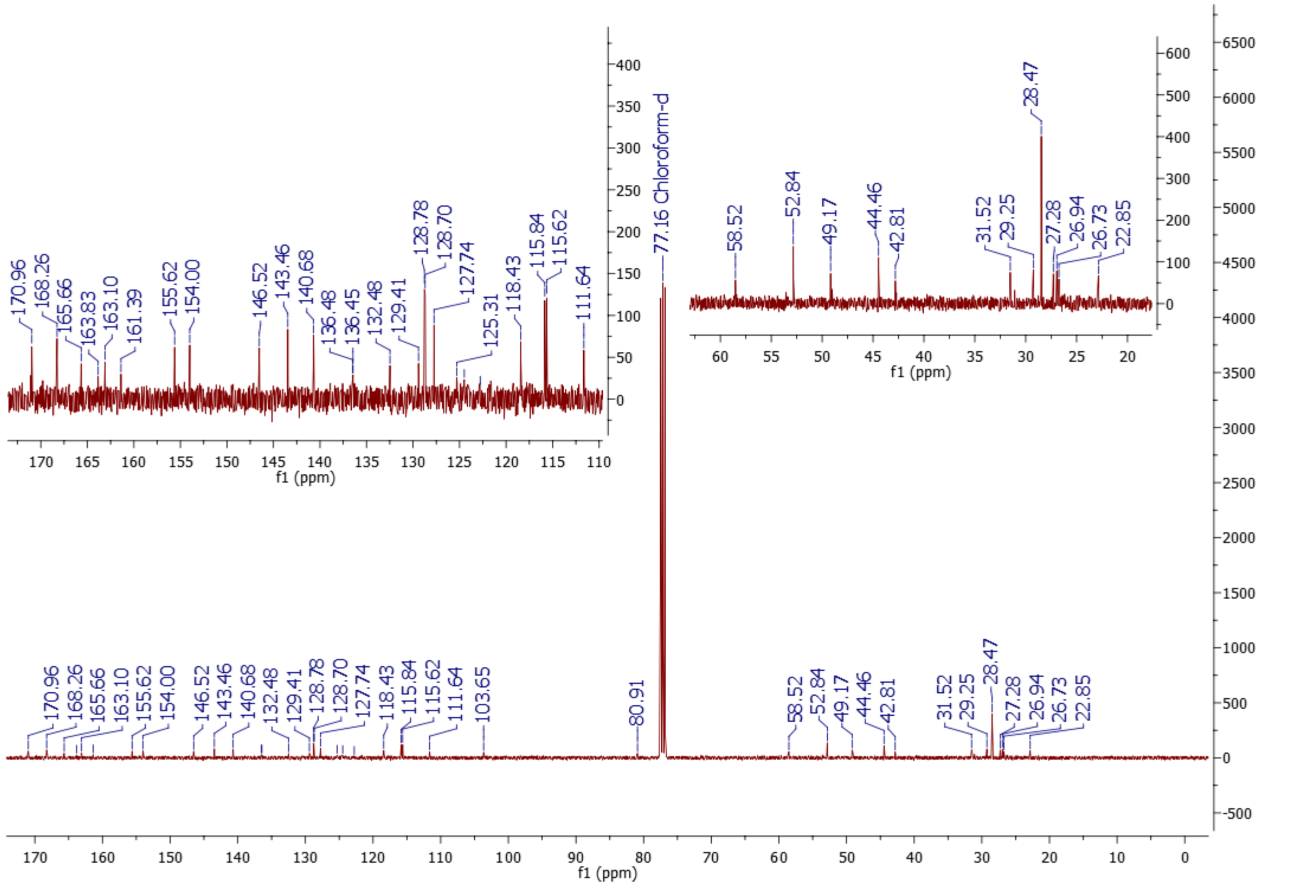

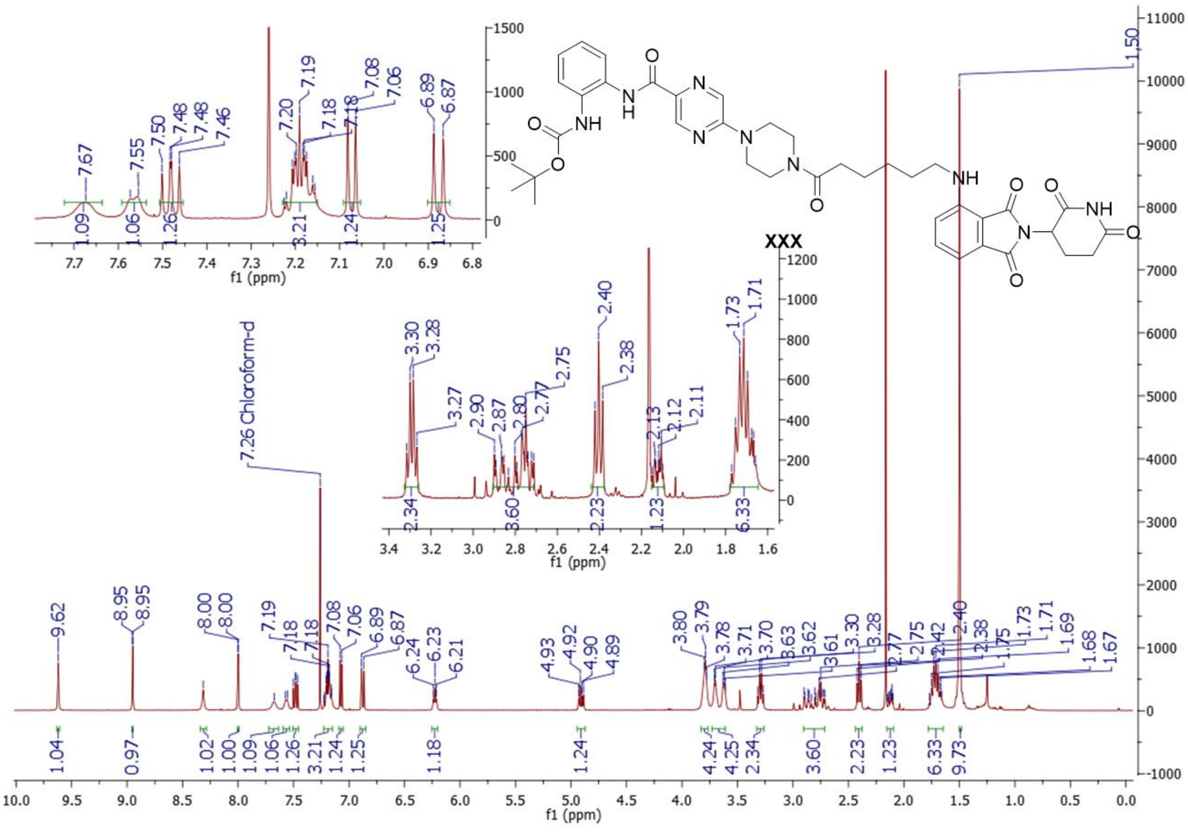

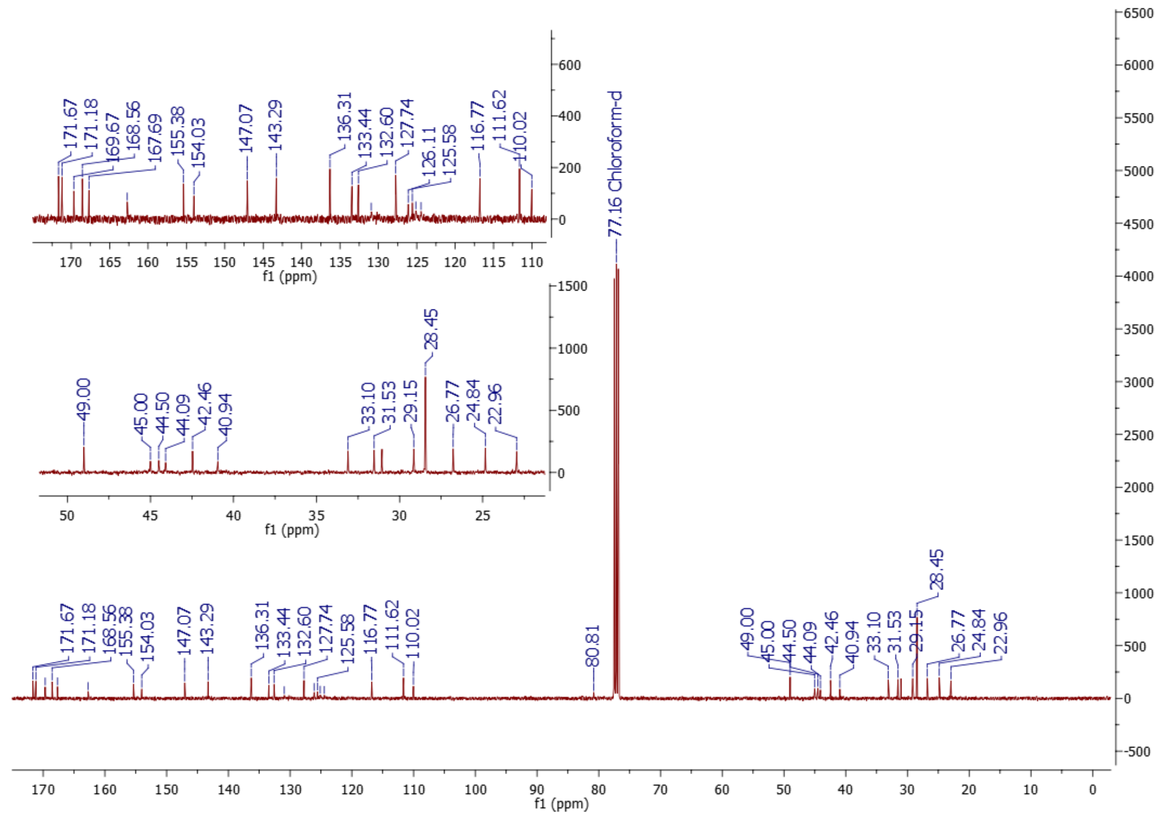

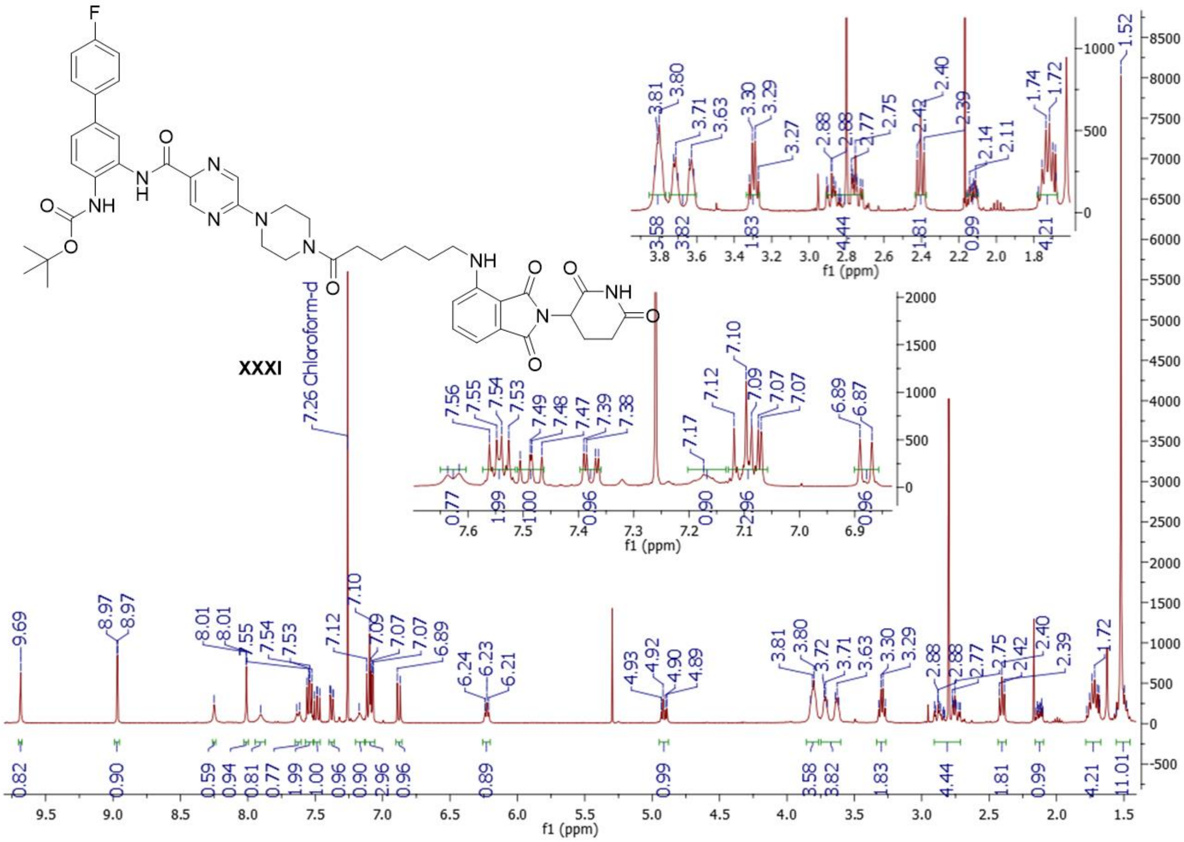
XXXI
7.26 Chloroform-d
f1 (ppm)

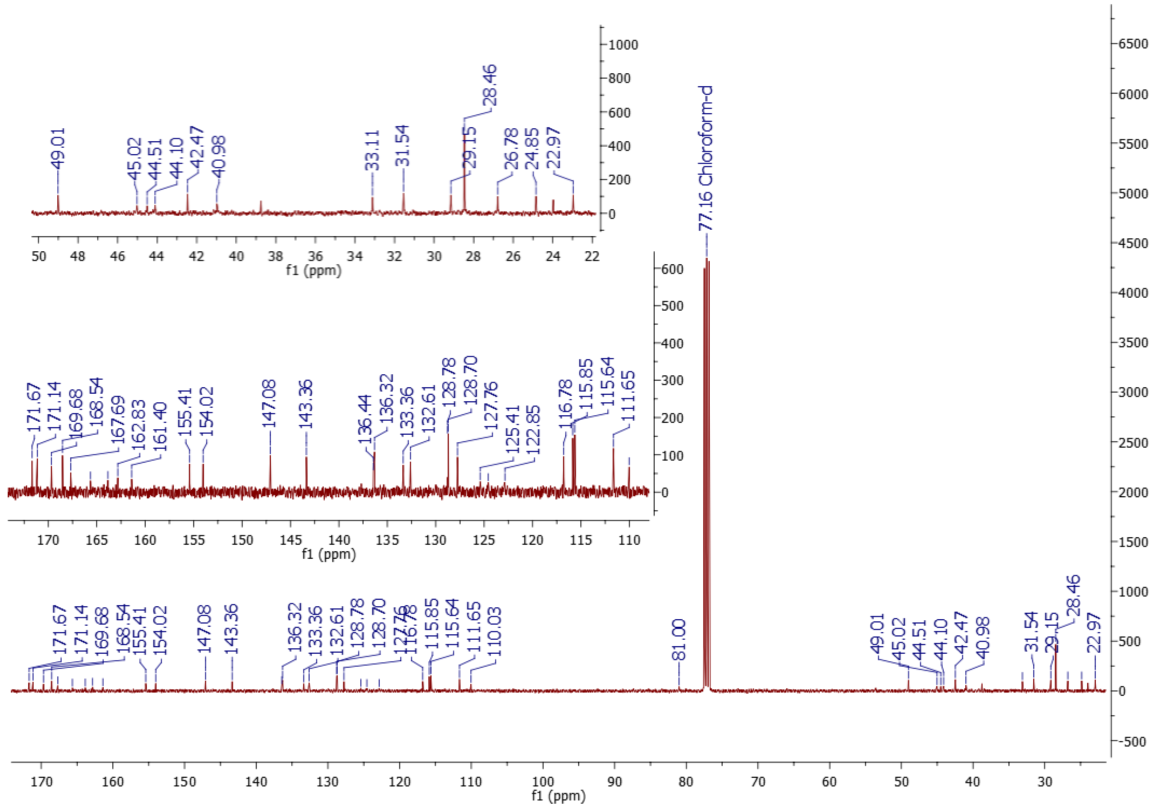
77.16 Chloroform-d
f1 (ppm)

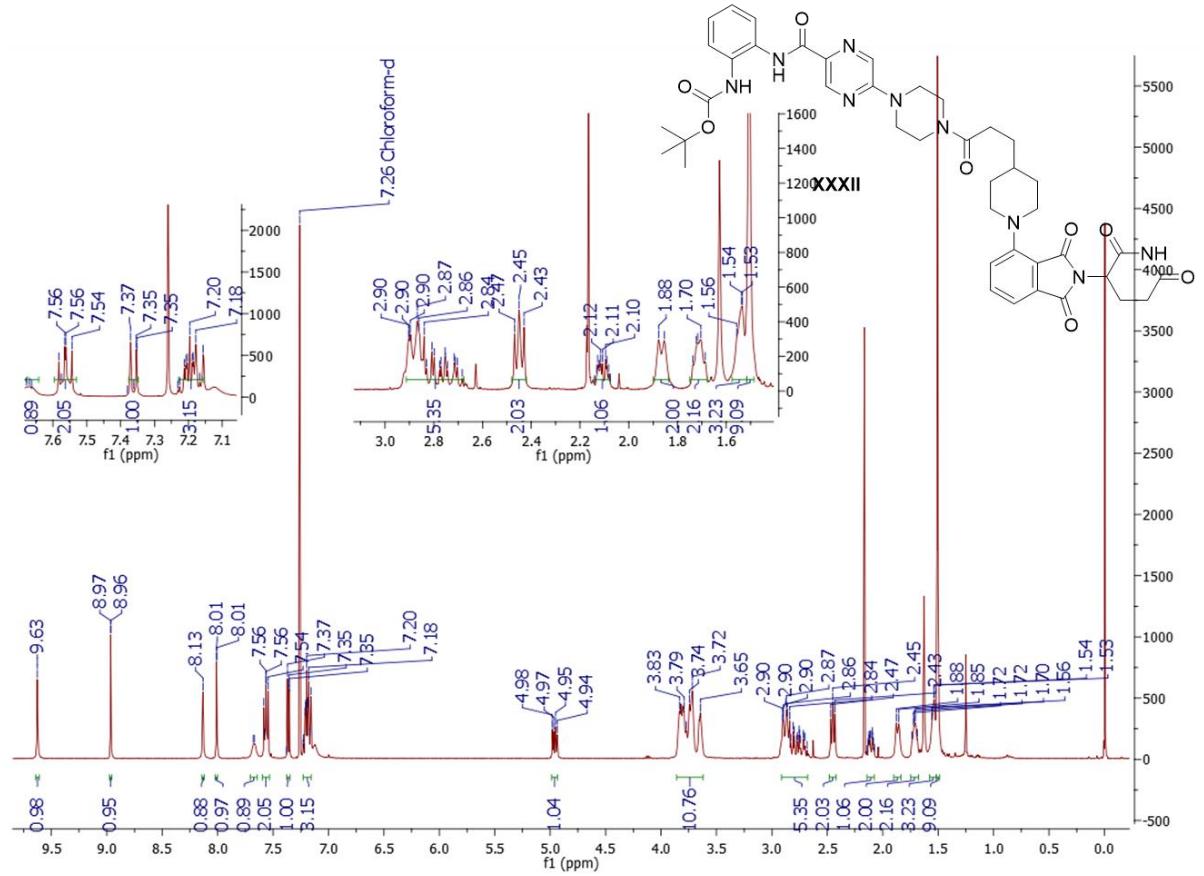
XXXII
7.26 Chloroform-d
9.63
8.97
8.96
8.13
8.01
8.01
7.56
7.56
7.54
7.37
7.35
7.35
7.20
7.18
4.98
4.97
4.95
4.94
3.83
3.79
3.74
3.72
3.65
2.90
2.90
2.90
2.87
2.86
2.84
2.47
2.45
2.43
2.12
2.11
2.10
1.88
1.85
1.72
1.72
1.70
1.56
1.54
1.53
0.98
0.95
0.88
0.97
0.89
2.05
1.00
3.15
1.04
10.76
5.35
2.03
1.06
2.00
2.16
3.23
9.09
f1 (ppm)

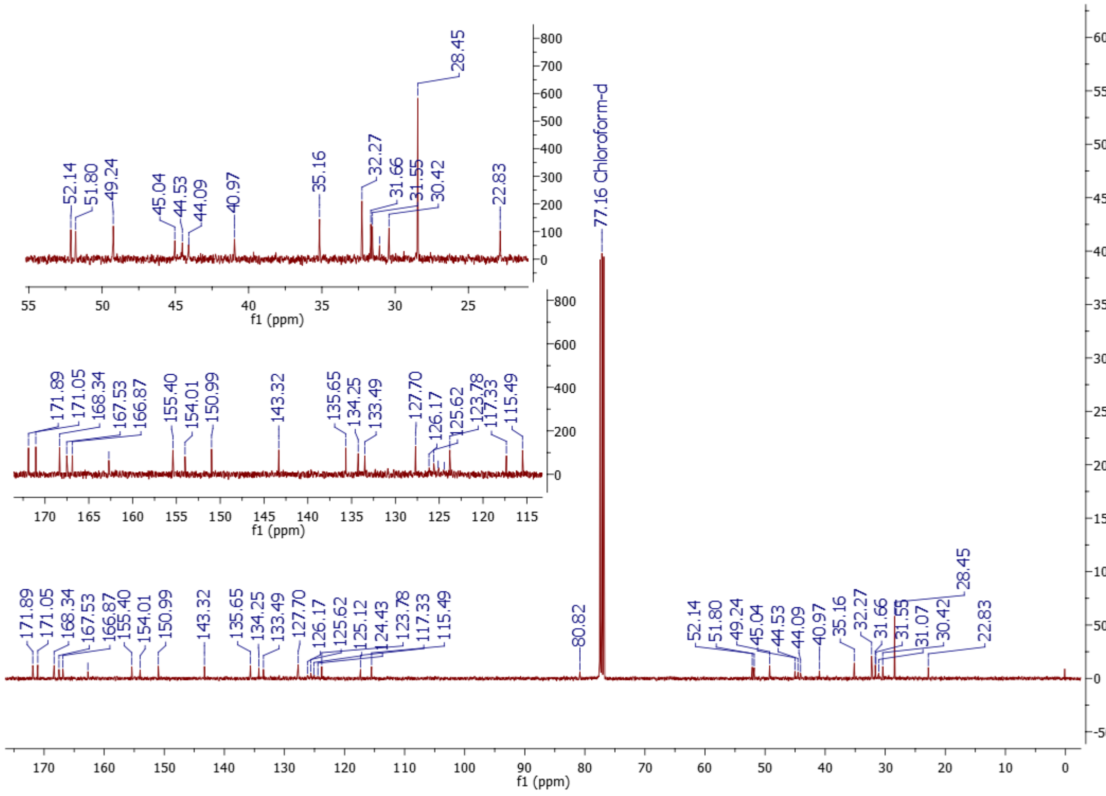
77.16 Chloroform-d
171.89
171.05
168.34
167.53
166.87
155.40
154.01
150.99
143.32
135.65
134.25
133.49
127.70
126.17
125.62
125.12
124.43
123.78
117.33
115.49
80.82
52.14
51.80
49.24
45.04
44.53
44.09
40.97
35.16
32.27
31.66
31.55
31.07
30.42
28.45
22.83
f1 (ppm)

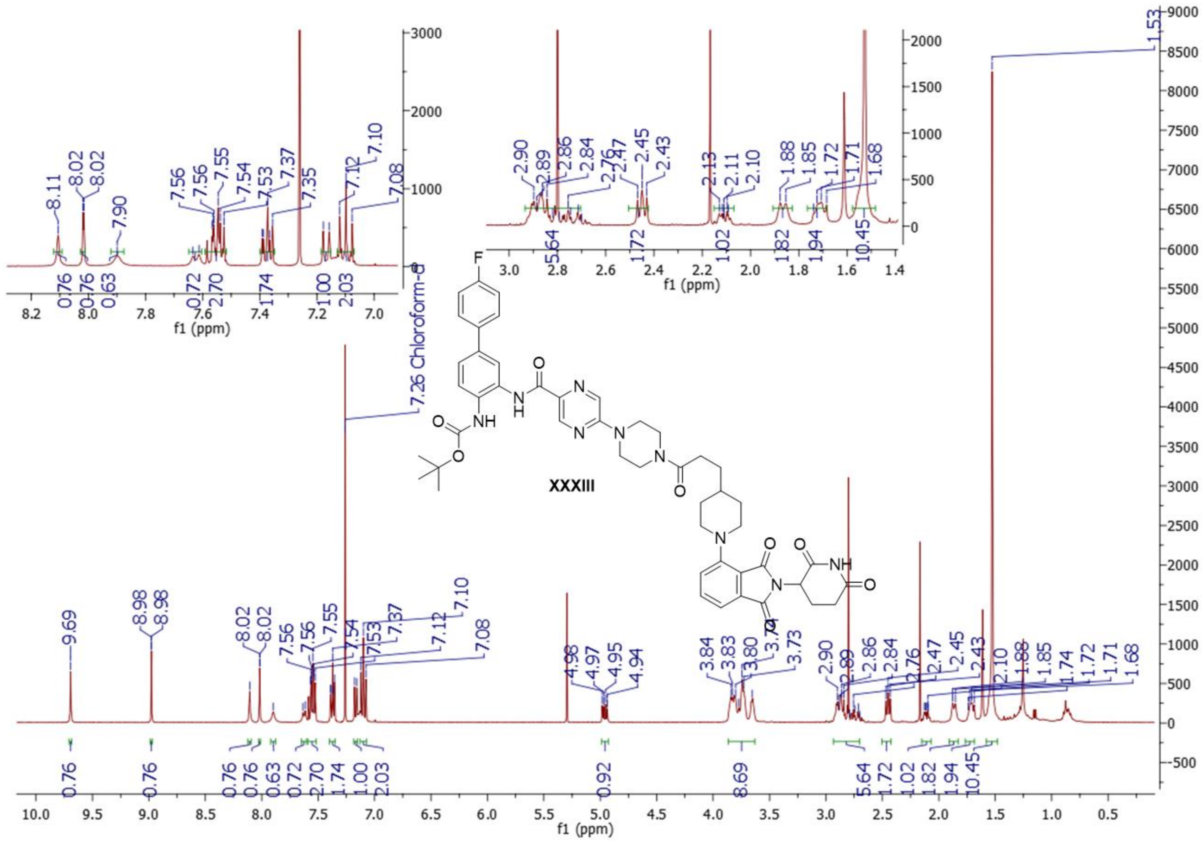
XXXIII
7.26 Chloroform-d
f1 (ppm)

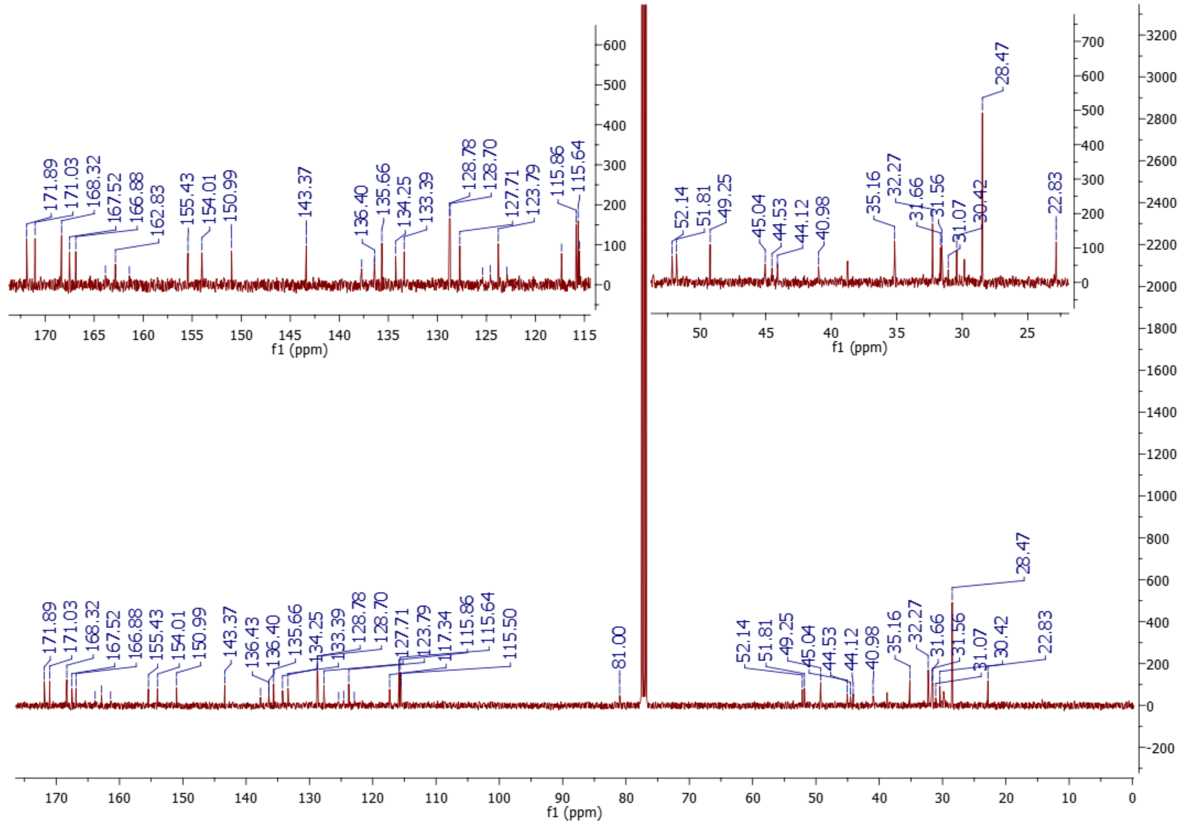
f1 (ppm)

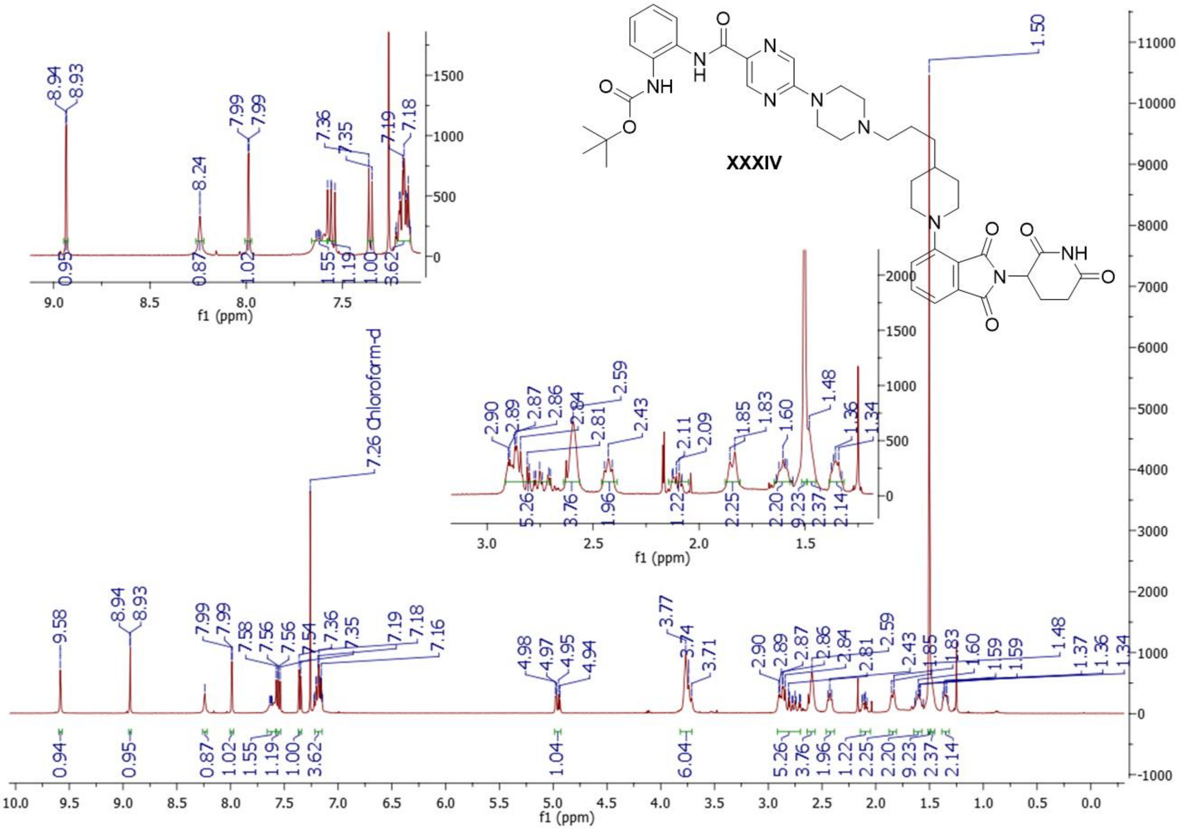
XXXIV
7.26 Chloroform-d
9.58
8.94
8.93
7.99
7.99
7.58
7.56
7.56
7.54
7.36
7.35
7.19
7.18
7.16
4.98
4.97
4.95
4.94
3.77
3.74
3.71
2.90
2.89
2.87
2.86
2.84
2.81
2.59
2.43
1.85
1.83
1.60
1.59
1.59
1.50
1.48
1.37
1.36
1.34
0.94
0.95
0.87
1.02
1.55
1.19
1.00
3.62
1.04
6.04
5.26
3.76
1.96
1.22
2.25
2.20
9.23
2.37
2.14
f1 (ppm)

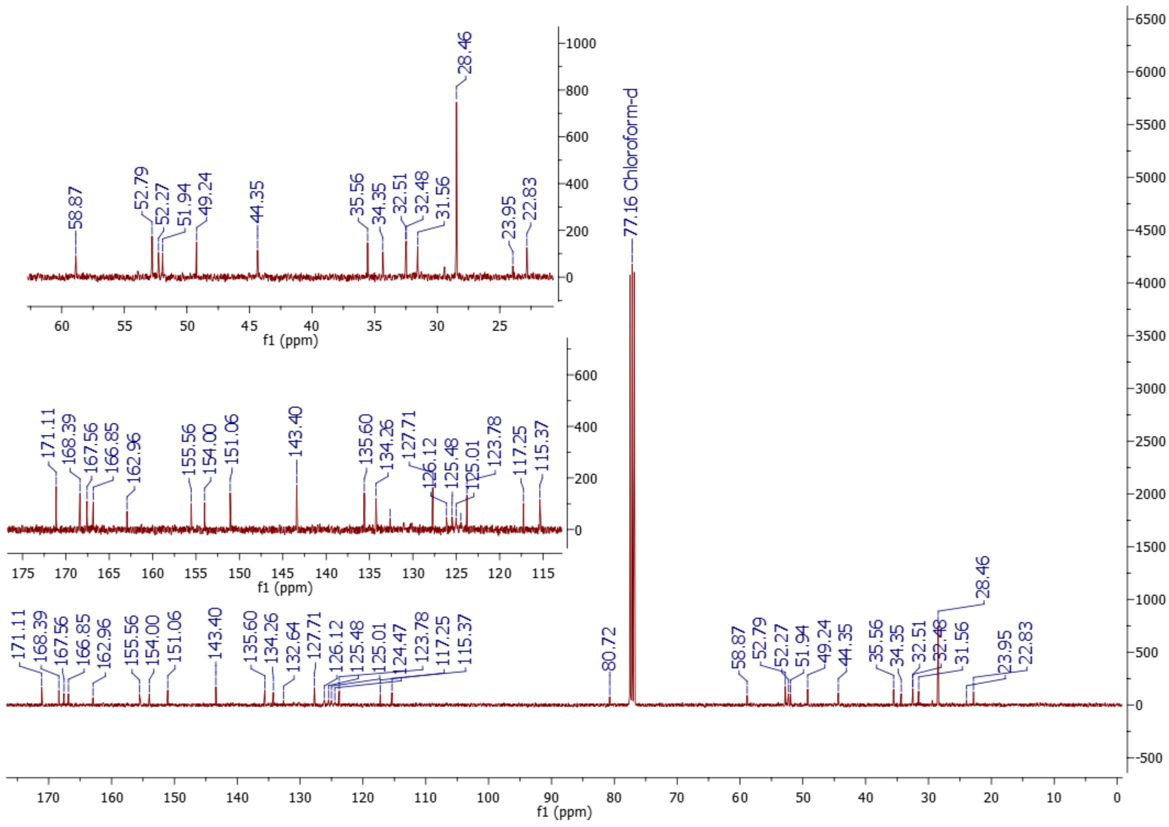
77.16 Chloroform-d
171.11
168.39
167.56
166.85
162.96
155.56
154.00
151.06
143.40
135.60
134.26
132.64
127.71
126.12
125.48
125.01
124.47
123.78
117.25
115.37
80.72
58.87
52.79
52.27
51.94
49.24
44.35
35.56
34.35
32.51
32.48
31.56
28.46
23.95
22.83
f1 (ppm)

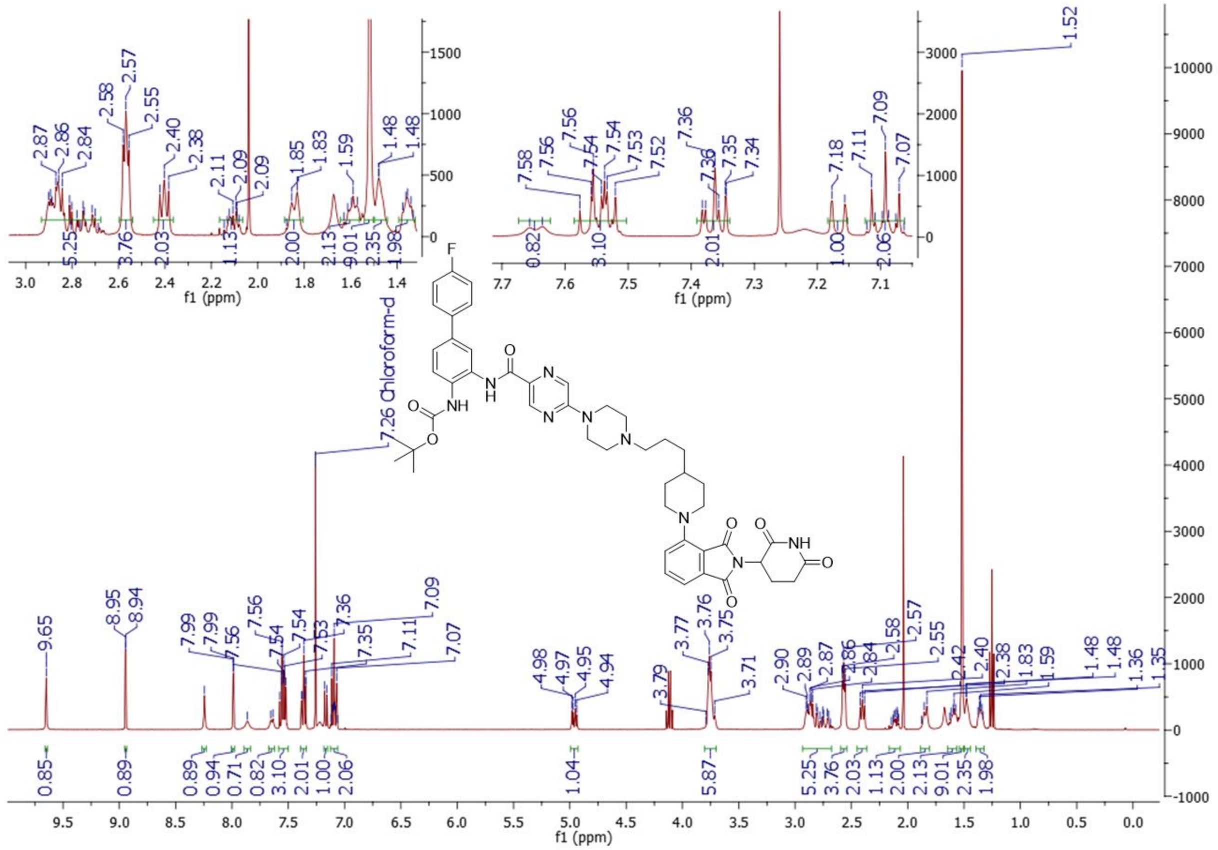

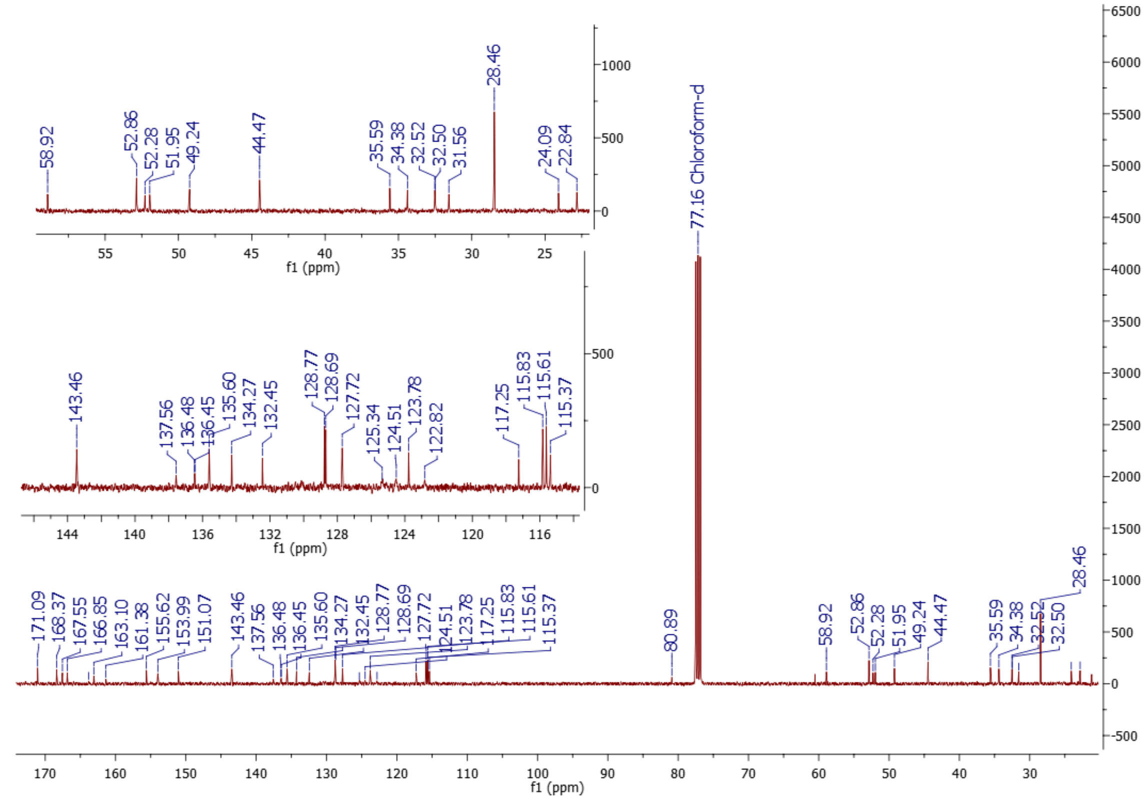

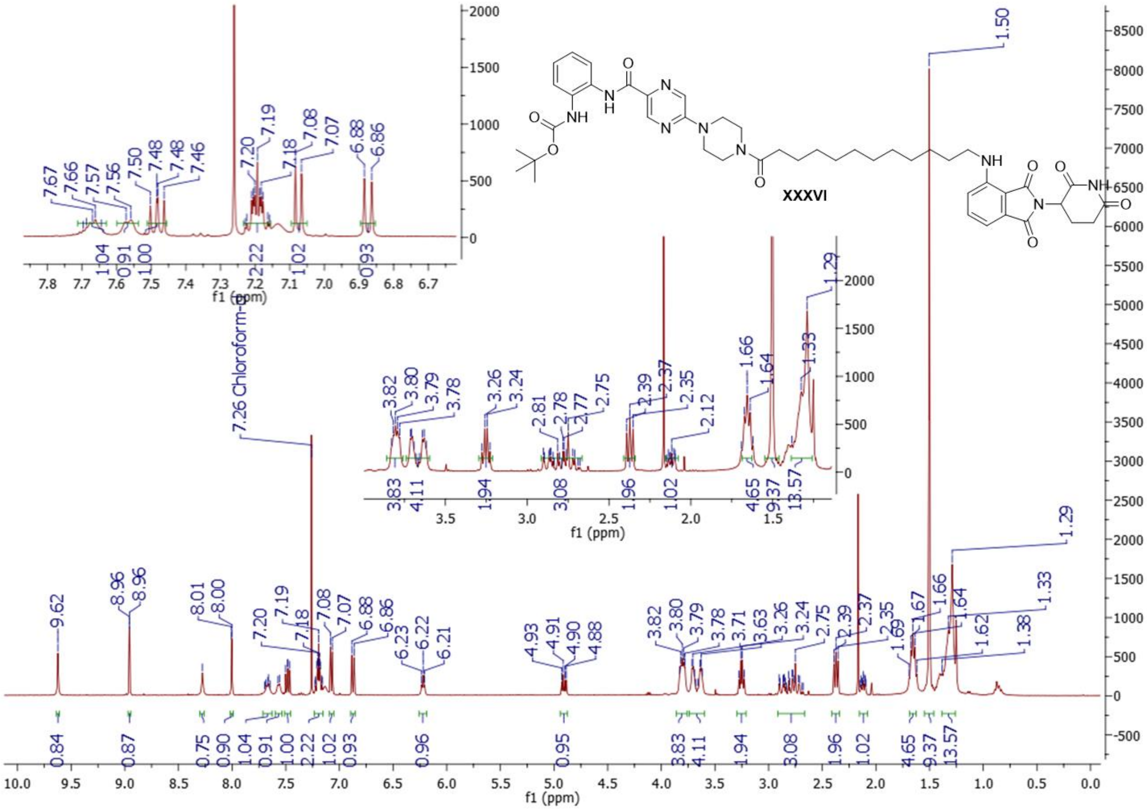
XXXVI
7.26 Chloroform-d
9.62
8.96
8.96
8.01
8.00
7.20
7.19
7.18
7.08
7.07
6.88
6.86
6.23
6.22
6.21
4.93
4.91
4.90
4.88
3.82
3.80
3.79
3.78
3.71
3.63
3.26
3.24
2.75
2.39
2.37
2.35
1.67
1.66
1.64
1.62
1.50
1.38
1.33
1.29
f1 (ppm)

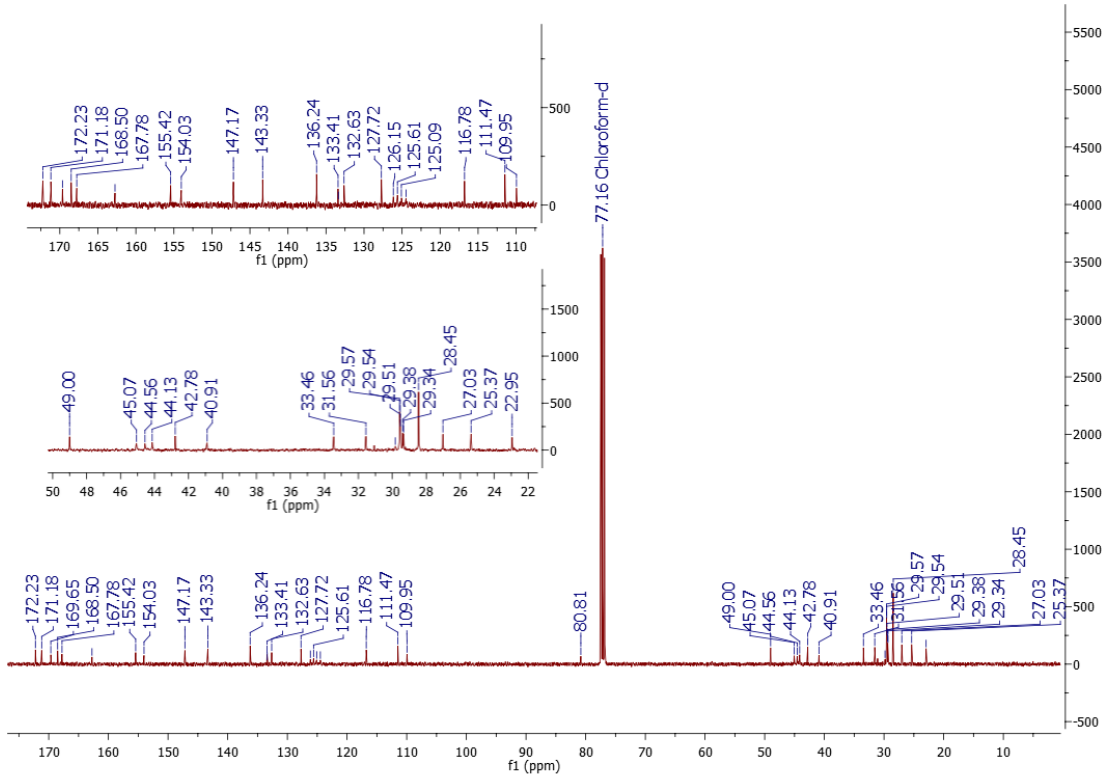
77.16 Chloroform-d
172.23
171.18
169.65
168.50
167.78
155.42
154.03
147.17
143.33
136.24
133.41
132.63
127.72
125.61
116.78
111.47
109.95
80.81
49.00
45.07
44.56
44.13
42.78
40.91
33.46
31.56
29.57
29.54
29.51
29.38
29.34
28.45
27.03
25.37
22.95
f1 (ppm)

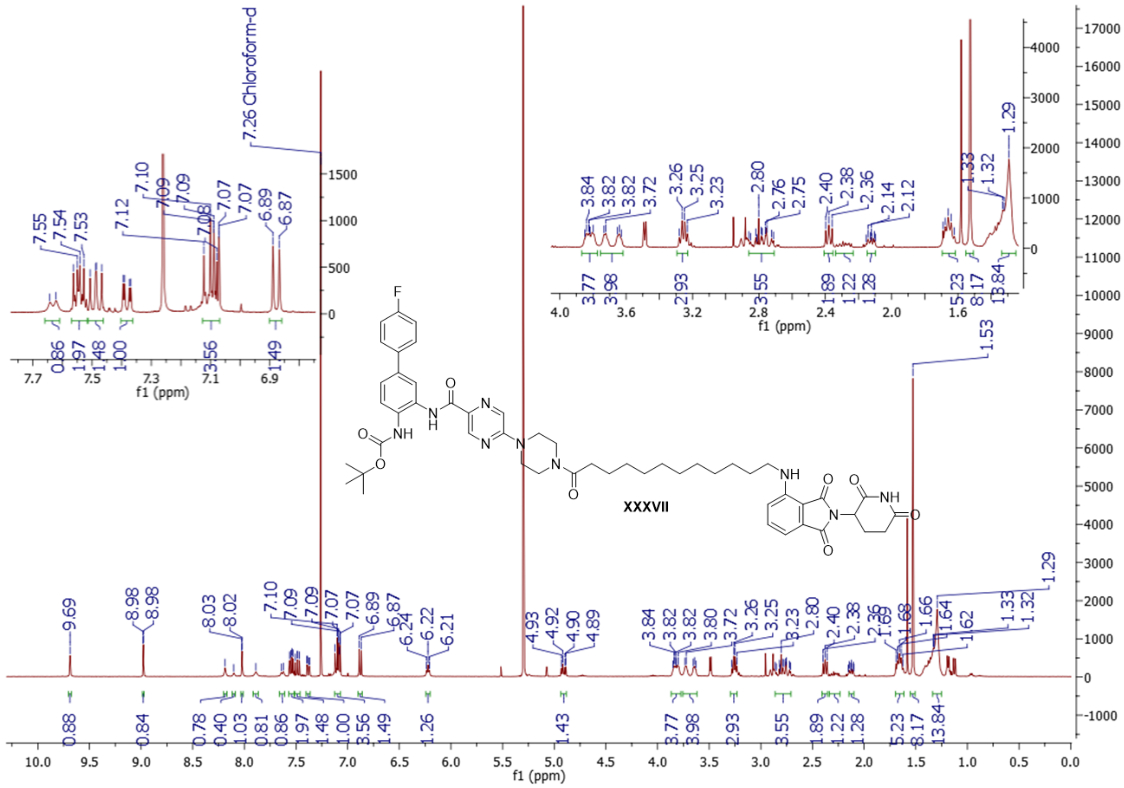

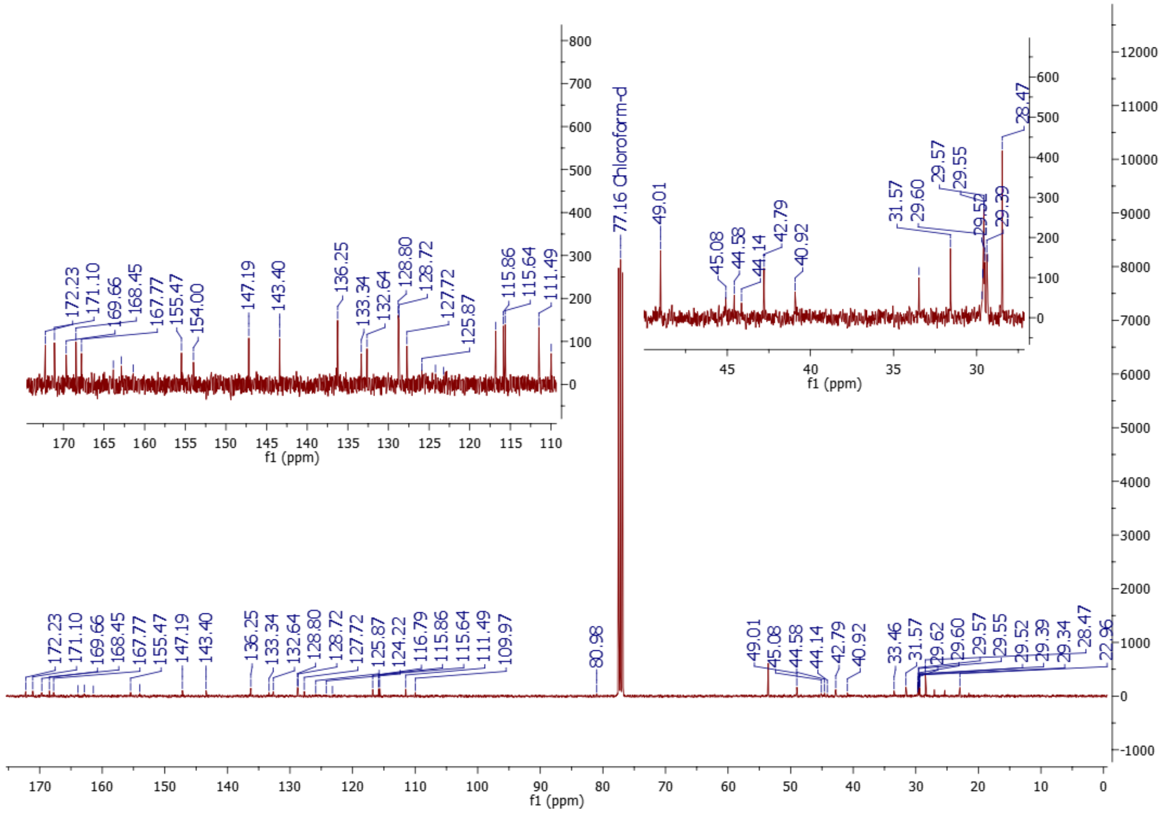

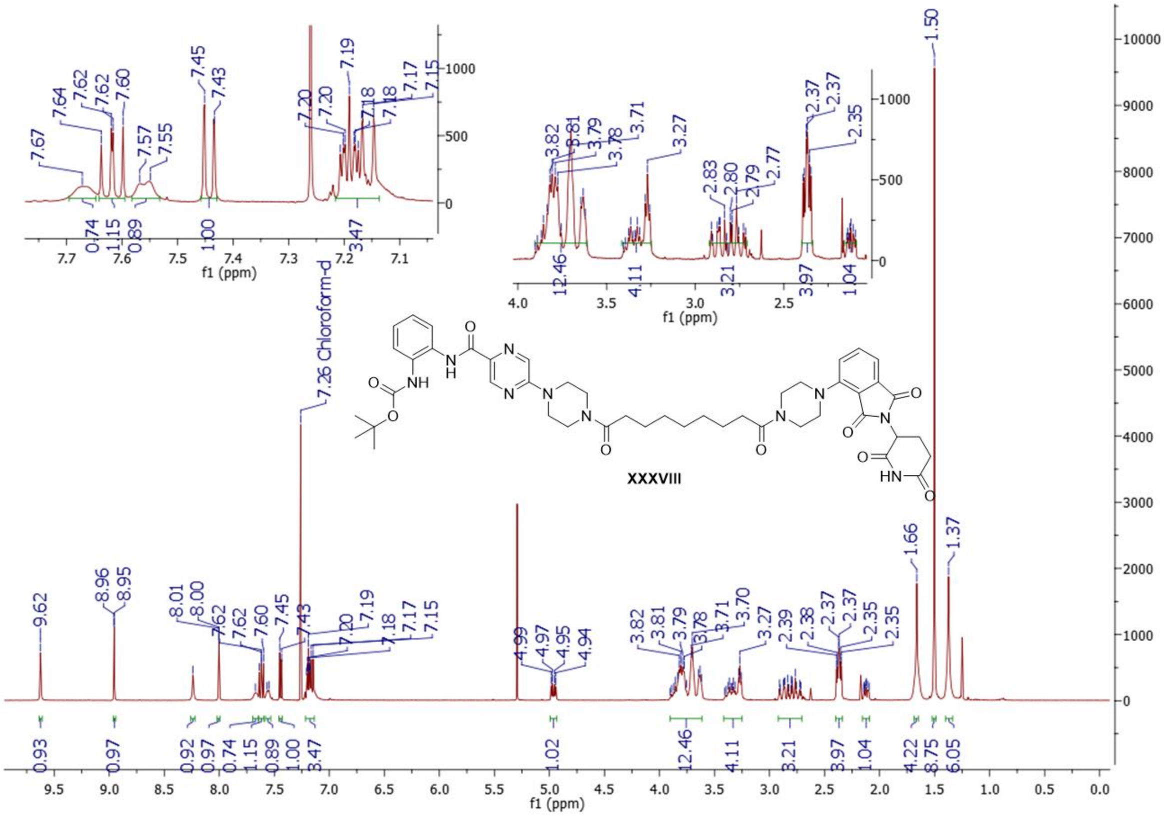
7.26 Chloroform-d
XXXVIII
f1 (ppm)

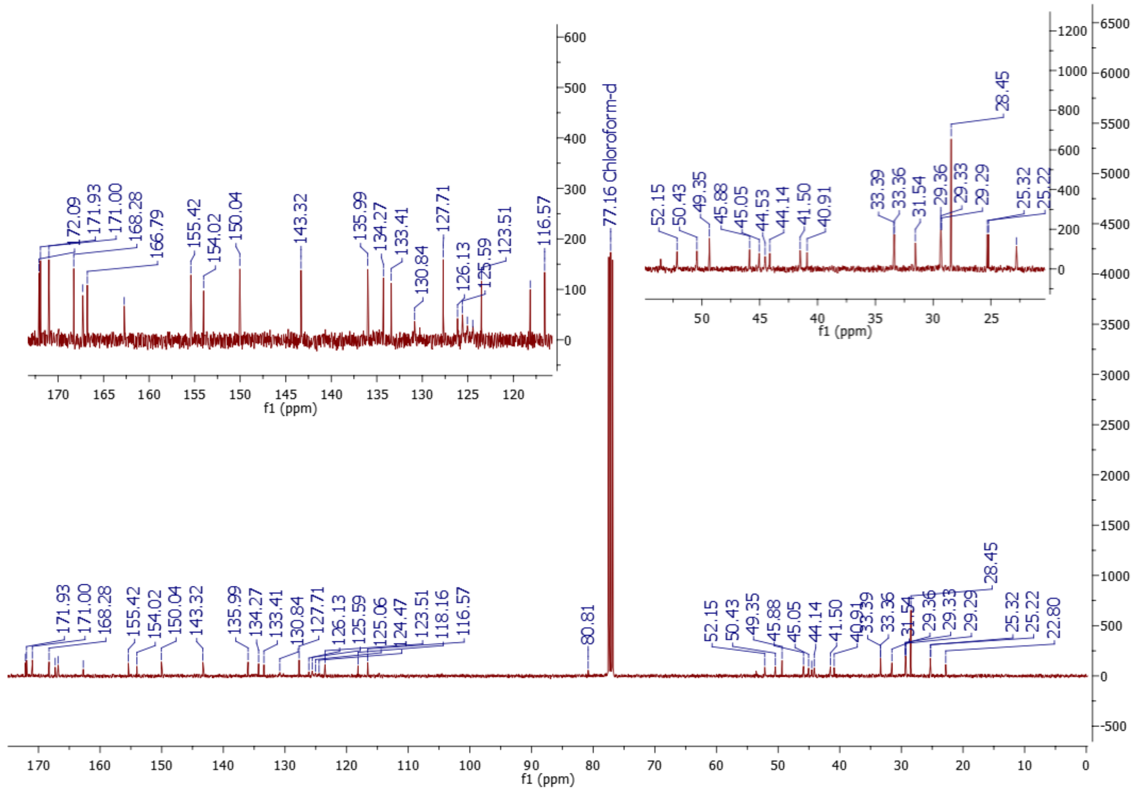
77.16 Chloroform-d
f1 (ppm)

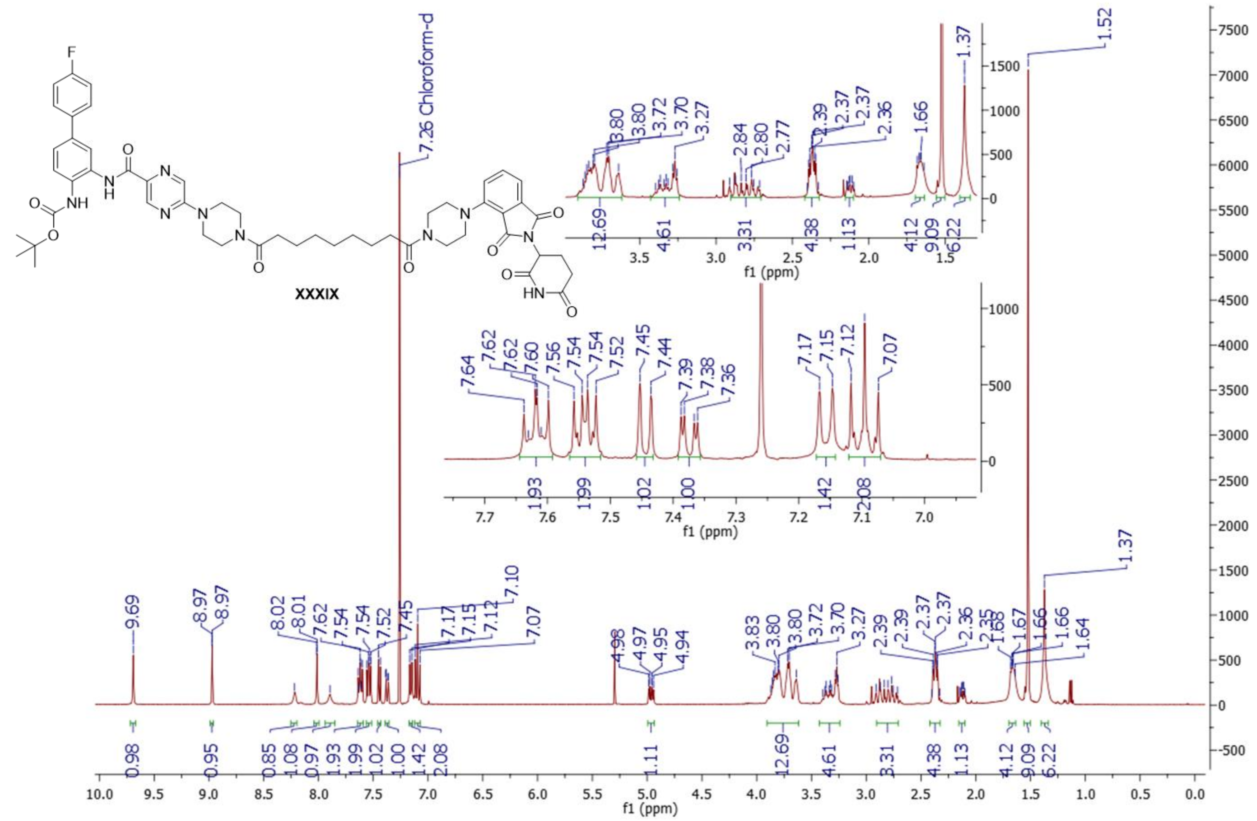
XXXIX
7.26 Chloroform-d
9.69
8.97
8.97
8.02
8.01
7.62
7.54
7.54
7.52
7.45
7.17
7.15
7.12
7.10
7.07
4.98
4.97
4.95
4.94
3.83
3.80
3.80
3.72
3.70
3.27
2.39
2.39
2.37
2.37
2.36
2.35
1.68
1.67
1.66
1.66
1.64
1.52
1.37
0.98
0.95
0.85
1.08
0.97
1.93
1.99
1.02
1.00
1.42
2.08
1.11
12.69
4.61
3.31
4.38
1.13
4.12
9.09
6.22
f1 (ppm)

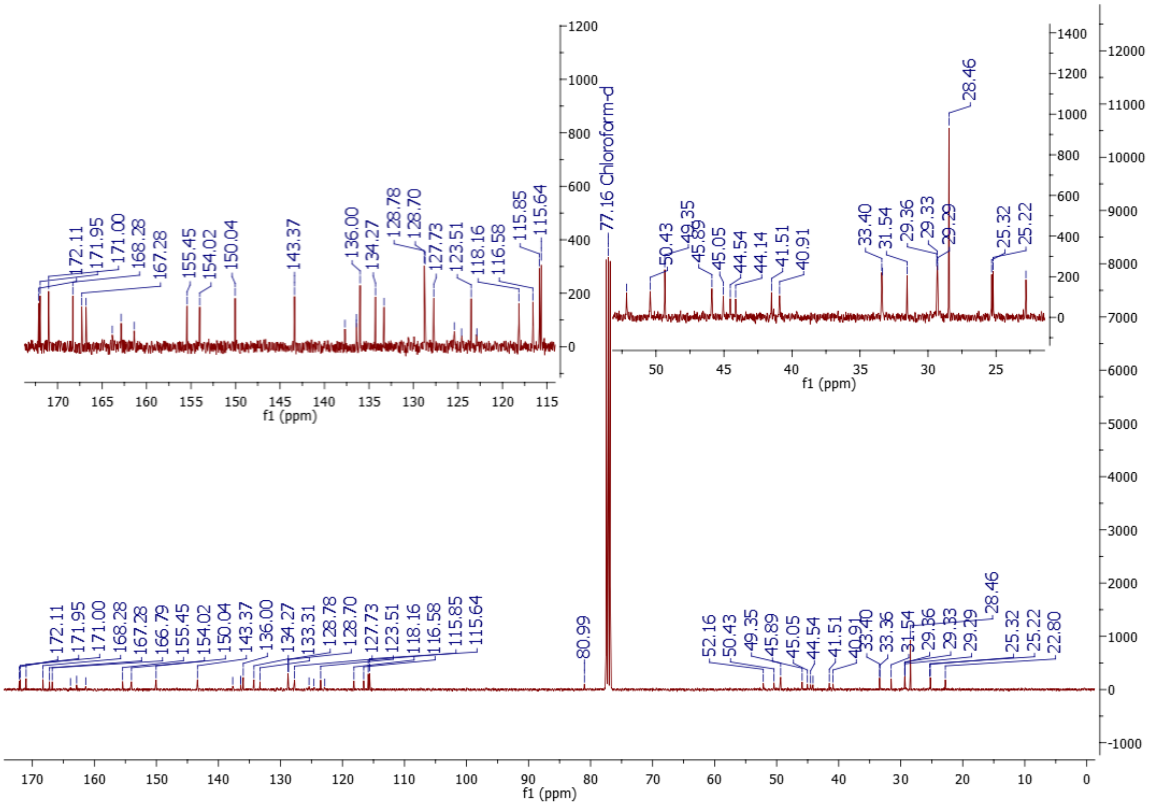
77.16 Chloroform-d
172.11
171.95
171.00
168.28
167.28
166.79
155.45
154.02
150.04
143.37
136.00
134.27
133.31
128.78
128.70
127.73
123.51
118.16
116.58
115.85
115.64
80.99
52.16
50.43
49.35
45.89
45.05
44.54
44.14
41.51
40.91
33.40
33.36
31.54
29.36
29.33
29.29
28.46
25.32
25.22
22.80
f1 (ppm)

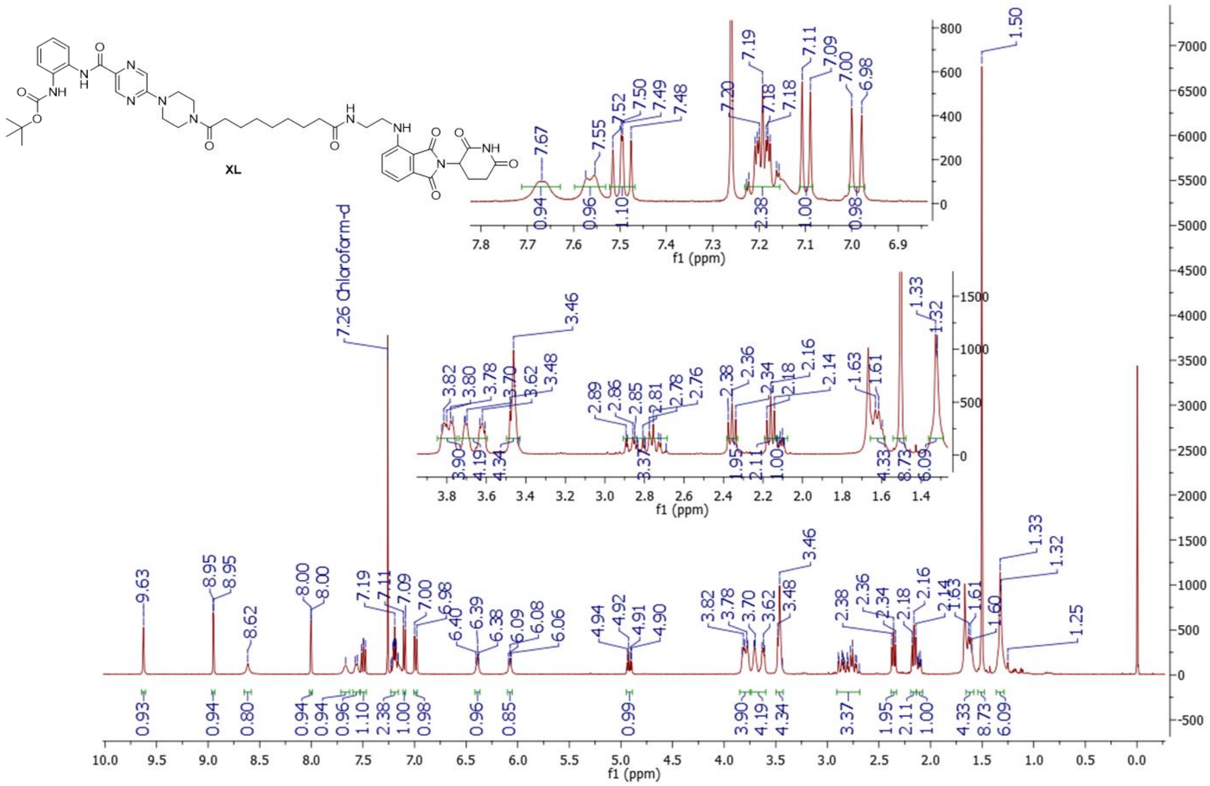

XL
7.26 Chloroform-d
f1 (ppm)
f1 (ppm)
f1 (ppm)

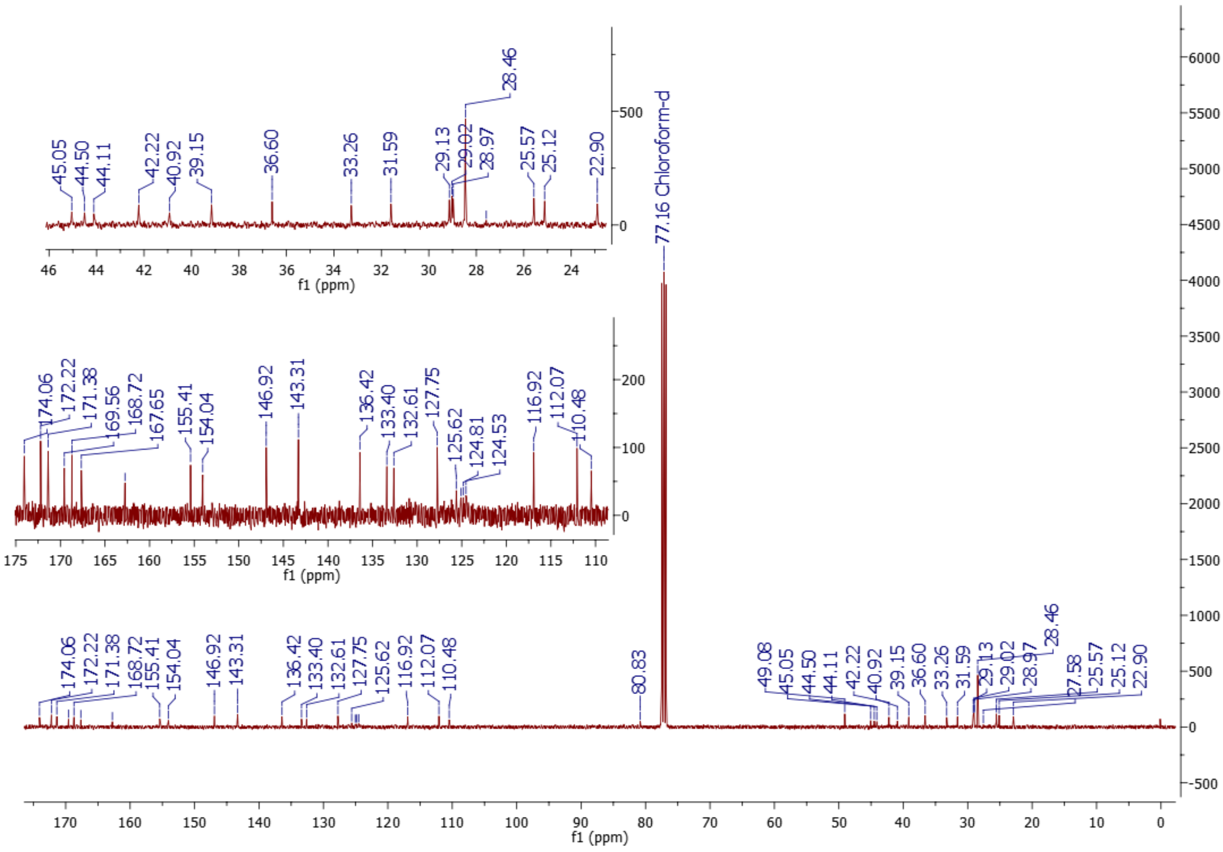

77.16 Chloroform-d
f1 (ppm)
f1 (ppm)
f1 (ppm)

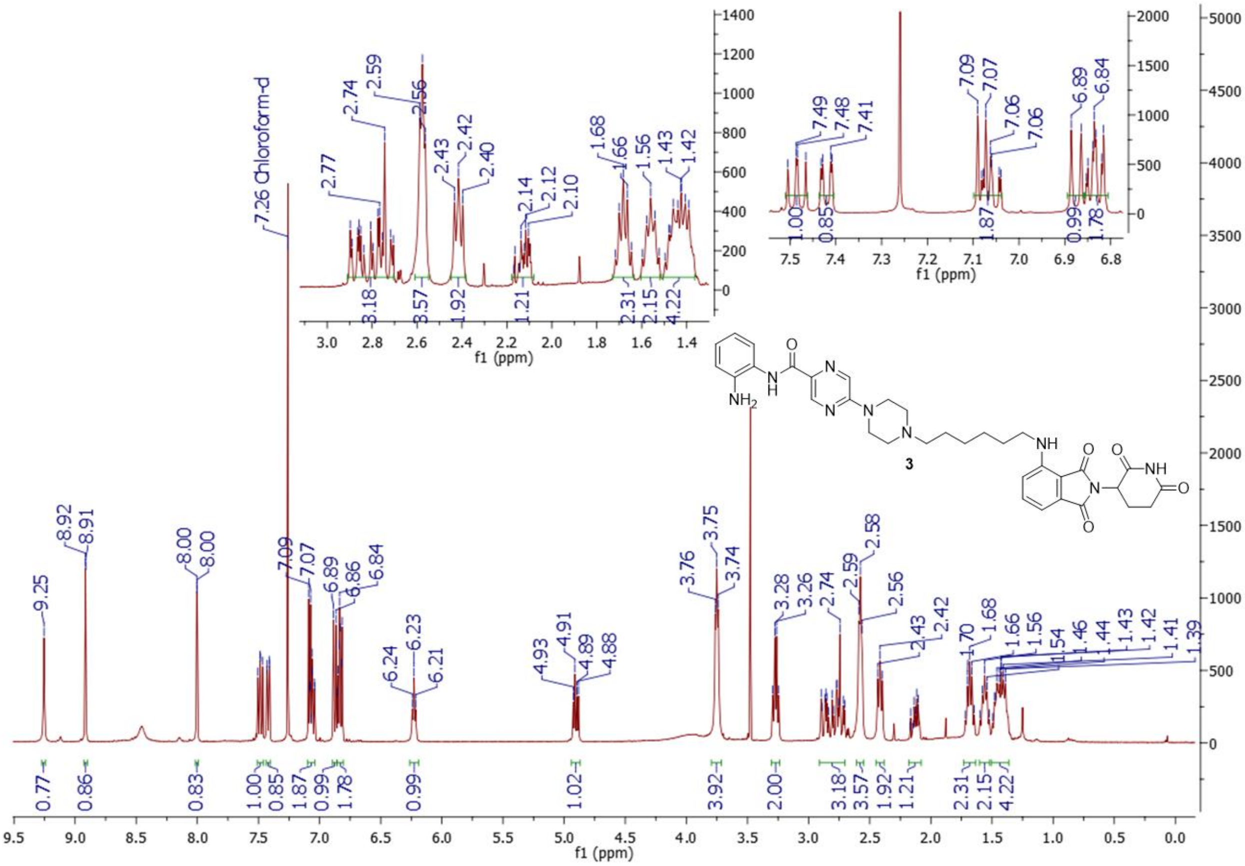

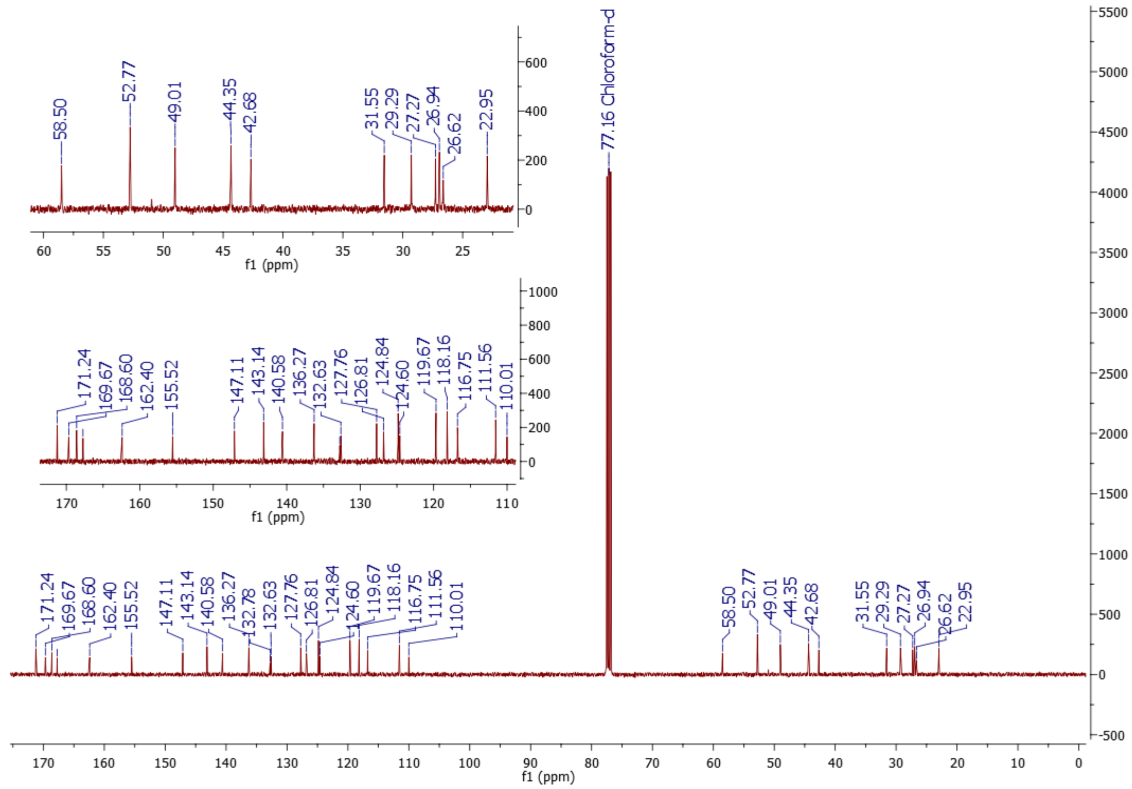

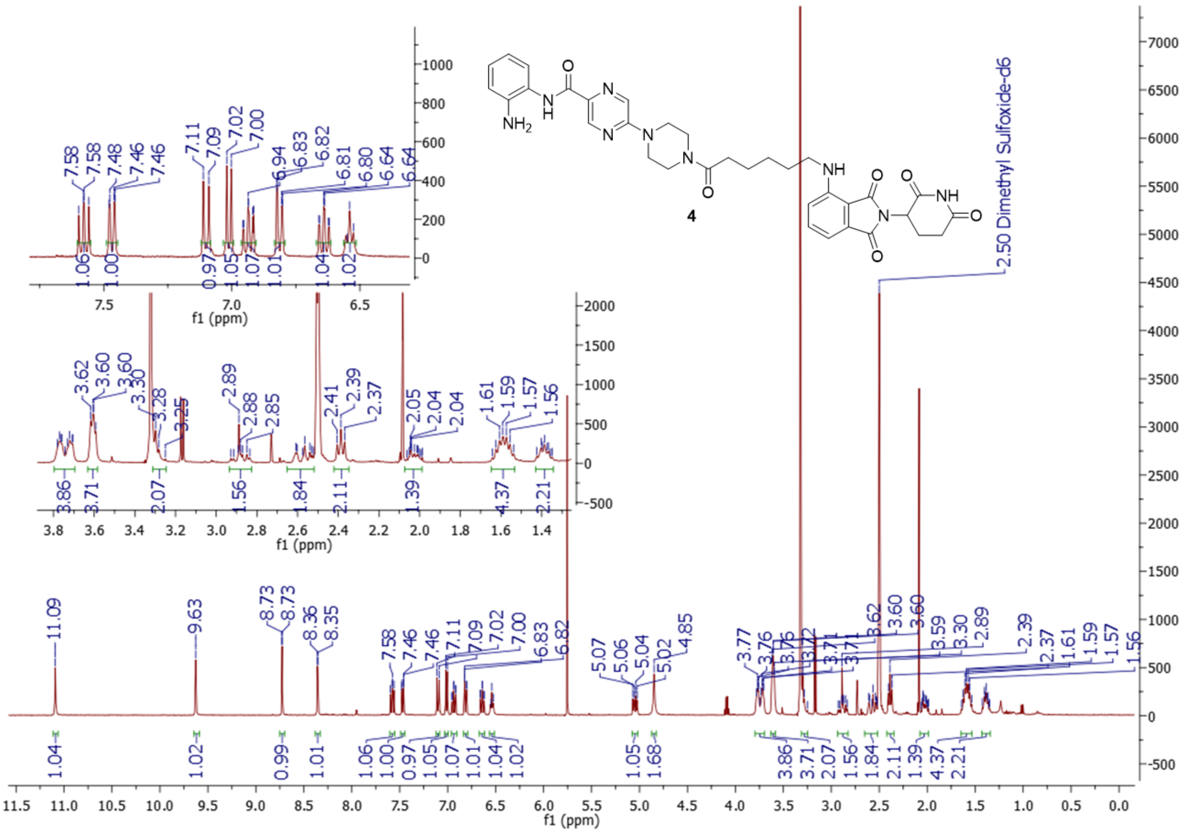

4
2.50 Dimethyl Sulfoxide-d6
f1 (ppm)

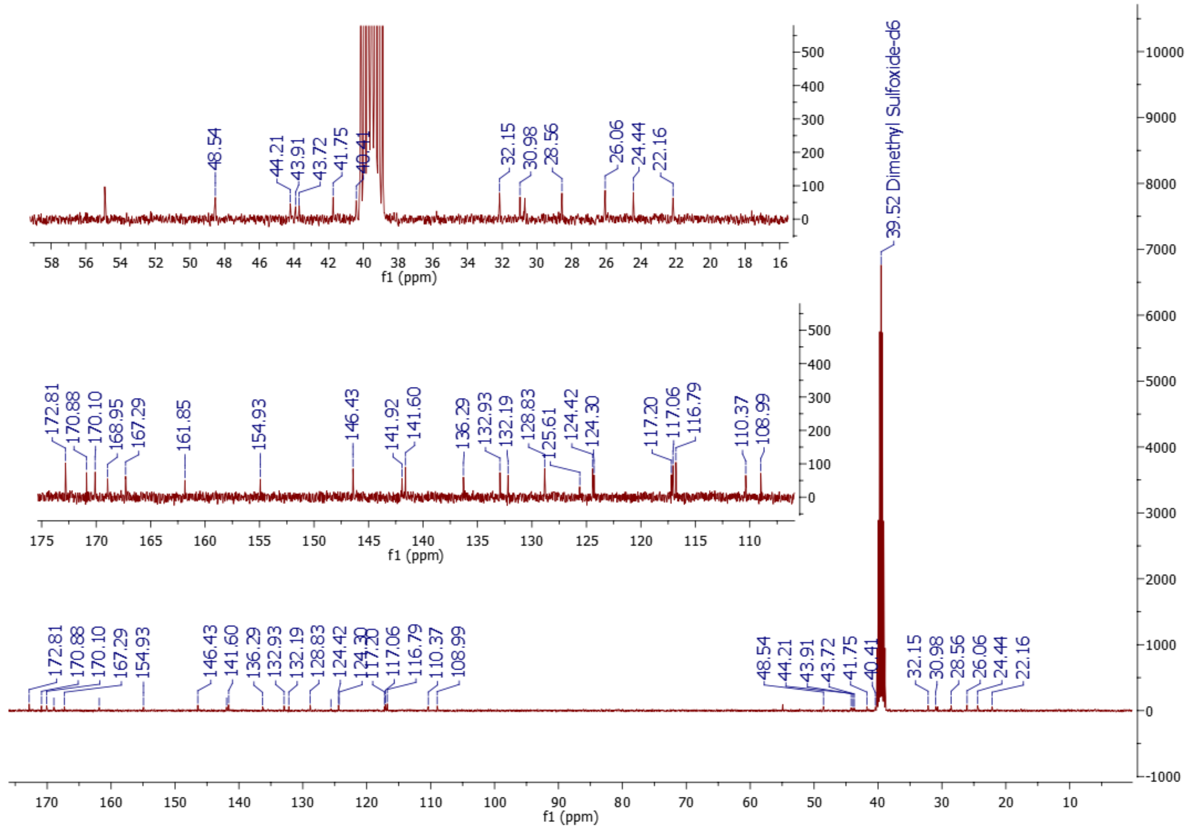

39.52 Dimethyl Sulfoxide-d6
f1 (ppm)

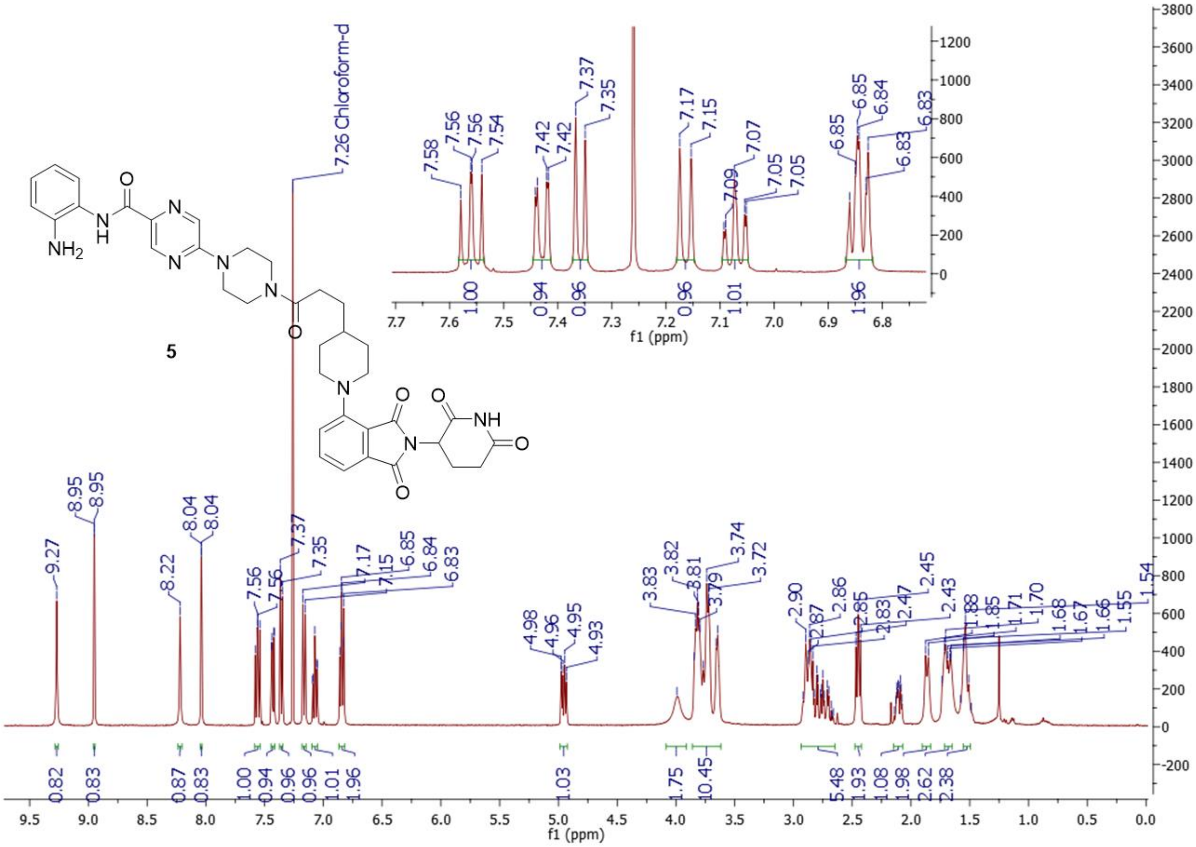

5
7.26 Chloroform-d
9.27
8.95
8.95
8.22
8.04
8.04
7.56
7.56
7.37
7.35
7.17
7.15
6.85
6.84
6.83
4.98
4.96
4.95
4.93
3.83
3.82
3.81
3.79
3.74
3.72
2.90
2.87
2.86
2.85
2.83
2.47
2.45
2.43
1.88
1.85
1.71
1.70
1.68
1.67
1.66
1.55
1.54
0.82
0.83
0.87
0.83
1.00
0.94
0.96
0.96
1.01
1.96
1.03
1.75
10.45
5.48
1.93
1.08
1.98
2.62
2.38
7.58
7.56
7.56
7.54
7.42
7.42
7.37
7.35
7.17
7.15
7.09
7.07
7.05
7.05
6.85
6.85
6.84
6.83
6.83
f1 (ppm)

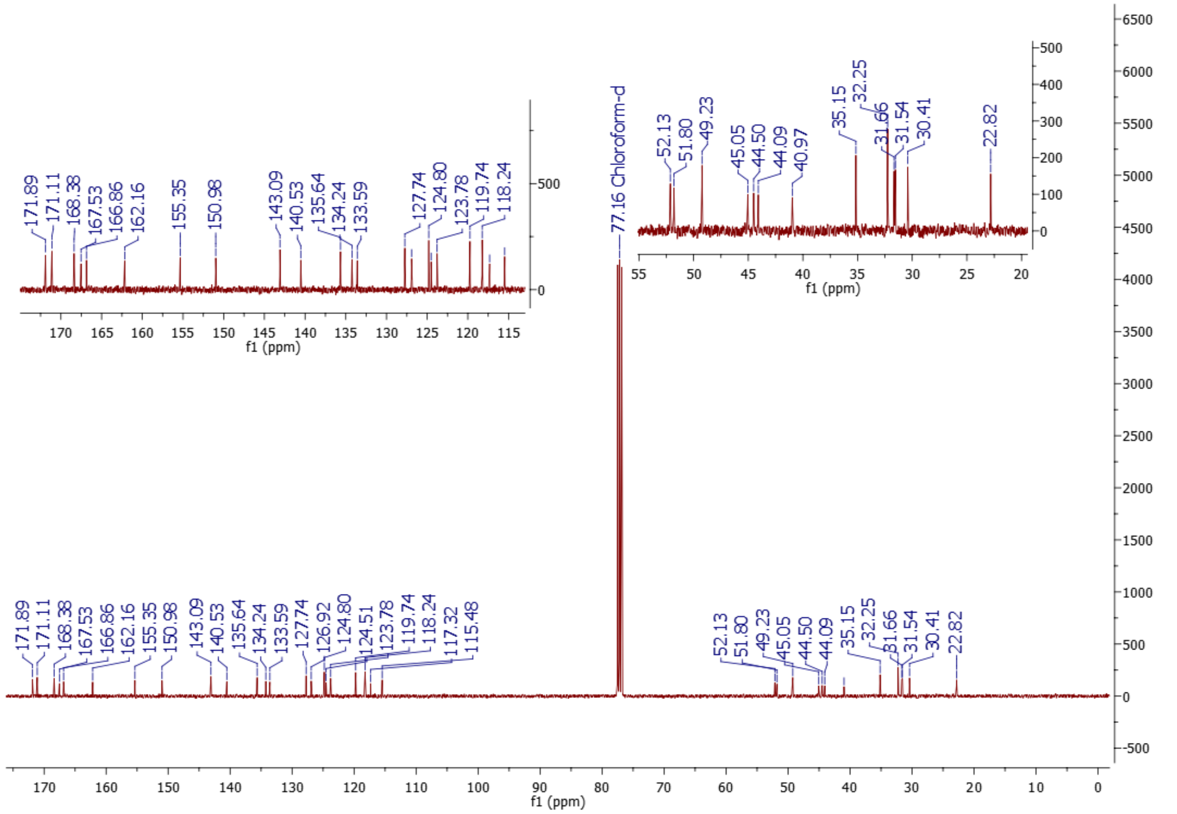

77.16 Chloroform-d
171.89
171.11
168.38
167.53
166.86
162.16
155.35
150.98
143.09
140.53
135.64
134.24
133.59
127.74
126.92
124.80
124.51
123.78
119.74
118.24
117.32
115.48
52.13
51.80
49.23
45.05
44.50
44.09
40.97
35.15
32.25
31.66
31.54
30.41
22.82
f1 (ppm)

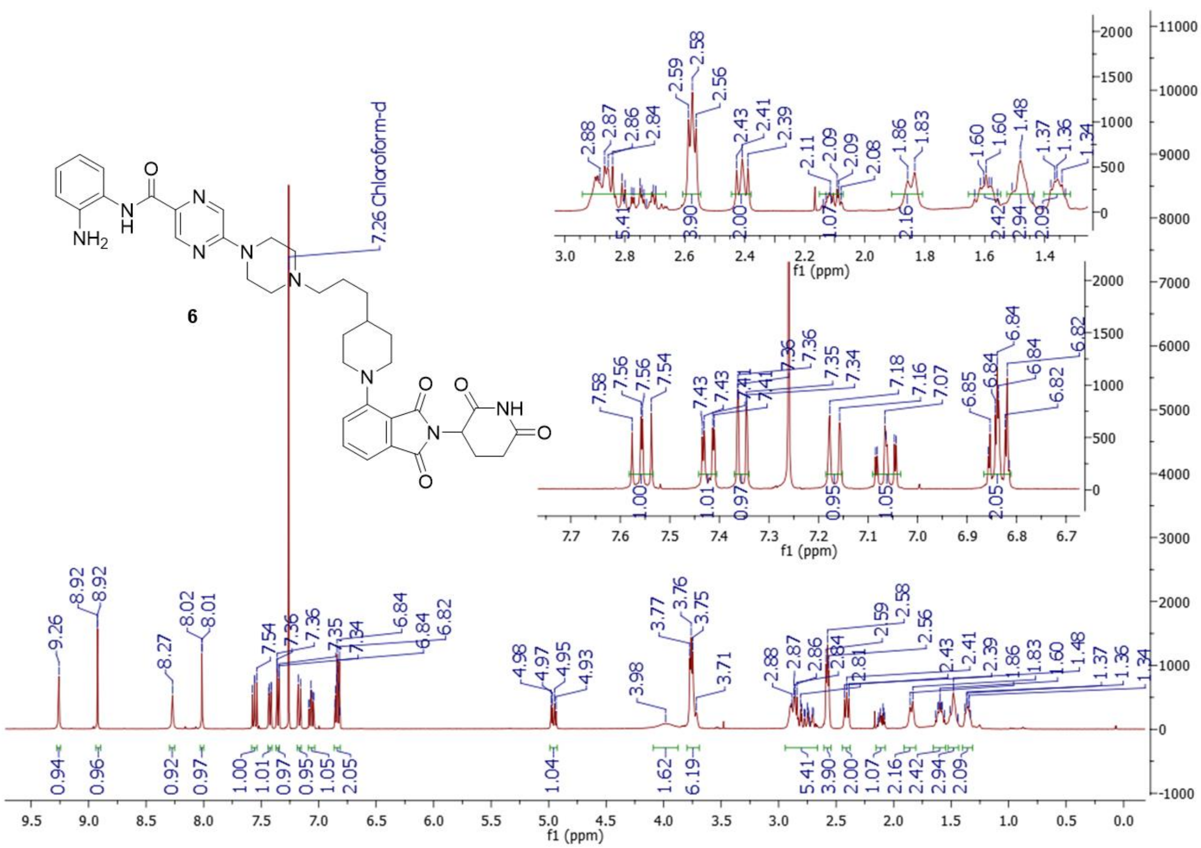
6
7.26 Chloroform-d
9.26 8.92 8.92 8.27 8.02 8.01 7.54 7.36 7.36 7.35 7.34 6.84 6.84 6.82 4.98 4.97 4.95 4.93 3.98 3.77 3.76 3.75 3.71 2.88 2.87 2.86 2.84 2.81 2.59 2.58 2.56 2.43 2.41 2.39 1.86 1.83 1.60 1.48 1.37 1.36 1.34
0.94 0.96 0.92 0.97 1.00 1.01 0.97 0.95 1.05 2.05 1.04 1.62 6.19 5.41 3.90 2.00 1.07 2.16 2.42 2.94 2.09
f1 (ppm)

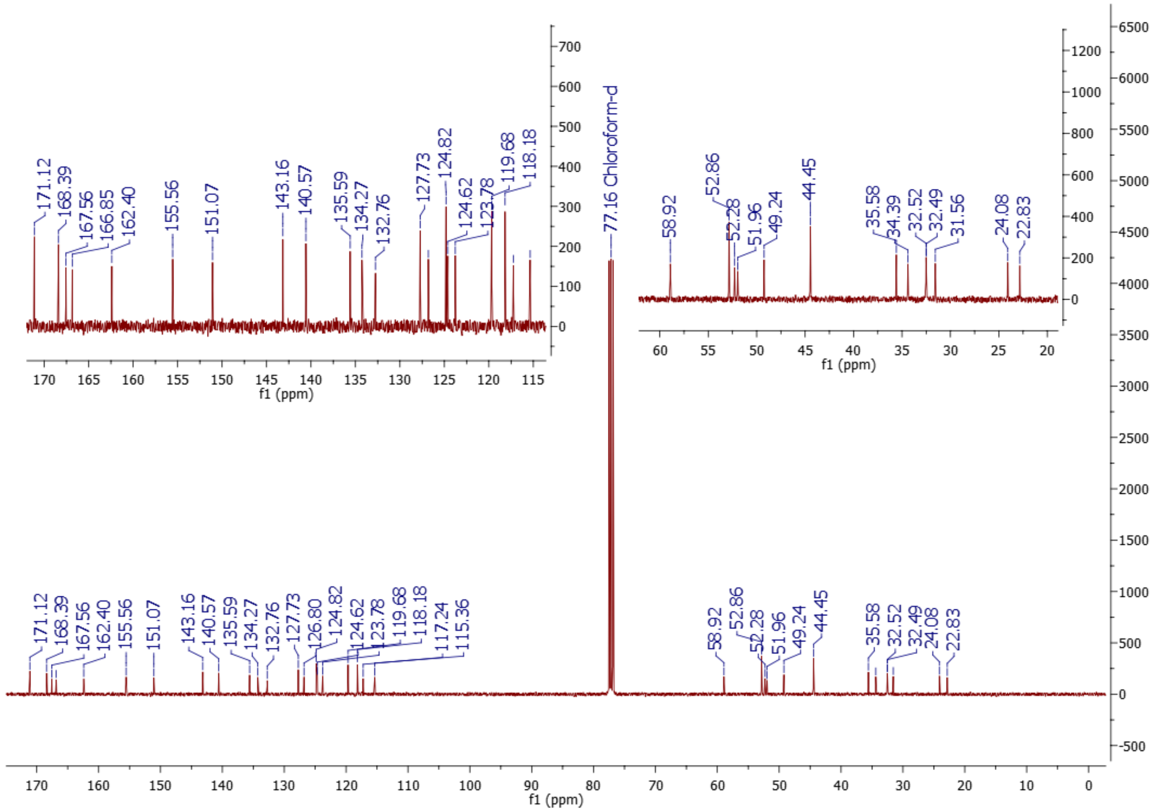
77.16 Chloroform-d
171.12 168.39 167.56 166.85 162.40 155.56 151.07 143.16 140.57 135.59 134.27 132.76 127.73 126.80 124.82 124.62 123.78 119.68 118.18 117.24 115.36
58.92 52.86 52.28 51.96 49.24 44.45 35.58 34.39 32.52 32.49 31.56 24.08 22.83
f1 (ppm)

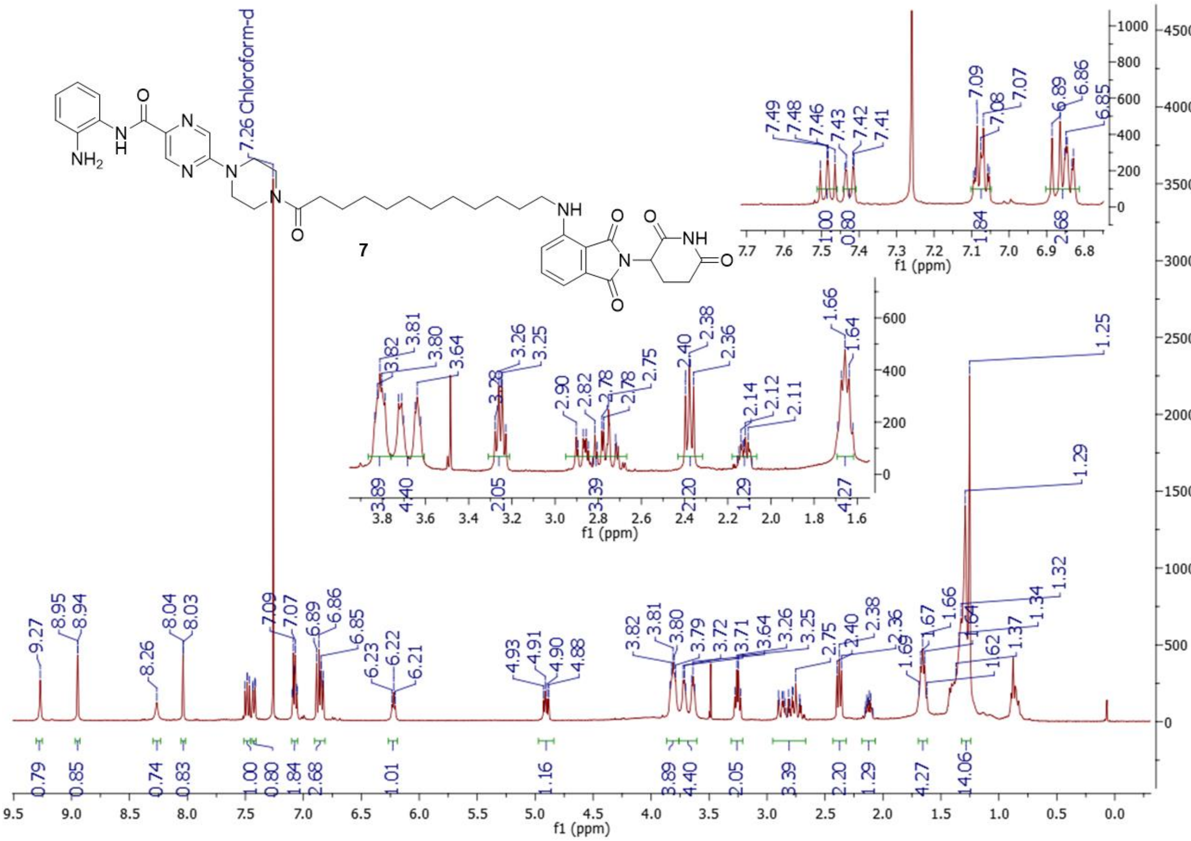

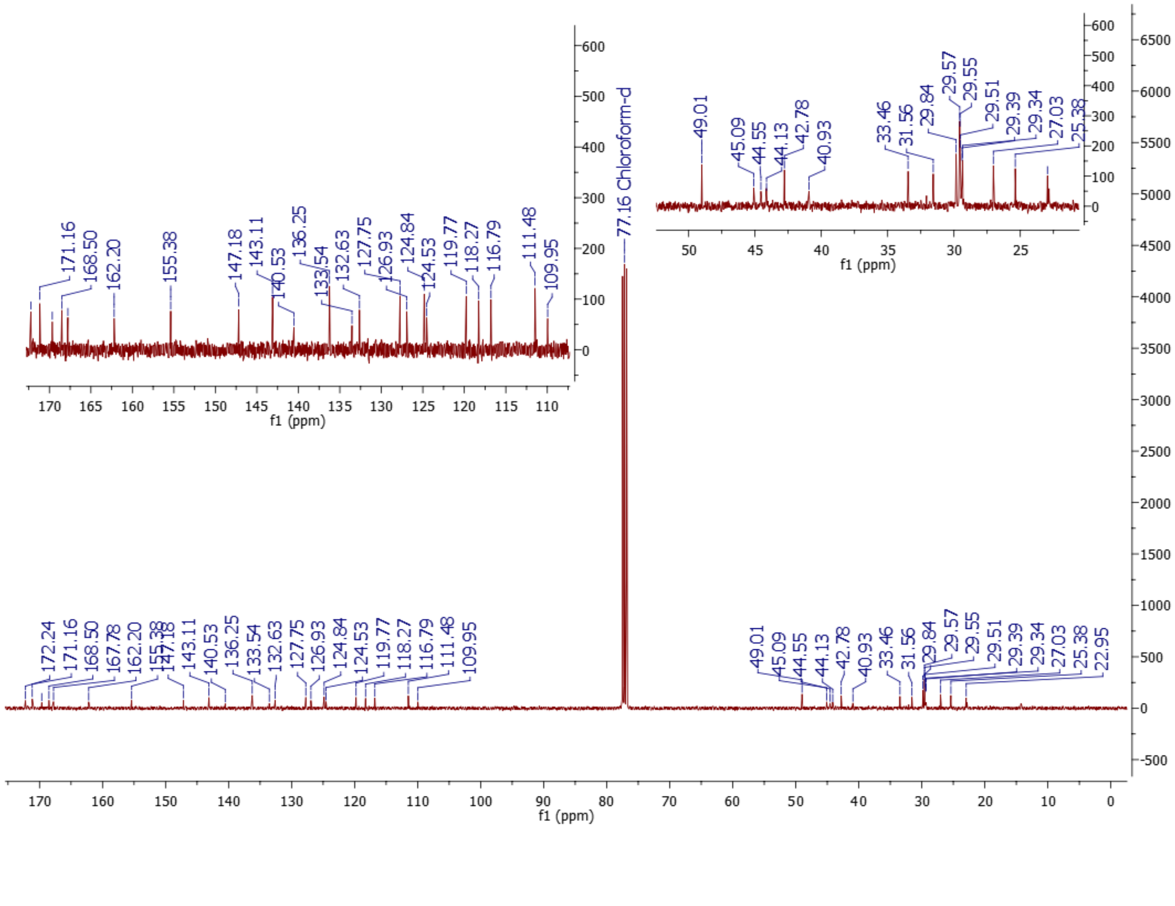

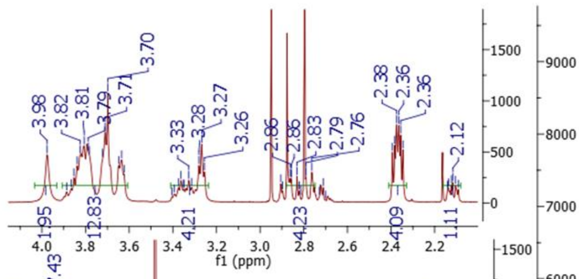

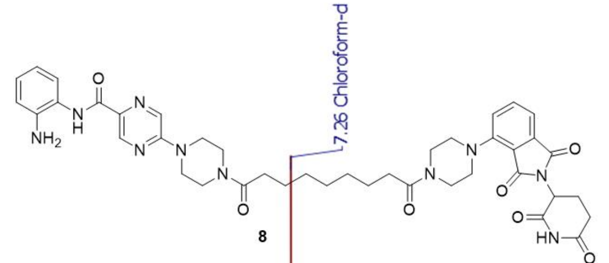

8

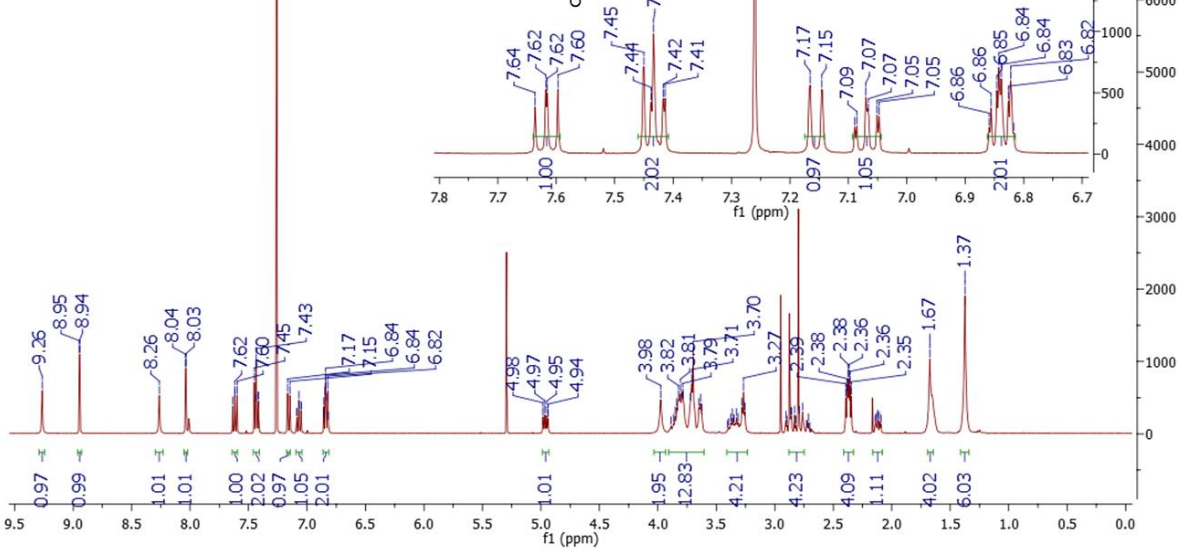
7.26 Chloroform-d
9.26 8.95 8.94 8.26 8.04 8.03 7.62 7.60 7.45 7.43 7.17 7.15 6.84 6.84 6.82
4.98 4.97 4.95 4.94
3.98 3.82 3.81 3.79 3.71 3.70 3.27 2.89 2.38 2.38 2.36 2.36 2.35 1.67 1.37
0.97 0.99 1.01 1.01 1.00 2.02 0.97 1.05 2.01 1.01 1.95 12.83 4.21 4.23 4.09 1.11 4.02 6.03
7.64 7.62 7.62 7.60 7.45 7.44 7.43 7.42 7.41 7.17 7.15 7.09 7.07 7.07 7.05 7.05 6.86 6.86 6.85 6.84 6.84 6.83 6.82
1.00 2.02 0.97 1.05 2.01
3.98 3.82 3.81 3.79 3.71 3.70 3.33 3.28 3.27 3.26 2.86 2.86 2.83 2.79 2.76 2.38 2.36 2.36 2.12
1.95 12.83 4.21 4.23 4.09 1.11
f1 (ppm)

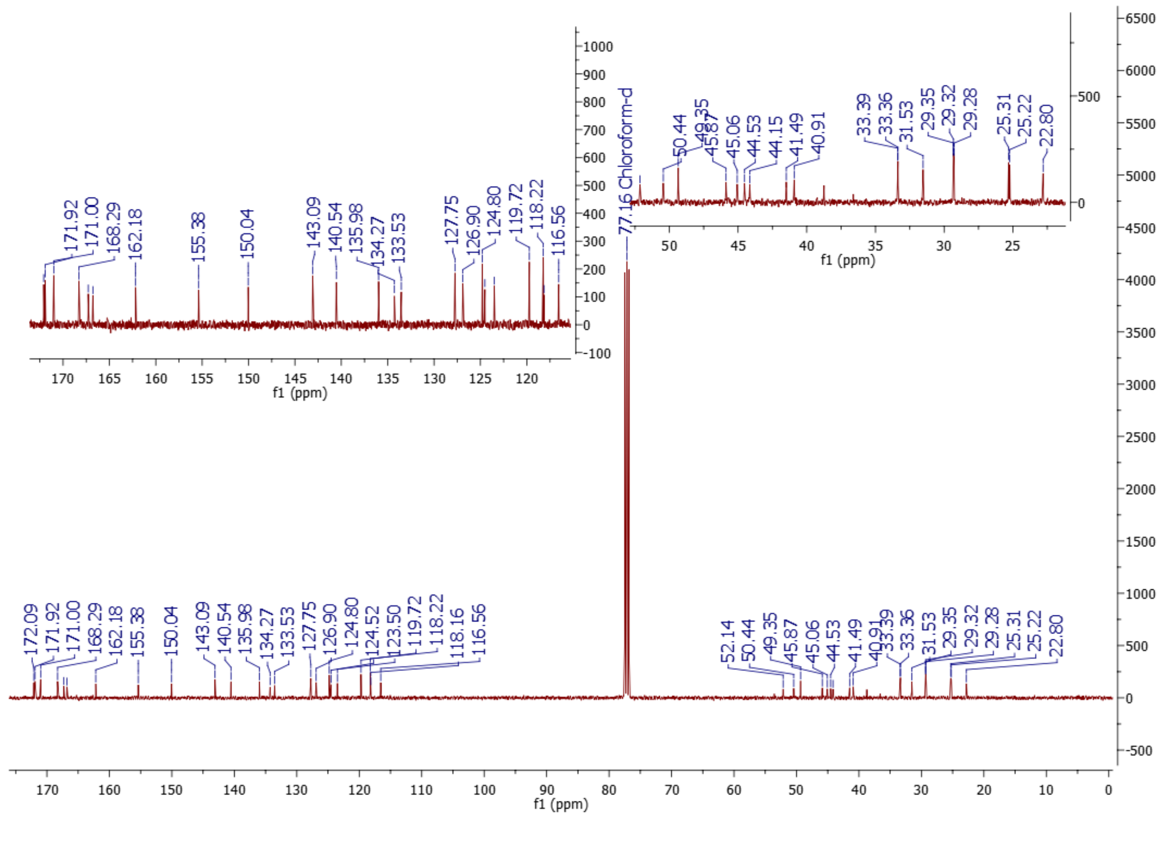
77.16 Chloroform-d
172.09 171.92 171.00 168.29 162.18 155.38 150.04 143.09 140.54 135.98 134.27 133.53 127.75 126.90 124.80 124.52 123.50 119.72 118.22 118.16 116.56
52.14 50.44 49.35 45.87 45.06 44.53 41.49 40.91 33.39 33.36 31.53 29.35 29.32 29.28 25.31 25.22 22.80
171.92 171.00 168.29 162.18 155.38 150.04 143.09 140.54 135.98 134.27 133.53 127.75 126.90 124.80 119.72 118.22 116.56
50.44 49.35 45.87 45.06 44.53 44.15 41.49 40.91 33.39 33.36 31.53 29.35 29.32 29.28 25.31 25.22 22.80
f1 (ppm)

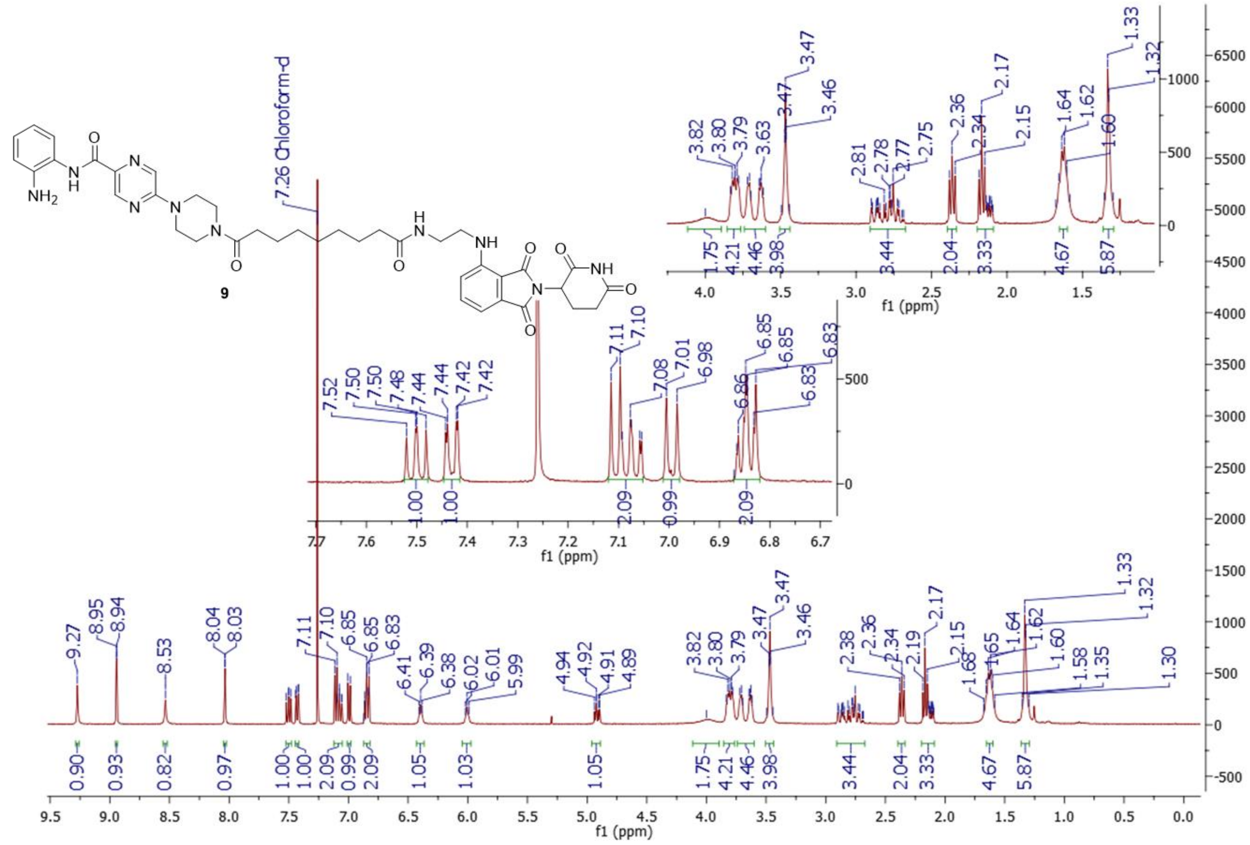

9
7.26 Chloroform-d
9.27
8.95
8.94
8.53
8.04
8.03
7.11
7.10
6.85
6.85
6.83
6.41
6.39
6.38
6.02
6.01
5.99
4.94
4.92
4.91
4.89
3.82
3.80
3.79
3.47
3.47
3.46
2.38
2.36
2.34
2.19
2.17
2.15
1.68
1.65
1.64
1.62
1.60
1.58
1.35
1.33
1.32
1.30
f1 (ppm)

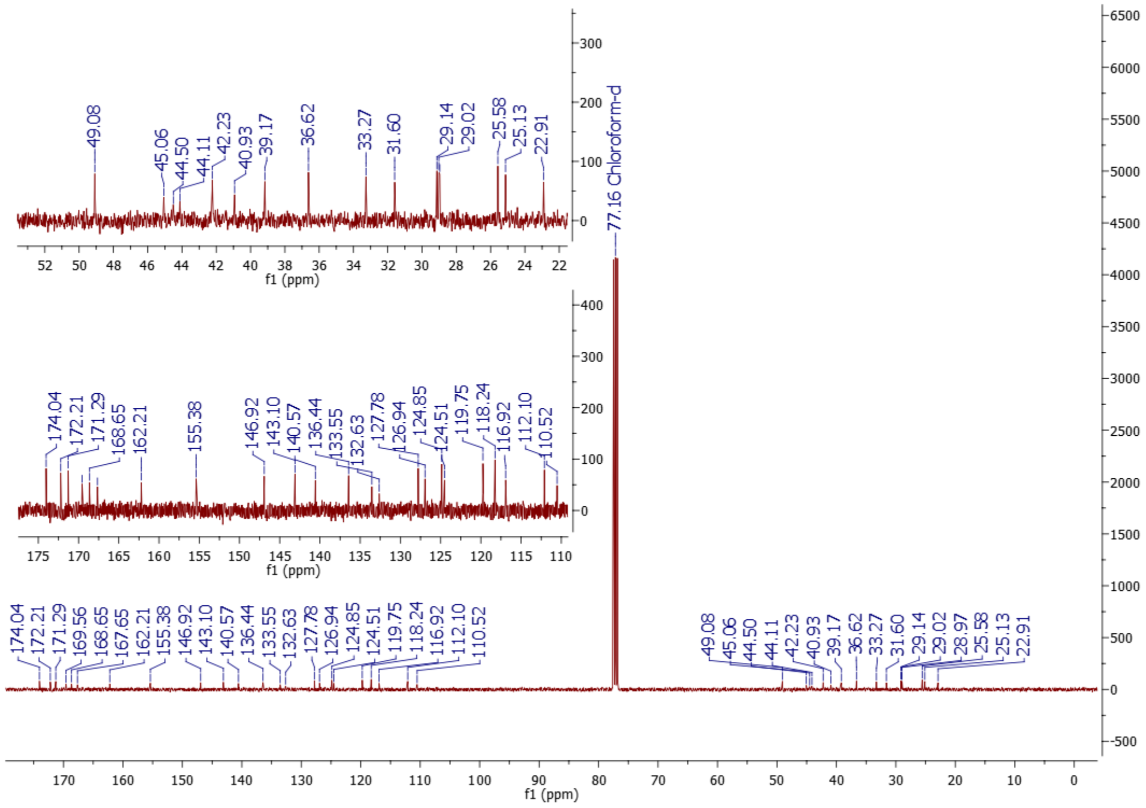
77.16 Chloroform-d
174.04
172.21
171.29
169.56
168.65
167.65
162.21
155.38
146.92
143.10
140.57
136.44
133.55
132.63
127.78
126.94
124.85
124.51
119.75
118.24
116.92
112.10
110.52
49.08
45.06
44.50
44.11
42.23
40.93
39.17
36.62
33.27
31.60
29.14
29.02
28.97
25.58
25.13
22.91
f1 (ppm)

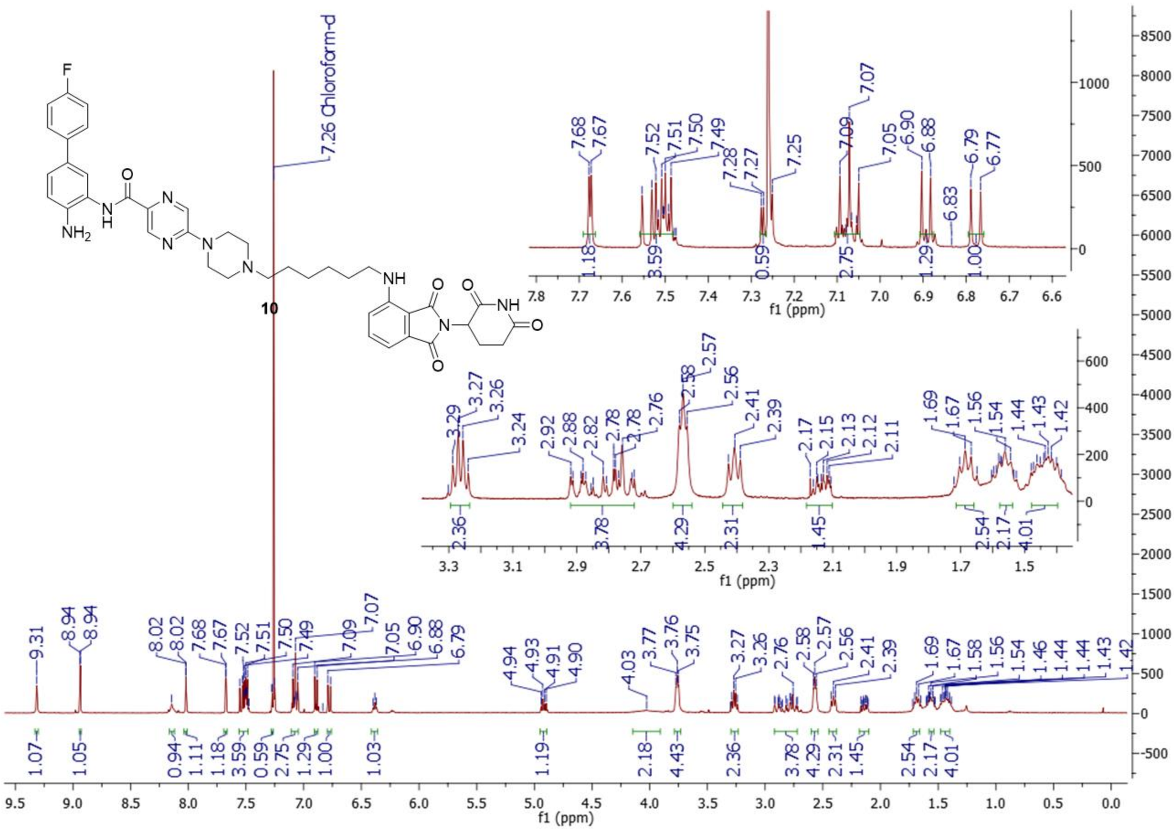
F
O
N
H
NH2
N
N
N
N
10
NH
O
O
O
NH
O
N
O
7.26 Chloroform-d
f1 (ppm)
f1 (ppm)
f1 (ppm)

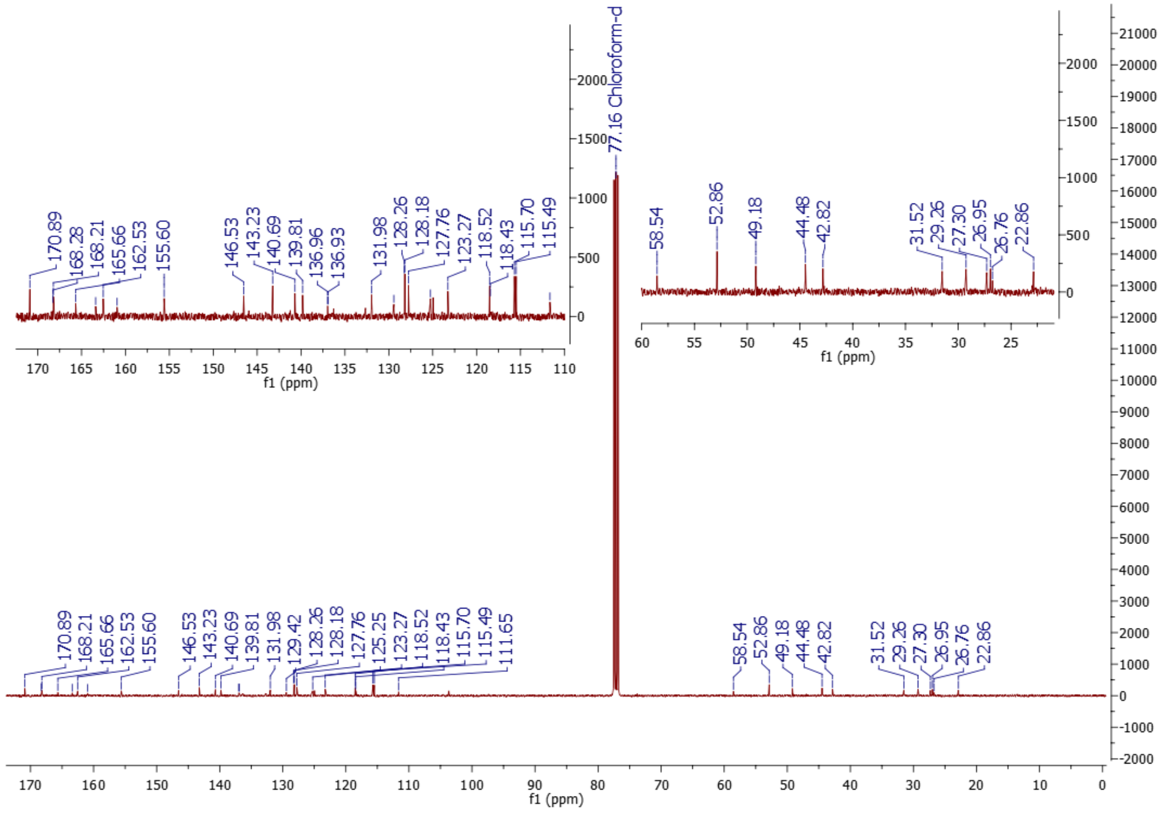
77.16 Chloroform-d
170.89
168.21
165.66
162.53
155.60
146.53
143.23
140.69
139.81
131.98
129.42
128.26
128.18
127.76
125.25
123.27
118.52
118.43
115.70
115.49
111.65
58.54
52.86
49.18
44.48
42.82
31.52
29.26
27.30
26.95
26.76
22.86
f1 (ppm)
f1 (ppm)
f1 (ppm)

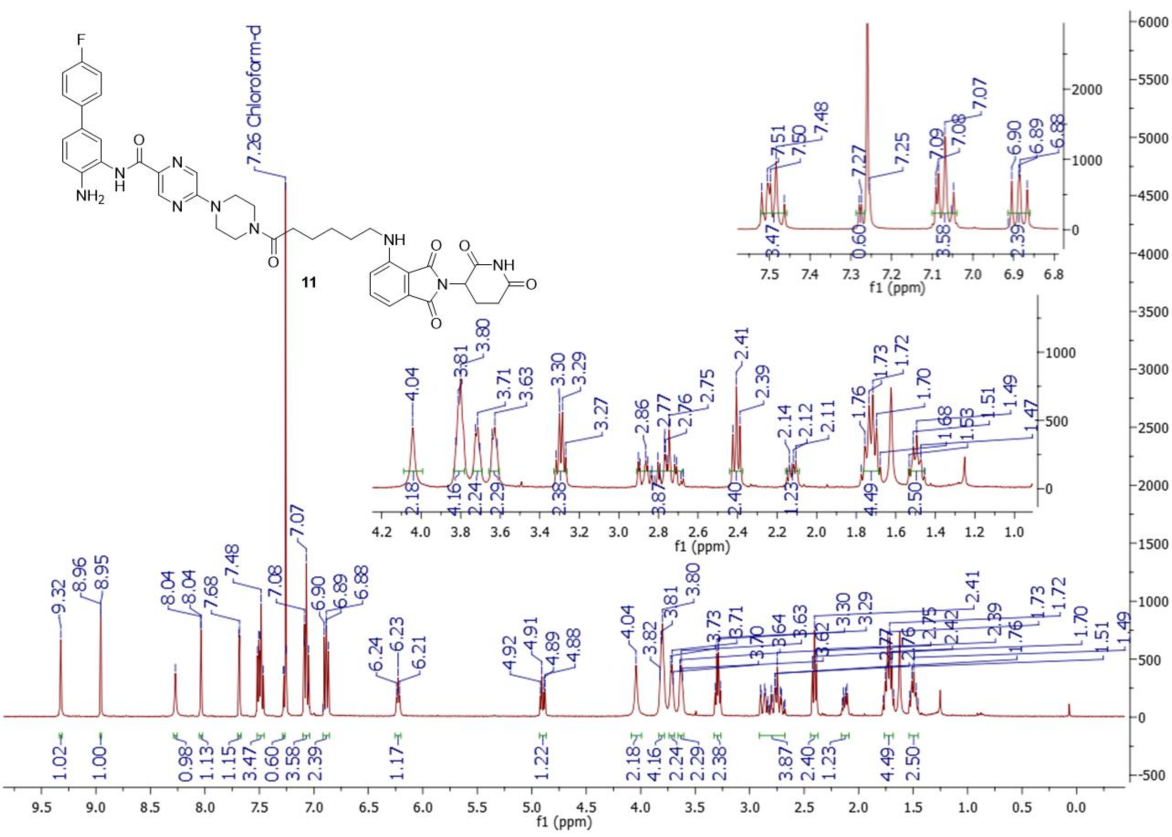

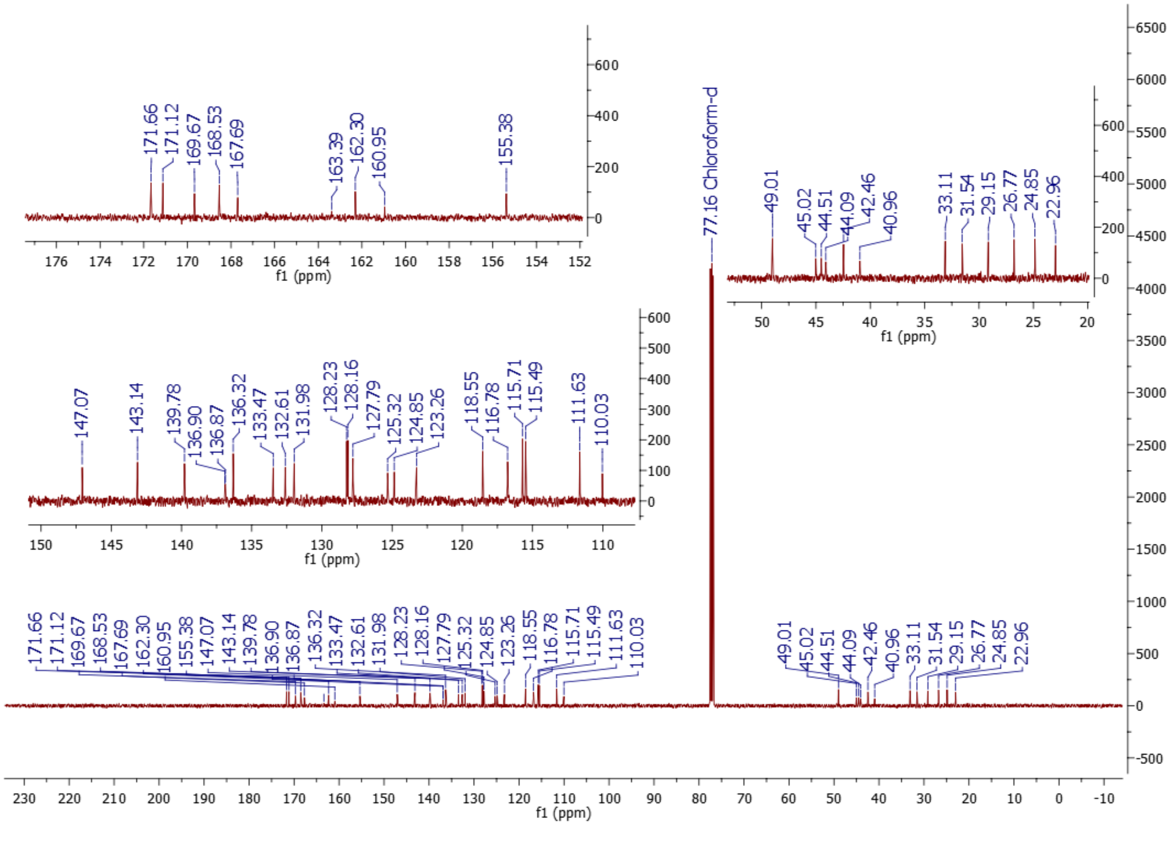

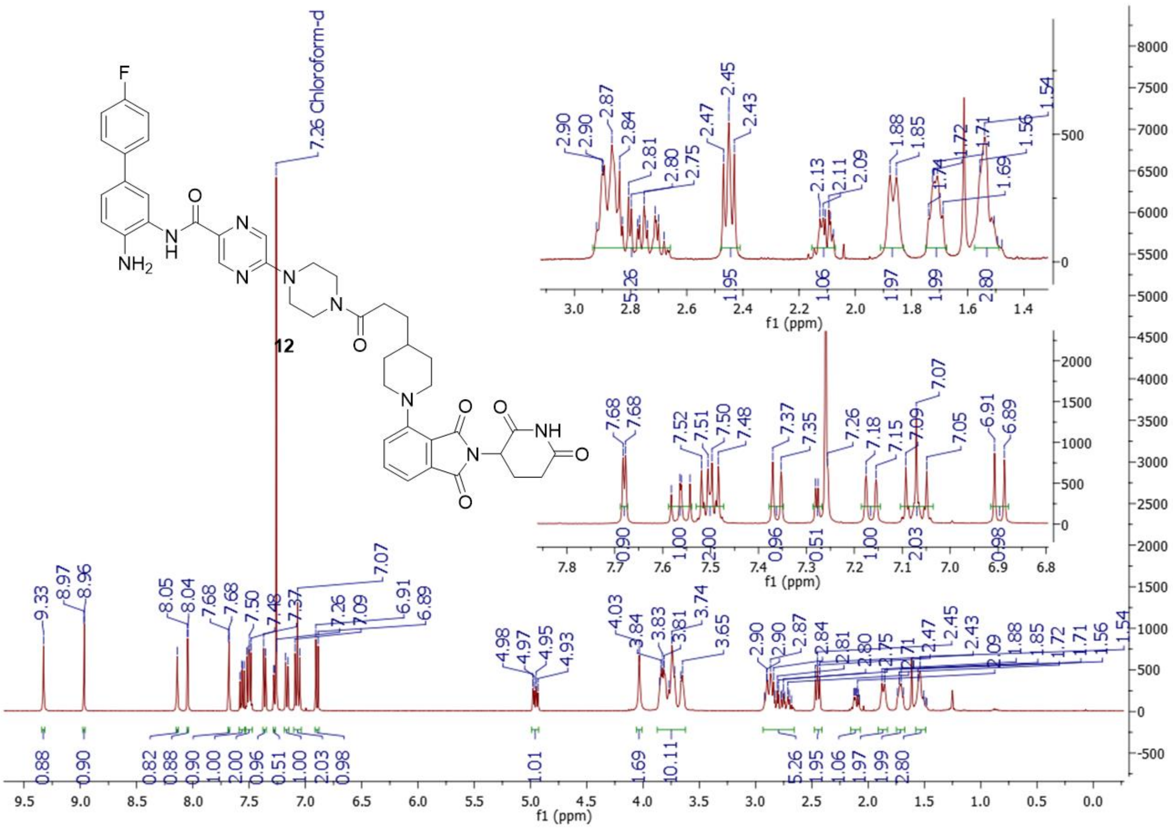
F
O
N
H
NH2
N
N
N
N
O
12
N
O
O
N
O
NH
O
7.26 Chloroform-d
2.90
2.90
2.87
2.84
2.81
2.80
2.75
2.47
2.45
2.43
2.13
2.11
2.09
1.88
1.85
1.74
1.72
1.71
1.69
1.56
1.54
5.26
1.95
1.06
1.97
1.99
2.80
f1 (ppm)
7.68
7.68
7.52
7.51
7.50
7.48
7.37
7.35
7.26
7.18
7.15
7.09
7.07
7.05
6.91
6.89
0.90
1.00
2.00
0.96
0.51
1.00
2.03
0.98
9.33
8.97
8.96
8.05
8.04
4.98
4.97
4.95
4.93
4.03
3.84
3.83
3.81
3.74
3.65
0.88
0.90
0.82
0.88
1.01
1.69
10.11
f1 (ppm)

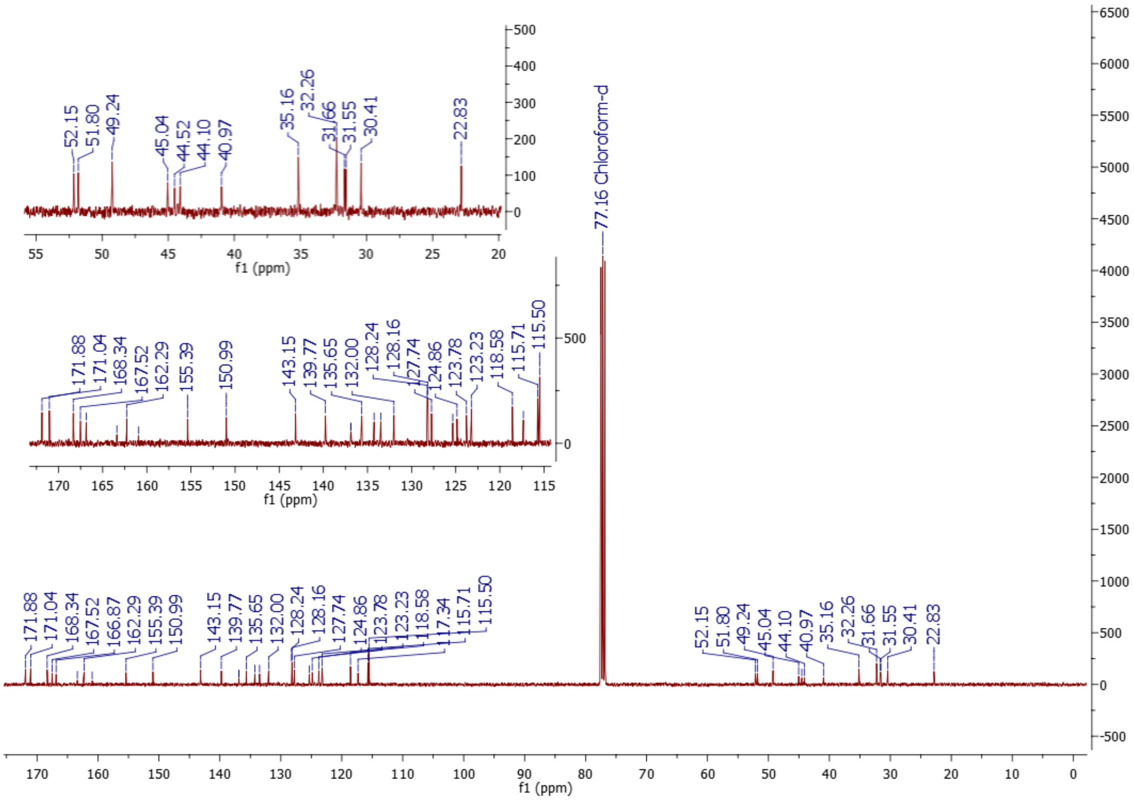
52.15
51.80
49.24
45.04
44.52
44.10
40.97
35.16
32.26
31.66
31.55
30.41
22.83
f1 (ppm)
171.88
171.04
168.34
167.52
162.29
155.39
150.99
143.15
139.77
135.65
132.00
128.24
128.16
127.74
124.86
123.78
123.23
118.58
115.71
115.50
f1 (ppm)
77.16 Chloroform-d
166.87
117.34
f1 (ppm)

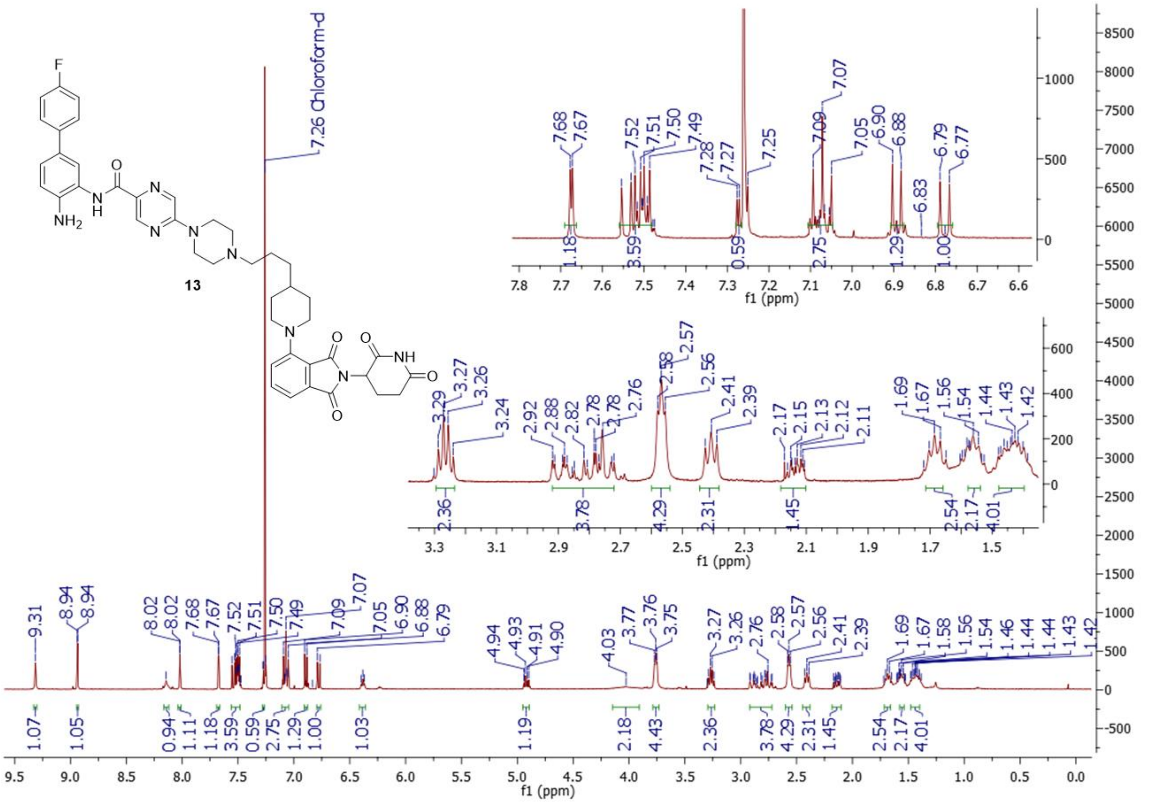

F
O
N
H
N
N
NH2
N
N
N
O
O
NH
O
O
N

13
7.26 Chloroform-d
9.31
8.94
8.94
8.02
8.02
7.68
7.67
7.52
7.51
7.50
7.49
7.09
7.07
7.05
6.90
6.88
6.79
4.94
4.93
4.91
4.90
4.03
3.77
3.76
3.75
3.27
3.26
2.76
2.58
2.57
2.56
2.41
2.39
1.69
1.67
1.58
1.56
1.54
1.46
1.44
1.44
1.43
1.42
1.07
1.05
0.94
1.11
1.18
3.59
0.59
2.75
1.29
1.00
1.03
1.19
2.18
4.43
2.36
3.78
4.29
2.31
1.45
2.54
2.17
4.01
f1 (ppm)

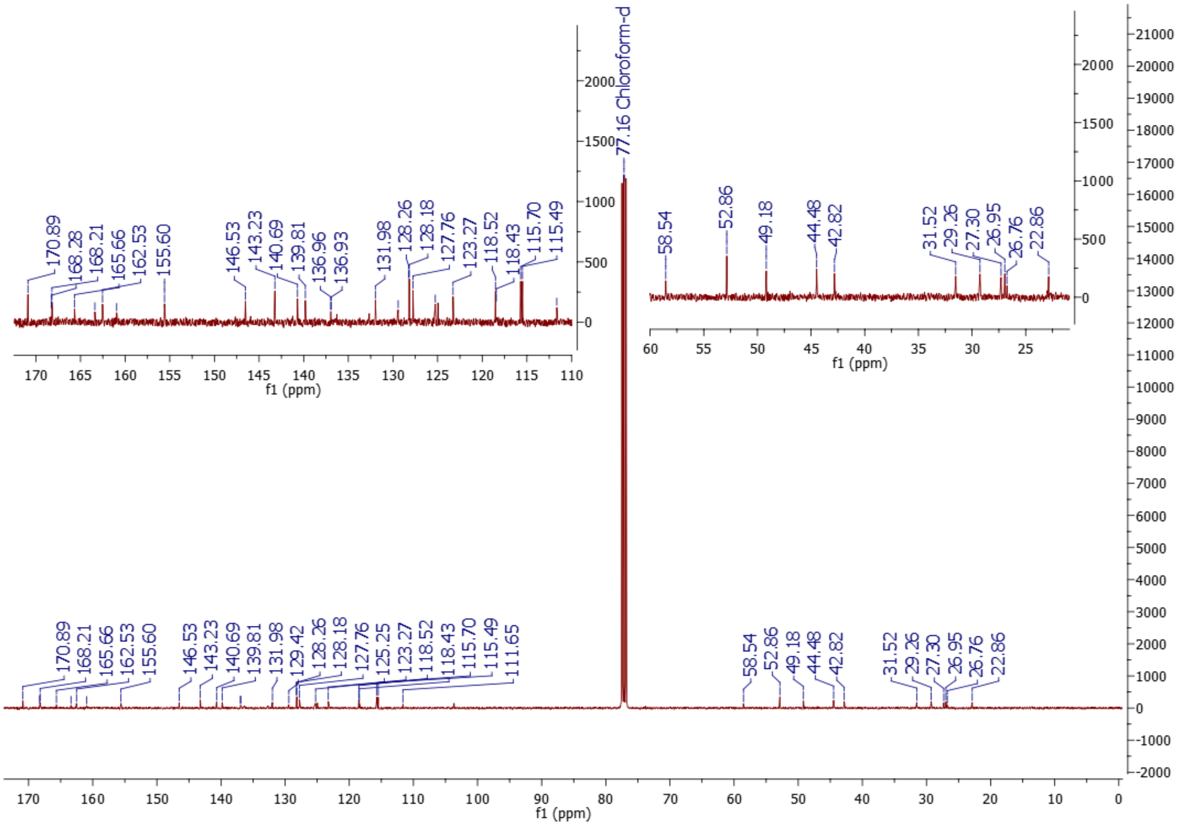

77.16 Chloroform-d
170.89
168.21
165.66
162.53
155.60
146.53
143.23
140.69
139.81
131.98
129.42
128.26
128.18
127.76
125.25
123.27
118.52
118.43
115.70
115.49
111.65
58.54
52.86
49.18
44.48
42.82
31.52
29.26
27.30
26.95
26.76
22.86
f1 (ppm)

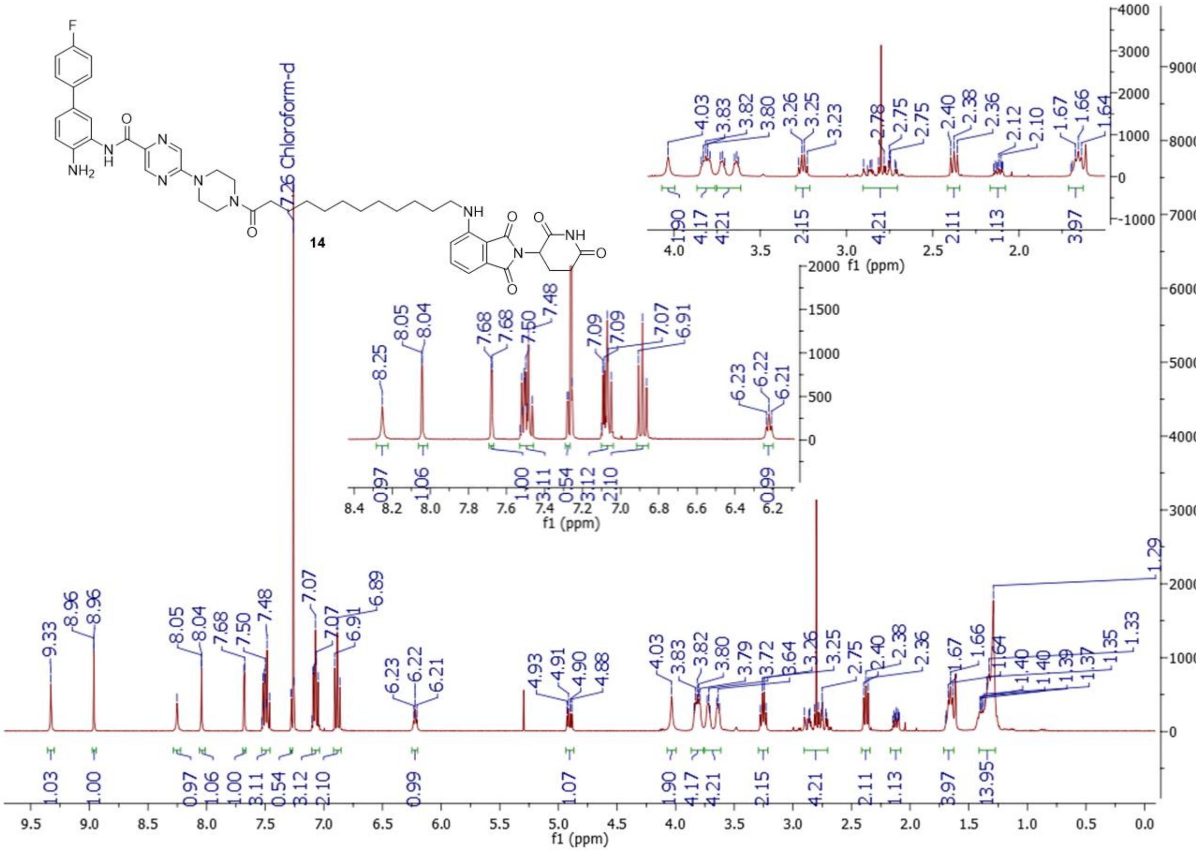

14
7.26 Chloroform-d
f1 (ppm)

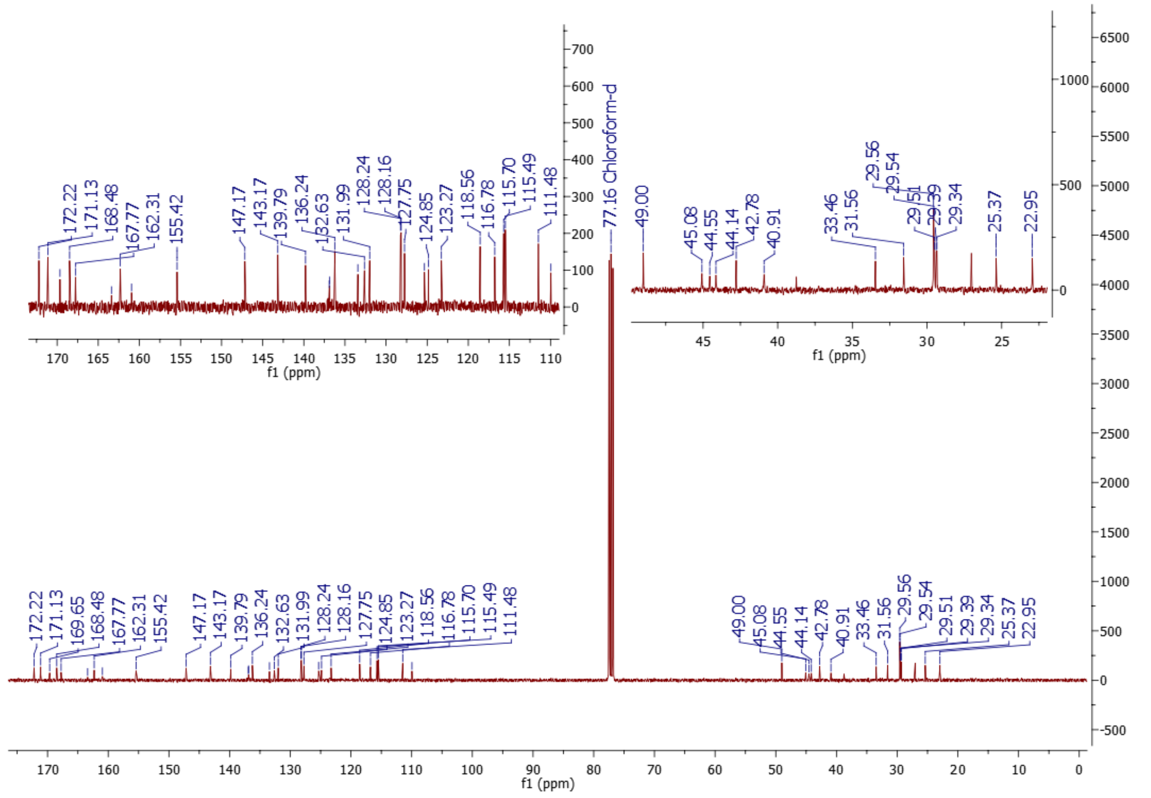

77.16 Chloroform-d
f1 (ppm)

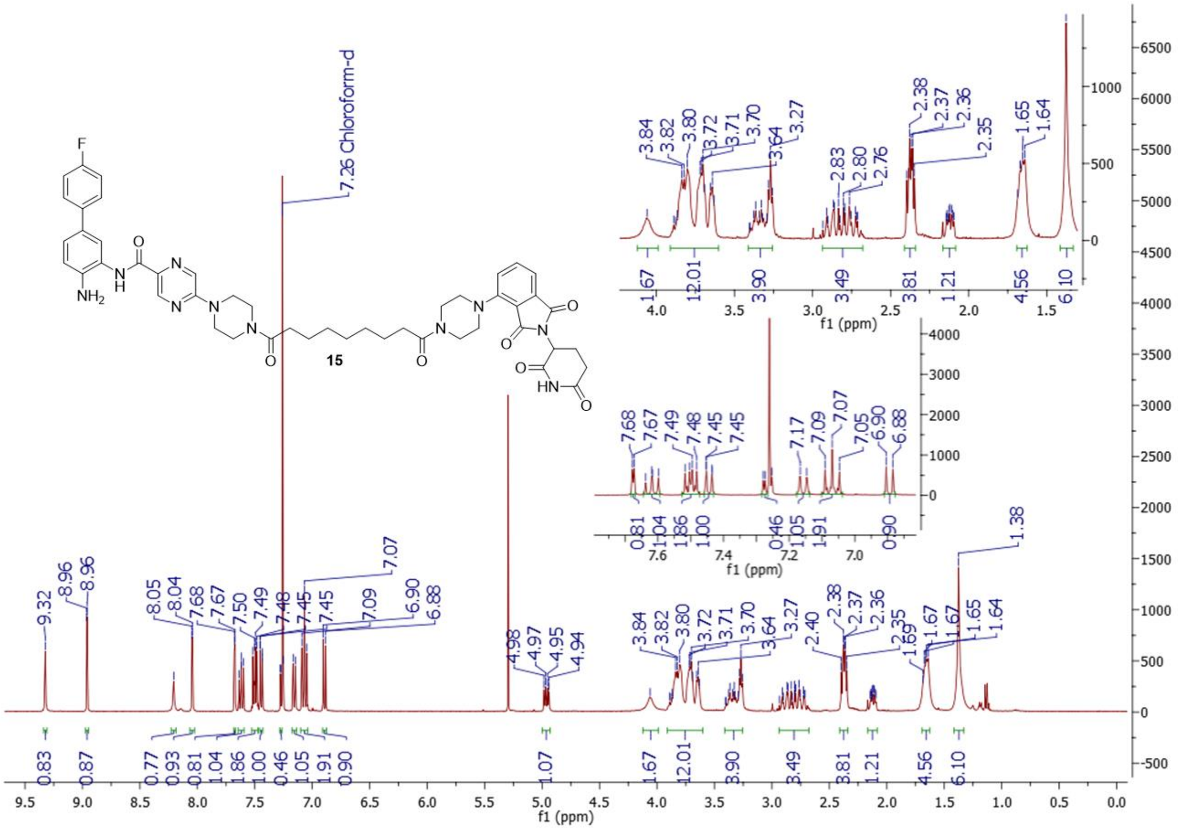
7.26 Chloroform-d
15
f1 (ppm)

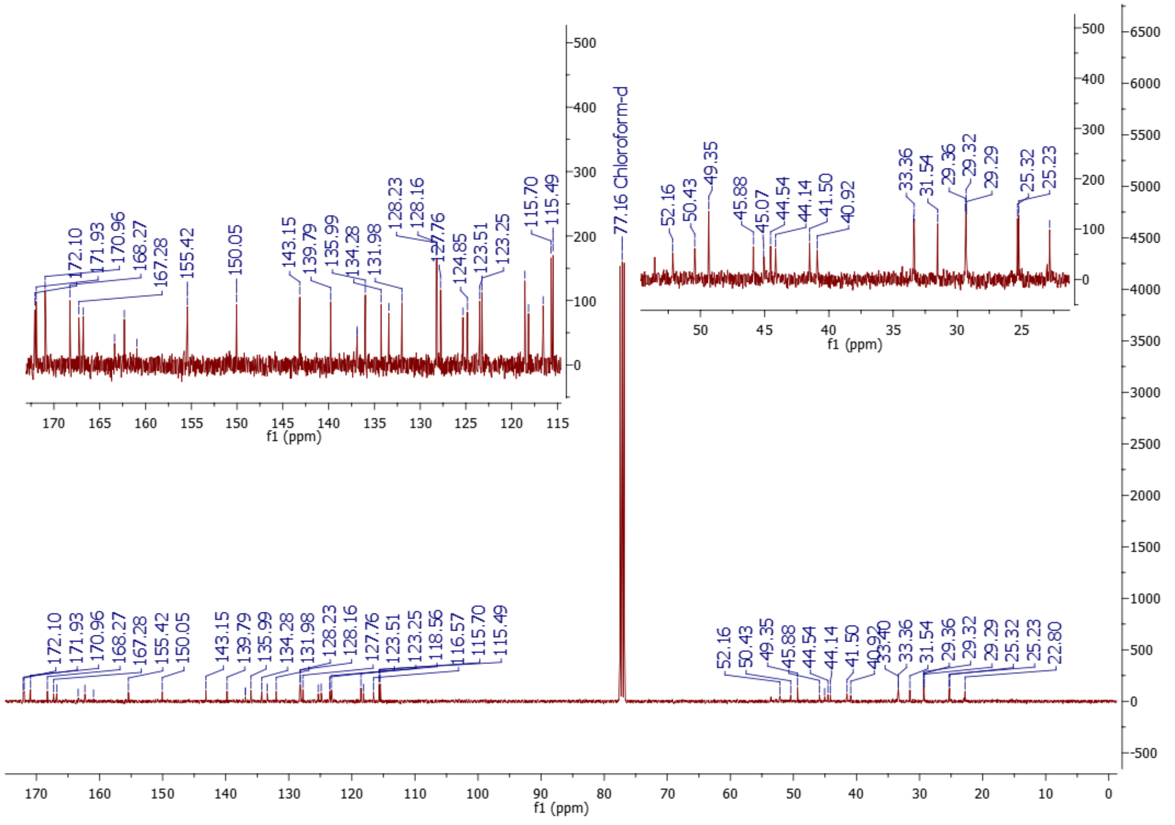
77.16 Chloroform-d
f1 (ppm)

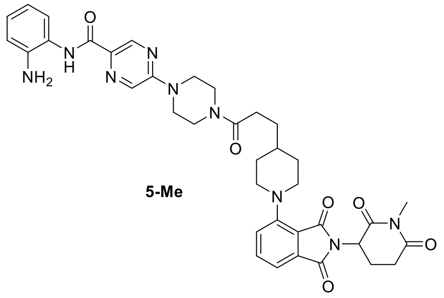

O
N
H
NH2
N
N
N
N
O
N
O
O
O
N
N
O
O

5-Me

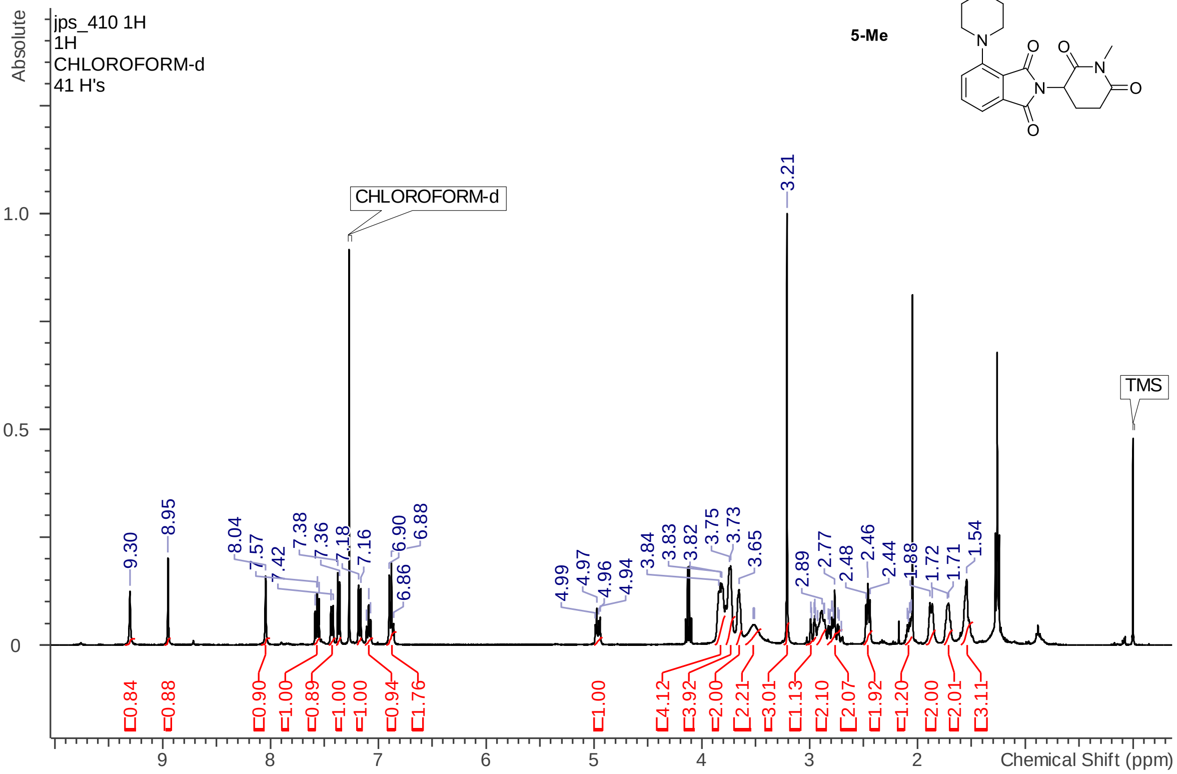

Absolute
jps_410 1H
1H
CHLOROFORM-d
41 H's
CHLOROFORM-d
TMS
9.30
8.95
8.04
7.57
7.42
7.38
7.36
7.18
7.16
6.90
6.88
6.86
4.99
4.97
4.96
4.94
3.84
3.83
3.82
3.75
3.73
3.65
3.21
2.89
2.77
2.48
2.46
2.44
1.88
1.72
1.71
1.54
0.84
0.88
0.90
1.00
0.89
1.00
1.00
0.94
1.76
1.00
4.12
3.92
2.00
2.21
3.01
1.13
2.10
2.07
1.92
1.20
2.00
2.01
3.11
1.0
0.5
0
9
8
7
6
5
4
3
2
Chemical Shift (ppm)

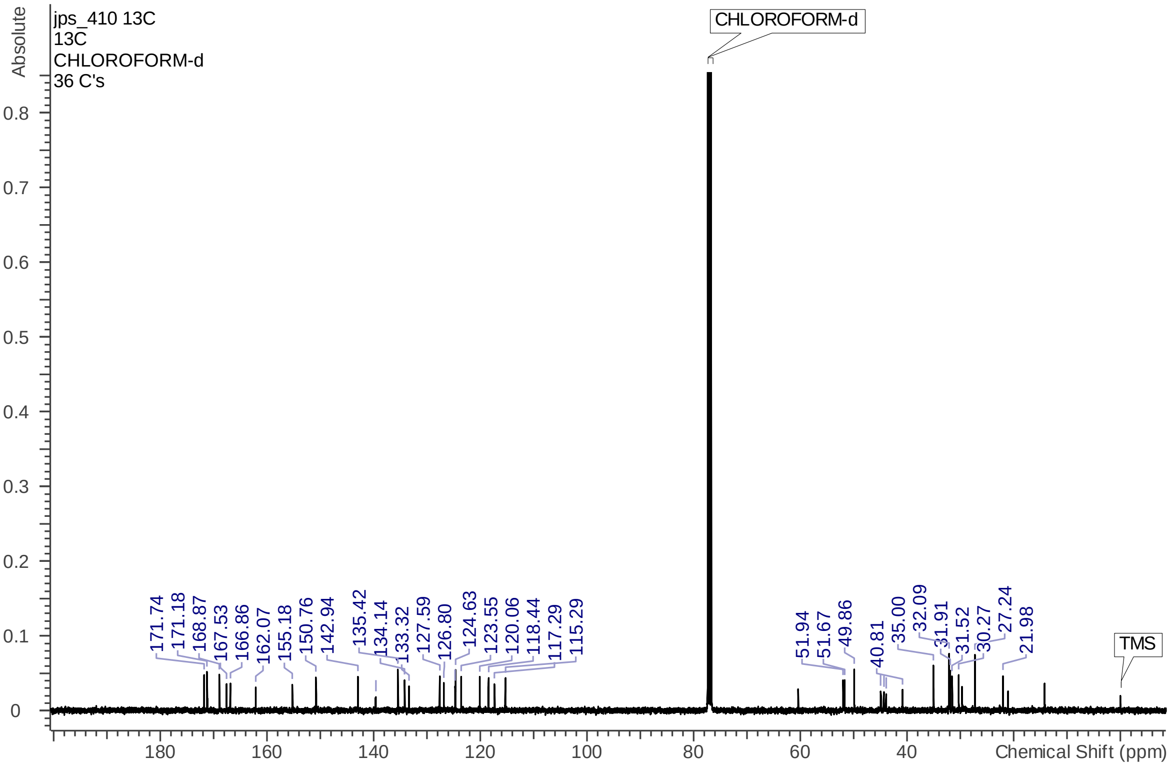

Absolute
jps_410 13C
13C
CHLOROFORM-d
36 C's
CHLOROFORM-d
TMS
171.74
171.18
168.87
167.53
166.86
162.07
155.18
150.76
142.94
135.42
134.14
133.32
127.59
126.80
124.63
123.55
120.06
118.44
117.29
115.29
51.94
51.67
49.86
40.81
35.00
32.09
31.91
31.52
30.27
27.24
21.98
0.8
0.7
0.6
0.5
0.4
0.3
0.2
0.1
0
180
160
140
120
100
80
60
40
Chemical Shift (ppm)

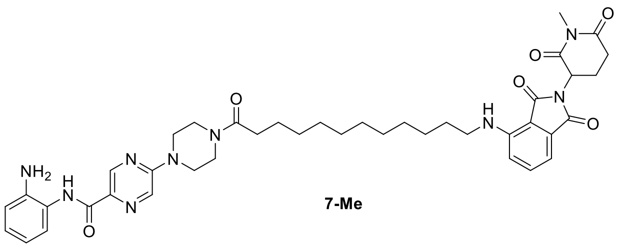
NH2
H
N
N
N
N
N
O
O
O
O
N
O
O
H
N
N
O

7-Me

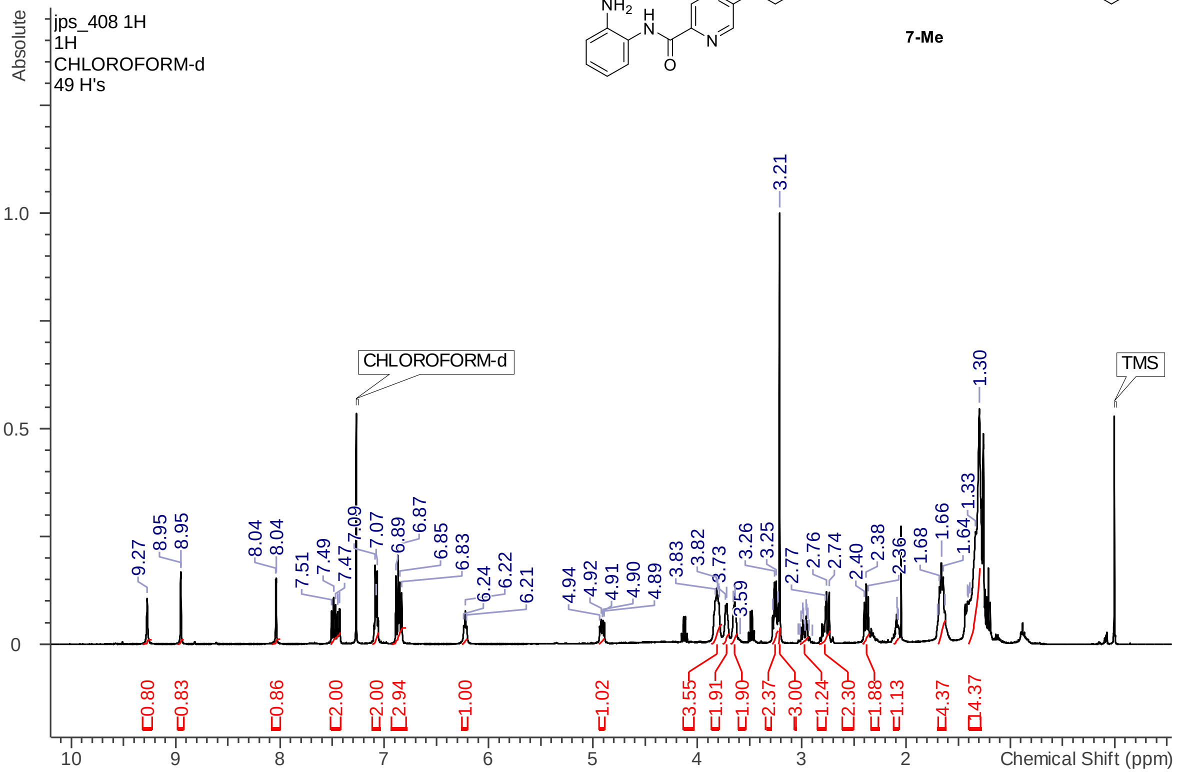
jps_408 1H
1H
CHLOROFORM-d
49 H's
Absolute
1.0
0.5
0
CHLOROFORM-d
TMS
9.27
8.95
8.95
8.04
8.04
7.51
7.49
7.47
7.09
7.07
6.89
6.87
6.85
6.83
6.24
6.22
6.21
4.94
4.92
4.91
4.90
4.89
3.83
3.82
3.73
3.59
3.26
3.25
3.21
2.77
2.76
2.74
2.40
2.38
2.36
1.68
1.66
1.64
1.33
1.30
0.80
0.83
0.86
2.00
2.00
2.94
1.00
1.02
3.55
1.91
1.90
2.37
3.00
1.24
2.30
1.88
1.13
4.37
14.37
10
9
8
7
6
5
4
3
2
Chemical Shift (ppm)

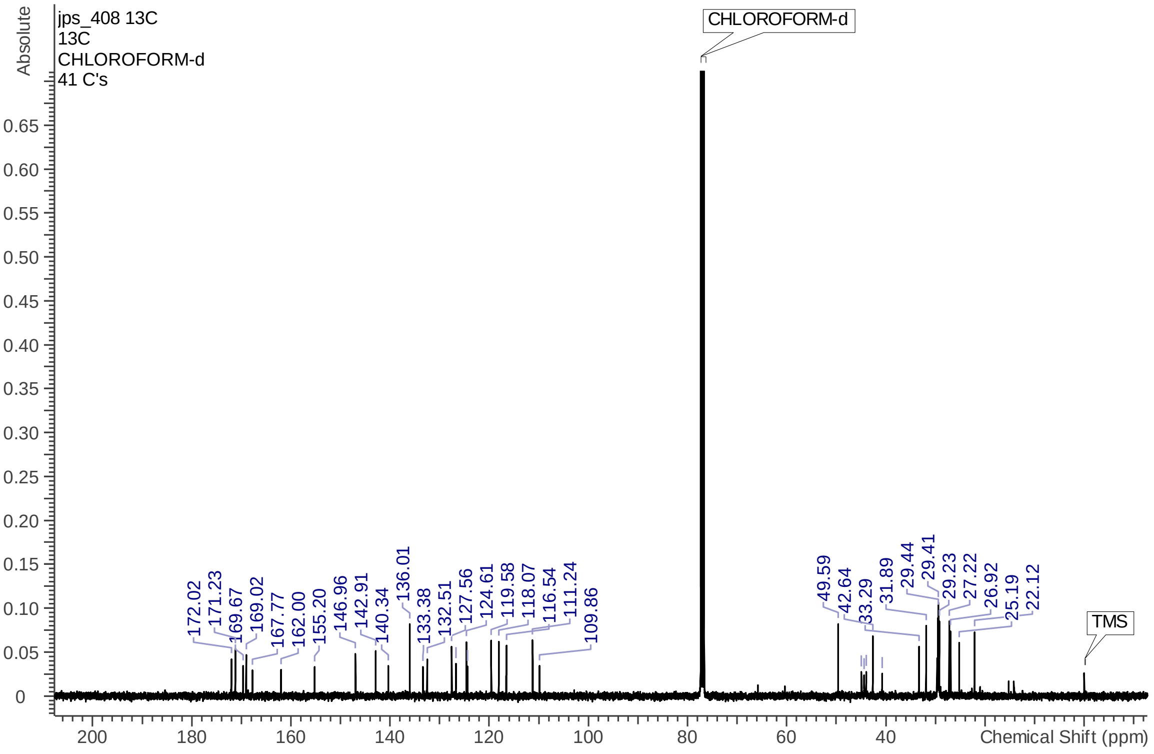
jps_408 13C
13C
CHLOROFORM-d
41 C's
Absolute
0.65
0.60
0.55
0.50
0.45
0.40
0.35
0.30
0.25
0.20
0.15
0.10
0.05
0
CHLOROFORM-d
TMS
172.02
171.23
169.67
169.02
167.77
162.00
155.20
146.96
142.91
140.34
136.01
133.38
132.51
127.56
124.61
119.58
118.07
116.54
111.24
109.86
49.59
42.64
33.29
31.89
29.44
29.41
29.23
27.22
26.92
25.19
22.12
200
180
160
140
120
100
80
60
40
Chemical Shift (ppm)

Appendix: Analytical HPLC

Compound 7

ARP-26

Sample Name:	**ARP-26**	*Injection Volume:*	**20.0**
Vial Number:	**BB1**	*Channel:*	**UV_VIS_1**
Sample Type:	**unknown**	*Wavelength:*	**214.0**
Control Program:	**5-100%B, 30 min gradiant, 45 min run unbu**	*Bandwidth:*	**2**
Quantif. Method:	**processing parameters**	*Dilution Factor:*	**1.0000**
Recording Time:	**23/10/2024 16:02**	*Sample Weight:*	**1.0000**
Run Time (min):	**45.00**	*Sample Amount:*	**1.0000**

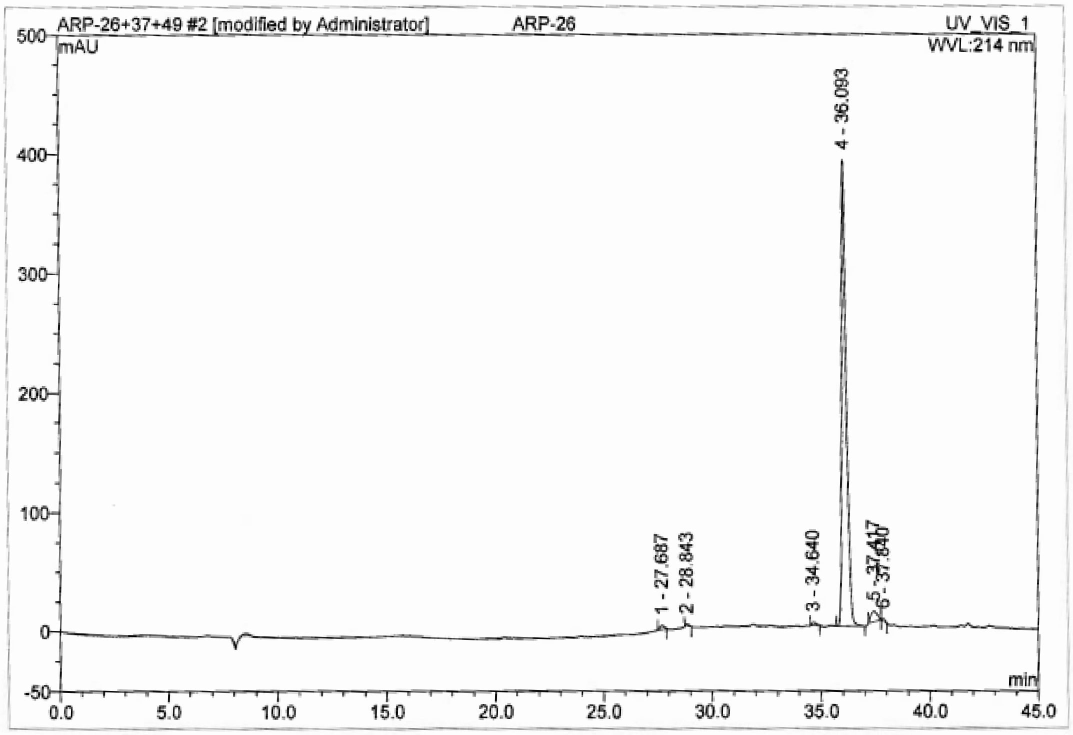

No.	Ret.Time min	Peak Name	Height mAU	Area mAU*min	Rel.Area %	Amount	Type
1	27.69	n.a.	3.426	0.705	0.71	n.a.	BMB*
2	28.84	n.a.	1.966	0.353	0.36	n.a.	BMB*
3	34.64	n.a.	2.924	0.658	0.66	n.a.	MB*
4	36.09	n.a.	390.211	94.220	95.15	n.a.	MB*
5	37.42	n.a.	8.181	2.828	2.86	n.a.	BMb*
6	37.84	n.a.	1.459	0.256	0.26	n.a.	bMB*
**Total:**			408.167	99.020	100.00	0.000	

Compound 5

ARP-37

Sample Name:	**ARP-37**	*Injection Volume:*	**20.0**
Vial Number:	**BB2**	*Channel:*	**UV_VIS_1**
Sample Type:	**unknown**	*Wavelength:*	**214.0**
Control Program:	**5-100%B, 30 min gradiant, 45 min run unbu**	*Bandwidth:*	**2**
Quantif. Method:	**processing parameters**	*Dilution Factor:*	**1.0000**
Recording Time:	**23/10/2024 16:48**	*Sample Weight:*	**1.0000**
Run Time (min):	**45.00**	*Sample Amount:*	**1.0000**

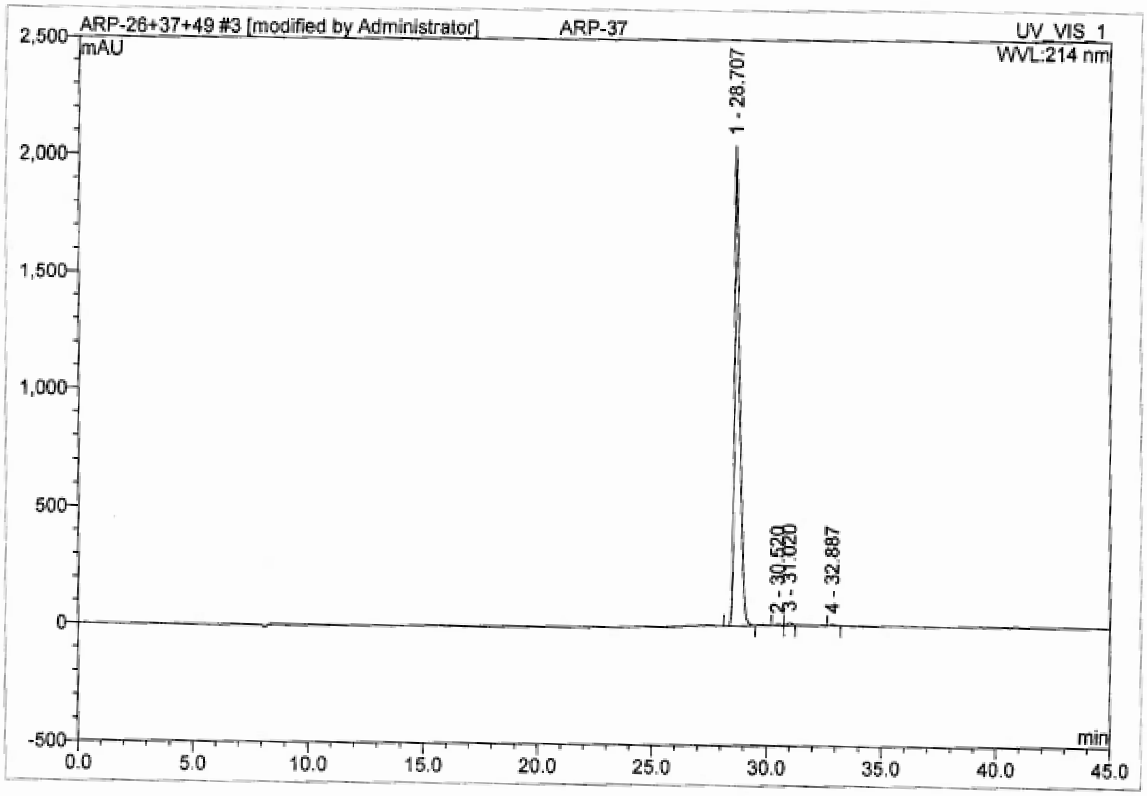

No.	Ret.Time min	Peak Name	Height mAU	Area mAU*min	Rel.Area %	Amount	Type
1	28.71	n.a.	2039.968	531.090	99.06	n.a.	BMB*
2	30.52	n.a.	4.538	1.072	0.20	n.a.	BMB
3	31.02	n.a.	10.941	2.439	0.45	n.a.	BMB
4	32.89	n.a.	6.934	1.526	0.28	n.a.	BMB
**Total:**			2062.381	536.127	100.00	0.000	

Compound 12

ARP-49

Sample Name:	**ARP-49**	*Injection Volume:*	**20.0**
Vial Number:	**BB3**	*Channel:*	**UV_VIS_1**
Sample Type:	**unknown**	*Wavelength:*	**214.0**
Control Program:	**5-100%B, 30 min gradiant, 45 min run unbu**	*Bandwidth:*	**2**
Quantif. Method:	**processing parameters**	*Dilution Factor:*	**1.0000**
Recording Time:	**23/10/2024 17:34**	*Sample Weight:*	**1.0000**
Run Time (min):	**45.00**	*Sample Amount:*	**1.0000**

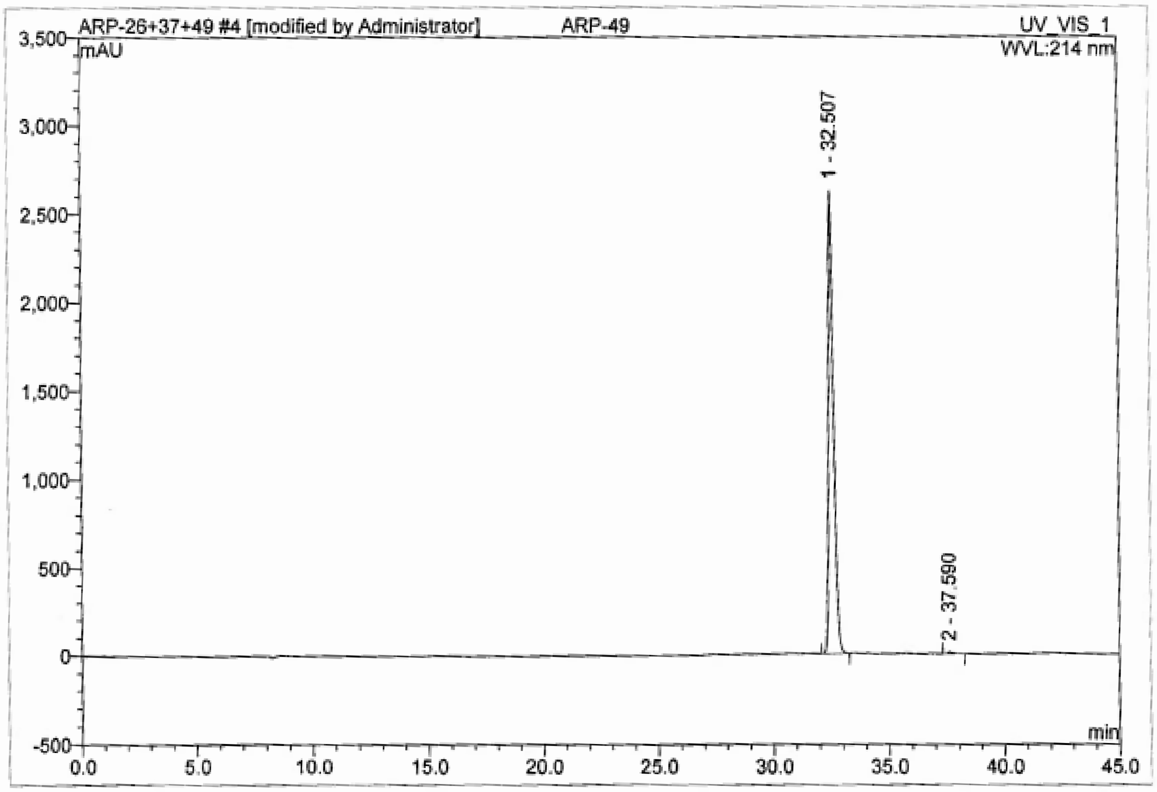

No.	Ret.Time min	Peak Name	Height mAU	Area mAU*min	Rel.Area %	Amount	Type
1	32.51	n.a.	2618.663	667.757	99.60	n.a.	BMB*
2	37.59	n.a.	9.976	2.683	0.40	n.a.	BMB
**Total:**			2628.639	670.439	100.00	0.000	

Appendix: Full Western Blots

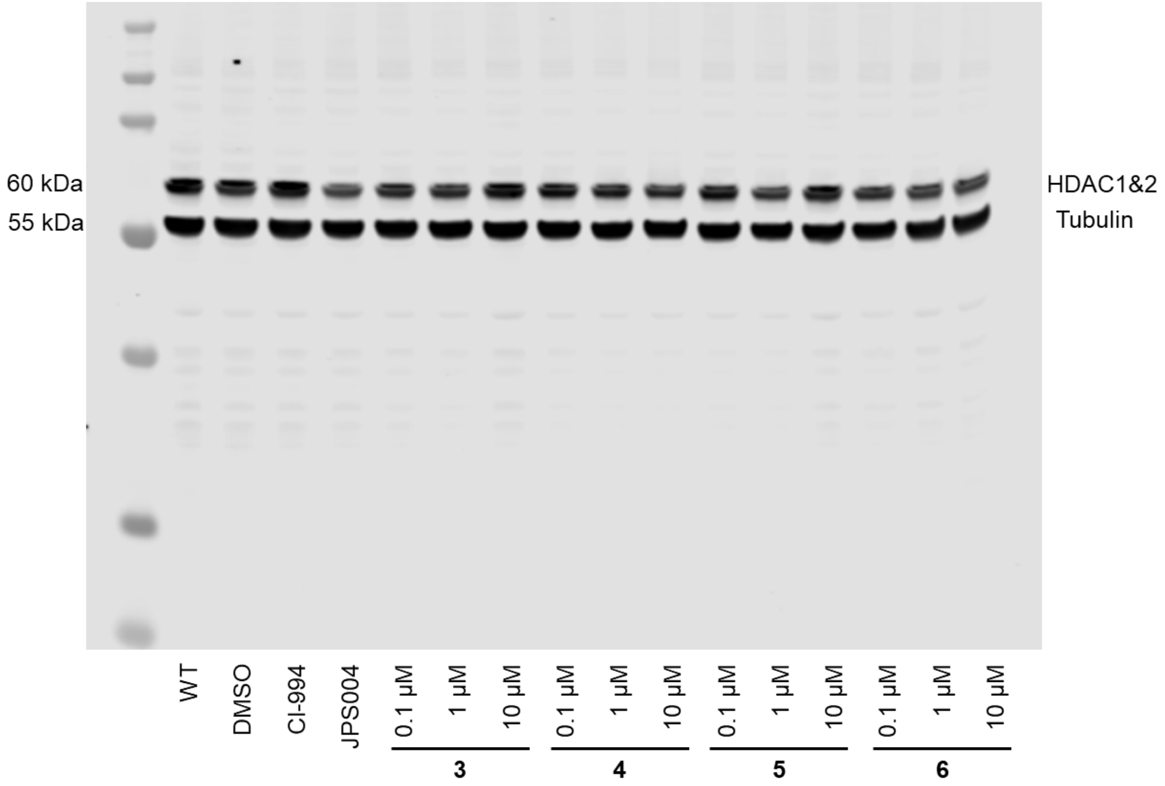

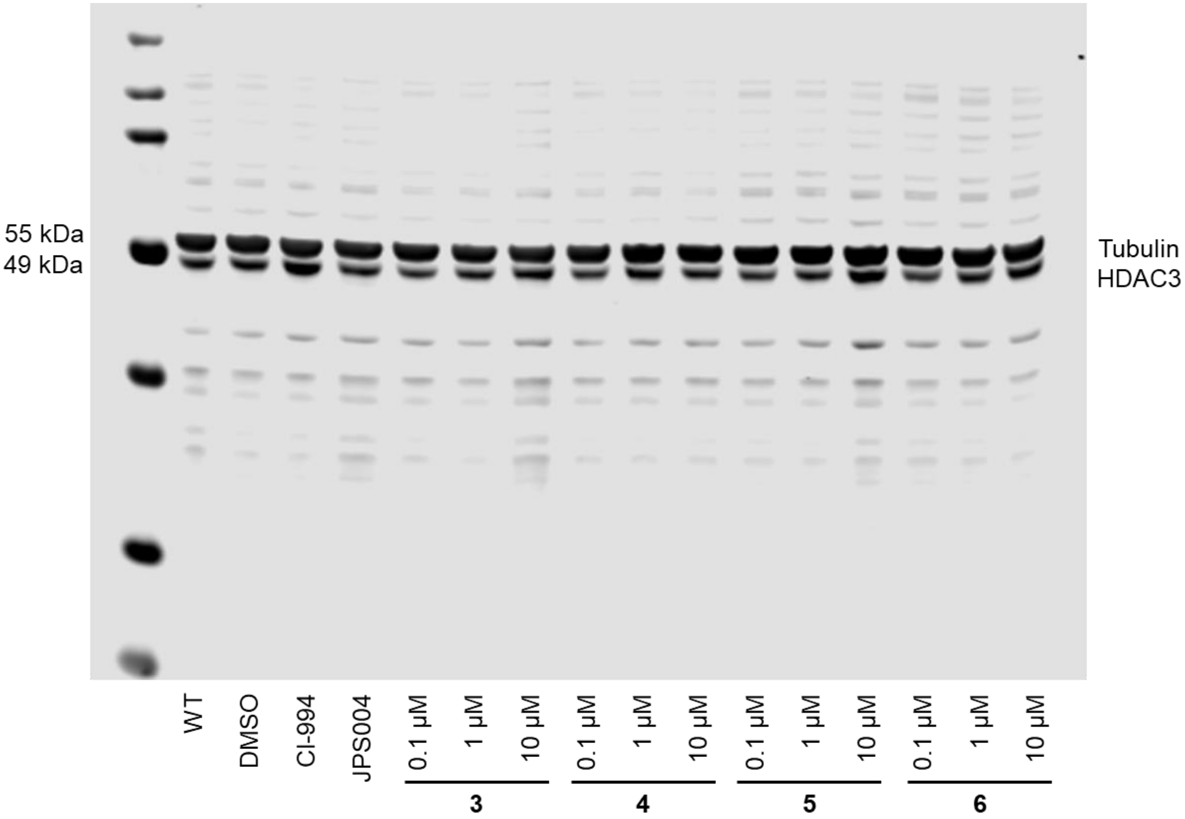

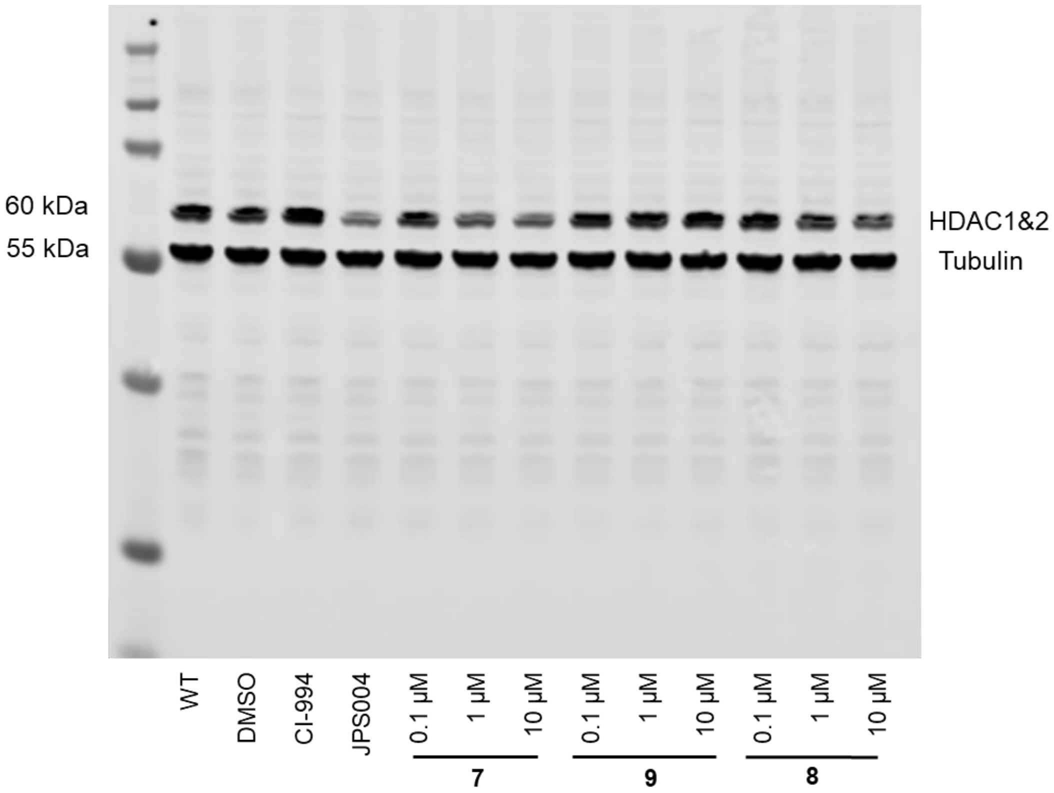
60 kDa
55 kDa
HDAC1&2
Tubulin
WT
DMSO
CI-994
JPS004
0.1 µM
1 µM
10 µM
0.1 µM
1 µM
10 µM
0.1 µM
1 µM
10 µM
7
9
8

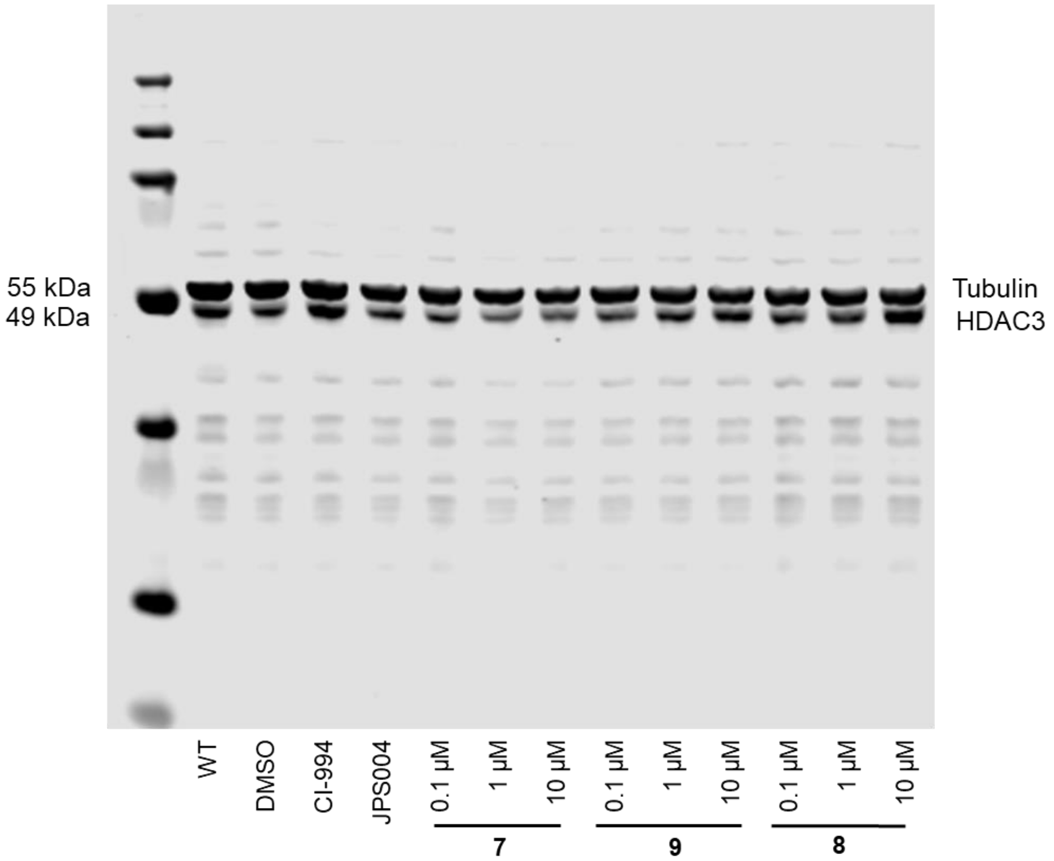
55 kDa
49 kDa
Tubulin
HDAC3
WT
DMSO
CI-994
JPS004
0.1 µM
1 µM
10 µM
0.1 µM
1 µM
10 µM
0.1 µM
1 µM
10 µM
7
9
8

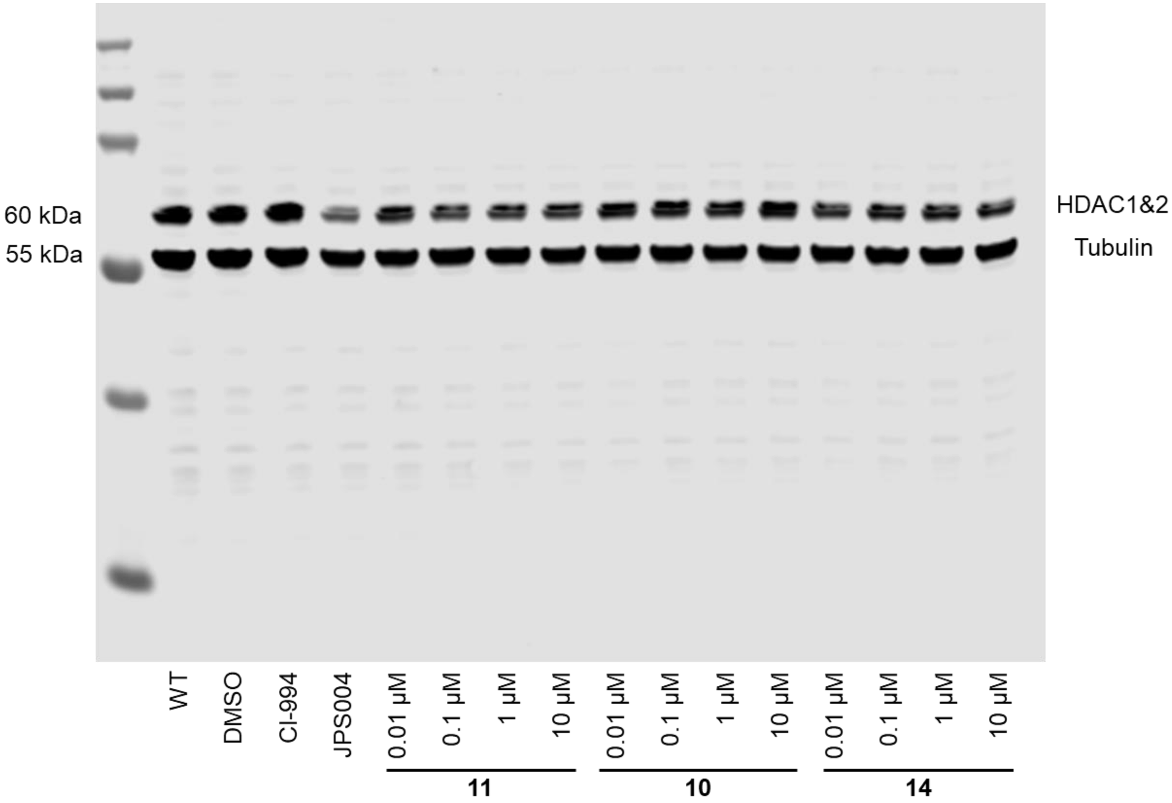
60 kDa
55 kDa
HDAC1&2
Tubulin
WT
DMSO
CI-994
JPS004
0.01 µM
0.1 µM
1 µM
10 µM
0.01 µM
0.1 µM
1 µM
10 µM
0.01 µM
0.1 µM
1 µM
10 µM
11
10
14

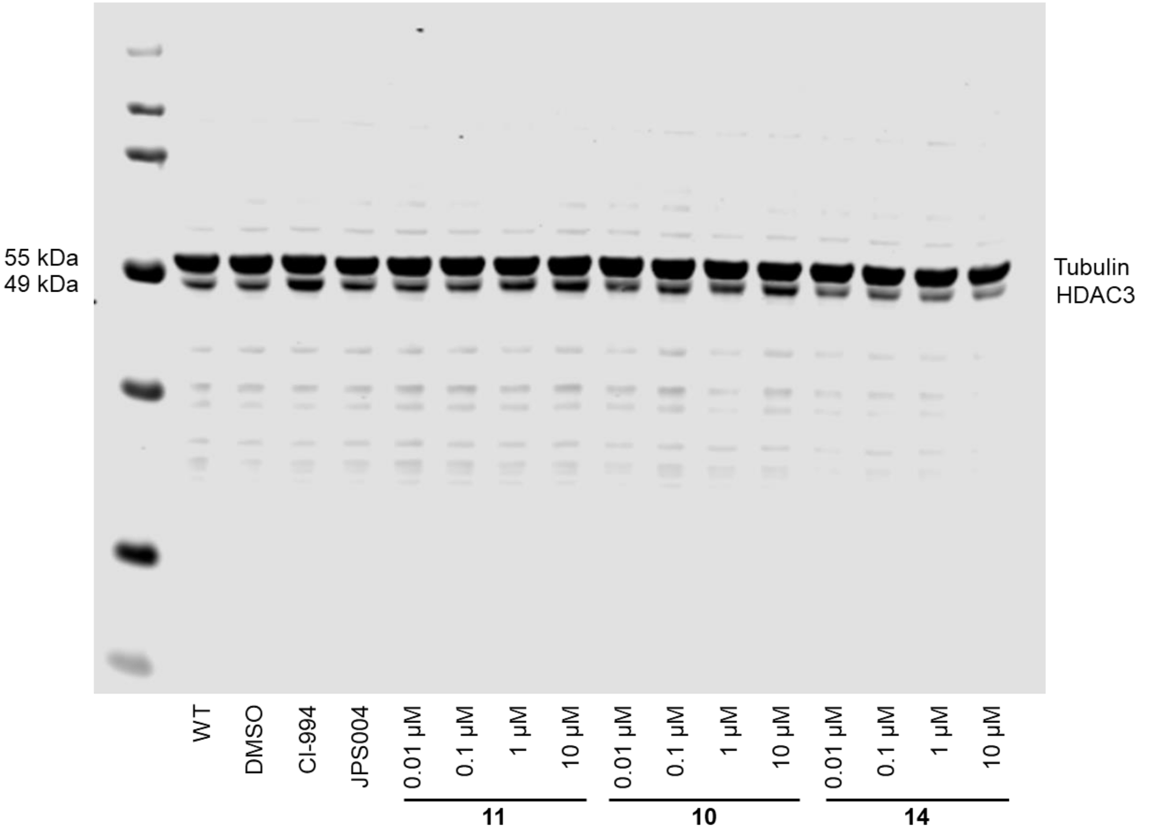
55 kDa
49 kDa
Tubulin
HDAC3
WT
DMSO
CI-994
JPS004
0.01 µM
0.1 µM
1 µM
10 µM
0.01 µM
0.1 µM
1 µM
10 µM
0.01 µM
0.1 µM
1 µM
10 µM
11
10
14

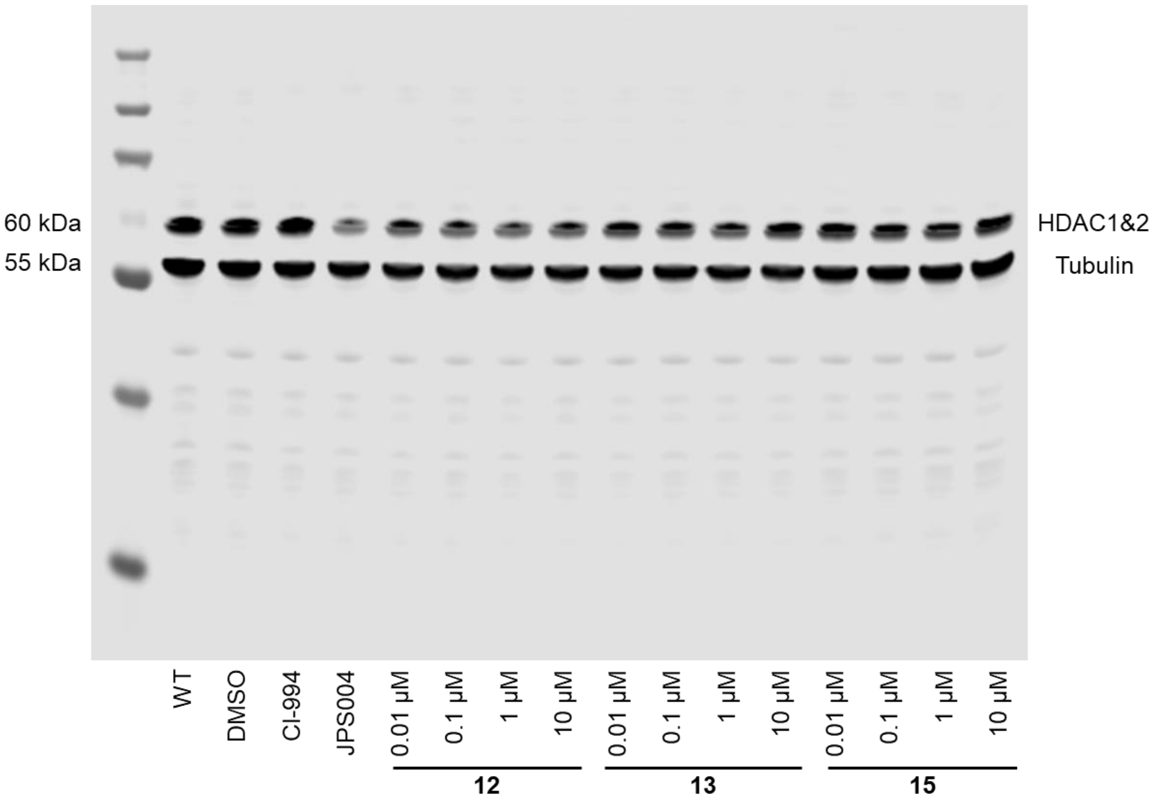
60 kDa
55 kDa
HDAC1&2
Tubulin
WT
DMSO
CI-994
JPS004
0.01 µM
0.1 µM
1 µM
10 µM
0.01 µM
0.1 µM
1 µM
10 µM
0.01 µM
0.1 µM
1 µM
10 µM
12
13
15

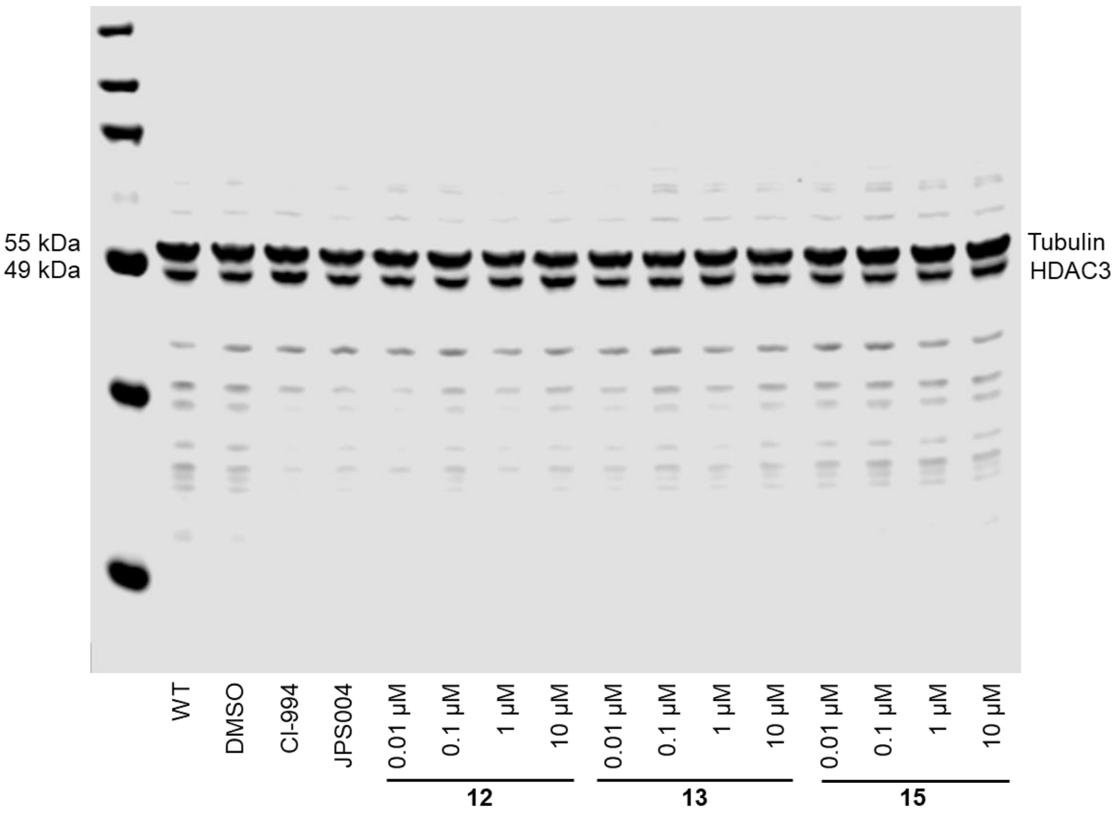
55 kDa
49 kDa
Tubulin
HDAC3
WT
DMSO
CI-994
JPS004
0.01 µM
0.1 µM
1 µM
10 µM
0.01 µM
0.1 µM
1 µM
10 µM
0.01 µM
0.1 µM
1 µM
10 µM
12
13
15

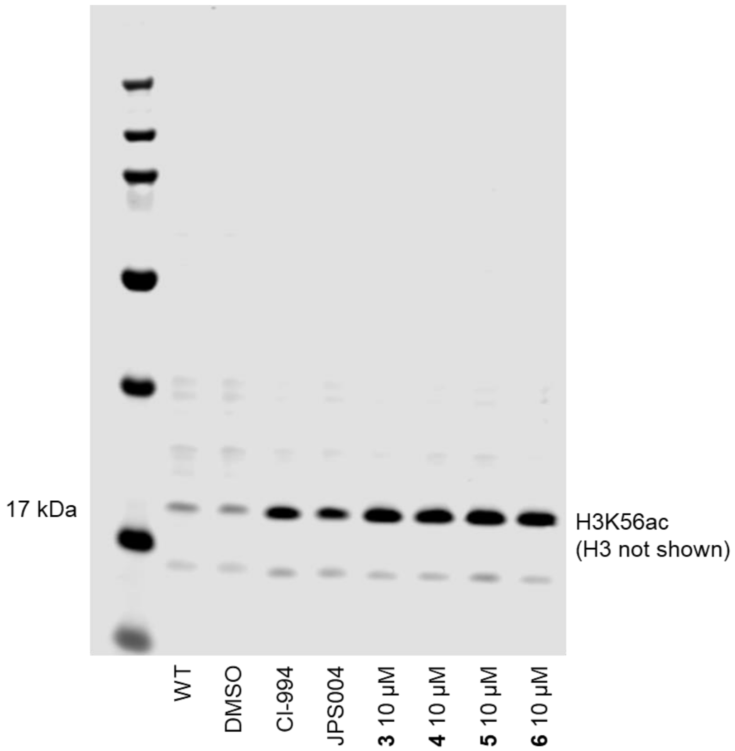
17 kDa
H3K56ac
(H3 not shown)
WT
DMSO
CI-994
JPS004
3 10 µM
4 10 µM
5 10 µM
6 10 µM

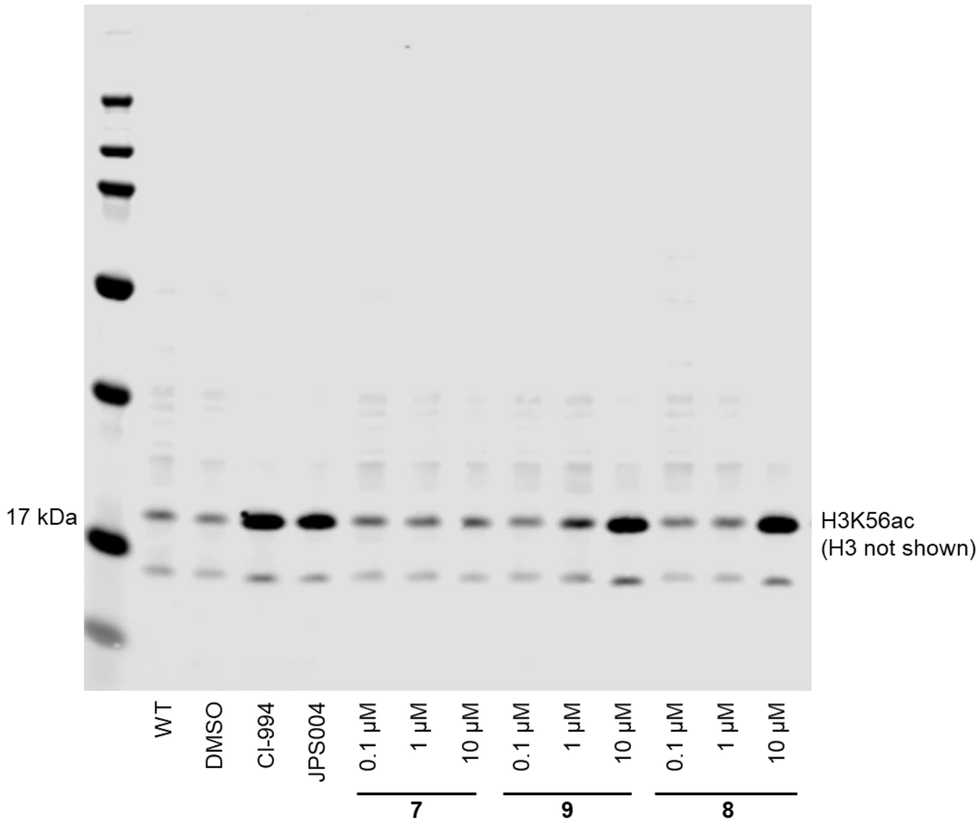
17 kDa
H3K56ac
(H3 not shown)
WT
DMSO
CI-994
JPS004
0.1 µM
1 µM
10 µM
0.1 µM
1 µM
10 µM
0.1 µM
1 µM
10 µM
7
9
8

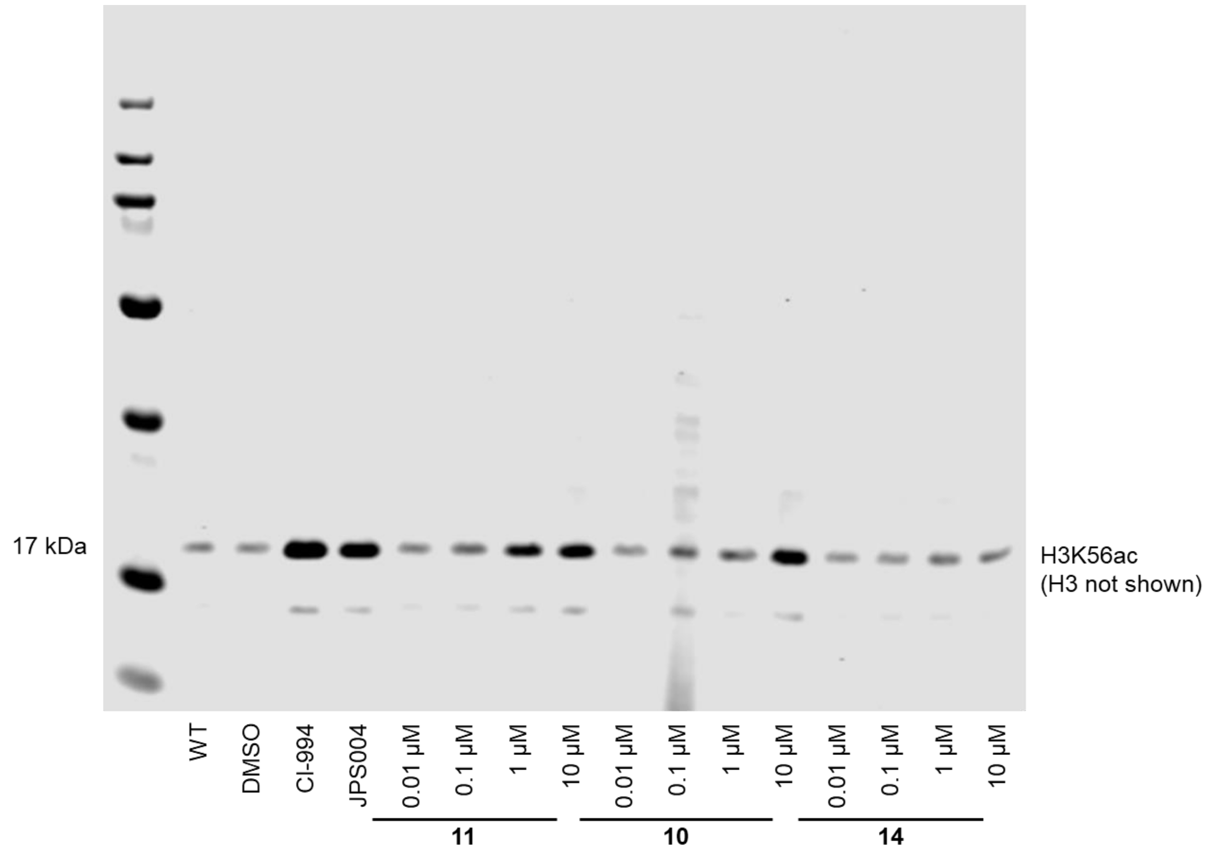
17 kDa
H3K56ac
(H3 not shown)
WT
DMSO
CI-994
JPS004
0.01 µM
0.1 µM
1 µM
10 µM
0.01 µM
0.1 µM
1 µM
10 µM
0.01 µM
0.1 µM
1 µM
10 µM
11
10
14

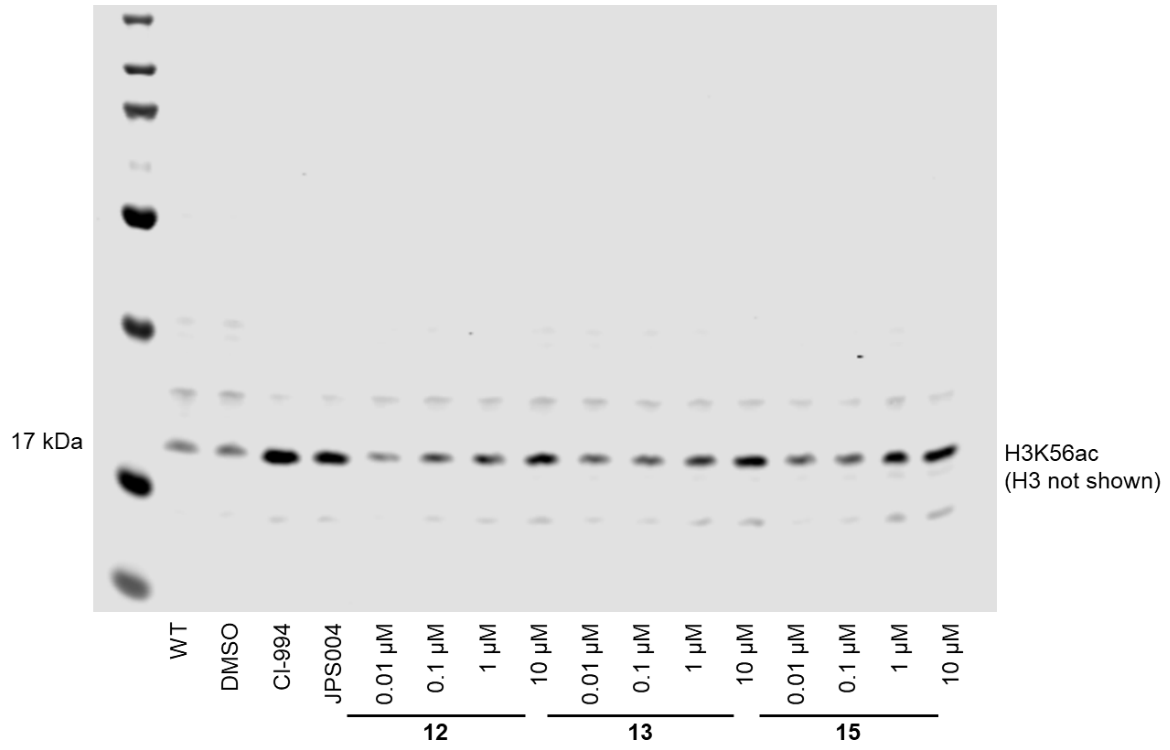
17 kDa
H3K56ac
(H3 not shown)
WT
DMSO
CI-994
JPS004
0.01 µM
0.1 µM
1 µM
10 µM
0.01 µM
0.1 µM
1 µM
10 µM
0.01 µM
0.1 µM
1 µM
10 µM
12
13
15

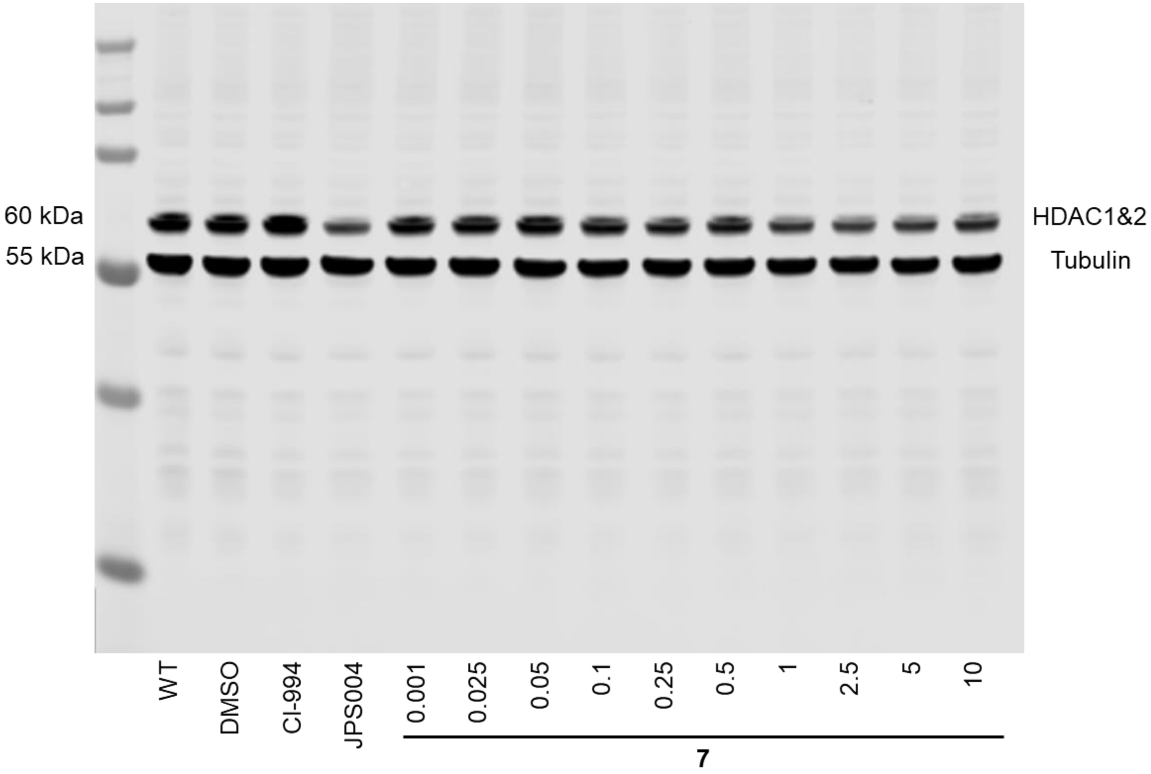
60 kDa
55 kDa
HDAC1&2
Tubulin
WT
DMSO
CI-994
JPS004
0.001
0.025
0.05
0.1
0.25
0.5
1
2.5
5
10
7

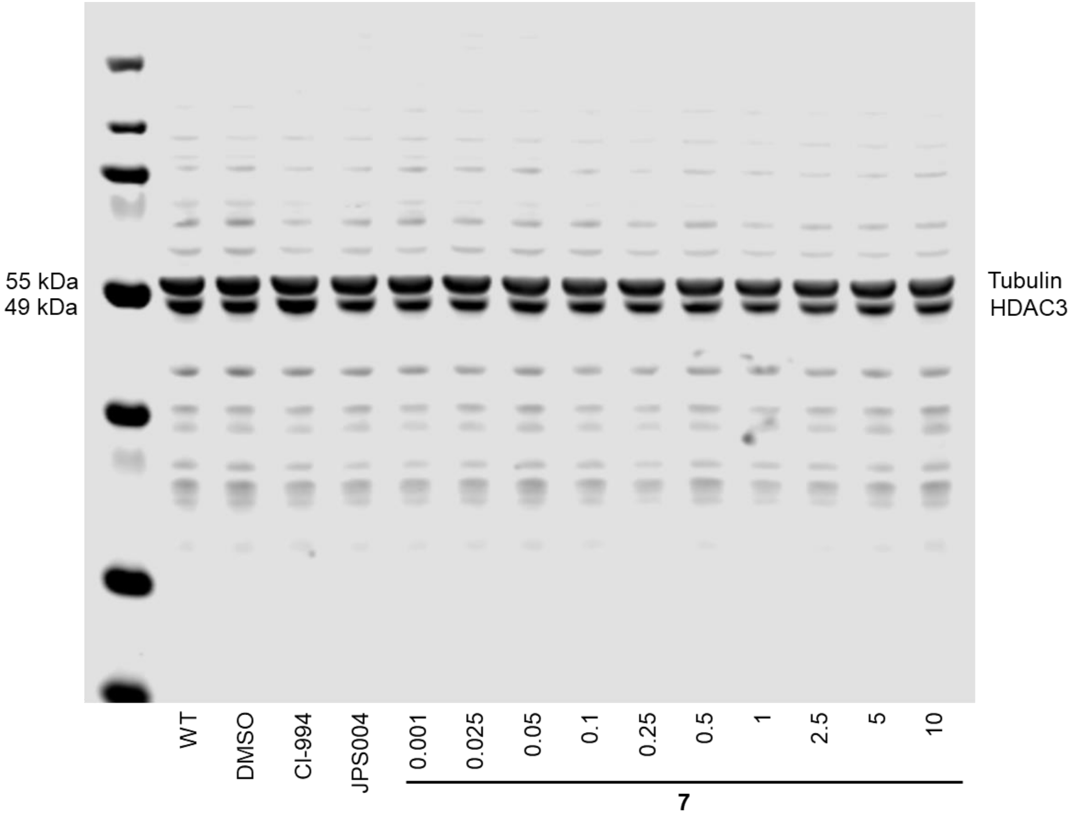
55 kDa
49 kDa
Tubulin
HDAC3
WT
DMSO
CI-994
JPS004
0.001
0.025
0.05
0.1
0.25
0.5
1
2.5
5
10
7

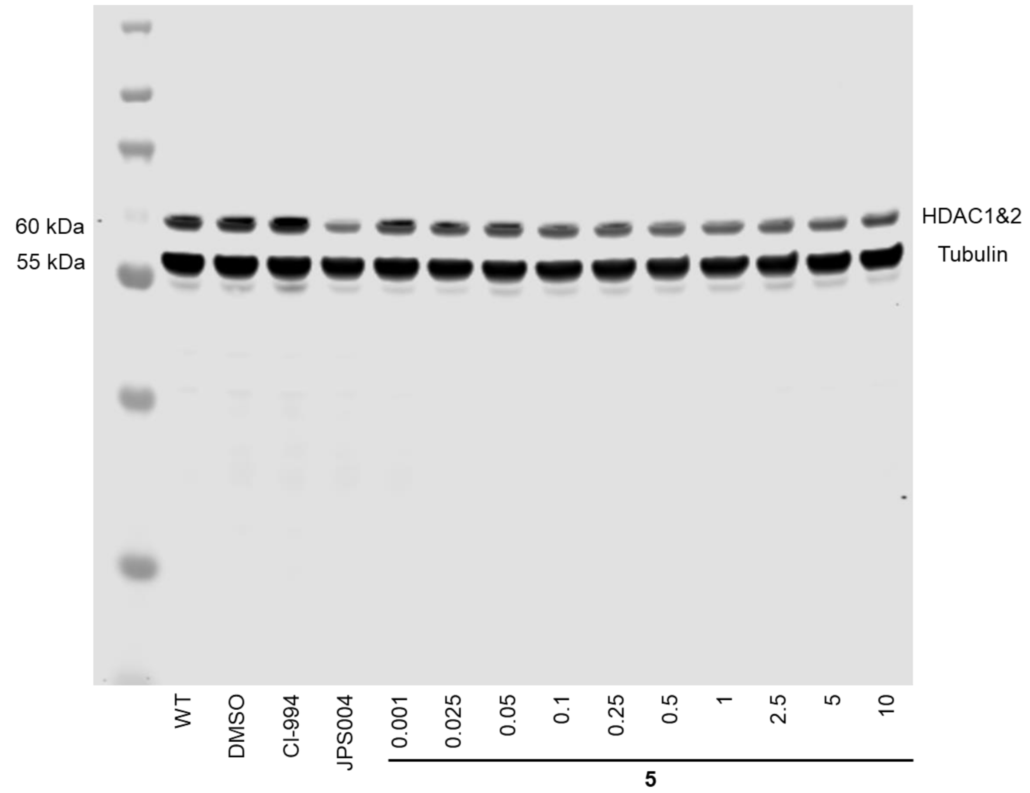
60 kDa
55 kDa
HDAC1&2
Tubulin
WT
DMSO
CI-994
JPS004
0.001
0.025
0.05
0.1
0.25
0.5
1
2.5
5
10
5

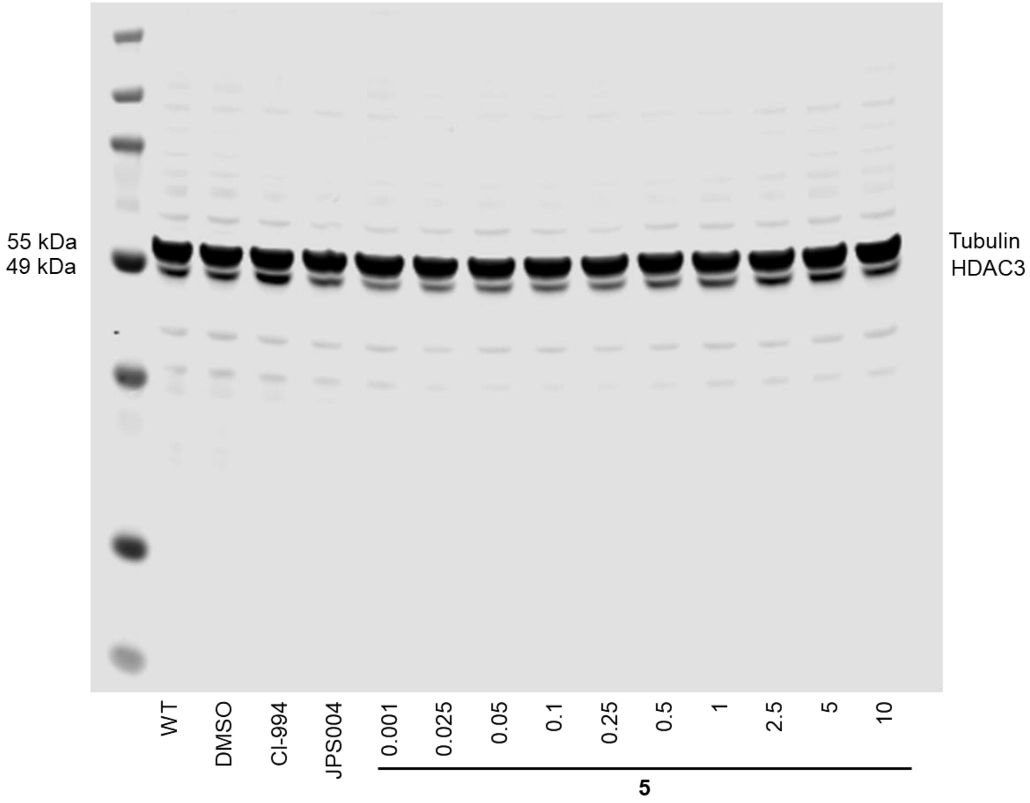
55 kDa
49 kDa
Tubulin
HDAC3
WT
DMSO
CI-994
JPS004
0.001
0.025
0.05
0.1
0.25
0.5
1
2.5
5
10
5

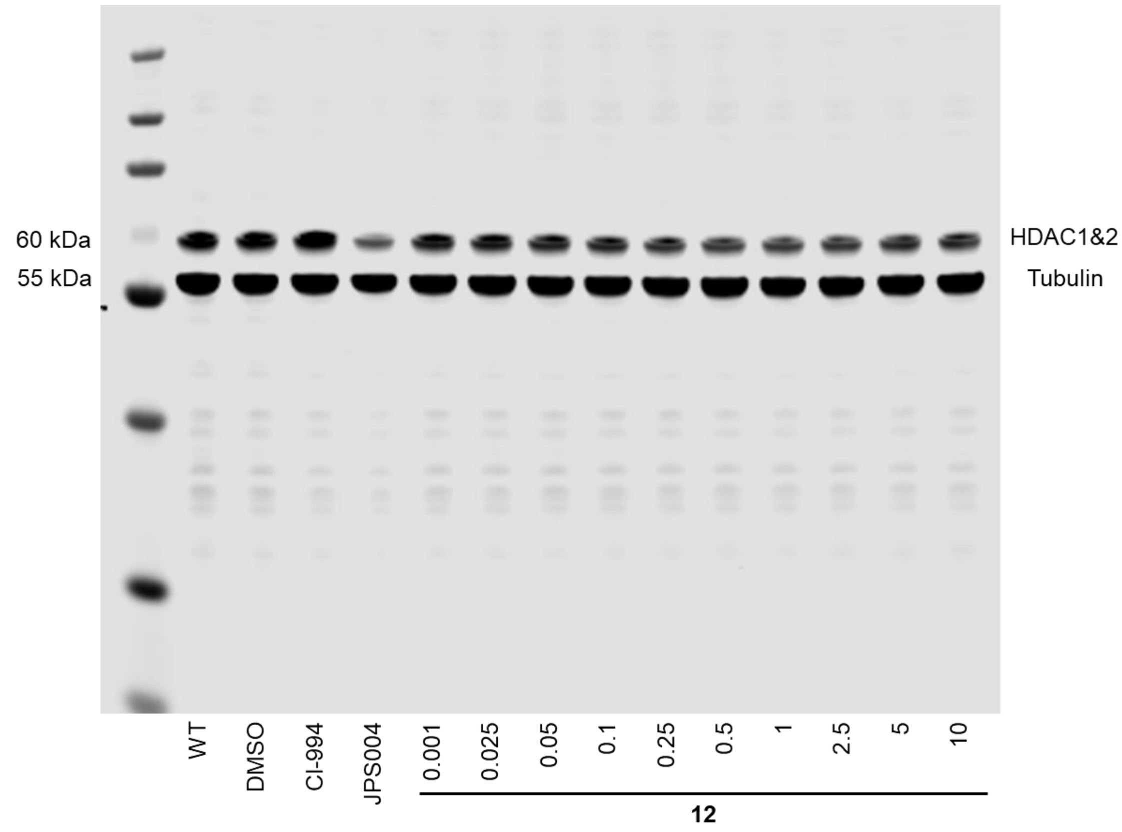
60 kDa
55 kDa
HDAC1&2
Tubulin
WT
DMSO
CI-994
JPS004
0.001
0.025
0.05
0.1
0.25
0.5
1
2.5
5
10
12

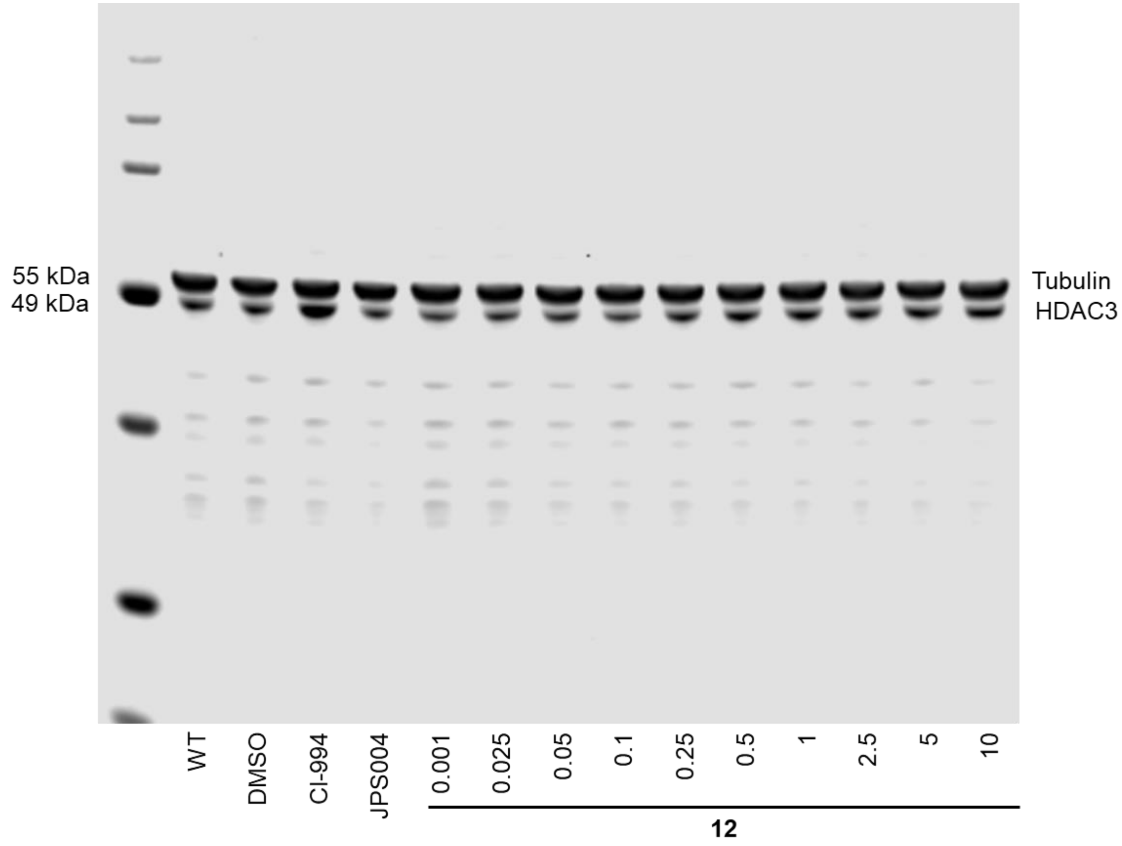
55 kDa
49 kDa
Tubulin
HDAC3
WT
DMSO
CI-994
JPS004
0.001
0.025
0.05
0.1
0.25
0.5
1
2.5
5
10
12

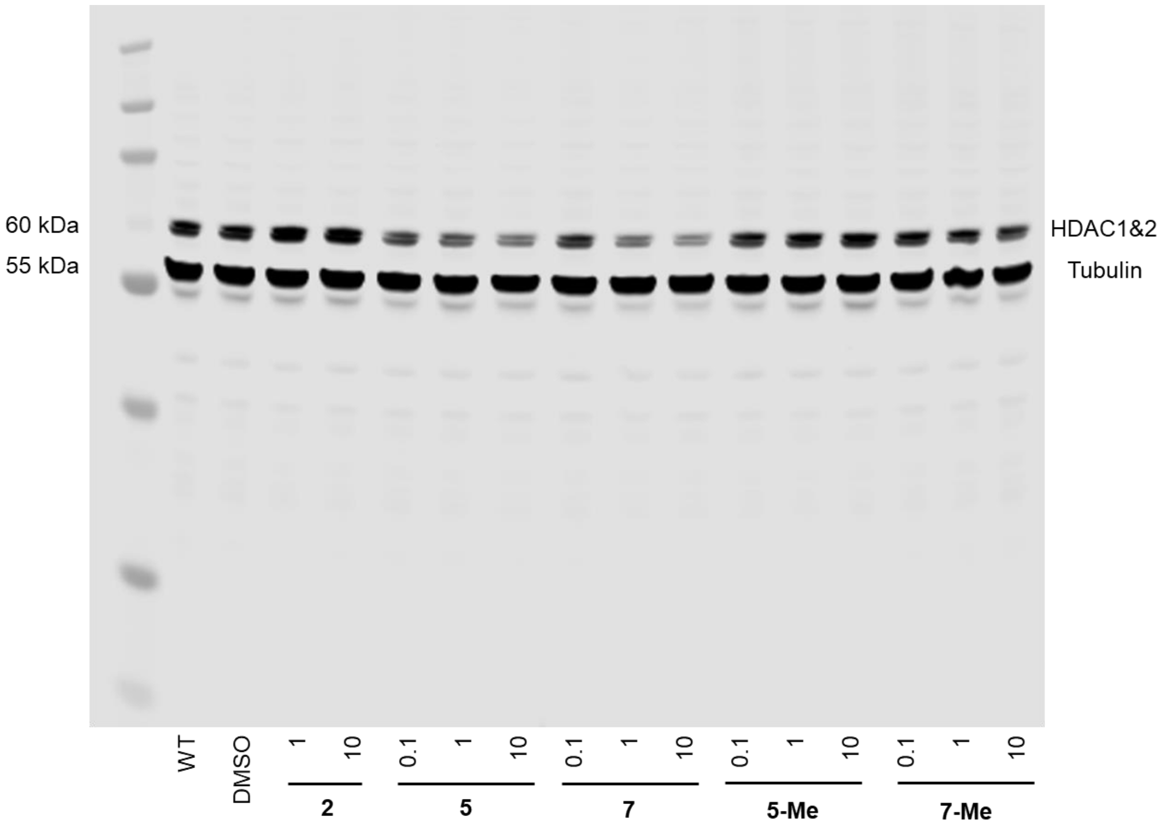
60 kDa
55 kDa
HDAC1&2
Tubulin
WT
DMSO
1
10
0.1
1
10
0.1
1
10
0.1
1
10
0.1
1
10
2
5
7
5-Me
7-Me

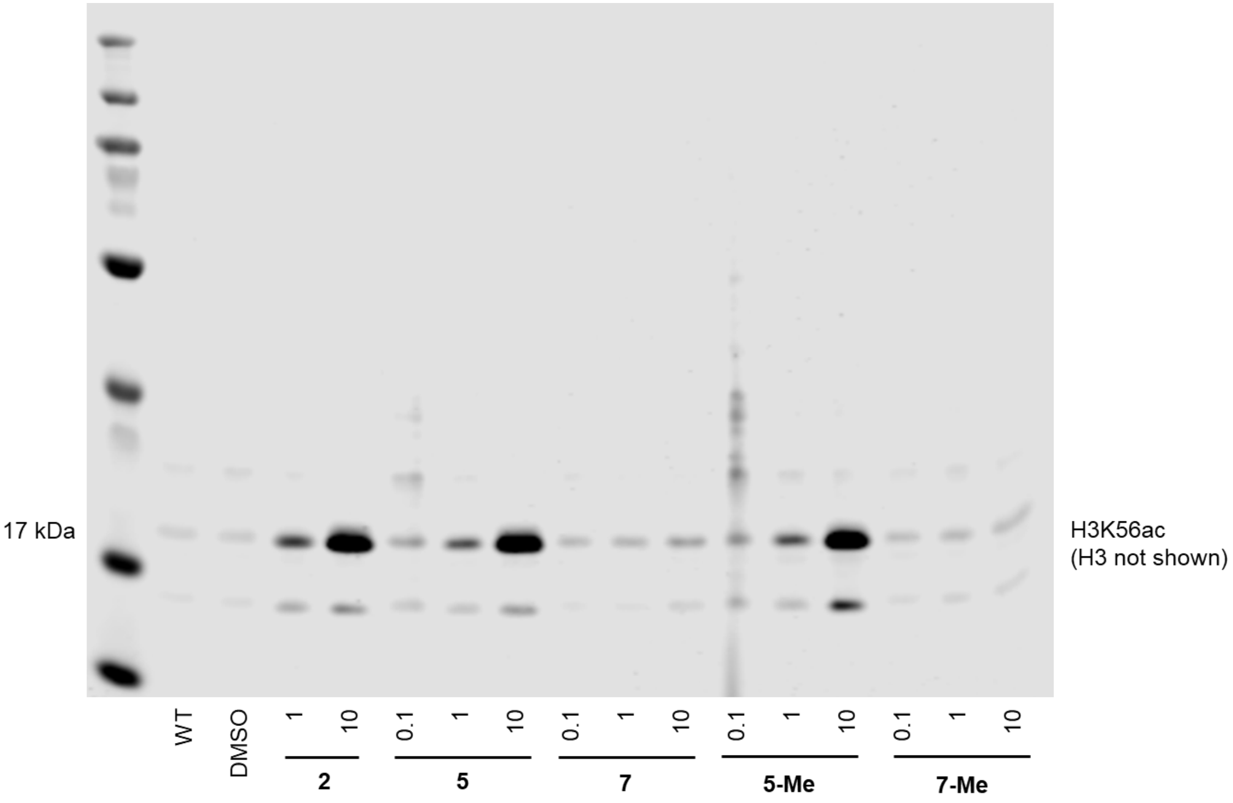
17 kDa
H3K56ac
(H3 not shown)
WT
DMSO
1
10
0.1
1
10
0.1
1
10
0.1
1
10
0.1
1
10
2
5
7
5-Me
7-Me
